# Single operation palladium catalysed C(sp^3^)–H functionalisation of tertiary aldehydes: investigations into transient imine directing groups†
†Electronic supplementary information (ESI) available: Experimental procedures and characterisation data; full optimisation table for arylation of pivaldehyde; studies on imine formation/hydrolysis in AcOD-*d*_4_; reaction plots with time for different directing groups and catalyst loading and addition of DMSO; effect of TFA addition; ^1^H NMR spectra of sample aldehyde regions; ^1^H and ^13^C NMR spectra for characterised compounds and crystal structure data. CCDC 1534094. For ESI and crystallographic data in CIF or other electronic format see DOI: 10.1039/c7sc01218g



**DOI:** 10.1039/c7sc01218g

**Published:** 2017-05-04

**Authors:** S. St John-Campbell, A. J. P. White, J. A. Bull

**Affiliations:** a Department of Chemistry , Imperial College London , South Kensington , London , SW7 2AZ , UK . Email: j.bull@imperial.ac.uk

## Abstract

Simple amine and diamine derivatives can promote the palladium catalysed direct β-C–H arylation of aliphatic aldehydes *via* transient imine formation. Trifluoroacetate was shown to be crucial in promoting the reaction. Sub-stoichiometric quantities of simple *N*-tosylethylenediamine was shown to form a bidentate directing group with an imine linkage. Isolation of an unsymmetrical palladacycle has shown different potential binding modes of the secondary NTs coordinating group by single crystal X-ray diffraction analysis, suggestive of a hemilabile ligand.

## Abstract 2

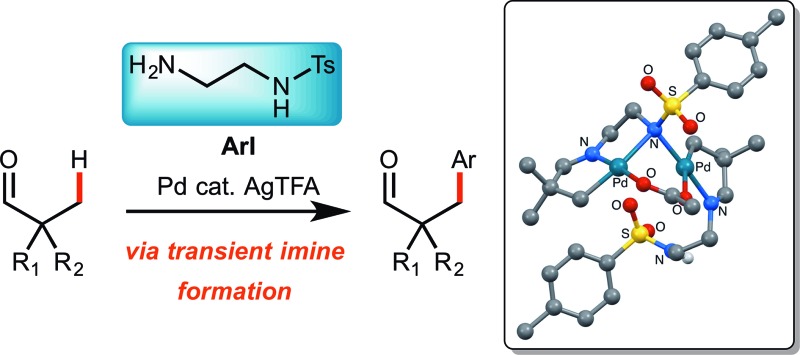
Mono and bidentate amine directing groups formed transiently though imine linkages promote C–H arylation of tertiary aldehydes.

## Introduction

Catalytic C–H functionalisation promises powerful strategies to derivatise otherwise inert sites of organic molecules.^1^ Using transition metal catalysts, in particular using palladium, this approach is becoming increasingly viable to streamline complex molecule synthesis. For C(sp^3^)–H functionalisation issues of low reactivity and regioselectivity are paramount. Several strategies have been described to control these issues, invoking an adjacent functional group to position a catalyst in an appropriate spatial arrangement to enable C–H activation,^2,3^ commonly through a concerted metalation–deprotonation mechanism.^4^

Daugulis,^5^ Yu,^6^ and others, made seminal advances in C(sp^3^)–H functionalisation through the use of amide linked bidentate and monodentate directing groups through Pd^0^/Pd^II^ and Pd^II^/Pd^IV^ redox cycles.^2^ The potential to cleave these groups from the substrate provided clear synthetic advantages, which encouraged the development of alternative directing groups to allow more facile removal.^7,8^ Nonetheless, these strategies require additional steps to install and remove the directing groups, which can be problematic and reduces the overall efficiency.

**Scheme 1:**
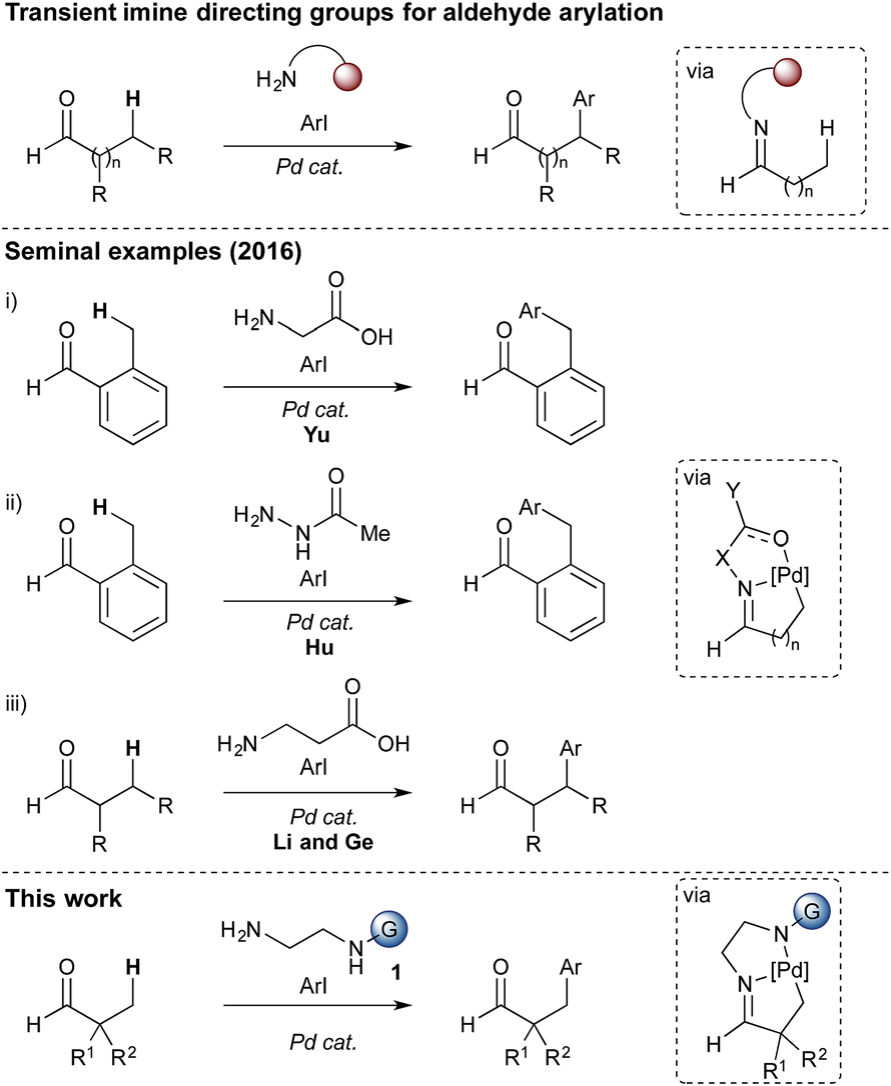
Transient directing groups for C–H arylation of aldehydes.

Very recently, and during the course of our investigations, the concept of transient directing groups has emerged, whereby the directing group is installed, C(sp^3^)–H functionalisation occurs, then the directing group is removed, all in one reaction pot (Scheme 1).^9^ The first report in this field, by Yu, described a transient imine linkage with α-amino acids to promote β-arylation of aliphatic ketones and benzylic arylation of *o*-tolualdehydes.^10^ A bidentate coordination was proposed involving the imine nitrogen and the carboxylate. A sub-stoichiometric amount of glycine was shown to be an effective promoter using a palladium catalyst and AgTFA, in a HFIP : AcOH solvent mixture. The use of l-*tert*-leucine afforded enantioselective arylation. Hu showed that an acetylhydrazone could act as a directing group for the arylation of *o*-tolualdehyde.^11^ Very recently Li and Ge reported the arylation of aliphatic aldehydes, *via* a 5,6-palladacyclic intermediate, using a β-amino acid to form an imine linked directing group.^12^

Dong,^13^ Ge,^14^ Murakami,^15^ and Yu^16^ also recently independently demonstrated the direct arylation of aliphatic amines, using aromatic or conjugated aldehydes to form directing groups incorporating the reverse imine. Yu has reported the *ortho*-C(sp^2^)–H functionalisation of benzaldehydes using transient directing groups to install various functional groups.^17,18^

Here we report investigations into a new class of transient, sub-stoichiometric directing group for single step β-C(sp^3^)–H arylation of tertiary aldehydes using palladium catalysis and aryl iodides. Over 25 different mono- and bidentate directing groups are investigated either as preformed imines or through *in situ* reversible imine formation with simple amines (**1**), with many successful in mediating the reaction. Some mechanistic studies are also described, including isolation of a palladacycle as an unusual unsymmetrical dimer, characterized by single crystal X-ray diffraction.

## Results and discussion

**Fig. 1:**
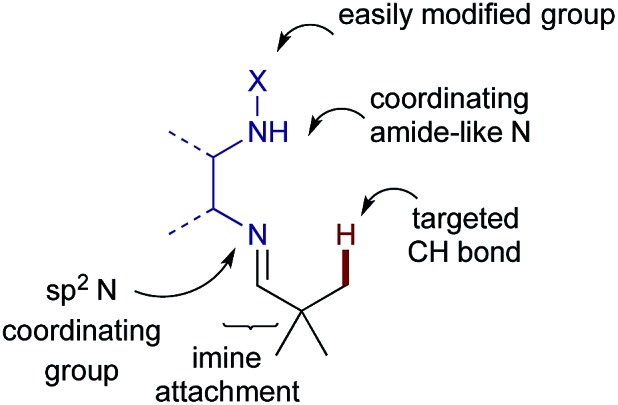
Proposed directing group structure.

At the outset of our investigation we envisaged that a reversible imine formation could transiently introduce directing functionality to promote C–H arylation on aldehyde substrates using simple primary amines. To mimic many of the successful bidentate amide-linked directing groups with a Pd^II^/Pd^IV^ catalytic cycle, we proposed moving the amide or related functional groups to a distal position, presenting a reversal of the *N*,*N*′-coordinating functionality (Fig. 1).^19^ Such groups would be easily prepared from ethylenediamine and we anticipated that simple modification of the *exo*-amide group would be suitable to tune the properties of the directing group.

**Scheme 2:**
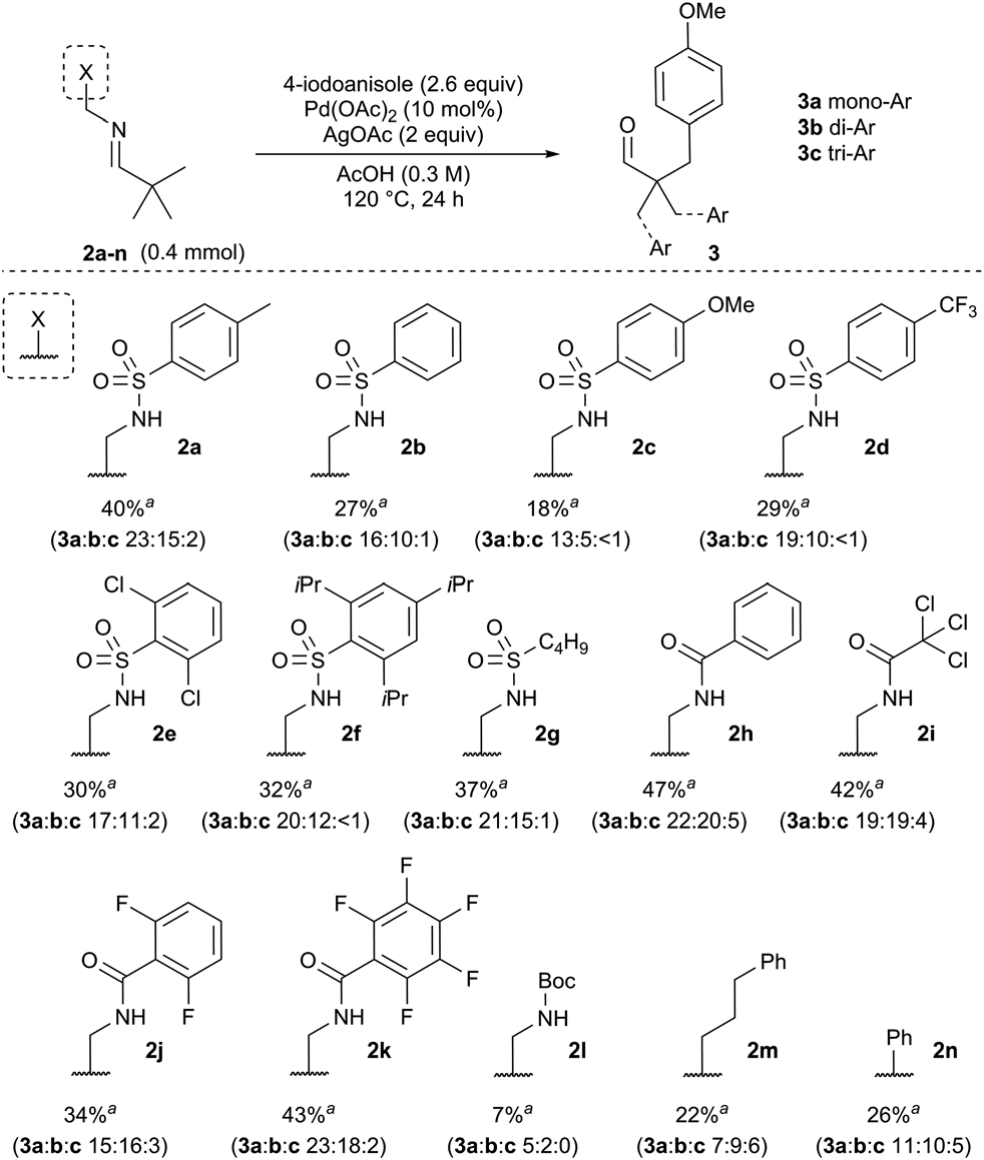
Screen of bidentate and monodentate directing groups for the arylation of pivaldehyde derived imines. ^*a*^Yields quoted as combined yield for **3a–c**, with separate mono-, di- and triarylated yields quoted below. Yields determined by ^1^H NMR using 1,3,5-trimethoxybenzene as an internal standard.

To establish the viability of the C–H arylation and to design the distal coordinating group, we initially preformed a series of imines from pivaldehyde and primary amines. These were designed to offer potential bidentate coordination through varied sulfonamides (**2a–g**), amides (**2h–k**) and a carbamate (**2l**) (Scheme 2). Simple alkyl and benzyl examples (**2m,n**) which could form only monodentate directing groups were also prepared. We initially subjected preformed imines to common arylation conditions using 4-iodoanisole.^5,7,20^ A low yield was obtained in the absence of solvent [*e.g.* 8% yield for **2a**], and although various other solvents were unsuccessful an effective arylation was obtained in acetic acid.^21^

After preliminary optimisation, various imines were subjected to the arylation conditions, using Pd(OAc)_2_ and AgOAc in acetic acid at 0.3 M concentration, over 24 h (Scheme 2). The screen revealed that both the amide and the sulfonamide containing imines could successfully direct the C–H arylation. After a simple work up, involving a filtration through silica, only the hydrolysed aldehyde product **3** was observed, with no residual imine **2**. A mixture of mono-, di- and tri-arylated products were obtained (**3a–c**).

Sulfonamide-containing imine **2a** gave the best result, with a 40% combined yield of aldehydes **3a–c**. Various other sulfonamides, with differing steric and electronic properties, gave similar yields. Generally, the amide-containing directing groups showed higher reactivity than the sulfonamides, with amide containing imine **2h** giving the highest yield of aldehydes **3a–c**. However, these gave both poorer selectivity of the monoarylated product, and also formed more highly functionalised aldehydes, giving overall a more complex mixture of products.^20^ Carbamate derivative **2l** showed much reduced yield though was presumably unstable under the reaction conditions. Interestingly, amines which formed monodentate directing groups also promoted arylation, with benzylamine **2n** affording a 26% yield. This highlighted that secondary binding was not crucial, but did enhance the yield. Indeed, the similarity across the different amines examined suggests that the secondary binding site may not be continuously bound to the metal centre throughout the catalytic cycle.

To realise a one-pot arylation of pivaldehyde, a 1 : 1 ratio of aldehyde and directing group was used in place of imine **2a** (Table 1). Unfortunately, under the conditions used above, only trace product **3** was observed (entry 1). Therefore, we investigated the reaction parameters initially using a stoichiometric quantity of amine **1a** and Pd(OAc)_2_ at 10 mol%. Importantly, on changing the silver source to AgTFA a 29% yield was achieved (entry 2). A wide range of solvents were investigated, along with solvent mixtures.^20^ The presence of acetic acid was critical, and various solvents were tolerated when used in combination with AcOH. The yield was improved when using hexafluoroisopropanol (HFIP) as a co-solvent.^22^ Similar yields were obtained with different AcOH : HFIP ratios and we chose to progress with a 1 : 1 mixture for maximum reproducibility (entry 3). HFIP alone as a solvent was ineffective (entry 4). Using TFA as a co-solvent with acetic acid was also a slight improvement on acetic acid alone. Varying the palladium pre-catalyst gave a successful reaction with PdCl_2_ and an improvement with Pd(OPiv)_2_ to a 47% yield (entry 7).

**Table 1:** Optimisation of aldehyde arylation with a transient directing group

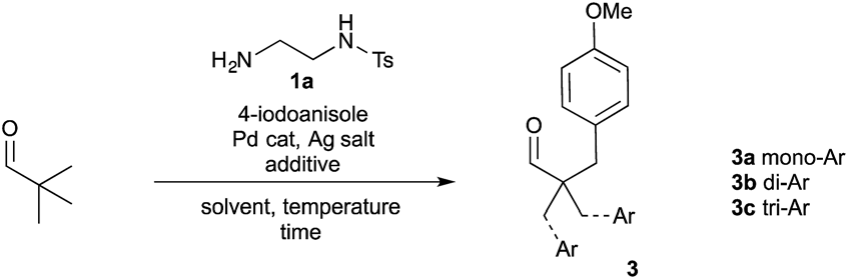					
Entry^*a*^	Solvent	Pd cat.	Ag salt	Yield^*b*^ (%) **3a** : **3b** : **3c**	Yield **3** combined (%)
1	AcOH	Pd(OAc)_2_	AgOAc	Trace	Trace
2	AcOH	Pd(OAc)_2_	AgTFA	15 : 11 : 3	29
3	HFIP : AcOH (1 : 1)	Pd(OAc)_2_	AgTFA	20 : 13 : 3	36
4	HFIP	Pd(OAc)_2_	AgTFA	9 : 0 : 0	9
5	TFA : AcOH (1 : 3)	Pd(OAc)_2_	AgTFA	20 : 13 : 1	34
6	HFIP : AcOH (1 : 1)	PdCl_2_	AgTFA	19 : 13 : 4	36
7	HFIP : AcOH (1 : 1)	Pd(OPiv)_2_	AgTFA	23 : 17 : 7	47
8^*c*^	HFIP : AcOH (1 : 1)	Pd(OPiv)_2_	AgTFA	22 : 15 : 8	45
9^*c*^ ^,^ ^*d*^	HFIP : AcOH (1 : 1)	Pd(OPiv)_2_	AgTFA	22 : 18 : 8	46
10^*c*^ ^,^ ^*d*^ ^,^ ^*e*^	HFIP : AcOH (1 : 1)	Pd(OPiv)_2_	AgTFA	29 : 19 : 8	56
**11** ^*c*^ ^,^ ^*d*^ ^,^ ^*e*^ ^,^ ^*f*^	**HFIP : AcOH (1 : 1)**	**Pd(OPiv)**_**2**_	**AgTFA**	**29 : 22 : 10**	**61**
12^*d*^ ^,^ ^*e*^ ^,^ ^*f*^ ^,^ ^*g*^	HFIP : AcOH (1 : 1)	Pd(OPiv)_2_	AgTFA	28 : 20 : 9	57
13^*d*^ ^,^ ^*e*^ ^,^ ^*f*^ ^,^ ^*h*^	HFIP : AcOH (1 : 1)	Pd(OPiv)_2_	AgTFA	26 : 16 : 5	47

^*a*^Reaction conditions: pivaldehyde (0.2 mmol), **1a** (1 equiv.), 4-iodoanisole (2.6 equiv.), Pd catalyst (10 mol%), silver salt (2 equiv.), 0.3 M, 120 °C, 24 h, unless otherwise stated.

^*b*^Yields determined by ^1^H NMR using 1,3,5-trimethoxybenzene as an internal standard.

^*c*^Reaction performed using 0.5 equiv. **1a**, 5 mol% Pd(OPiv)_2_.

^*d*^3 h reaction time.

^*e*^Added DMSO (1 equiv.).

^*f*^Reaction performed at 130 °C, and 0.5 M concentration.

^*g*^0.25 equiv. **1a**, 5 mol% Pd(OPiv)_2_.

^*h*^0.10 equiv. **1a**, 5 mol% Pd(OPiv)_2_.

Pleasingly, the loadings of both amine **1a** and Pd(OPiv)_2_ could be lowered to 0.5 equiv. and 5 mol% respectively without affecting the reaction yield (entry 8). Notably, an almost identical reaction profile over time was observed under these conditions (entries 7 and 8; and ESI†).^20^ The reaction was rapid, reaching maximum conversion within 3 h (entry 9). At this stage, numerous additives were investigated; while many Lewis basic additives were tolerated, the maximum increase in yield was on addition of DMSO, with 1 equiv. optimal (entry 10).^20,23^ A 61% overall yield of arylated products was obtained by performing the reaction at 130 °C and 0.5 M concentration. Finally, further reduction in the loading of amine **1a** to 0.25 and 0.1 equiv. was well tolerated, resulting in only a marginal reduction in yield (57% and 47% respectively).

To highlight the crucial role of trifluoroacetate to the reaction conditions,^24^ we directly compared the effect of TFA additives (Table 2).

**Table 2:** Effect of TFA additives on the arylation of pivaldehyde using **1a**

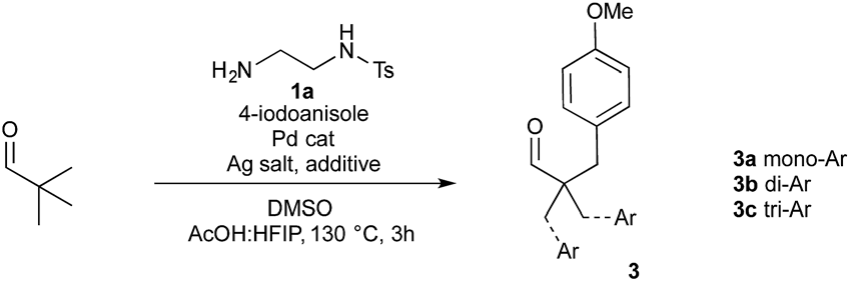					
Entry^*a*^	Pd cat.	Ag salt	Additive	Yield^*b*^ (%) **3a** : **3b** : **3c**	Yield **3** combined (%)
1	Pd(OAc)_2_	AgOAc	—	2 : 0 : 0	2
2	Pd(OAc)_2_	AgTFA	—	26 : 19 : 9	54
3	Pd(OAc)_2_	AgOAc	TFA^*c*^	21 : 17 : 9	47
4	Pd(TFA)_2_	AgOAc	—	9 : trace : 0	9

^*a*^Conditions: pivaldehyde (0.2 mmol), **1a** (0.5 equiv.), 4-iodoanisole (2.6 equiv.), Pd cat (5 mol%), Ag salt (2 equiv.), DMSO (1 equiv.), HFIP : AcOH (1 : 1, 0.5 M), 130 °C, 3 h.

^*b*^Yields determined by ^1^H NMR using 1,3,5-trimethoxybenzene as an internal standard.

^*c*^2 equiv.

Using the optimised conditions, but with palladium acetate and AgOAc, only a trace amount of aldehyde **3** was formed (entry 1). Using AgTFA (2 equiv.) in place of AgOAc returned the yield to 54%. Interestingly, when using AgOAc with trifluoroacetic acid (2 equiv.) as an additive a 47% yield could still be achieved, despite the large excess of acetate from the solvent.^25^ Using Pd(TFA)_2_ (5 mol%; comparable to 0.1 equiv. of TFA) gave a 9% yield of monoarylated aldehyde **3a**. In contrast, in the arylation of the preformed imines the presence of TFA in the reaction mixture was not a requirement. Indeed, running the arylation on imine **2a** with AgTFA gave the same yield as using AgOAc. Given the lower concentration of imine in the one-pot process,^21^ a more electrophilic Pd(TFA)X species may improve coordination to the imine and overall facilitate the CMD process.

**Scheme 3:**
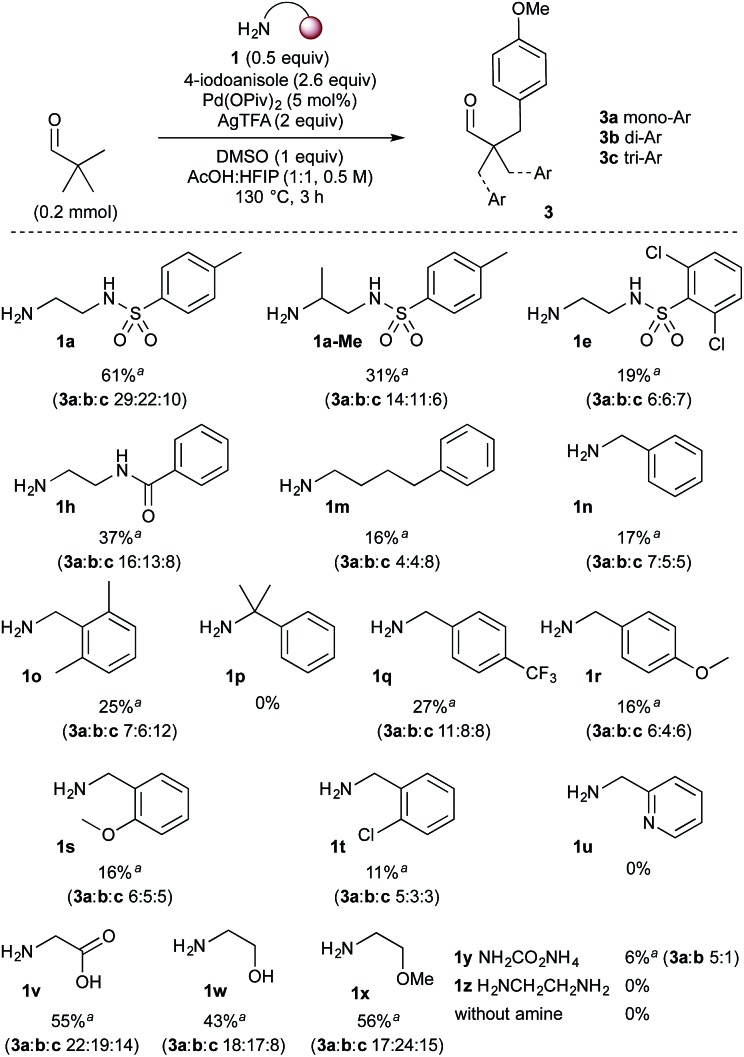
Screen of bidentate and monodentate directing groups for the direct arylation of pivaldehyde. Reaction conditions: pivaldehyde (0.2 mmol), 4-iodoanisole (2.6 equiv.), HFIP : AcOH (1 : 1, 0.5 M). ^*a*^Yields quoted as combined yield for **3a–c**, with separate mono-, di- and triarylated yields quoted below. Yields determined by ^1^H NMR using 1,3,5-trimethoxybenzene as an internal standard.

With the highest yielding conditions (Table 1, entry 11), various mono- and bidentate directing groups were investigated, to further explore potential directing group design. Remarkably, the vast majority promoted the arylation to some extent (Scheme 3). *N*-Tosylethylenediamine **1a** continued to provide the best directing group.^26^ Adding a methyl group adjacent to the primary amine gave a decreased yield, presumably due to reduced imine formation. Sulfonamide **1e** and amide **1h**, which gave similar yields in the preformed imines, were less successful than **1a**. The monodentate directing groups gave generally lower yields. Several benzylamine derivatives were investigated (**1n–u**), including those with secondary coordinating groups (**1s–u**), for which the best yield of 27% was with 4-trifluoromethylbenzylamine **1q**. No arylation occurred with hindered derivative **1p**. 2-Picolylamine appeared to directly complex with palladium and was unsuccessful in the reaction. Other simple bidentate directing groups were also examined. Under these conditions glycine **1v**, as used by Yu,^10^ formed the arylated product in 55% yield with an increased proportion of the tri and diarylated products. Amino alcohol **1w** and 2-methoxyethan-1-amine **1x** gave good yields, also with a greater tendency to form di- and triarylated aldehydes. A simple NH imine formed from ammonium carbamate **1y** also gave the arylated product, though in very low yield. Using ethylenediamine itself (**1z**) also gave no product formation, most likely due to coordination to the Pd catalyst. Crucially with no amine added, there was no evidence of product formation.

**Scheme 4:**
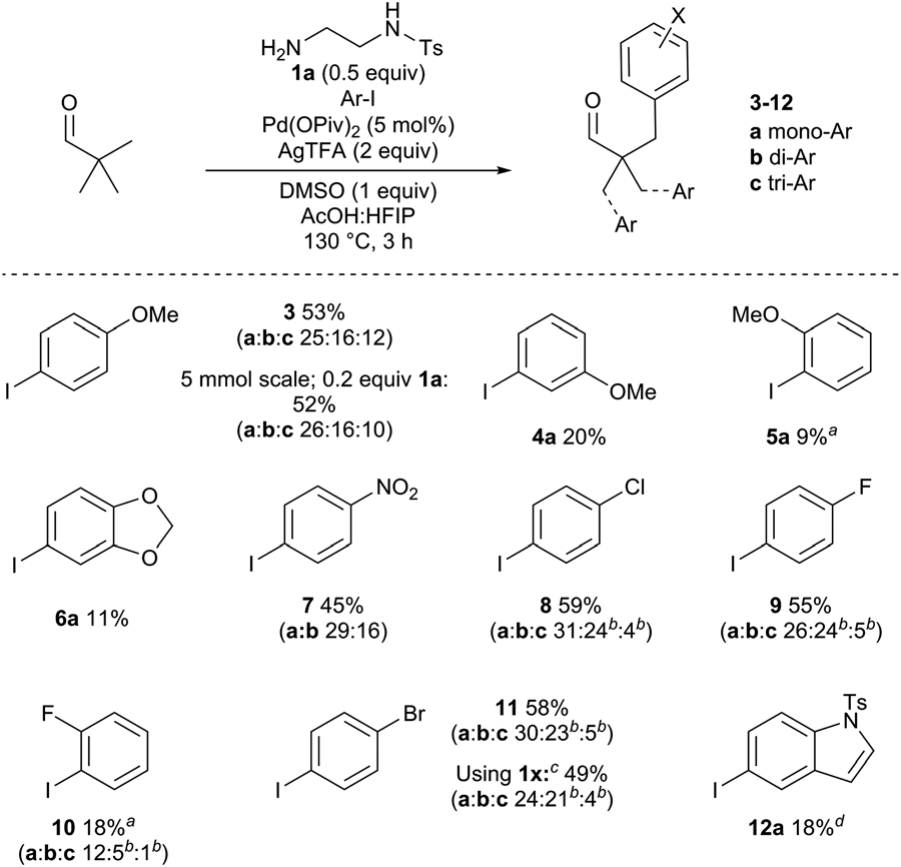
Scope of aryl iodides to form aldehydes **3–12**. Reaction conditions: pivaldehyde (0.4 mmol), **1a** (0.5 equiv.), Ar-I (2.6 equiv.), HFIP : AcOH (1 : 1, 0.5 M). Yields correspond to sum of yields of isolated products, with individual yields a–c given below. ^*a*^Reaction time of 24 h. ^*b*^Isolated as a mixture of di- and tri-arylated products. ^*c*^Using **1x** in place of **1a**. ^*d*^Reaction time of 6 h.

Next, the steric and electronic requirements of the aryl iodide coupling partner were explored using pivaldehyde and amine **1a** (Scheme 4). Under the optimised conditions, 4-iodoanisole afforded a 53% combined isolated yield of the separated mono-, di- and triarylated aldehydes **3a–c**. While arylation occurred at each CH_3_ group, there was no evidence of arylation at the benzylic methylene position under these conditions. The reaction was performed on a larger scale with a reduced amine loading (5 mmol scale, 0.2 equiv. **1a**). The reaction efficiency was maintained, providing arylated aldehyde **3** in 52% yield. The *meta*- and importantly the *ortho*-iodoanisole examples were viable, albeit with sequentially lower yields of **4** and **5** respectively due to increased steric demands. Using 5-iodo-1,3-benzodioxole gave a low yield of arylated product **6a**. The electron poor 1-iodo-4-nitrobenzene gave 45% of **7**, along with small amounts of the homo-dimer of the aryl iodide. *para*-Chloro- and fluoro-iodobenzenes gave good yields of aldehydes **8** and **9**, and the *ortho*-fluoro example gave 18% yield aldehyde 10. *para*-Bromoiodobenzene was run with both **1a** and **1x** as the directing group to form **11**. Although the yields for both directing groups were similar (within 10%), **1x** gave a higher proportion of diarylation. It is likely that for pivaldehyde multiple arylations may occur without exiting the catalytic cycle after the monoarylation, the extent of which is affected by the structure the directing groups.^27^ Pyridyl and thienyl iodides were not well tolerated under the reaction conditions (<10% yield). However, a 5-iodoindole derivative was successful to afford **12a** in 18% yield.

**Scheme 5:**
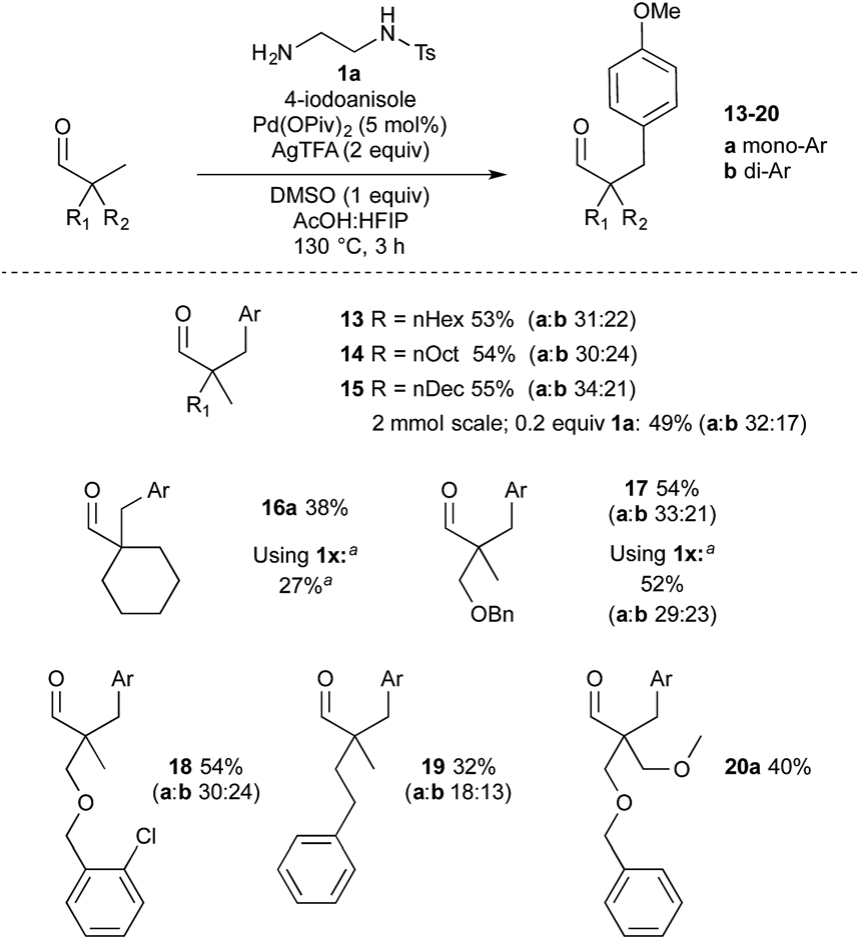
Reaction scope varying the aldehyde. Ar = C_6_H_4_OMe. Reaction conditions: aldehyde (0.4 mmol), **1a** (0.5 equiv.), 4-iodoanisole (2.6 equiv.), HFIP : AcOH (1 : 1, 0.5 M). Yields correspond to sum of yields of isolated products, with individual yields a and b given below. ^*a*^Using **1x** in place of **1a**.

**Scheme 6:**
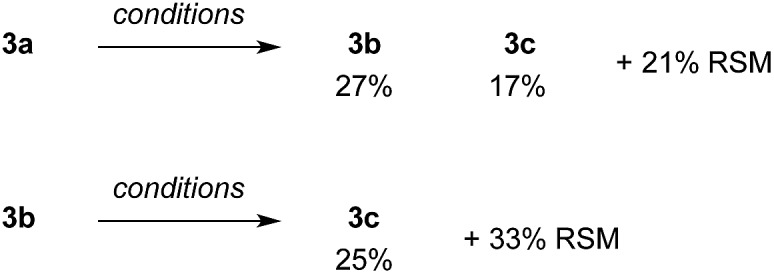
Arylation of **3a** and **3b**. Conditions: **3a** (0.099 mmol) or **3b** (0.064 mmol), **1a** (0.5 equiv.), 4-iodoanisole (2.6 equiv.), Pd(OPiv)_2_ (5 mol%), AgTFA (2 equiv.), DMSO (1 equiv.), HFIP : AcOH (1 : 1, 0.5 M), 130 °C, 3 h. Yields determined by ^1^H NMR using 1,3,5-trimethoxybenzene as an internal standard.

The aldehyde component was then investigated (Scheme 5). Higher molecular weight aldehydes bearing an alkyl chain, gave consistent yields around 55% of arylated products **13–15**. The reaction to form aldehyde **15** was also performed on a larger scale using a lower loading of amine **1a** (0.2 equiv.), which gave a comparable result. Cyclohexyl methyl example **16a** was successful with both **1a** and **1x** directing groups affording 38% and 27% monoarylation respectively. Benzyl and 2-chlorobenzyl ether examples **17** and **18** were formed in good yield. Benzyl ether **17** was also formed using 2-methoxy-1-ethylamine **1x**, which gave a comparable yield to **1a**, again with an increased proportion of diarylation. Phenethyl example **19** was formed in a 32% total yield, and bis-ether-containing aldehyde **20** in 40% yield. Reapplying monoarylated aldehyde **3a** and diarylated **3b** to the reaction conditions afforded the corresponding further arylated aldehydes (Scheme 6) with some recovered substrate. Secondary centres, and aldehydes with acidic α-hydrogen atoms were not cleanly arylated under these conditions.

**Scheme 7:**
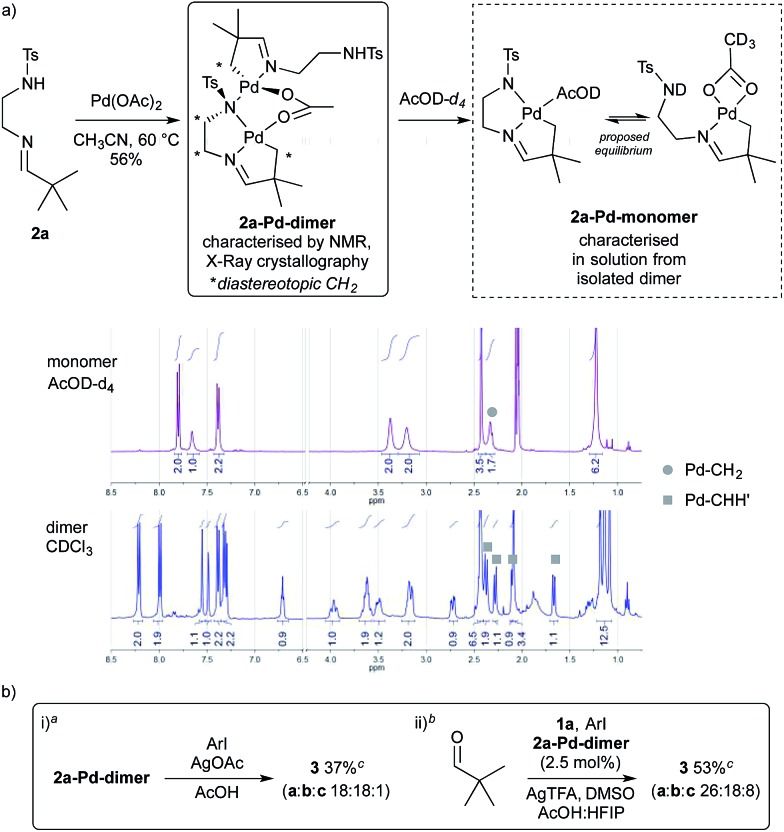
Formation and reactions of **2a-Pd-dimer**. ^*a*^Conditions: **2a-Pd-dimer** (0.04 mmol), 4-iodoanisole (6.0 equiv.), AgOAc (4.0 equiv.), AcOH (0.3 M), 120 °C, 24 h. ^*b*^Conditions: pivaldehyde (0.2 mmol), **1a** (0.5 equiv.), 4-iodoanisole (2.6 equiv.), **2a-Pd-dimer** (2.5 mol%), AgTFA (2.0 equiv.), HFIP : AcOH (1 : 1, 0.5 M), 130 °C, 3 h. ^*c*^Yields determined by ^1^H NMR using 1,3,5-trimethoxybenzene as an internal standard.

To gain some insight into the role of the sulfonamide as a secondary coordinating group, we formed a palladacycle from imine **2a** by reaction with palladium acetate in MeCN (Scheme 6). The reaction was diluted with toluene and filtered through Celite to give a 56% yield of the palladacycle, a sample of which was further purified by recrystallisation. By ^1^H NMR this gave complex signals indicative of an unsymmetrical dimeric structure, containing different imine environments and several diastereotopic CH_2_ signals, as well as one NH signal with an integral of 1H (Scheme 7a).

**Fig. 2:**
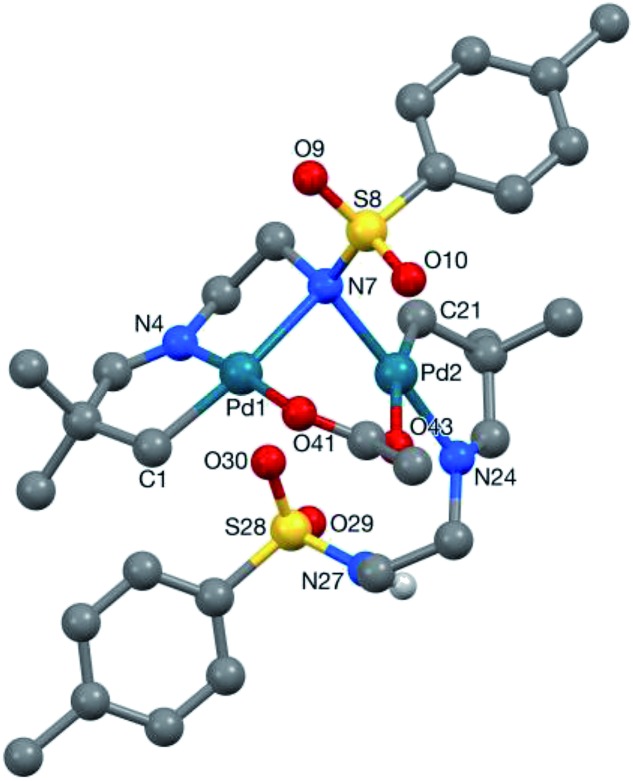
Crystal structure of **2a-Pd-dimer**. Selected bond lengths (Å), Pd1–C1 2.009(3), Pd1–N4 1.952(3), Pd1–N7 2.253(3), Pd1–O41 2.046(2), Pd2–N7 2.085(3), Pd2–C21 2.003(3), Pd2–N24 2.018(3), Pd2–O43 2.244(2). The Pd1···Pd2 separation is 3.1098(3) Å.

It was clear that the sulfonamide may display different coordination modes in its role as a secondary coordinating group.^28^ Pleasingly we obtained the crystal structure of **2a-Pd-dimer**, proving the palladacycle structure, which indeed showed the two sulfonamides adopted very different binding modes (Fig. 2).^29^ The first directing group (C1-based ligand) binds to the palladium centre (Pd1) in a tridentate *C*,*N*,*N*′ fashion utilising C1, the imine nitrogen N4, and the sulfonamide nitrogen anion (N7), the latter of which also binds to Pd2.^30,31^ This binding mode is consistent with the imine and sulfonamide acting as a bidentate directing group. By contrast, the other imine (C21-based ligand), coordinates to Pd2 through C21 and the imine nitrogen (N24) only. The NH-sulfonamide was retained, and not coordinated to Pd.^32^ The coordination sphere at each metal centre was completed by a bridging acetate group. Each metal centre has a slightly distorted square planar coordination geometry, consistent with the Pd^II^ oxidation state.^33^ The two Pd-coordination planes are steeply inclined with respect to each other [82.63(7)°] such that the Pd1···Pd2 separation is 3.1098(3) Å.

When **2a-Pd-dimer** was dissolved in AcOD a monomeric species **2a-Pd-monomer** was formed, which was characterised in solution by ^1^H and ^13^C NMR. Due to the broadening of the directing group CH_2_ signals (3.38 and 3.20 ppm) we speculated that there may be an equilibrium between the free and coordinated sulfonamide group, complemented by changing acetate coordination (Scheme 7a). Furthermore, monitoring the reaction between **2a** and palladium acetate *in situ* by ^1^H NMR in MeCN-*d*_3_ suggested a monomeric palladacycle was formed in solution.^34^ This suggests that a monomeric species would be dominant under the reaction conditions.

Reacting **2a-Pd-dimer** with 4-iodoanisole in AcOH, afforded a 37% yield of arylated aldehyde **3** (Scheme 7b,i). Importantly, using **2a-Pd-dimer** (2.5 mol%, *i.e.* 5% Pd) as the catalyst for the arylation of pivaldehyde under the optimised conditions, gave 53% yield of aldehyde **3**, in the presence of additional 0.5 equiv. **1a** (Scheme 7b,ii).^35^ These results support the palladacycle monomer being an intermediate in the reaction.

On the basis of these observations, and previous reports, we propose a Pd^II^/Pd^IV^ mechanism with coordination of an electrophilic Pd(TFA)X species to a transiently formed imine, followed by palladacycle formation, likely through a CMD mechanism. Oxidative addition of the Pd^II^-palladacycle into the aryl iodide followed by reductive elimination forms the C–C bond. The Pd^II^ species may decomplex and re-enter the catalytic cycle, or remain bound to the imine and undergo arylation again. The imine is then readily hydrolysed to the aldehyde to regenerate the directing group. The binding modes of the N–Ts groups in **2a-Pd-dimer**, and the reactivity when using monodentate directing groups, suggests that the secondary binding site is not likely to be continuously bound to the catalyst while bound to the imine.

## Conclusions

In summary, we have described a palladium catalysed β-C(sp^3^)–H functionalisation on tertiary aldehyde substrates with aryl iodides, assisted by simple bidentate and monodentate primary amine derivatives. We have shown a wide variety of simple amines and diamine derivatives, used in sub-stoichiometric quantities, form transient imine linkages as directing groups to promote aldehyde C–H arylation, in a single synthetic operation. Arylation protocols have been developed both for preformed imine species as well as through transient imine formation.

Studies using a new directing group, derived from *N*-tosylethylenediamine **1a**, have highlighted crucial factors in the success of the transient directing group. The reaction required small quantities of TFA, up to 2 equivalents, even in an acetic acid solvent, to promote the arylation of pivaldehyde. The presence of the second N–Ts coordination site, resulted in increased yields; a general observation with the potentially bidentate groups. However, it is likely that the second site is not coordinated to Pd throughout the catalytic cycle. The structure of a palladacycle formed from imine **2a** was an unusual unsymmetrical dimer in the solid state, as confirmed by single crystal X-ray diffraction, where only one of the two N–Ts groups was coordinated to Pd. It is likely the monomeric form constitutes an intermediate in the reaction, presumed to be a Pd^II^/Pd^IV^ cycle. The isolated palladacyclic dimer provided an effective catalyst for the reaction of pivaldehyde with 4-iodoanisole. The aldehyde arylation using *N*-tosylethylenediamine **1a** was effective on a variety of aldehyde substrates and aryl iodide coupling partners with various steric and electronic properties. This study offers insight we expect to be valuable in the future development of directing groups to be formed transiently to promote C–H functionalisation. We are now examining the use of imine-linked directing groups for the functionalisation of unactivated C(sp^3^)–H bonds toward pharmaceutically relevant compounds.
