# Supplementary material for: Single operation palladium catalysed C(sp3)–H functionalisation of tertiary aldehydes: investigations into transient imine directing groups
Source: Chem Sci. 2017 May 4;8(7):4840–7. doi: 10.1039/c7sc01218g (PMC5603794; doi:10.1039/c7sc01218g)
Supplement: Supplementary file 1 [file SC-008-C7SC01218G-s001.pdf]

Electronic Supplementary Material (ESI) for Chemical Science.
This journal is © The Royal Society of Chemistry 2017

SUPPORTING INFORMATION

Single Operation Palladium Catalysed C(sp^3)–H Functionalisation of Tertiary Aldehydes: Investigations into Transient Imine Directing Groups

Sahra St John-Campbell, Andrew J. P. White and James A. Bull*
Department of Chemistry, Imperial College London, South Kensington, London SW7 2AZ, UK

*E-mail: j.bull@imperial.ac.uk

Table of Contents

Table of Contents S02
General Experimental Considerations S03
Extended optimisation table for arylation of pivaldehyde with a transient directing group S04
Reaction profiles for decreased loading of **1a** / catalyst and addition of DMSO additive S05
Effect of TFA equivalents on reaction yield S06
Imine formation and hydrolysis in AcOD-*d*$_4$ S07
Experimental procedures and characterisation data S10
- Preparation of directing groups (**1a-k**) S10
- Preparation of imines (**2a-n**) S15
- C(sp^3)–H arylation of imines S20
- Directing group selection S20
- Preparation of aldehydes (**S5-S22**) S21
- C(sp^3)–H arylation of aldehydes with a transient directing group (**3-20**) S27
- Synthesis and arylation of palladacycle dimer (**2a-Pd-dimer**) S41
- Formation of palladacycle monomer (**2a-Pd-monomer**) as a solution in AcOD-*d*$_4$ S42

NMR study of the formation of palladacycle monomer in MeCN-*d*$_3$ S43
X-Ray crystal structure of palladacycle dimer (**2a-Pd-dimer**) S44
References S47
^{1}H and ^{13}C NMR spectra for selected compounds S48

General Experimental Considerations

All nonaqueous reactions were run under an inert atmosphere (argon) with flame-dried glassware using standard techniques. Anhydrous solvents were obtained by filtration through drying columns (THF, diethyl ether, CH_2Cl_2). Acetic acid, AgOAc and all palladium catalysts were purchased from Sigma Aldrich and used as provided. Hexafluoroisopropanol and AgTFA were purchased from Fluorochem and used as provided. Commercial aldehydes were used as provided or distilled over CaH_2. The purity of the aldehyde had a significant effect on reaction yield. All other commercial reagents were used as supplied or purified by standard techniques where necessary. Reactions were performed in microwave vials sealed with Fisherbrand 20 mm aluminium, plain, centre hole, moulded septa butyl, dark grey, 55° shore A, 3.0 mm caps.

Flash column chromatography was performed using 230-400 mesh silica with the indicated solvent system according to standard techniques. Analytical thin-layer chromatography (TLC) was performed on precoated, glass-backed silica gel plates. Visualisation of the developed chromatogram was performed by UV absorbance (254 nm), aqueous potassium permanganate, *p*-anisaldehyde, phosphomolybdic acid or vanillin stains.

Infrared spectra (ν_{max}, FTIR ATR) were recorded in reciprocal centimeters (cm^{-1}). Nuclear magnetic resonance spectra were recorded on 400 MHz spectrometers. Chemical shifts for ^1H NMR spectra are recorded in parts per million from tetramethylsilane with the solvent resonance as the internal standard (chloroform δ = 7.27 ppm, acetic acid δ = 7.04 ppm or DMSO δ = 2.50 ppm). Data is reported as follows: chemical shift [multiplicity (s = singlet, d = doublet, t = triplet, q = quartet, hept = heptet, m = multiplet and b = broad), coupling constant in Hz, integration, assignment]. ^{13}C NMR spectra were recorded with complete proton decoupling. Chemical shifts are reported in parts per million from tetramethylsilane with the solvent resonance as the internal standard (chloroform: δ = 77.00 ppm, acetic acid δ = 20.00 ppm or DMSO δ = 39.52 ppm). ^{19}F NMR spectra were recorded with complete proton decoupling. *J* values are reported in Hz. Assignments of $^1H/^{13}C$ spectra were made by the analysis of δ/J values, and COSY, HSQC, and HMBC experiments as appropriate. Melting points are uncorrected.

Extended optimisation table for arylation of pivaldehyde with a transient directing group

Full optimisation process for the direct aldehyde arylation starting from the pre-formed imine **2a** arylation conditions (Table 1, entry 1). Variables considered were Ag salt, solvent, Pd catalyst, additives as well as loadings of **1a** and catalyst and finally time and temperature. Most crucial was the change to AgTFA, use of HFIP as a co-solvent and addition of a donor lignand, with DMSO being optimal. Final optimisations to achieve maximum yield and reproducibility resulted in a 0.5 M concentration and 130 °C reaction temperature.

entry	catalyst	Ag salt	solvent	additive	yield **3a** (%)[a]	yield **3b** (%)[a]	yield **3c** (%)[a]	total yield (%)[a]
1	$Pd(OAc)_2$	AgOAc	AcOH	None	Trace	0	0	Trace
2	$Pd(OAc)_2$	Ag_3PO_4	AcOH	-	2	0	0	2
3	$Pd(OAc)_2$	Ag_2SO_4	AcOH	-	2	0	0	2
4	$Pd(OAc)_2$	AgTFA	AcOH	-	15	11	3	29
5	$Pd(OAc)_2$	AgF	AcOH	-	2	0	0	2
6	$Pd(OAc)_2$	AgTFA	HFIP	-	5	3	0	8
7	$Pd(OAc)_2$	AgTFA	DMSO	-	0	0	0	0
8	$Pd(OAc)_2$	AgTFA	Toluene	-	0	0	0	0
9	$Pd(OAc)_2$	AgTFA	DCE	-	2	0	0	2
10	$Pd(OAc)_2$	AgTFA	^{t}BuOH	-	0	0	0	0
11	$Pd(OAc)_2$	AgTFA	TFA	-	0	0	0	0
12	$Pd(OAc)_2$	AgTFA	PivOH	-	11	6	2	19
13	$Pd(OAc)_2$	AgTFA	$CH_3CH_2CO_2H$	-	9	4	Trace	13
14	$Pd(OAc)_2$	AgTFA	TCA	-	0	0	0	0
15	$Pd(OAc)_2$	AgTFA	Chloroacetic acid	-	8	3	Trace	11
16	$Pd(OAc)_2$	AgTFA	CHOOH	-	Trace	0	0	Trace
17	$Pd(OAc)_2$	AgTFA	HFIP:ACOH (3:1)	-	19	14	6	39
18	$Pd(OAc)_2$	AgTFA	HFIP:AcOH (1:1)	-	20	13	3	36
19	$Pd(OAc)_2$	AgTFA	HFIP:AcOH (1:3)	-	20	13	3	36
20	$Pd(OAc)_2$	AgTFA	DCE:AcOH (3:1)	-	11	0	0	11
21	$Pd(OAc)_2$	AgTFA	^{i}BuOH:AcOH (3:1)	-	Trace	0	0	Trace
22	$Pd(OAc)_2$	AgTFA	Toluene:AcOH (3:1)	-	17	5	3	25
23	$Pd(OAc)_2$	AgTFA	Toluene:AcOH (1:1)	-	15	6	Trace	21
24	$Pd(OAc)_2$	AgTFA	Toluene:AcOH (1:3)	-	18	5	Trace	23
20	$Pd(OAc)_2$	AgTFA	TFA:AcOH (1:9)	-	15	13	3	31
21	$Pd(OAc)_2$	AgTFA	TFA:AcOH (1:3)	-	20	13	1	34
22	$Pd(OAc)_2$	AgTFA	TFA:AcOH (1:1)	-	14	4	0	18
23	$PdCl_2$	AgTFA	HFIP:AcOH (1:1)	-	19	13	4	36
24	$Pd(OPiv)_2$	AgTFA	HFIP:AcOH (1:1)	-	23	17	7	47
25	$Pd(TFA)_2$	AgTFA	HFIP:AcOH (1:1)	-	20	12	5	37
26	$Pd(OPiv)_2$	AgTFA	HFIP:AcOH (1:1)	$Mn(OAc)_2$	20	11	4	35
27	$Pd(OPiv)_2$	AgTFA	HFIP:AcOH (1:1)	PivOH	20	13	4	37
28	$Pd(OPiv)_2$	AgTFA	HFIP:AcOH (1:1)	MesCOOH	23	13	4	40
29	$Pd(OPiv)_2$	AgTFA	HFIP:AcOH (1:1)	H_2O	18	12	5	35
30	$Pd(OPiv)_2$	AgTFA	HFIP:AcOH (1:1)	DMSO	24	14	5	43
31	$Pd(OPiv)_2$	AgTFA	HFIP:AcOH (1:1)	DMSO[b]	22	15	4	41
32	$Pd(OPiv)_2$	AgTFA	HFIP:AcOH (1:1)	Pyrrolidine	17	6	1	24
33	$Pd(OPiv)_2$	AgTFA	HFIP:AcOH (1:1)	Benzoquinone[c]	0	0	0	0
34[d]	$Pd(OPiv)_2$	AgTFA	HFIP:AcOH (1:1)	-	15	11	5	31
35[e]	$Pd(OPiv)_2$	AgTFA	HFIP:AcOH (1:1)	-	22	15	8	45
36[f]	$Pd(OPiv)_2$	AgTFA	HFIP:AcOH (1:1)	-	18	11	6	35
37[eg]	$Pd(OPiv)_2$	AgTFA	HFIP:AcOH (1:1)	-	22	18	8	46
38[eg]	$Pd(OPiv)_2$	AgTFA	HFIP:AcOH (1:1)	DMSO	28	18	9	55
39[eg]	$Pd(OPiv)_2$	AgTFA	HFIP:AcOH (1:1)	DMSO[c]	29	19	8	56
40[eg]	$Pd(OPiv)_2$	AgTFA	HFIP:AcOH (1:1)	KF[c]	14	8	5	27
41[eg]	$Pd(OPiv)_2$	AgTFA	HFIP:AcOH (1:1)	LiCl[c]	21	12	6	39
42[eg]	$Pd(OPiv)_2$	AgTFA	HFIP:AcOH (1:1)	DMA[c]	24	15	8	47
43[eg]	$Pd(OPiv)_2$	AgTFA	HFIP:AcOH (1:1)	DMPU[c]	22	15	9	46
44[eg]	$Pd(OPiv)_2$	AgTFA	HFIP:AcOH (1:1)	H_2O[c]	15	8	4	27
45[eg]	$Pd(OPiv)_2$	AgTFA	HFIP:AcOH (1:1)	DMF[c]	27	18	10	55
46[egh]	$Pd(OPiv)_2$	AgTFA	HFIP:AcOH (1:1)	DMSO[c]	29	22	10	61
47[ghi]	$Pd(OPiv)_2$	AgTFA	HFIP:AcOH (1:1)	DMSO[c]	28	20	9	57
48[ghj]	$Pd(OPiv)_2$	AgTFA	HFIP:AcOH (1:1)	DMSO[c]	26	16	5	47

Table S2: Optimisation of direct aldehyde arylation. Conditions: pivaldehyde (0.20 mmol), *N*-tosylethylenediamine **1a** (1 equiv), 4-iodoanisole (2.6 equiv), Pd catalyst (10 mol%), Ag source (2 equiv), additive (30 mol%), solvent (0.3 M), 120 C, 24 h unless stated otherwise. [a] Yields determined by ^{1}H NMR using 1,3,5-trimethoxybenzene as an internal standard. [b] 60 mol%. [c] 1.0 equiv. [d] 10 mol% $Pd(OPiv)_2$, 0.50 equiv **1a**. [e] 5 mol% $Pd(OPiv)_2$, 0.50 equiv **1a**. [f] 2.5 mol% $Pd(OPiv)_2$, 0.25 equiv **1a**. [g] 3 h. [h] 130 °C, 0.5 M. [i] 5 mol% $Pd(OPiv)_2$, 0.25 equiv **1a**. [j] 5 mol% $Pd(OPiv)_2$, 0.10 equiv **1a**.

Reaction profile experiments

Reaction profile for pivaldehyde arylation with different loadings of 1a and catalyst

A reaction profile was constructed using discrete experiments with different reaction times to compare the reaction progress at different loadings of **1a** and catalyst (figure S1; a) 10 mol% $Pd(OPiv)_2$, 1 equiv **1a** b) 5 mol% $Pd(OPiv)_2$, 0.5 equiv **1a**). The decreased loadings (Fig S1b) gave an almost identical reaction profile.

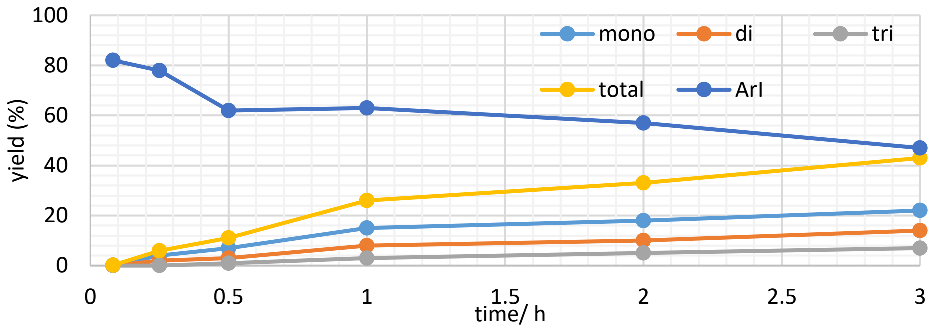

a) 10 mol% $Pd(OPiv)_2$, 1 equiv **1a**

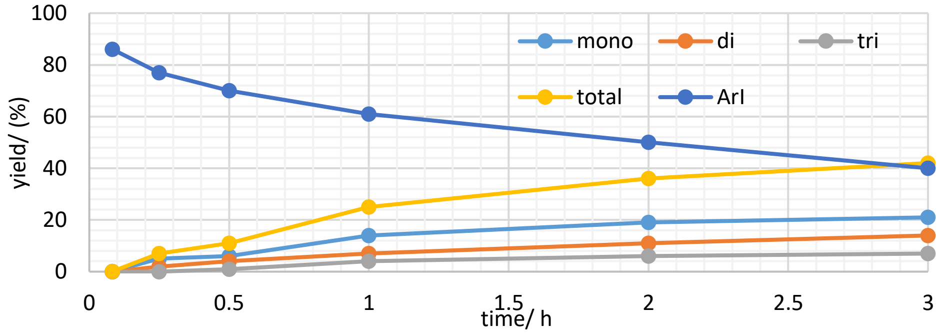

b) 5 mol% $Pd(OPiv)_2$, 0.5 equiv **1a**

Figure S1: Reaction profiles at different loadings of directing group and catalyst. Conditions: pivaldehyde (0.20 mmol), *N*-tosylethylenediamine **1a**, 4-iodoanisole (2.6 equiv), $Pd(OPiv)_2$, AgTFA (2 equiv), HFIP:AcOH (1:1, 0.3 M), 120 °C.

Reaction profile illustrating the effect of the DMSO additive

To observe the effect of the DMSO additive on the reaction, discrete reactions were carried out with different reaction times, with and without the additive (figure S2). The study demonstrated that the additon of DMSO gave both an enhancement in the yield of the desired arylated products, and also an increase in the quantitiy of unreacted aryl iodide (despite the increased yield). This suggests the DMSO may play a role in the prevention of unwanted side reactions involving the aryl iodide.

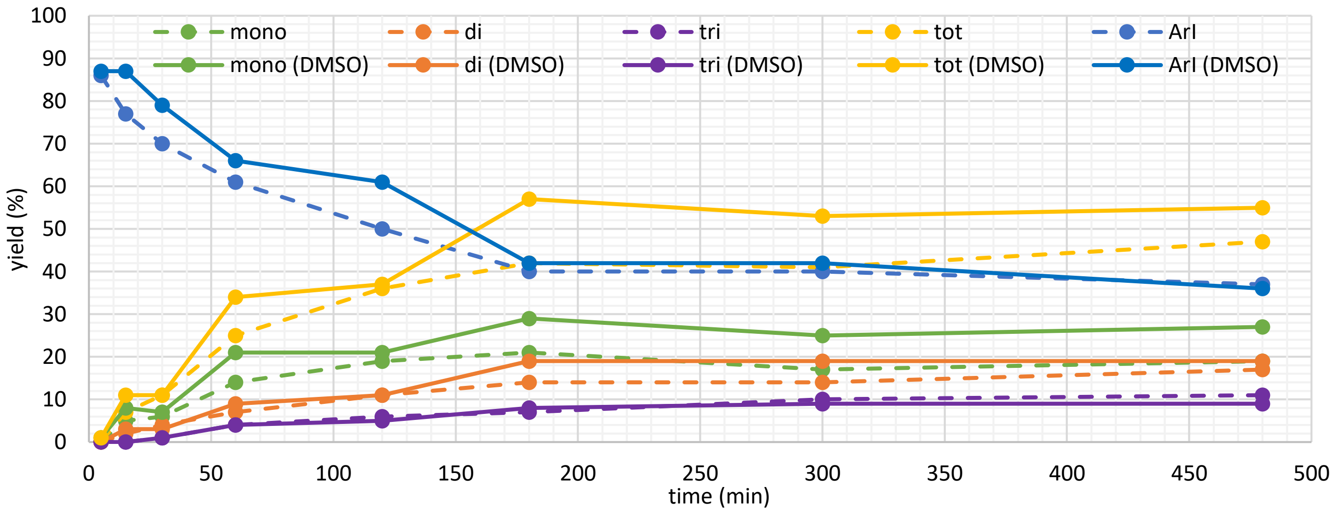

Figure S2: Reaction profile with and without DMSO additive. Conditions: pivaldehyde (0.20 mmol), *N*-tosylethylenediamine 1a (0.50 equiv), 4-iodoanisole (2.6 equiv), $Pd(OAc)_2$ (5 mol%), AgOAc (2 equiv), DMSO (1.0 equiv), HFIP:AcOH (1:1, 0.3 M), 120 °C.

Effect of TFA equivalents on reaction yield

When using $Pd(OAc)_2$ and AgOAc, therefore removing any TFA source from the reaction, there is a positive trend in the total yield when the amount of TFA added as an additive is increased (Figure S3). This vaule reaches a maximum at 2 equivalents, appearing to plateau at a 45% yield when 3 equivalents of TFA are used.

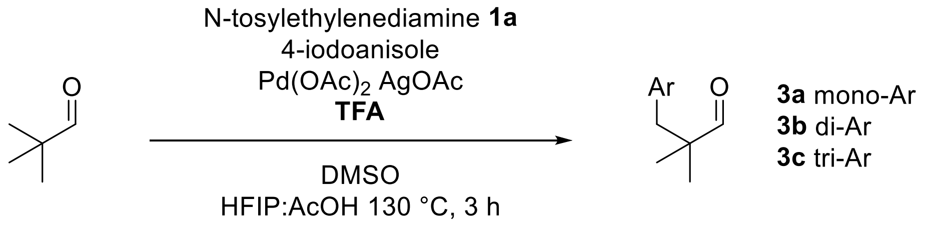

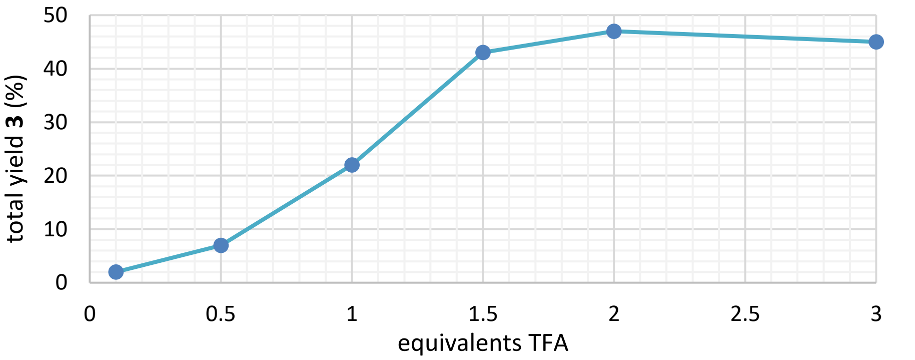

Figure S3: Effect of TFA on reaction yield. Conditions: pivaldehyde (0.20 mmol), *N*-tosylethylenediamine 1a (0.50 equiv), 4-iodoanisole (2.6 equiv), $Pd(OAc)_2$ (5 mol%), AgOAc (2 equiv), DMSO (1.0 equiv), HFIP:AcOH (1:1, 0.3 M), 120 °C, 3 h.

Imine formation and hydrolysis in AcOD-*d₄*

Imine formation in AcOD-d₄

From a 1:1 mixture of the free directing group **1a** and pivaldehyde, the amount of imine formation was calculated when the components were combined in AcOD-*d₄*.

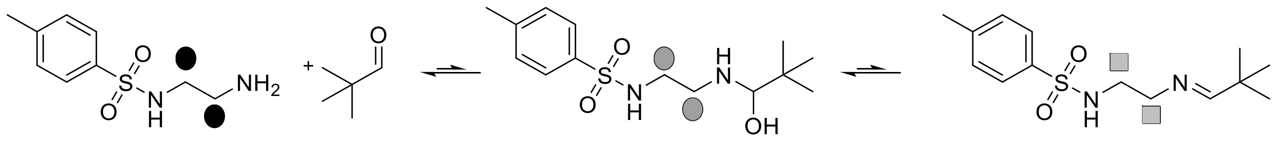

1a **2a**

Pivaldehyde (16.6 µL, 0.15 mmol), directing group **1a** (32.1 mg, 0.15 mmol), 1,3,5 trimethoxybenzene (9.0 mg, 0.054 mmol) and AcOD-*d₄* (0.5 mL) were combined in a Young's NMR tube and the ^{1}H NMR was immediately recorded (figure S4). 8% (0.012 mmol) of the components combined to form imine **2a**, with small amounts of a hydrolysis intermediate also present.

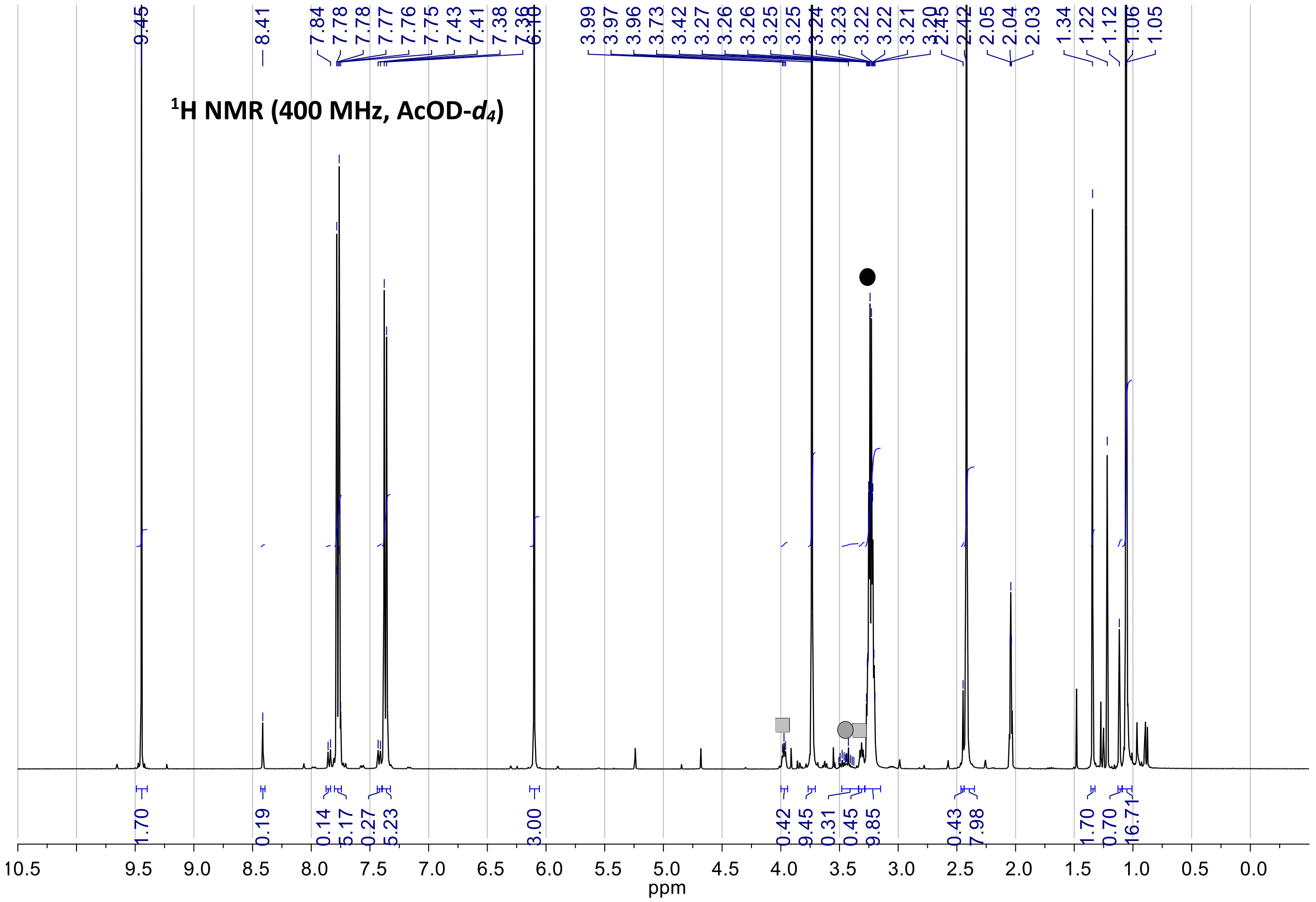

Figure S4: ^{1}H NMR of **1a** and pivaldehyde mixture in AcOD-*d₄*.

Imine formation in AcOD-d₄:HFIP (1:1)

The amount of imine formed in the improved solvent system of HFIP:AcOD-d_4 was also calculated.

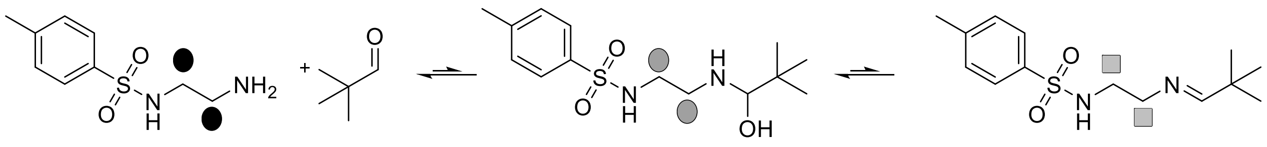

1a **2a**

Pivaldehyde (16.6 µL, 0.15 mmol), **1a** (32.1 mg, 0.15 mmol), 1,3,5 trimethoxybenzene (6.4 mg, 0.038 mmol) HFIP (0.25 mL) and AcOD-d_4 (0.25 mL) were combined in a Young's NMR tube and the ^{1}H NMR was immediately recorded (figure S5). Imine **2a** was observed in 22% (0.033 mmol) with small amounts of a hydrolysis intermediate also present.

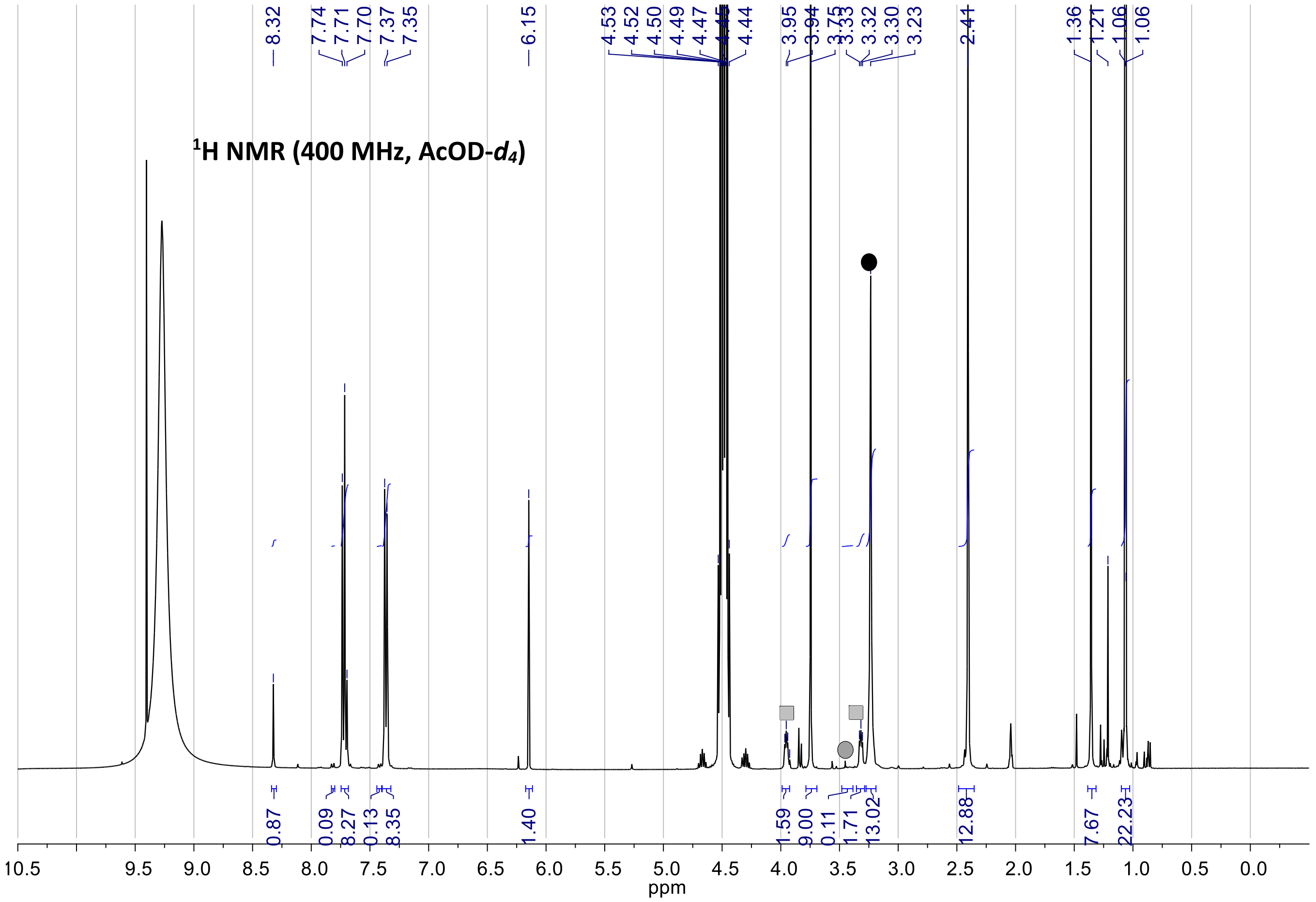

Figure S5: ^{1}H NMR of **1a** and pivaldehyde mixture in HFIP:AcOD-d_4 (1:1).

entry	solvent (0.3 M)	yield **1a** (%)^a	yield pivaldehyde (%)^a	yield intermediate (%)^a	yield imine **2a** (%)^a	total **1a** containing species (%)^a
1	AcOD	88	64	3	8	99
2	AcOD:HFIP (1:1)	83	62	1	22	106

Table S1: Yields of imine formation experiments. ^{a} Yields determined by ^{1}H NMR using 1,3,5-trimethoxybenzene as an internal standard.

Imine hydrolysis in AcOD-d₄

To detect any possible hydrolysis of the imine in the arylation solvent system, the imine was combined with AcOD-d_4 and observed by ^{1}H NMR.

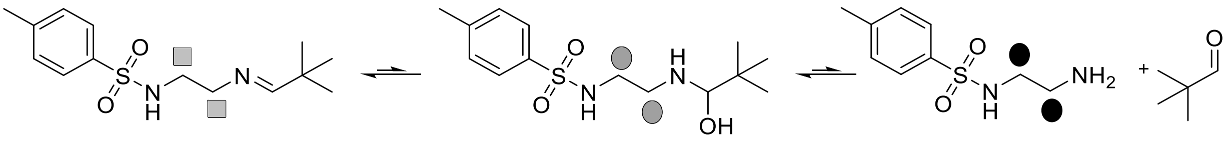

2a **1a**

Imine **2a** (41.9 mg 0.15 mmol), 1,3,5 trimethoxybenzene (10.1 mg, 0.060 mmol) and AcOD-d_4 (0.5 mL) were combined in a Young's NMR tube and the ^{1}H NMR was immediately recorded (figure S6). Of imine that was dissolved at this stage (53%), 56% (0.045 mmol) remained as imine **2a**, 15% (0.012 mmol) was the hydrolysis intermediate and 29% (0.023 mmol) was free directing group **1a**. Heating the sample enabled full dissolution of the imine however the ratio of imine hydrolysis was unchanged.

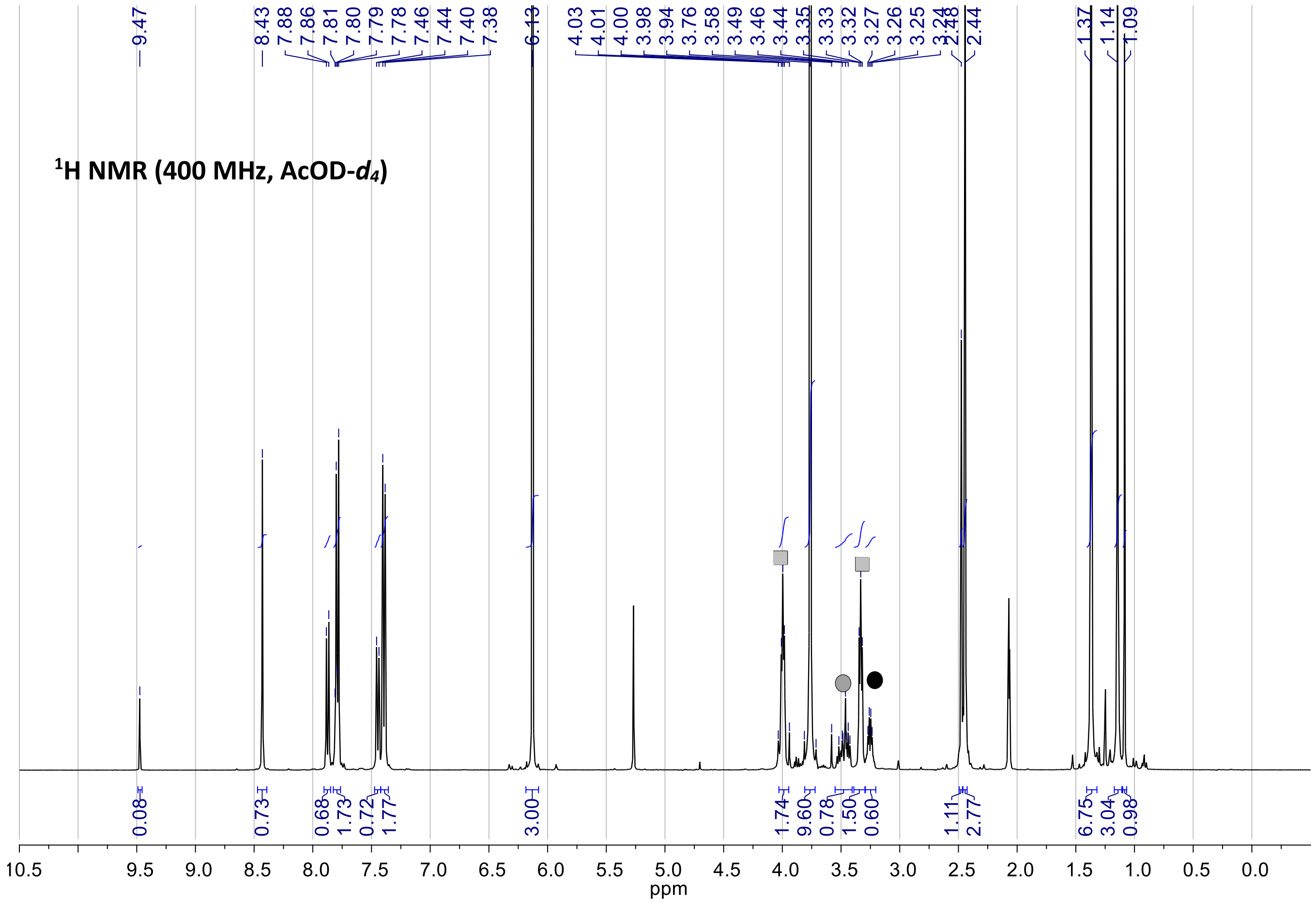

Figure S6: ^{1}H NMR of imine in AcOD-d_4.

Experimental procedures and characterisation data

Preparation of directing groups (1a-k)

General Procedure A

The relevant sulfonyl chloride (1.0 equiv) was added portionwise over a period of 1 h to a stirred solution of ethylenediamine (10 equiv) in CH_2Cl_2 (0.1 M) at 0 °C. The solution was stirred at 0 °C for 0.5 h then warmed to 25 °C and stirred for 18 h. The reaction mixture was diluted with CH_2Cl_2 and extracted with 1 M aqueous HCl. The combined aqueous extracts were basified to pH 10 with NaOH pellets and the product extracted with CH_2Cl_2. The combined organic extracts were dried ($MgSO_4$), filtered and the solvent removed under reduced pressure to afford the corresponding sulfonamides.

General Procedure B

tert-Butyl *N*-(2-aminoethyl)carbamate (1.0 equiv), the relevant carboxylic acid (1.0 equiv), EDC (2.5 equiv), HOBt (1.5 equiv) and DIPEA (3.5 equiv) were combined in CH_2Cl_2 (0.3 M) and the reaction was stirred at 25 °C for 20 h. The reaction mixture was diluted with saturated aqueous $NaHCO_3$ and the product extracted with CH_2Cl_2. The combined organic extracts were dried ($MgSO_4$), filtered and solvent removed under reduced pressure. Purification by flash chromatography (SiO_2, EtOAc:pentane:MeOH) afforded the corresponding Boc intermediate which was stirred in a (1:1) mixture of TFA and CH_2Cl_2 (0.3 M) at 25 °C until the reaction was complete by TLC. The solvents were removed under reduced pressure and the product was dissolved in diethyl ether and evaportated (× 3) to remove traces of TFA and afford the corresponding TFA salts.

***N*-(2-Aminoethyl)-4-methylbenzene-1-sulfonamide (1a)**

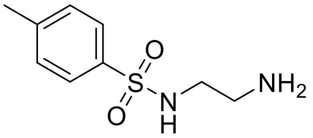

General procedure A was followed using tosyl chloride (2.06 g, 10.0 mmol) to afford sulfonamide **1a** as a white solid (6.35 g, 60%). R_f 0.28 (20% (1% NH_3 in MeOH)/CH_2Cl_2), mp = 123–124 °C (lit = 122–123 °C)[1]. IR (film)/cm^{-1} 3358, 3296, 2591 (br), 1592, 1311, 1298, 1148, 1095, 1054. ^{1}H NMR (400 MHz, $CDCl_3$) δ 7.77–7.75 (m, 2H, Ar-H), 7.33–7.30 (m, 2H, Ar-H), 2.96 (dd, *J* = 6.5, 4.8 Hz, 2H, NH*CH₂*), 2.79 (dd, *J* = 6.5, 4.8 Hz, 2H, NH_2*CH_2*), 2.43 (s, 3H, CH_3), 1.79 (bs, 2H, NH_2). ^{13}C NMR (101 MHz, $CDCl_3$) δ 143.4 (Ar-C_q), 136.9 (Ar-C_q), 129.7 (2 × Ar-C), 127.1 (2 × Ar-C), 45.4 ($NHCH_2$), 40.8 (NH_2CH_2), 21.5 (CH_3). Spectroscopic data for this compound (^{1}H NMR, IR)[2] and ^{13}C NMR[3] is consistent with that shown in the literature.

***N*-(2-Aminoethyl)benzenesulfonamide (1b)**

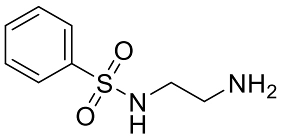

General procedure A was followed using benzenesulfonyl chloride (1.27 mL, 10.0 mmol) to afford sulfonamide **1b** as an off-white solid (634 mg, 32%). R_f 0.23 (20% (1% NH_3 in MeOH)/CH_2Cl_2). mp = 90–91 °C. IR (film)/cm^{-1} 3368 (w, N–H), 3312 (w, N–H), 2931, 2844, 2598, 1596. 1495, 1454, 1436, 1316, 1298, 1261, 1149 (s), 1094, 1028. ^{1}H NMR (400 MHz, $CDCl_3$) δ 7.88 (d, *J* = 7.3 Hz, 2H, Ph-H), 7.60–7.50 (m, 3H, Ph-H), 2.99–2.96 (m, 2H, CH_2), 2.81–2.78 (m, 2H, CH_2), 2.60 (bs, 2H, NH_2). ^{13}C NMR (101 MHz, $CDCl_3$) δ 139.9 (Ph-C_q), 132.6 (Ph-C), 129.1 (2 × Ph-C), 127.0 (2 × Ph-C), 45.3 (CH_2), 40.9 (CH_2). HRMS (ESI) m/z Calcd. for $C_8H_{13}N_2O_2S$ $[M+H]^+$: 201.0698; Found: 201.0697. Spectroscopic data (^{1}H NMR, ^{13}C NMR, MS)[4] and (IR)[5] for this compound is consistent with that shown in the literature.

***N*-(2-Aminoethyl)-4-methoxybenzene-1-sulfonamide (1c)**

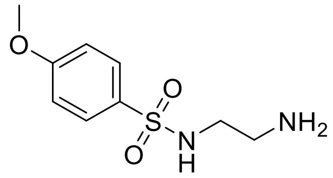

General procedure A was followed using 4-methoxybenzenesulfonyl chloride (2.06 g, 10.0 mmol) to afford sulfonamide **1c** as a white solid (1.63 g, 71%). R_f 0.17 (20% (1% NH_3 in MeOH)/CH_2Cl_2). mp = 98–100 °C (lit = 90–91 °C)[5]. IR (film)/cm^{-1} 3368 (w, N–H), 3312 (w, N–H), 2933, 2844, 2585, 1596, 1579, 1495, 1454, 1435, 1298, 1261, 1148 (s), 1094, 1028. ^{1}H NMR (400 MHz, $CDCl_3$) δ 7.84–7.79 (m, 2H, Ar-H), 7.01–6.96 (m, 2H, Ar-H), 3.88, (s, 3H, OCH_3), 2.95 (dd, *J* = 6.5, 4.8 Hz, 2H, CH_2), 2.79 (dd, *J* = 6.5, 4.8 Hz, 2H, CH_2), 1.53 (bs, 2H, NH_2). ^{13}C NMR (101 MHz, $CDCl_3$) δ 162.8 (Ar-C_q), 131.5 (Ar-C_q), 129.2 (2 × Ar-C), 114.2 (2 × Ar-C), 55.6 (OCH_3), 45.4 (CH_2), 40.8 (CH_2). HRMS (ESI) m/z Calcd. for $C_9H_{15}N_2O_3S$ [M+H]$^+$: 231.0803; Found: 231.0811. Spectroscopic data (^{1}H NMR, ^{13}C NMR) for this compound is consistent with that shown in the literature.[5]

***N*-(2-Aminoethyl)-4-(trifluoromethyl)benzene-1-sulfonamide (1d)**

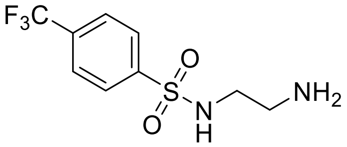

General procedure A was followed using 4-(trifluoromethyl)benzene-1-sulfonyl chloride (2.45 g, 10.0 mmol) to afford sulfonamide **1d** as a white solid (2.11 g, 79%). R_f 0.21 (20% (1% NH_3 in MeOH)/CH_2Cl_2). mp = 112–116 °C (lit = 103–105 °C)[4]. IR (film)/cm^{-1} 3371, 2970, 2637, 1738, 1606, 1402, 1365, 1325, 1217, 1153, 1127, 1100, 1062, 1043, 1016. ^{1}H NMR (400 MHz, $CDCl_3$) δ 8.02 (d, *J* = 8.2 Hz, 2H, Ar-H), 7.80 (d, *J* = 8.2 Hz, 2H, Ar-H), 3.01 (t, *J* = 5.6 Hz, 2H, CH_2), 2.83 (t, *J* = 5.6 Hz, 2H, CH_2), 1.90 (bs, 2H, NH_2). ^{13}C NMR (101 MHz, $CDCl_3$) δ 143.6 (Ar-C_q), 134.5 (q, J_{CF} = 33.2 Hz, CF_3Ar-C_q), 127.5 (2 × Ar-C), 126.4 (q, J_{CF} = 3.7 Hz, 2 × Ar-C), 123.4 (q, J_{CF} = 272.8 Hz, CF_3), 45.2 (CH_2), 40.7 (CH_2). ^{19}F NMR (377 MHz, $CDCl_3$) δ –63.01 HRMS (ESI) m/z Calcd. for $C_9H_{12}N_2O_2SF_3$ [M+H]$^+$: 269.0572; Found: 269.0584. Spectroscopic data (^{1}H NMR, ^{13}C NMR) for this compound is consistent with that shown in the literature.[4]

***N*-(2-Aminoethyl)-2,6-dichlorobenzene-1-sulfonamide (1e)**

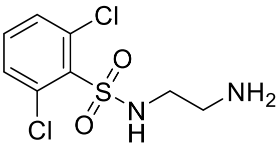

General procedure A was followed using 2,6-dichlorobenzenesulfonyl chloride (980 mg, 4.00 mmol) to afford sulfonamide **1e** as a white solid (921 mg, 86%). R_f 0.34 (20% (1% NH_3 in MeOH)/CH_2Cl_2). mp = 121–123 °C IR (film)/cm^{-1} 3371, 3312, 2936, 1736, 1594, 1560, 1424, 1330, 1292, 1160, 1096, 1047. ^{1}H NMR (400 MHz, $CDCl_3$) δ 7.48 (d, *J* = 8.0 Hz, 2H, Ar-H), 7.35 (t, *J* = 8.0 Hz, 1H, Ar-H), 3.07–3.04 (m, 2H, CH_2), 2.85–2.83 (m, 2H, CH_2), 2.39 (bs, 2H, NH_2). ^{13}C NMR (101 MHz, $CDCl_3$) δ 135.3 (Ar-C_q), 134.9 (2 × Ar-C_q), 132.4 (Ar-C), 131.4 (2 × Ar-C), 45.5 (CH_2), 40.8 (CH_2). HRMS (ESI) m/z Calcd. for $C_8H_{11}N_2O_2SCl_2$ [M+H]$^+$: 268.9918; Found: 268.9928.

***N*-(2-Aminoethyl)-2,4,6-tris(propan-2-yl)benzene-1-sulfonamide (1f)**

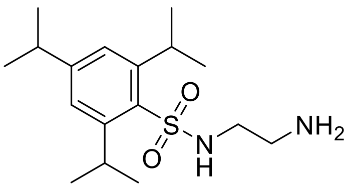

General procedure A was followed using 2,4,6-triisopropylbenzenesulfonyl chloride (7.58 g, 25.0 mmol) to afford sulfonamide **1f** as a white solid (257 mg, 3%). R_f 0.35 (20% (1% NH_3 in MeOH)/CH_2Cl_2). mp = 123–124 °C. IR (film)/cm^{-1} 3369, 2955, 1738, 1594, 1563, 1458, 1422, 1363, 1314, 1295, 1247, 1229, 1153, 1093, 1060. ^{1}H NMR (400 MHz, $CDCl_3$) δ 7.17 (s, 2H, Ar-H), 5.02 (bs, 1H, NH), 4.18 (hept, *J* = 6.6 Hz, 2H, C*H*(CH_3)$_2$), 3.01–2.98 (m, 2H, CH_2), 2.96–9.87 (m, 1H, C*H*(CH_3)$_2$), 2.86–2.84 (m, 2H, CH_2), 1.27 (t, *J* = 6.6 Hz, 18H, CH(C*H*$_3$)$_2$). ^{13}C NMR (101 MHz, $CDCl_3$) δ 152.6 (Ar-C_q), 150.2 (2 × Ar-C_q), 132.2 (Ar-C_q), 123.8 (2 × Ar-C), 44.9 (CH_2), 40.8 (CH_2), 34.1 (*C*H(CH_3)$_2$), 29.6 (2 × *C*H(CH_3)$_2$), 24.9 (2 × CH(*C*H$_3$)$_2$), 23.6 (CH(*C*H$_3$)$_2$). HRMS (ESI) m/z Calcd. for $C_{17}H_{31}N_2O_2S$ [M+H]$^+$: 327.2106; Found: 327.2100.

***N*-(2-Aminoethyl)butane-1-sulfonamide (1g)**

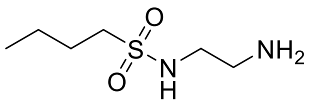

General procedure A was followed using 1-butanesulfonyl chloride (1.94 mL, 15.0 mmol) to afford sulfonamide **1g** as a pale yellow wax (628 mg, 23%). R_f 0.19 (20% (1% NH_3 in MeOH)/CH_2Cl_2). IR (film)/cm^{-1} 3295, 2961, 2874, 1596, 1466, 1314, 1276, 1134, 921. ^{1}H NMR (400 MHz, $CDCl_3$) δ 3.15–3.12 (m, 2H, CH_2), 3.05–3.01 (m, 2H, CH_2), 2.90–2.87 (m, 2H, CH_2), 2.73 (bs, 2H, NH_2), 1.83–1.75 (m, 2H, CH_2), 1.50–1.41 (m, 2H, CH_2), 0.95 (t, *J* = 7.4 Hz, 2H, CH_3). ^{13}C NMR (101 MHz, $CDCl_3$) δ 52.3 (CH_2), 45.4 (CH_2), 41.6 (CH_2), 25.6 (CH_2), 21.5 (CH_2), 13.6 (CH_3). HRMS (ESI) m/z Calcd. for $C_6H_{17}N_2O_2S$ [M+H]$^+$: 181.1011; Found: 181.1018.

***N*-(2-Azaniumylethyl)benzamide trifluoroacetate (1h)**

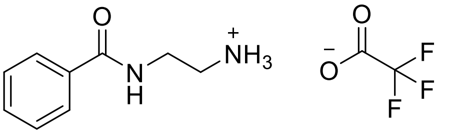

General procedure B was followed using benzoic acid (1.46 g, 12.0 mmol) to afford the intermediate *tert*-butyl *N*-[2-(phenylformamido)ethyl]carbamate **S1** as a white solid (673 mg, 85%). R_f 0.26 (40% EtOAc/pentane). mp = 130–131 °C. IR (film)/cm^{-1} 3354, 3324, 2291, 2970, 2934, 1685, 1637, 1525 (s), 1447, 1387, 1327, 1277, 1234, 1166, 1151. ^{1}H NMR (400 MHz, $CDCl_3$) δ 7.82 (d, *J* = 7.4 Hz, 2H, Ph-H), 7.51–7.47 (m, 1H, Ph-H), 7.44–7.40 (m, 2H, Ph-H), 7.21 (bs, 1H, NH), 5.05 (bs, 1H, NH) 3.58–3.54 (m, 2H, BocNHC*H$_2$*), 3.41 (dd, *J* = 10.8, 5.7 Hz, 2H, CONHC*H$_2$*), 1.43 (s, 9H, C(CH_3)$_3$). ^{13}C NMR (101 MHz, $CDCl_3$) δ 167.8 (C=O), 157.5 (C=O), 134.1 (Ph-C_q), 131.4 (Ph-C), 128.5 (2 × Ph-C), 127.0 (2 × Ph-C), 80.0 (*C*(CH_3)$_3$), 42.0 (BocNH*CH$_2$*), 39.9 (CONH*CH$_2$*), 28.3 (C(*C*H$_3$)$_3$). Spectroscopic data (^{1}H NMR, ^{13}C NMR, IR) for this compound is consistent with that shown in the literature.[6] Boc deprotection of *tert*-butyl *N*-[2-(phenylformamido)ethyl]carbamate **S1** (2.59 g, 9.81 mmol) gave title salt **1h** as an off-white solid (2.77 g, 100%). mp = 130–131 °C. IR (film)/cm^{-1} 3300, 3017, 1779 (w, C=O), 1669 (C=O), 1624, 1595, 1549, 1517, 1425, 1316, 1172 (s), 1158 (s), 1125 (s), 1036. ^{1}H NMR (400 MHz, DMSO) δ 8.64 (t, *J* = 5.4 Hz, 1H, CONH), 8.00–7.73 (bm, 5H, NH_3, 2 × Ph-H), 7.57–7.46 (m, 3H, Ph-H), 3.51 (q, *J* = 6.1 Hz, 2H, $NHCH_2$), 3.03–2.96 (m, 2H, NH_3*CH$_2$*). ^{13}C NMR (101 MHz, DMSO) δ 166.9 (C=O), 160.8 (C=O), 134.0 (Ph-C_q), 131.4 (Ph-C), 128.3 (2 × Ph-C), 127.3 (2 × Ph-C), 38.7 (CH_2), 37.1 (CH_2). HRMS (ESI) m/z Calcd. for $C_9H_{13}N_2O$ [M+H]$^+$: 165.1028; Found: 165.1035.

tert-Butyl N-[2-(2,2,2-trichloroacetamido)ethyl]carbamate (S2)

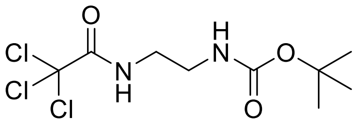

Trichloroacetyl chloride was added dropwise to a stirred solution of *tert*-butyl *N*-(2-aminoethyl)carbamate (316 µL, 2.00 mmol) and pyridine (163 µL, 2.20 mmol) in CH_2Cl_2 (3.3 mL) at 0 °C. The reaction was allowed to warm to 25 °C and was stirred for 1 h. The reaction mixture was concentrated under reduced pressure and the resulting residue purified by column chromatography (silica, 30% EtOAc/Pentane) to afford amide **S2** as a white solid (526 mg, 86%). Rf 0.40 (30% EtOAc/pentane). mp = 145–146 °C. IR (film)/cm^{-1} 3319, 2966, 2876, 1682 (C=O), 1579, 1516, 1434, 1367, 1310, 1275, 1233, 1159. ^1H NMR (400 MHz, $CDCl_3$) δ 7.95 (bs, 1H, NH), 4.91 (bs, 1H, NH), 3.49–3.45 (m, 2H, CH_2, BocNH*CH₂*), 3.43–3.40 (m, 2H, CH_2, CONHCH_2), 1.45 (s, 9H, (C(CH_3)$_3$). ^{13}C NMR (101 MHz, $CDCl_3$) δ 162.7 (C=O), 157.6 (C=O), 92.4 (CCl_3), 80.5 (*C*(CH_3)$_3$), 43.9 (BocNHCH_2), 39.1 (CONHCH_2), 28.3 (C(CH_3)$_3$). HRMS (ESI) m/z Calcd. for $C_9H_{14}N_2O_3Cl_3$ $[M+H]^+$: 303.0070; Found: 303.0063.

N-(2-Azaniumylethyl)-2,2,2-trichloroacetamide trifluoroacetate (1i)

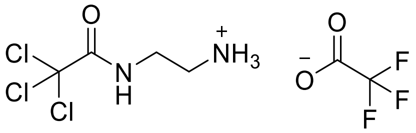

tert-Butyl *N*-[2-(2,2,2-trichloroacetamido)ethyl]carbamate **S2** (475 mg, 1.56 mmol) was stirred in a mixture of TFA (2.5 mL) and CH_2Cl_2 (2.5 mL) at 25 °C for 18 h. The solvents were removed under reduced pressure and the resulting gum dissolved in diethyl ether and evaporated (x3) to afford the title salt **1i** as an off-white solid (425 mg, 85%). mp = 124–126 °C. IR (film)/cm^{-1} 3047 (br), 1698 (C=O), 1675 (C=O), 1536, 1444, 1230, 1202, 1179 (s), 1140 (s), 1092, 1029. ^1H NMR (400 MHz, DMSO) δ 9.14 (t, *J* = 5.2 Hz, 1H, NH), 7.91 (bs, 3H, NH_3), 3.44 (dd, *J* = 12.5, 6.7 Hz, 2H, CH_2), 2.96 (t, *J* = 6.7 Hz, 2H, CH_2). ^{13}C NMR (101 MHz, DMSO) δ 162.1 (C=O), 92.4 (CCl_3), 38.6 (CH_2), 37.6 (CH_2). HRMS (ESI) m/z Calcd. for $C_4H_8N_2OCl_3$ $[M+H]^+$: 204.9702; Found: 204.9697.

N-(2-Azaniumylethyl)-2,6-difluorobenzamide trifluoroacetate (1j)

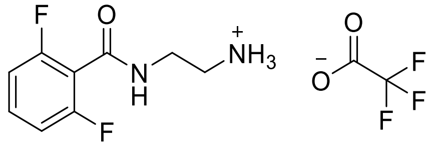

General procedure B was followed using 2,6-difluorobenzoic acid (1.90 g, 12.0 mmol) followed by recrystallisation in EtOAc to afford the intermediate *tert*-butyl *N*-{2-[(2,6-difluorophenyl)formamido]ethyl}carbamate **S3** as a white solid (2.22 g, 62%). R_f 0.26 (40% EtOAc/pentane). mp = 141 °C. IR (film)/cm^{-1} 3306, 2977, 2927, 1696 (C=O), 1652 (C=O), 1626, 1534, 1467, 1367, 1324, 1286, 1235, 1156, 1022, 1004. ^1H NMR (400 MHz, $CDCl_3$) δ 7.36 (tt, *J* = 8.4, 6.4 Hz, 1H, Ar-H), 6.97–6.91 (m, 2H, Ar-H), 6.70 (bs, 1H, NH), 4.94 (bs, 1H, NH), 3.59 (dd, *J* = 11.2, 5.5 Hz, 2H, BocNHCH_2), 3.40 (dd, *J* = 11.2, 5.5 Hz, 2H, CONHCH_2), 1.43 (s, 9H, C(CH_3)$_3$). ^{13}C NMR (101 MHz, $CDCl_3$) δ 161.2 (C=O), 160.0 (dd, J_{CF} = 252.6, 7.1 Hz, 2 × Ar-C_qF), 131.6 (t, J_{CF} = 10.2 Hz, FAr-C), 114.3 (Ar-C_q), 112.0 (dd, J_{CF} = 20.0, 5.1 Hz, 2 × FAr-C) 79.9 (*C*(CH_3)$_3$), 41.4 (BocNHCH_2), 39.9 (CONHCH_2), 28.3 (C(CH_3)$_3$) ^{19}F NMR (377 MHz, $CDCl_3$) δ –112.2. HRMS (ESI) m/z Calcd. for $C_{14}H_{19}N_2O_3F_2$ $[M+H]^+$: 301.1364; Found: 301.1368. Boc deprotection of *tert*-butyl *N*-{2-[(2,6-difluorophenyl)formamido]ethyl}carbamate **S3** (2.22 g, 7.40 mmol) gave the title salt **1j** as an off-white solid (2.11 g, 94%). mp = 127–128 °C. IR (film)/cm^{-1} 3259, 3083, 2672, 1679 (C=O), 1655, 1644, 1624, 1574, 1560, 1466, 1320, 1234, 1199 (s), 1178 (s), 1150 (s), 1132 (s), 1003. ^1H NMR (400 MHz, DMSO) δ 8.90 (t, *J* = 5.4 Hz, 1H, NH), 7.94 (bs, 3H, NH_3), 7.54 (tt, *J* = 8.4, 6.6 Hz, 1H, Ar-H), 7.25–7.16 (m, 2H, Ar-H), 3.48 (dd, *J* = 12.9, 7.0 Hz, 2H, NHCH_2), 2.95 (bs, 2H, NH_3CH_2). ^{13}C NMR (101 MHz, DMSO) δ 160.1 (C=O), 158.8 (dd, J_{CF} = 248.9, 7.0 Hz, 2 × Ar-C_qF), 132.0 (t, J_{CF} = 9.2 Hz, FAr-C), 114.8 (Ar-C_q), 112.0 (dd, J_{CF} = 19.7, 5.2 Hz, 2 × FAr-C), 38.0 (CH_2), 36.9 (CH_2). ^{19}F NMR (377 MHz, DMSO) δ –73.7, –113.7. HRMS (ESI) m/z Calcd. for $C_9H_{11}N_2OF_2$ $[M+H]^+$: 201.0839; Found: 201.0838.

***N*-(2-Azaniumylethyl)-2,3,4,5,6-pentafluorobenzamide trifluoroacetate (1k)**

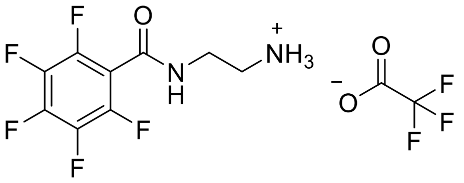

General procedure B was followed using pentafluorobenzoic acid (1.90 g, 8.90 mmol) followed by recrystallisation in EtOAc to afford the intermediate *tert*-butyl *N*-{2-[(2,3,4,5,6-pentafluorophenyl)formamido]ethyl}carbamate **S4** as a white solid (1.12 g, 35%). R_f 0.33 (40% EtOAc/pentane). mp = 141 °C. IR (film)/cm^{-1} 3351, 3304, 2950, 1688 (C=O), 1663 (C=O), 1520, 1483, 1448, 1335, 1283, 1237, 1173, 1120. ^{1}H NMR (400 MHz, $CDCl_3$) δ 7.18 (bs, 1H, NH), 4.95 (bs, 1H, NH), 3.60–3.56 (m, 2H, BocNH*CH₂*), 3.40 (dd, *J* = 11.2, 5.9 Hz, 2H, CONH*CH₂*), 1.43 (s, 9H, $C(CH_3)_3$). ^{13}C NMR (101 MHz, $CDCl_3$) δ 157.8 (C=O), 157.5 (C=O), 80.4 (*C*$(CH_3)_3$), 42.5 (BocNHCH_2), 39.6 (CONHCH_2), 28.2 (C(CH$_3$)$_3$). Ar-C signals not observed due to complex coupling. ^{19}F NMR (377 MHz, $CDCl_3$) δ –140.42 to –140.56 (m, 2F), –151.08 (t, *J* = 20.5 Hz), –160.31 (td, *J* = 21.2, 6.2 Hz, 2F). HRMS (ESI) m/z Calcd. for $C_{14}H_{16}N_2O_3F_5$ [M+H]$^+$: 355.1081; Found: 355.1082. Boc deprotection of *tert*-butyl *N*-{2-[(2,3,4,5,6-pentafluorophenyl)formamido]ethyl}carbamate **S4** (1.12 g, 3.16 mmol) gave the title salt **1k** as an off-white solid (1.09 g, 87%). mp = 162–165 °C. IR (film)/cm^{-1} 3320, 2966, 2906, 2861, 2819, 1670, 1644, 1579, 1546, 1457, 1357, 1303, 1204, 1179, 1115. ^{1}H NMR (400 MHz, DMSO) δ 9.20 (t, *J* = 5.3 Hz, 1H, NH), 7.96 (bs, 3H, NH_3), 3.51 (dd, *J* = 12.8, 6.7 Hz, 2H, NHCH_2), 2.97 (t, *J* = 6.9 Hz, 2H, NH_3CH_2). ^{13}C NMR (101 MHz, DMSO) δ 157.2 (C=O), 37.9 (CH_2), 37.1 (CH_2). Ar-C signals not observed due to complex coupling. ^{19}F NMR (377 MHz, DMSO) δ –73.9, –141.5 (dd, *J* = 23.1, 5.9 Hz, 2F), –152.65 (t, *J* = 22.0 Hz), –161.26 to –161.72 (m, 2H). HRMS (ESI) m/z Calcd. for $C_9H_8N_2OF_5$ [M+H]$^+$: 255.0557; Found: 255.0550.

***N*-(2-Aminopropyl)-4-methylbenzenesulfonamide (1a-Me)**

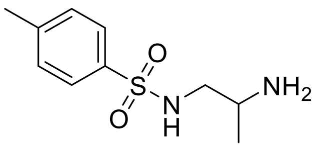

Tosyl chloride (2.06 g, 10.0 mmol) was added portionwise over a period of 1 h to a stirred solution of propane-1,2-diamine (8.5 mL, 100 mmol) in CH_2Cl_2 (0.1 M) at 0 °C. The solution was stirred at 0 °C for 0.5 h then warmed to 25 °C and stirred for 18 h. The reaction mixture was diluted with CH_2Cl_2 and extracted with 1 M aqueous HCl. The combined aqueous extracts were basified to pH 10 with NaOH pellets and the product extracted with CH_2Cl_2. The combined organic extracts were dried ($MgSO_4$), filtered and the solvent removed under reduced pressure to afford the sulfonamide **1a-Me** as a white solid (2.02 g, 92%). R_f 0.34 (20% (1% NH_3 in MeOH)/CH_2Cl_2), mp = 93–96 °C. IR (film)/cm^{-1} 2701, 1739, 1597, 1323, 1278, 1149, 1070, 1089. ^{1}H NMR (400 MHz, $CDCl_3$) δ 7.75 (d, J = 8.2 Hz, 2H, Ar-H), 7.32 (d, J = 8.1 Hz, 2H, Ar-H), 3.00–2.93 (m, 2H, CH_2), 2.72–2.51 (m, 1H, C*H*(CH_3)), 2.44 (s, 3H, Ar-CH_3), 1.43 (bs, 2H, NH_2), 1.04 (t, J = 5.5 Hz, 3H, CH_3). ^{13}C NMR (101 MHz, $CDCl_3$) δ 143.3 (Ar-C_q), 136.9 (Ar-C_q), 129.7 (Ar-C), 127.0 (Ar-C), 50.4 (*C*H(CH_3)), 46.2 (CH_2), 21.8 (CH_3), 21.5 (Ar-CH_3). HRMS (ESI) m/z Calcd. for $C_{10}H_{17}NO_2S$ [M+H]$^+$: 229.1011; Found: 229.1022.

Preparation of imines (2a-n)

General Procedure C

Pivaldehyde (1 equiv) was added to a stirred suspension of amine (1 equiv) and magnesium sulfate (2 equiv) in CH_2Cl_2 (0.3 M) and the reaction was stirred at 25 °C overnight. The reaction mixture was filtered through a bed of Celite, concentrated under reduced pressure and dissolved in either diethyl ether or toluene and evaporated to afford the corresponding imines.

General Procedure D

Pivaldehyde (1 equiv) was added to a stirred suspension of amine salt (1 equiv), triethylamine (1.5 equiv) and magnesium sulfate (2 equiv) in CH_2Cl_2 (0.3 M) and the reaction was stirred at 25 °C overnight. The reaction mixture was filtered through a bed of Celite and diluted with CH_2Cl_2. An equal volume of a 1:1 mixture of saturated aqueous $NaHCO_3$ and distilled water was added and the product extracted with CH_2Cl_2, dried ($MgSO_4$), filtered and concentrated under reduced pressure to afford the corresponding imines.

***N*-{2-[(*E*)-(2,2-Dimethylpropylidene)amino]ethyl}-4-methylbenzene-1-sulfonamide (2a)**

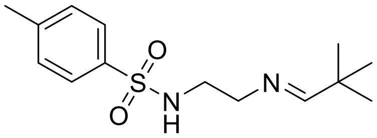

General procedure C was followed using *N*-(2-aminoethyl)-4-methylbenzene-1-sulfonamide **1a** (535 mg, 2.50 mmol) to afford imine **2a** as a white solid (712 mg, 100%) as a mixture of major and minor stereoisomers (3.5:1). NMR data quoted for the major isomer only. mp = 66–67 °C. IR (film)/cm^{-1} 3286, 2959, 2867, 1665, 1598, 1454, 1400, 1326, 1305, 1156 (s), 1091. ^{1}H NMR (400 MHz, $CDCl_3$) δ 7.77–7.74 (m, 2H, Ar-H), 7.49 (s, 1H, N=CH), 7.34–7.30 (m, 2H, Ar-H), 4.75 (t, *J* = 5.6 Hz, 1H, NH), 3.40 (t, *J* = 5.6 Hz, 2H, CH=N*CH₂*), 3.20–3.13 (m, 2H, NH*CH₂*), 2.44 (s, 3H, Ar-CH_3), 1.03 (s, 9H, $C(CH_3)_3$). ^{13}C NMR (101 MHz, $CDCl_3$) δ 174.7 (CH=N), 143.4 (Ar-C_q), 137.1 (Ar-C_q), 129.7 (2 × Ar-C), 127.1 (2 × Ar-C), 59.2 (CH=N*CH₂*), 43.8 ($NHCH_2$), 36.3 (*C*$(CH_3)_3$) 26.8 (C(*C*$H_3)_3$), 21.5 (Ar-CH_3). HRMS (ESI) m/z Calcd. for $C_{14}H_{23}N_2O_2S$ [M+H]$^+$: 283.1480; Found: 283.1474.

***N*-{2-[(*E*)-(2,2-Dimethylpropylidene)amino]ethyl}benzenesulfonamide (2b)**

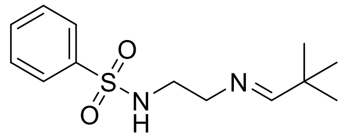

General procedure C was followed using *N*-(2-aminoethyl)-benzenesulfonamide **1b** (300 mg, 1.50 mmol) to afford imine **2b** as a yellow oil (312 mg, 78%) as a mixture of major and minor stereoisomers (7.3:1). NMR data quoted for the major isomer only. IR (film)/cm^{-1}3301, 2958, 2867, 1738, 1665, 1477, 1446, 1364, 1324, 1217, 1156, 1091. ^{1}H NMR (400 MHz, $CDCl_3$) δ 7.89–7.86 (m, 2H, Ph-H), 7.64–7.50 (m, 3H, Ph-H), 7.48 (t, *J* = 1.2 Hz, 1H, N=CH), 4.88 (bs, 1H, NH), 3.40 (td, *J* = 5.7, 1.1 Hz, 2H, CH=N*CH₂*), 3.20–3.17 (m, 2H, NH*CH₂*), 1.02 (s, 9H, $C(CH_3)_3$). ^{13}C NMR (101 MHz, $CDCl_3$) δ 174.8 (CH=N), 140.0 (Ar-C_q), 132.6 (Ar-C), 129.1 (2 × Ar-C), 127.0 (2 × Ar-C), 59.1 (CH=N*CH₂*), 43.8 ($NHCH_2$), 36.3 (*C*$(CH_3)_3$), 26.8 (C(*C*$H_3)_3$). HRMS (ESI) m/z Calcd. for $C_{13}H_{20}N_2O_2S$ [M+H]$^+$: 269.1318; Found: 269.1309.

_N_-{2-[(_E_)-(2,2-Dimethylpropylidene)amino]ethyl}-4-methoxybenzene-1-sulfonamide (2c)

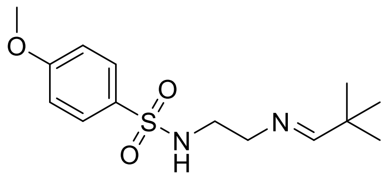

General procedure C was followed using *N*-(2-aminoethyl)-4-methoxybenzene-1-sulfonamide **1c** (345 mg, 1.50 mmol) to afford imine **2c** as an off-white solid (349 mg, 78%) as a mixture of major and minor stereoisomers (6.1:1). NMR data quoted for the major isomer only. mp = 76–79 °C. IR (film)/cm^{-1} 3370, 3312, 2956, 2868, 1737, 1665, 1596, 1579, 1498, 1461, 1413, 1327, 1302, 1257, 1151 (s), 1092, 1024. ^{1}H NMR (400 MHz, $CDCl_3$) δ 7.82–7.79 (m, 2H, Ar-H), 7.49 (t, *J* = 1.2 Hz, 1H, N=CH), 7.00–6.96 (m, 2H, Ar-H), 4.79 (bs, 1H, NH), 3.87 (s, 3H, OCH_3), 3.41–3.38 (m, 2H, CH=N*CH₂*), 3.18–3.11 (m, 2H, NH*CH₂*), 1.02 (s, 9H, $C(CH_3)_3$). ^{13}C NMR (101 MHz, $CDCl_3$) δ 174.8 (CH=N), 162.8 (Ar-C_q), 129.7 (Ar-C_q), 129.2 (2 × Ar-C), 114.2 (2 × Ar-C), 59.2 (CH=N*CH₂*), 55.6 (OCH_3), 43.7 ($NHCH_2$), 36.3 (*C*(CH_3)$_3$), 26.8 (C(*C*H$_3$)$_3$). HRMS (ESI) m/z Calcd. for $C_{14}H_{22}N_2O_2S$ [M+H]$^+$: 299.1424; Found: 299.1422

_N_-{2-[(_E_)-(2,2-Dimethylpropylidene)amino]ethyl}-4-(trifluoromethyl)benzene-1-sulfonamide (2d)

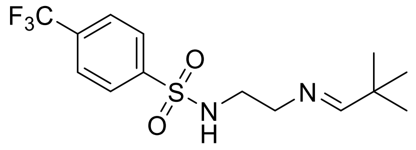

General procedure C was followed using *N*-(2-aminoethyl)-4-(trifluoromethyl)benzene-1-sulfonamide **1d** (500 mg, 1.87 mmol) to afford imine **2d** as a white solid (612 mg, 98%) as a mixture of major and minor stereoisomers (10.1:1). NMR data quoted for the major isomer only. mp = 87–89 °C. IR (film)/cm^{-1} 3075, 2969, 2867, 1738, 1666, 1474, 1457, 1404, 1358, 1333, 1319, 1294, 1229, 1217, 1169, 1138, 1092, 1061, 1018. NMR data quoted for the major isomer only. ^{1}H NMR (400 MHz, $CDCl_3$) δ 8.01 (d, *J* = 8.2 Hz, 2H, Ar-H), 7.80 (t, *J* = 8.2 Hz, 2H, Ar-H), 7.51 (t, *J* = 1.2 Hz, 1H, N=CH), 4.98 (s, 1H, NH), 3.42 (td, *J* = 5.6, 1.2 Hz, 2H, CH=N*CH₂*), 3.24–3.17 (m, 2H, NH*CH₂*), 1.02 (s, 9H, $C(CH_3)_3$). ^{13}C NMR (101 MHz, $CDCl_3$) δ 175.0 (CH=N), 143.7 (Ar-C_q), 134.5 (q, J_{CF} = 33.1 Hz, Ar-C_q), 127.6 (2 × Ar-C), 126.3 (q, J_{CF} = 3.7 Hz, 2 × Ar-C), 123.4 (q, J_{CF} = 272.7 Hz, CF_3), 59.0 (CH_2), 43.9 (CH_2), 36.4 (*C*(CH_3)$_3$), 26.7 (C(*C*H$_3$)$_3$). ^{19}F NMR (377 MHz, CDCl3) δ –63.1. HRMS (ESI) m/z Calcd. for $C_{14}H_{18}N_2O_2F_3S$ [M+H]$^+$: 335.1034; Found: 335.1041.

2,6-Dichloro-_N_-{2-[(_E_)-(2,2-dimethylpropylidene)amino]ethyl}benzene-1-sulfonamide (2e)

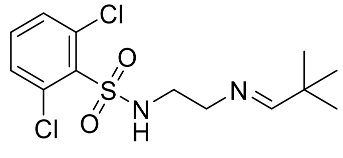

General procedure C was followed using *N*-(2-aminoethyl)-2,6-dichlorobenzene-1-sulfonamide **1e** (404 mg, 1.50 mmol), to afford imine **2e** as an off-white solid (405 mg, 80%) as a mixture of major and minor stereoisomers (8.1:1). NMR data quoted for the major isomer only. mp = 70–71 °C. IR (film)/cm^{-1} 3295, 2955, 2862, 1738, 1669, 1569, 1558, 1471, 1424, 1397, 1359, 1334, 1216, 1938, 1172, 1082, 1042, 1038. ^{1}H NMR (400 MHz, $CDCl_3$) δ 7.54 (t, *J* = 1.2 Hz, 1H, N=CH), 7.49–7.47 (m, 2H, Ar-H), 7.37–7.33 (m, 1H, Ar-H), 5.79, (bs, 1H, NH), 3.45 (td, *J* = 5.6, 1.1 Hz, 2H, CH=N*CH₂*), 3.26–3.23 (m, 2H, NH*CH₂*), 1.05 (s, 9H, $C(CH_3)_3$). ^{13}C NMR (101 MHz, $CDCl_3$) δ 174.9 (CH=N), 135.3 (Ar-C_q), 134.9 (2 × Ar-C_q) 132.3 (Ar-C), 131.4 (2 × Ar-C), 58.8 (CH=N*CH₂*), 44.0 ($NHCH_2$), 36.4 (*C*(CH_3)$_3$), 26.8 (C(*C*H$_3$)$_3$). HRMS (ESI) m/z Calcd. for $C_{13}H_{16}N_2O_2SCl_2$ [M+H]$^+$: 337.0544; Found: 337.0550.

N-{2-[(E)-(2,2-Dimethylpropylidene)amino]ethyl}-2,4,6-tris(propan-2-yl)benzene-1-sulfonamide (2f)

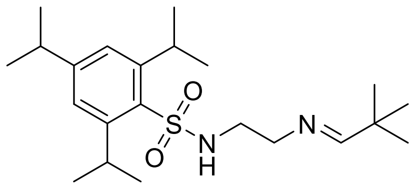

General procedure C was followed using *N*-(2-aminoethyl)-2,4,6-tris(propan-2-yl)benzene-1-sulfonamide **1f** (230 mg, 0.71 mmol) to afford imine **2f** as a white solid (270 mg, 97%) as a single stereoisomer. mp = 88–91 °C. IR (film)/cm^{-1} 3316, 2959, 2868, 1738, 1665, 1601, 1462, 1425, 1405, 1380, 1361, 1318, 1255, 1229, 1216, 1153, 1097, 1058, 1039. ^{1}H NMR (400 MHz, $CDCl_3$) δ 7.58 (t, *J* = 1.2 Hz, 1H, N=CH), 7.17 (s, 2H, Ar-H), 4.77 (t, *J* = 6.2 Hz, 1H, NH), 4.21–4.11 (m, 2H, *CH*(CH_3)$_2$), 3.48 (td, *J* = 5.6, 1.1 Hz, 2H, CH=N*CH$_2$*), 3.19–3.15 (m, 3H, NH*CH$_2$*), 2.96–2.85 (m, 2H, *CH*(CH_3)$_2$), 1.27 (t, *J* = 6.9 Hz, 18H, CH(*CH$_3$*)$_2$), 1.04 (s, 9H, C(CH_3)$_3$). ^{13}C NMR (101 MHz, $CDCl_3$) δ 174.7 (CH=N), 152.6 (Ar-C_q), 150.2 (3 × Ar-C_q), 123.8 (2 × Ar–C), 59.5 (CH=N*CH$_2$*), 43.4 ($NHCH_2$), 36.4 (*C*(CH_3)$_3$), 34.1 (*CH*(CH_3)$_2$), 29.6 (2 × *CH*(CH_3)$_2$), 26.8 (C(*CH$_3$*)$_3$), 24.9 (2 × CH(*CH$_3$*)$_2$), 23.6 (CH(*CH$_3$*)$_2$). HRMS (ESI) m/z Calcd. for $C_{22}H_{37}N_2O_2S$ [M+H]$^+$: 393.2576; Found: 393.2570.

N-{2-[(E)-(2,2-Dimethylpropylidene)amino]ethyl}butane-1-sulfonamide (2g)

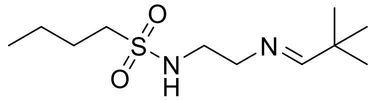

General procedure C was followed using *N*-(2-aminoethyl)butane-1-sulfonamide **1g** (400 mg, 2.22 mmol) to afford imine **2g** as a yellow oil (457 mg, 83%) as a mixture of major and minor stereoisomers (19:1). NMR data quoted for the major isomer only. IR (film)/cm^{-1} 3294, 2960, 2873, 1666, 1458, 1363, 1321, 1140 (s), 1096. ^{1}H NMR (400 MHz, $CDCl_3$) δ 7.61 (t, *J* = 1.2 Hz, 1H, N=CH), 4.59 (bs, 1H, NH), 3.51 (td, *J* = 5.7, 1.2 Hz, 2H, CH=N*CH$_2$*), 3.33–2.29 (m, 2H NH*CH$_2$*), 3.05–3.01 (m, 2H, CH_2), 1.83–1.75 (m, 2H, CH_2), 1.50–1.41 (m, 2H, CH_2), 1.06 (s, 9H, C(CH_3)$_3$), 0.95 (t, *J* = 7.4 Hz, 3H, CH_3). ^{13}C NMR (101 MHz, $CDCl_3$) δ 174.9 (CH=N), 60.0 (CH_2), 52.3 (CH_2), 43.9 (CH_2), 36.4 (*C*(CH_3)$_3$), 26.8 (C(*CH$_3$*)$_3$), 25.6 (CH_2), 21.5 (CH_2), 13.6 (CH_3). HRMS (ESI) m/z Calcd. for $C_{11}H_{24}N_2O_2S$ [M+H]$^+$: 249.1631; Found: 249.1629.

N-{2-[(E)-(2,2-Dimethylpropylidene)amino]ethyl}benzamide (2h)

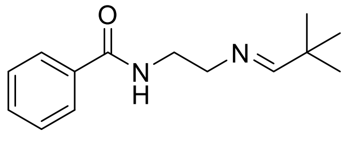

General procedure D was followed using *N*-(2-azaniumylethyl)benzamide trifluoroacetate **1h** (566 mg, 2.00 mmol) to afford imine **2h** as an off-white solid (404 mg, 87%) as a mixture of major and minor stereoisomers (7.3:1). NMR data quoted for the major isomer only. mp = 82–84 °C IR (film)/cm^{-1} 3319, 2966, 2907, 2861, 2819, 1671, 1644, 1602, 1579, 1547, 1457, 1358, 1303, 1260, 1205, 1115, 1020. ^{1}H NMR (400 MHz, $CDCl_3$) δ 7.78–7.75 (m, 2H, Ph-H), 7.60 (t, *J* = 1.2 Hz, 1H, N=CH), 7.52–7.48 (m, 1H, Ph-H), 7.45–7.41 (m, 2H, Ph-H), 6.53 (bs, 1H, NH), 3.71–3.66 (m, 2H, NH*CH$_2$*), 3.59 (t, *J* = 5.8 Hz, 2H, CH=N*CH$_2$*), 1.07 (s, 9H, C(CH_3)$_3$). ^{13}C NMR (101 MHz, $CDCl_3$) δ 174.3 (CH=N), 167.3 (C=O), 134.7 (Ph-C_q), 131.4 (Ph-C), 128.5 (2 × Ph-C), 126.8 (2 × Ph-C), 59.7 (CH=N*CH$_2$*), 40.6 ($NHCH_2$), 36.3 (*C*(CH_3)$_3$), 26.9 (C(*CH$_3$*)$_3$). HRMS (ESI) m/z Calcd. for $C_{14}H_{21}N_2O$ [M+H]$^+$: 233.1654; Found:. 233.1648.

2,2,2-Trichloro-*N*-{2-[(*E*)-(2,2-dimethylpropylidene)amino]ethyl}acetamide (2i)

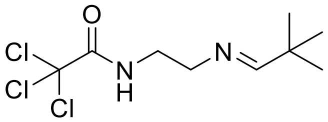

General procedure D was followed using *N*-(2-azaniumylethyl)-2,2,2-trichloroacetamide trifluoroacetate **1i** (383 mg, 1.20 mmol) to afford imine **2i** as an orange gum (328 mg, 100%) as a mixture of major and minor stereoisomers (7.3:1). NMR data quoted for the major isomer only. IR (film)/cm^{-1} 3311, 2961, 2862, 1693 (C=O), 1661, 1521, 1474, 1439, 1359, 1264, 1203, 1120. ^{1}H NMR (400 MHz, $CDCl_3$) δ 7.61 (t, *J* = 1.2 Hz, 1H, N=CH), 7.12 (bs, 1H, NH), 3.64–3.59 (m, 2H, NH*CH₂*), 3.57–3.54 (m, 2H, CH=N*CH₂*), 1.08 (s, 9H, $C(CH_3)_3$). ^{13}C NMR (101 MHz, $CDCl_3$) δ 174.9 (CH=N), 161.8 (C=O), 58.5 (CH=NCH_2), 41.7 ($NHCH_2$), 36.4 (*C*$(CH_3)_3$), 26.8 (C(*C*$H_3)_3$). HRMS (ESI) m/z Calcd. for $C_9H_{16}N_2OCl_3$ $[M+H]^+$: 273.0328; Found: 273.0323.

***N*-{2-[(*E*)-(2,2-Dimethylpropylidene)amino]ethyl}-2,6-difluorobenzamide (2j)**

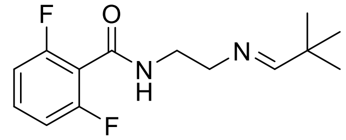

General procedure D was followed using *N*-(2-azaniumylethyl)-2,6-difluorobenzamide trifluoroacetate **1j** (453 mg, 1.50 mmol) to afford imine **2j** as a pale yellow solid (157 mg, 40%) as a mixture of major and minor stereoisomers (4.3:1). NMR data quoted for the major isomer only. mp = 65–70 °C. IR (film)/cm^{-1} 3267, 3097, 2959, 2867, 1650 (s, C=O), 1625 (s), 1592, 1564, 1465 (s), 1437, 1360, 1312, 1272, 1234, 1114, 1004 (s). ^{1}H NMR (400 MHz, $CDCl_3$) δ 7.62 (s, 1H, N=CH), 7.36 (tt, *J* = 8.5, 6.3 Hz, 1H, Ar-H), 6.97–6.91 (m, 2H, Ar-H), 6.27 (bs, 1H, NH), 3.73–7.68 (m, 2H, NHCH_2), 3.60–3.57 (m, 2H, CH=NCH_2), 1.06 (s, 9H, $C(CH_3)_3$). ^{13}C NMR (101 MHz, $CDCl_3$) δ 174.6 (CH=N), 160.3 (dd, J_{CF} = 253.0, 6.9 Hz, (2 × Ar-C_qF), 160.3 (C=O), 131.6 (t, J_{CF} = 10.4 Hz, FAr-C), 112.0 (m, 2 × FAr-C), 59.6 (CH=NCH_2), 40.6 ($NHCH_2$), 36.3 (*C*$(CH_3)_3$), 26.8 ($C(CH_3)_3$). ^{19}F NMR (377 MHz, $CDCl_3$) δ –112.2. HRMS (ESI) m/z Calcd. for $C_{14}H_{19}N_2OF_2$ $[M+H]^+$: 269.1465; Found: 269.1470.

***N*-{2-[(*E*)-(2,2-Dimethylpropylidene)amino]ethyl}-2,3,4,5,6-pentafluorobenzamide (2k)**

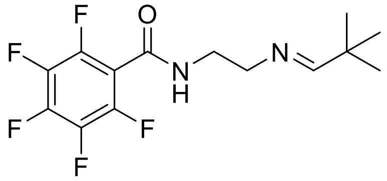

General procedure D was followed using *N*-(2-azaniumylethyl)-2,3,4,5,6-pentafluorobenzamide trifluoroacetate **1k** (552 mg, 1.50 mmol) to afford imine **2k** as a pale yellow solid (291 mg, 60%) as a mixture of major and minor stereoisomers (7.3:1). NMR data quoted for the major isomer only. mp = 74–75 °C. IR (film)/cm^{-1} 3289, 2964, 1738 (w), 1653 (s, C=O), 1555, 1520, 1359, 1331, 1269, 1114, 1066, 1047. ^{1}H NMR (400 MHz, $CDCl_3$) δ 7.63 (t, *J* = 1.1 Hz, 1H, N=CH), 6.39 (bs, 1H, NH), 3.71–3.67 (m, 2H, NHCH_2), 3.59–3.56 (m, 2H, CH=NCH_2), 1.07 (s, 9H, $C(CH_3)_3$). ^{13}C NMR (101 MHz, $CDCl_3$) δ 174.7 (CH=N), 157.3 (C=O), 59.1 (CH=NCH_2), 41.1 ($NHCH_2$), 36.3 (*C*$(CH_3)_3$), 26.8 (C(*C*$H_3)_3$), Ar-C signals not observed due to complex coupling. ^{19}F NMR (377 MHz, $CDCl_3$) δ –140.4 to –140.6 (m), –150.7 to –150.8 (m), –156.0 to –160.1 (m). HRMS (ESI) m/z Calcd. for $C_{14}H_{16}N_2OF_5$ $[M+H]^+$: 323.1183; Found: 323.1182.

tert-Butyl N-{2-[(E)-(2,2-dimethylpropylidene)amino]ethyl}carbamate (2l)

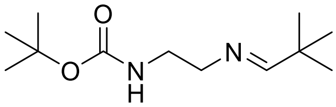

General procedure C was followed using *N*-Boc-ethylenediamine **1l** (636 mg, 4.00 mmol) to afford imine **2l** as a yellow oil (913 mg, 100%) as a mixture of major and minor stereoisomers (10.1:1). NMR data quoted for the major isomer only. IR (film)/cm^{-1} 3344, 2964, 1694 (C=O), 1667, 1512, 1477, 1453, 1364, 1268, 1249, 1167, 1117. ^{1}H NMR (400 MHz, $CDCl_3$) δ 7.53 (t, *J* = 1.1 Hz, 1H, N=CH), 4.74 (bs, 1H, NH), 3.45 (t, *J* = 5.7 Hz, 2H, CH=N*CH₂*), 3.34–3.30 (m, 2H, NH*CH₂*), 1.43 (s, 9H, $C(CH_3)_3$ (Boc)), 1.06 (s, 9H, N=CHC(CH_3)$_3$). ^{13}C NMR (101 MHz, $CDCl_3$) δ 174.1 (CH=N), 155.8 (C=O), 79.1 (*C*(CH_3)$_3$ (Boc)), 60.3 (CH=NCH_2), 41.2 (NHCH_2), 36.2 (*C*(CH_3)$_3$), 28.6 (C(CH_3)$_3$), 26.8 (C(*C*H_3)$_3$). HRMS (ESI) m/z Calcd. for $C_{12}H_{25}N_2O_2$ [M+H]$^+$: 229.1916; Found: 229.1922.

(*E*)-2,2-Dimethyl-N-(4-phenylbutyl)propan-1-imine (2m)

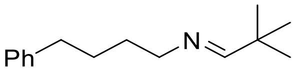

General procedure C was followed using 4-phenylbutan-1-amine **1m** (474 µL, 3.00 mmol) to afford imine **2m** as a yellow oil (699 mg, 100%) as a single stereoisomer. IR (film)/cm^{-1} 3027, 2932, 2861, 2863, 1667, 1496, 1453, 1363. ^{1}H NMR (400 MHz, $CDCl_3$) δ 7.50 (t, *J* = 1.2 Hz, 1H. N=CH), 7.30–7.26 (m, 2H, Ph-H), 7.20–7.16 (m, 3H, Ph-H), 3.38 (td, *J* = 6.9, 1.1 Hz, 2H CH=NCH_2), 2.63 (t, *J* = 6.9 Hz, 2H, NHCH_2), 1.65–1.60 (m, 4H, 2 × CH_2), 1.07 (s, 9H, C(CH_3)$_3$). ^{13}C NMR (101 MHz, $CDCl_3$) δ 171.9 (CH=N), 142.6 (Ph-C_q), 128.4 (2 × Ph-C), 128.2 (2 × Ph-C), 125.6 (Ph-C), 61.1 (CH_2), 35.9 (CH_2), 35.6 (*C*(CH_3)$_3$), 30.4 (CH_2), 28.9 (CH_2), 26.9 (C(*C*H_3)$_3$). HRMS (pNSI) m/z Calcd. for $C_{15}H_{24}N$ [M+H]$^+$: 218.1903; Found: 218.1904.

(*E*)-*N*-Benzyl-2,2-dimethylpropan-1-imine (2n)

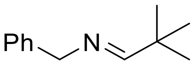

General procedure C was followed using benzylamine **1n** (327 µL mg, 3.00 mmol) to afford imine **2n** as a colourless amourphous solid (578 mg, 100%) as a single stereoisomer. IR (film)/cm^{-1} 2959, 2866, 2815, 1665, 1453, 1363. ^{1}H NMR (400 MHz, $CDCl_3$) δ 7.66 (t, *J* = 1.3 Hz, 1H, N=CH), 7.35–7.31 (m, 2H, Ph-H), 7.26–7.23 (m, 3H, Ph-H), 4.59 (s, 2H,CH_2), 1.13 (s, 9H, C(CH_3)$_3$). ^{13}C NMR (101 MHz, $CDCl_3$) δ 173.4 (CH=N), 139.7 (Ph-C_q), 128.3 (2 × Ph-C), 127.6 (2 × Ph-C), 126.7 (Ph-C), 64.5 (CH_2), 36.3 (*C*(CH_3)$_3$) 26.9 (C(*C*H_3)$_3$). Spectroscopic data for this compound (^{1}H NMR, ^{13}C NMR) is consistent with that shown in the literature.[7]

C(sp³)–H arylation of imines

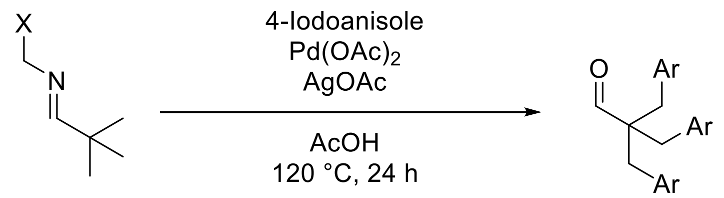

General Precedure E

Imine (0.40 mmol), 1-iodo-4-methoxybenzene (243 mg, 1.04 mmol), palladium acetate (9.0 mg, 10 mol%), silver acetate (133 mg, 0.80 mmol) and acetic acid (1.3 mL) were combined in a flame dried microwave vial. The vial was purged with argon, sealed and heated to 120 °C for 24 h. The reaction was allowed to cool to room temperature, dissolved in CH_2Cl_2, filtered through a short plug of silica, washed with CH_2Cl_2 and concentrated under reduced pressure. Yields of the arylated aldehyde products were calculated by ^{1}H NMR using *gem*-dimethyl (mono: 1.05 ppm), methyl (di: 0.97 ppm) and methylene (tri: 2.85 ppm) signals in comparison to a known amount of 1,3,5-trimethoxybenzene as an internal standard.

Directing group selection

Selection of the directing group to use for examination of the reaction scope considered both the yield of **3**, giving priority to monoarylation product **3a**, as well as the presence of side products as shown by increased complexity in the ^{1}H NMR spectrum. For example, in the imine arylation reactions, sulfonamide directing groups gave marginally lower yields of **3** than the amide directing groups, however, they gave a considerably less complex aldehyde region by ^{1}H NMR and better selectivity of the monoarylated product (figure S7).

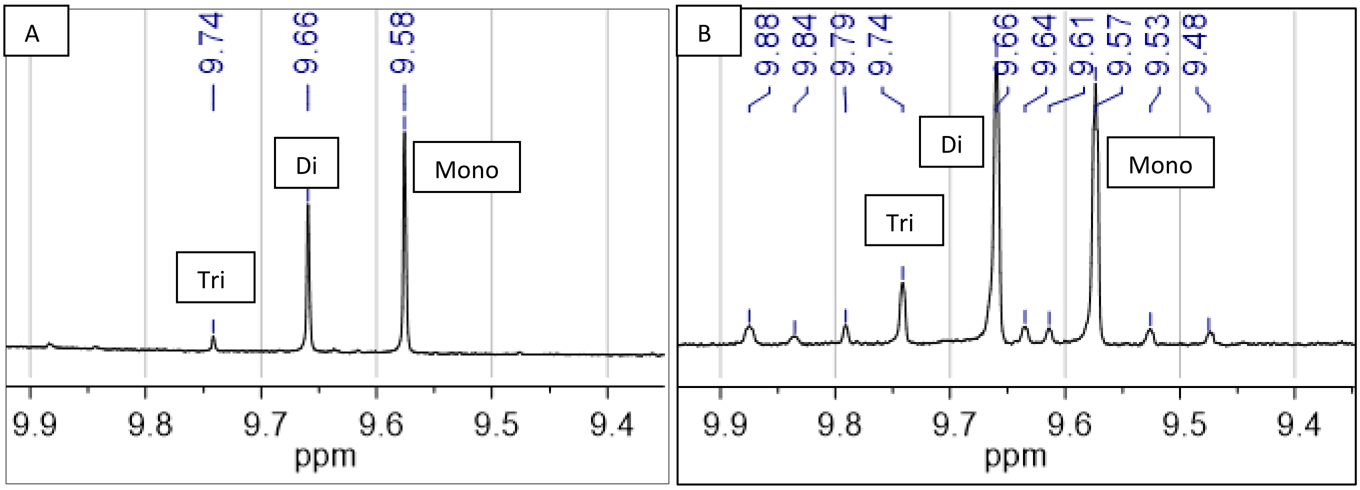

Figure S7: Aldehyde regions of imine arylation crude ^{1}H NMR using A) N-{2-[(*E*)-(2,2-dimethylpropylidene)amino]ethyl}-4-methylbenzene-1-sulfonamide **(2a)** and B) 2,2,2-trichloro-*N*-{2-[(*E*)-(2,2-dimethylpropylidene)amino]ethyl}acetamide **(2i)**.

Preparation of aldehydes (S5-S22)

Preparation of aldehydes (S7, S10, S13)

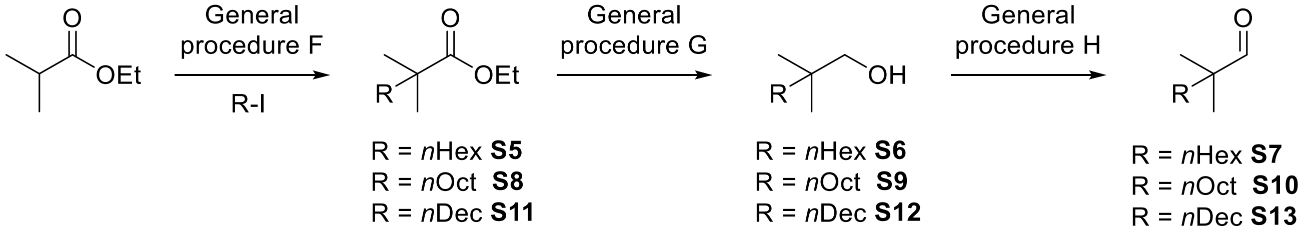

General Procedure F

Based on conditions developed by Shi.[8] *n*-Butyllithium (2.76 mL, 2.28 M in hexanes) was added dropwise to a stirred solution of freshly distilled diisopropylamine (883 µL, 6.30 mmol) in THF (6 mL) at -78 °C. The reaction was warmed to 0 °C and stirred for 30 min. The prepared LDA was then cooled to -78 °C and ethyl isobutyrate (806 µL, 6.00 mmol) was added dropwise and the reaction stirred at -78 °C for 1 h. The iodoalkane (6.18 mmol, 1.03 equiv) was added dropwise and the reaction stirred at room temperature overnight. The reaction was poured into ice water and extracted with diethyl ether (3 × 15 mL), the combined organic extracts were washed with brine, dried ($MgSO_4$), filtered and concentrated under reduced pressure. Purification by flash chromatography (SiO_2, diethyl ether/pentane) afforded the corresponding alkylated ester.

General Procedure G

DIBAL (5.25 mL, 1 M in hexane) was added dropwise to a stirred solution of ester (2.50 mmol) in CH_2Cl_2 (2.8 mL). The reaction was warmed to 0 °C and stirred overnight. The reaction was quenched by addition of MeOH (1.25 mL), diluted with brine and filted through Celite, the aqueous phase was extracted with Diethyl ether (3 × 15 mL), dried ($MgsO_4$), fitered and concentrated under reduced pressure. Purification by flash chromatography (SiO_2, Diethyl ether/pentane) affored the corresponding alcohol.

General Procedure H

Dess-Martin periodane (1.2 equiv) was added to a stirred solution of alcohol (1 equiv) in CH_2Cl_2 (0.2 M) at 25 °C and the reaction was stirred for 1 h. The reaction was quenched by addition of aqueous sodium thiosulfate (10% w/v) and the crude product extracted with CH_2Cl_2. The combined organic extracts were dried ($MgSO_4$), filted and concentrated under reduced pressure. Purification by flash chromatography (SiO_2, Diethyl ether/pentane) affored the corresponding aldehyde.

Ethyl 2,2-dimethyloctanoate (S5)

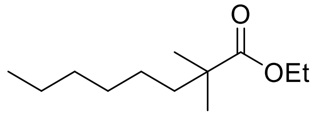

General procedure F was followed using 1-iodohexane (912 µL, 6.18 mmol) to afford alkylated ester **S5** as a colourless oil (1.07 g, 89%). R_f 0.63 (10% diethyl ether/pentane). IR (film)/cm^{-1} 2930, 2859, 1728 (s, C=O), 1472, 1176, 1144, 1029. ^{1}H NMR (400 MHz, $CDCl_3$) δ 4.12 (q, *J* = 7.1 Hz, 2H, OCH_2), 1.52–1.48 (m, 2H, CH_2), 1.32–1.18 (m, 11H, 4 × CH_2 + CH_3), 1.16 (s, 6H, $C(CH_3)_2$), 0.88 (t, *J* = 6.8 Hz, 3H, CH_3). ^{13}C NMR (101 MHz, $CDCl_3$) δ 178.2 (C=O), 60.1 (OCH_2), 42.2 ($\mathit{C}(CH_3)_2$), 40.8 (CH_2), 31.7 (CH_2), 29.8 (CH_2), 25.1 ($C(\mathit{C}H_3)_2$), 24.8 (CH_2), 22.6 (CH_2), 14.2 (CH_3), 14.1 (CH_3). HRMS (pNSI) m/z Calcd. For $C_{12}H_{25}O_2$ $[M+H]^+$: 201.1849; Found: 201.1849. Spectroscopic data for this compound (^{1}H NMR) is consistent with that shown in the literature.[9]

2,2-Dimethyloctan-1-ol (S6)

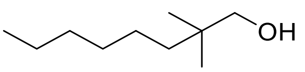

General procedure G was followed using ethyl 2,2-dimethyloctanoate **S5** (501 mg, 2.50 mmol) to afford alcohol **S6** as a colourless oil (233 mg, 59%). R_f 0.15 (20% diethyl ether/pentane). IR (film)/cm^{-1} 3323 (br, OH), 2858, 2927, 2858, 1468, 1363, 1041. ^{1}H NMR (400 MHz, $CDCl_3$) δ 3.32 (s, 2H, CH_2), 1.34–1.20 (m, 11H, 5 × CH_2 + OH), 0.91–0.87 (m, 9H, 3 × CH_3). ^{13}C NMR (101 MHz, $CDCl_3$) δ 72.1 (OCH_2), 38.7 (CH_2), 35.0 (*C*(CH_3)$_2$), 31.9 (CH_2), 30.3 (CH_2), 23.83 (C(*C*H_3)$_2$), 23.81 (CH_2), 22.7 (CH_2), 14.1 (CH_3). Spectroscopic data for this compound (^{1}H NMR) is consistent with that shown in the literature.[9]

2,2-Dimethyloctanal (S7)

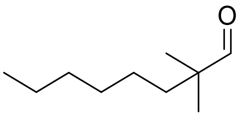

General procedure H was followed using ethyl 2,2-dimethyloctanol **S6** (223 mg, 1.41 mmol) to afford aldehyde **S7** as a colourless oil (177 mg, 79%). R_f 0.50 (5% diethyl ether/pentane). IR (film)/cm^{-1} 2959, 2929, 2858, 2690, 1726 (s, C=O), 1468. ^{1}H NMR (400 MHz, $CDCl_3$) δ 9.45 (s, 1H, CHO), 1.48–1.44 (m, 2H, CH_2), 1.33–1.15 (m, 8H, 4 × CH_2), 1.05 (s, 6H, C(CH_3)$_2$), 0.92–0.84 (t, *J* = 6.8 Hz, 3H, CH_3). ^{13}C NMR (101 MHz, $CDCl_3$) δ 206.6 (C=O), 45.8 (*C*(CH_3)$_2$), 37.3 (CH_2), 31.6 (CH_2), 29.9 (CH_2), 24.2 (CH_2), 22.6 (CH_2), 21.3 (C(*C*H_3)$_2$), 14.0 (CH_3). HRMS (ASAP(SOLID)) m/z Calcd. For $C_{10}H_{21}O$ $[M+H]^+$: 157.1592; Found: 157.1587. Spectroscopic data for this compound (^{1}H NMR) is consistent with that shown in the literature.[9]

Ethyl 2,2-dimethyldecanoate (S8)

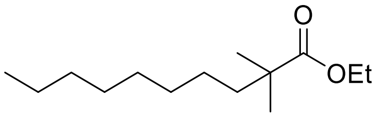

General procedure F was followed using 1-iodooctane (1.12 mL, 6.18 mmol) to afford alkylated ester **S8** as a colourless oil (846 mg, 62%). R_f 0.63 (10% diethyl ether/pentane). IR (film)/cm^{-1} 2925, 2855, 1729 (s, C=O), 1469, 1174, 1143, 1028. ^{1}H NMR (400 MHz, $CDCl_3$) δ 4.12 (q, *J* = 7.1 Hz, 2H, OCH_2), 1.52–1.48 (m, 2H, CH_2), 1.34–1.17 (m, 15H, 6 × CH_2 + CH_3), 1.16 (s, 6H, C(CH_3)$_2$), 0.88 (t, *J* = 6.9 Hz, 3H, CH_3). ^{13}C NMR (101 MHz, $CDCl_3$) δ 178.2 (C=O), 60.1 (OCH_2), 42.2 (*C*(CH_3)$_2$), 40.8 (CH_2), 31.9 (CH_2), 30.1 (CH_2), 29.4 (CH_2), 29.3 (CH_2), 25.1 (C(*C*H_3)$_2$), 24.9 (CH_2), 22.7 (CH_2), 14.2 (CH_3), 14.1 (CH_3). HRMS (ESI) m/z Calcd. For $C_{14}H_{29}O_2$ $[M+H]^+$: 229.2168; Found: 229.2177.

2,2-Dimethyldecan-1-ol (S9)

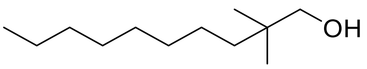

General procedure G was followed using ethyl 2,2-dimethyldecanoate **S8** (580 mg, 2.50 mmol) to afford alcohol **S9** as a colourless oil (361 mg, 78%). R_f 0.22 (20% diethyl ether/pentane). IR (film)/cm^{-1} 3358 (br, OH), 1738, 1467, 1363, 1038. ^{1}H NMR (400 MHz, $CDCl_3$) δ 3.32 (s, 2H, OCH_2), 1.42 (bs, 1H, OH), 1.33–1.20 (m, 14H, 7 × CH_2), 0.91–0.87 (m, 9H, 3 x CH_3). ^{13}C NMR (101 MHz, $CDCl_3$) δ 72.1 (OCH_2), 38.7 (CH_2), 35.0 (*C*(CH_3)$_2$), 31.9 (CH_2), 30.6 (CH_2), 29.6 (CH_2), 29.3 (CH_2), 23.9 (CH_2), 23.8 (C(*C*H_3)$_2$), 22.7 (CH_2), 14.1 (CH_3). HRMS (ASAP(DCM)) m/z Calcd. For $C_{12}H_{25}O$ $[M-H]^-$: 185.1905; Found: 185.1901.

2,2-Dimethyldecanal (S10)

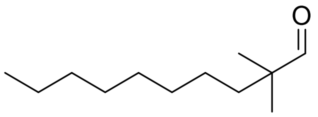

General procedure H was followed using ethyl 2,2-dimethyldecanol **S9** (254 mg, 1.41 mmol) to afford aldehyde **S10** as a colourless oil (209 mg, 81%). R_f 0.41 (5% diethyl ether/pentane). IR (film)/cm^{-1} 2958, 2926, 2855, 1727, 1467. ^{1}H NMR (400 MHz, $CDCl_3$) δ 9.45 (s, 1H, CHO), 1.47–1.43 (m, 2H, CH_2), 1.30–1.20 (m, 12H, 6 × CH_2), 1.04 (s, 6H, $C(CH_3)_2$), 0.88 (t, *J* = 6.9 Hz, 3H, CH_3). 13C NMR (101 MHz, CDCl3) δ 206.6 (C=O), 45.8 (*C*$(CH_3)_2$), 37.4 (CH_2), 31.8 (CH_2), 30.2 (CH_2), 29.4 (CH_2), 29.2 (CH_2), 24.3 (CH_2), 22.6 (CH_2), 21.3 (C(*C*$H_3)_2$), 14.1 (CH_3). HRMS (ASAP(SOLID)) m/z Calcd. For $C_{12}H_{25}O$ $[M+H]^+$: 185.1905; Found: 185.1903. Spectroscopic data for this compound (^{1}H NMR) is consistent with that shown in the literature.[10]

Ethyl 2,2-dimethyldodecanoate (S11)

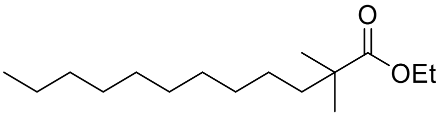

General procedure F was followed using 1-iododecane (1.32 mL, 6.18 mmol) to afford alkylated ester **S11** as a colourless oil (945 mg, 61%). R_f 0.15 (Pentane). IR (film)/cm^{-1} 2924, 2854, 1729 (s, C=O), 1467, 1173, 1142, 1028. ^{1}H NMR (400 MHz, $CDCl_3$) δ 4.12 (q, *J* = 7.1 Hz, 2H, OCH_2), 1.52–1.48 (m, 2H, CH_2), 1.30–1.20 (m, 19H, 8 × CH_2 + CH_3), 1.16 (s, 6H $C(CH_3)_2$), 0.89 (t, *J* = 6.9 Hz, 3H, CH_3). ^{13}C NMR (101 MHz, $CDCl_3$) δ 178.2 (C=O), 60.1 (OCH_2), 42.2 (*C*$(CH_3)_2$), 40.8 (CH_2), 31.9 (CH_2), 30.1 (CH_2), 29.6 (2 × CH_2), 29.5 (CH_2), 29.3 (CH_2), 25.1 (C(*C*$H_3)_2$), 24.9 (CH_2), 22.7 (CH_2), 14.2 (CH_3), 14.1 (CH_3). HRMS (pNSI) m/z Calcd. for $C_{16}H_{33}O_2$ $[M+H]^+$: 257.2475; Found: 257.2478.

2,2-Dimethyldodecan-1-ol (S12)

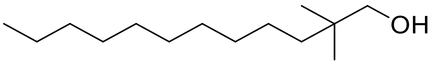

General procedure G was followed using ethyl 2,2-dimethyldodecanoate **S11** (641 mg, 2.50 mmol) to afford alcohol **S12** as a colourless oil (447 mg, 83%). R_f 0.31 (20% diethyl ether/pentane). IR (film)/cm^{-1} 3358 (br, OH), 2955, 2923 (s), 2853, 1738, 1467, 1364, 1037. ^{1}H NMR (400 MHz, $CDCl_3$) δ 3.32 (s, 2H, CH_2), 1.32–1.20 (m, 19H, 9 × CH_3 + OH), 0.90–0.87 (m, 9H, 3 × CH_3). ^{13}C NMR (101 MHz, $CDCl_3$) δ 72.1 (OCH_2), 38.7 (CH_2), 35.0 (*C*$(CH_3)_2$), 31.9 (CH_2), 30.6 (CH_2), 29.7 (CH_2), 29.6 (2 × CH_2), 29.3 (CH_2), 23.9 (CH_2), 23.8 (C(*C*$H_3)_2$), 22.7 (CH_2), 14.1 (CH_3). HRMS (ASAP(DCM)) m/z Calcd. For $C_{14}H_{29}O$ $[M-H]^-$: 213.2218; Found: 213.2214.

2,2-Dimethyldodecanal (S13)

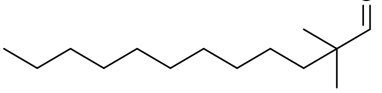

General procedure H was followed using ethyl 2,2-dimethyldodecanol **S12** (302 mg, 1.41 mmol) to afford aldehyde **S13** as a colourless oil (193 mg, 64%). R_f 0.47 (5% diethyl ether/pentane). IR (film)/cm^{-1} 2924, 2854, 1728 (s, C=O), 1467, 1365, 1217. ^{1}H NMR (400 MHz, $CDCl_3$) δ 9.45 (s, 1H, CHO), 1.47–1.43 (m, 2H, CH_2), 1.30–1.20 (m, 18H, 9 × CH_2), 1.04 (s, 6H, $C(CH_3)_2$), 0.89 (t, *J* = 6.9 Hz, 3H, CH_3). ^{13}C NMR (101 MHz, $CDCl_3$) δ 206.6 (C=O), 45.8 (*C*$(CH_3)_2$), 37.4 (CH_2), 31.9 (CH_2), 30.2 (CH_2), 29.6 (2 × CH_2), 29.5 (CH_2), 29.3 (CH_2), 24.3 (CH_2), 22.7 (CH_2), 21.3 (C(*C*$H_3)_2$), 14.1 (CH_3). HRMS (ASAP(SOLID)) m/z Calcd. For $C_{14}H_{29}O$ $[M+H]^+$: 213.2218; Found: 213.2218.

Preparation of aldehydes (S15, S17, S19, S22)

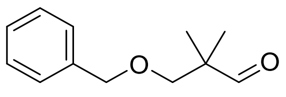

S15

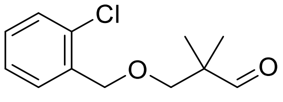

S17

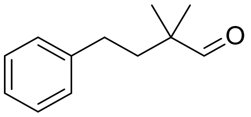

S19

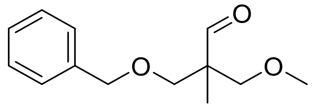

S22

3-(Benzyloxy)-2,2-dimethylpropan-1-ol (S14)

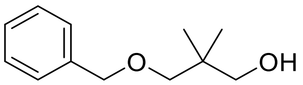

Sodium hydride (400 mg, 10.0 mmol, as a 60% dispersion in mineral oil) was slowly added to a solution of 2,2-dimethylpropane-1,3-diol (1.04 g, 10.0 mmol) in THF (35 mL) at 0 °C. After 10 minutes at 0 °C benzyl bromide (950 µL, 8.00 mmol) was added dropwise and the reaction was stirred at 25 °C over the weekend. The reaction was quenched by addition of saturated aqueous ammonium chloride solution (35 mL) and the aqueous layer was extracted with CH_2Cl_2 (3 × 35 mL). The combined organic layers were dried ($MgSO_4$), filtered and solvent removed under reduced pressure. Purification by flash chromatography (25% EtOAc/pentane) afforded benzylated alcohol **S14** as a pale yellow oil (1.07 g, 69%). R_f 0.42 (25% EtOAc/pentane). IR (film)/cm^{-1} 3399 (br, OH), 2955, 2869, 1453, 1360, 1094, 1044. ^{1}H NMR (400 MHz, $CDCl_3$) δ 7.40–7.28 (m, 5H, Ar-H), 4.53 (s, 2H, OCH_2), 3.47 (d, *J* = 5.8 Hz, 2H, $C\textit{H}_2$OH), 3.34 (s, 2H, OCH_2), 2.69 (t, *J* = 5.8 Hz, 1H, OH), 0.95 (s, 6H, $C(CH_3)_2$). ^{13}C NMR (101 MHz, $CDCl_3$) δ 138.1 (Ph-C_q), 128.4 (2 × Ph-C), 127.6 (Ph-C), 127.4 (2 × Ph-C), 79.3 (CH_2), 73.5 (CH_2), 71.6 (CH_2), 36.2 (*C*$(CH_3)_2$), 21.8 (C(*C*$H_3)_2$). Spectroscopic data for this compound (^{1}HNMR),[11] (^{13}C NMR)[12] and (IR)[13] is consistent with that shown in the literature.

3-(Benzyloxy)-2,2-dimethylpropanal (S15)

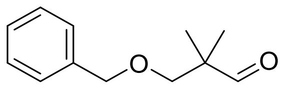

General procedure G was followed using 3-(benzyloxy)-2,2-dimethylpropan-1-ol **S14** (365 mg, 1.88 mmol) to afford aldehyde **S15** as a colourless oil (292 mg, 81%). R_f 0.46 (10% EtOAc/pentane). IR (film)/cm^{-1} 2971, 2931, 1726 (m, C=O), 1454, 1245, 1095, 1012. ^{1}H NMR (400 MHz, $CDCl_3$) δ 9.58 (s, 1H, CHO), 7.38–7.28 (m, 5H, Ar-H), 4.52 (s, 2H, OCH_2), 3.46 (s, 2H, OCH_2), 1.10 (s, 6H, $C(CH_3)_2$). ^{13}C NMR (101 MHz, $CDCl_3$) δ 205.3 (CHO), 138.1 (Ph-C_q), 128.4 (2 × Ph-C), 127.6 (Ph-C), 127.4 (2 × Ph-C), 75.1 (CH_2), 73.4 (CH_2), 47.1 (*C*$(CH_3)_2$), 19.0 (C(*C*$H_3)_2$). Spectroscopic data for this compound (^{1}H NMR),[12] (^{13}C NMR)[14] and (IR)[13] is consistent with that shown in the literature.

3-((2-Chlorobenzyl)oxy)-2,2-dimethylpropan-1-ol (S16)

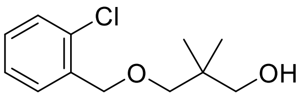

Sodium hydride (400 mg, 10.0 mmol, as a 60% dispersion in mineral oil) was slowly added to a solution of 2,2-dimethylpropane-1,3-diol (1.04 g, 10.0 mmol) in THF (35 mL) at 0 °C. After 10 minutes at 0 °C, 2-chlorobenzyl bromide (1.04 mL, 8.00 mmol) was added dropwise and the reaction was stirred at 25 °C overnight. The reaction was quenched by addition of saturated aqueous ammonium chloride solution (35 mL) and the aqueous layer was extracted with CH_2Cl_2 (3 × 35 mL). The combined organic layers were dried ($MgSO_4$), filtered and solvent removed under reduced pressure. Purification by flash chromatography (25% EtOAc/pentane) afforded benzylated alcohol **S16** as a pale yellow oil (819 mg, 40%). R_f 0.18 (20% EtOAc/pentane). IR (film)/cm^{-1} 3390 (br, OH), 2956, 2870, 1473, 1443, 1355, 1098, 1049. ^{1}H NMR (400 MHz, $CDCl_3$) δ 7.45–7.43 (m, 1H, Ar-H), 7.38–7.35 (m, 1H, Ar-H), 7.30–7.22 (m, 2H, Ar-H), 4.61 (s, 2H, CH_2), 3.49 (s, 2H, CH_2), 3.41 (s, 2H, CH_2), 2.46 (bs, 1H, OH), 0.97 (s, 6H, $C(CH_3)_2$). ^{13}C NMR (101 MHz, $CDCl_3$) δ 135.8 (Ar-C_q), 133.0 (Ar-C_q), 129.3 (Ar-C), 128.9 (Ar-C), 128.8 (Ar-C), 126.8 (Ar-C), 79.7 (CH_2), 71.6 (CH_2), 70.7 (CH_2), 36.4 (*C*$(CH_3)_2$), 21.9 (C(*C*$H_3)_2$). HRMS (ESI) m/z Calcd. For $C_{12}H_{18}O_2Cl$ $[M+H]^+$: 229.0995; Found: 229.0993.

3-((2-Chlorobenzyl)oxy)-2,2-dimethylpropanal (S17)

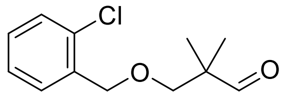

General procedure G was followed using 3-((2-chlorobenzyl)oxy)-2,2-dimethylpropan-1-ol **S16** (345 mg, 1.50 mmol) to afford aldehyde **S17** as a colourless oil (200 mg, 59%). R_f 0.40 (10% EtOAc/pentane). IR (film)/cm^{-1} 2969, 2872 1730 (s, C=O), 1443, 1472, 1098, 1051. ^{1}H NMR (400 MHz, $CDCl_3$) δ 9.61 (s, 1H, CHO), 7.43–7.41 (m, 1H, Ar-H), 7.35–7.33 (m, 1H, Ar-H), 7.28–7.19 (m, 2H, Ar-H), 4.60 (s, 2H, CH_2), 3.56 (s, 2H, CH_2), 1.13 (s, 6H, $C(CH_3)_2$). ^{13}C NMR (101 MHz, $CDCl_3$) δ 205.1 (CHO), 135.7 (Ar-C_q), 132.6 (Ar-C_q), 129.1 (Ar-C), 128.62 (Ar-C), 128.56 (Ar-C), 126.7 (Ar-C), 75.6 (CH_2), 70.3 (CH_2), 47.1 ($\mathit{C}(CH_3)_2$), 19.0 ($C(\mathit{C}H_3)_2$). HRMS (ESI) m/z Calcd. For $C_{12}H_{16}O_2Cl$ $[M+H]^+$: 227.0833; Found: 227.0835.

2,2-Dimethyl-4-phenylbutan-1-ol (S18)

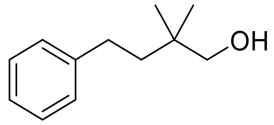

Conditions developed by Madsen.[15] Benzyl magnesium bromide (2.5 mL, 0.94 M in THP, formed from magnesium turnings and benzyl bromide in THP using standard techniques) and 3,3-dimethyloxetane (0.5 mL, 4.85 mmol) were combined in a 2–5 mL microwave vial. The vial was purged with Ar, sealed, and heated to 180 °C in a microwave reactor for 4 h. The reaction was allowed to cool to room temperature, diluted with Diethyl ether, quenched with water and the aqueous phase extracted with Diethyl ether (3 × 20 mL). The combined organic extracts were washed with saturated aqueous ammonium chloride solution, water and brine, dried ($MgSO_4$), filtered and concentrated under reduced pressure. Purification by flash chromatography (SiO_2, 20% EtOAc/pentane) afforded alcohol **S18** as a pale yellow oil (252 mg, 60%). R_f 0.38 (10% diethyl ether/pentane). IR (film)/cm^{-1} 3355 (br, OH), 2953, 2868, 1496, 1472, 1454, 1364, 1047, 1030. ^{1}H NMR (400 MHz, $CDCl_3$) δ 7.31–7.27 (m, 2H, Ar-H), 7.21–7.17 (m, 3H, Ar-H), 3.39 (s, 2H, OCH_2), 2.62–2.57 (m, 2H, CH_2), 1.60–1.56 (m, 2H, CH_2), 1.39 (bs, 1H, OH), 0.97 (s, 6H, $C(CH_3)_2$). ^{13}C NMR (101 MHz, $CDCl_3$) δ 143.1 (Ph-C_q), 128.34 (2 × Ph-C), 128.27 (2 × Ph-C), 125.6 (Ph-C), 71.8 (OCH_2), 40.9 (CH_2), 35.3 ($\mathit{C}(CH_3)_2$), 30.5 (CH_2), 23.8 ($C(\mathit{C}H_3)_2$). Spectroscopic data for this compound (^{1}H NMR, ^{13}C NMR, IR) is consistent with that shown in the literature.[16]

2,2-Dimethyl-4-phenylbutanal (S19)

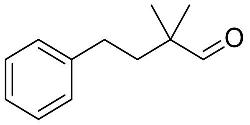

General procedure G was followed using 2,2-dimethyl-4-phenylbutan-1-ol **S18** (178 mg, 1.00 mmol) to afford aldehyde **S19** as a colourless oil (151 mg, 86%). R_f 0.32 (5% diethyl ether/pentane). IR (film)/cm^{-1} 1965, 2868, 1696, 1723 (s, C=O), 1497, 1469, 1454, 1366. ^{1}H NMR (400 MHz, $CDCl_3$) δ 9.50 (s, 1H, CHO), 7.31–7.28 (m, 2H, Ph-H), 7.22–7.17 (m, 3H, Ph-H), 2.56–2.52 (m, 2H, CH_2), 1.82–1.78 (m, 2H, CH_2), 1.14 (s, 6H, $C(CH_3)_2$). ^{13}C NMR (101 MHz, $CDCl_3$) δ 205.9 (CHO), 141.9 (Ph-C_q), 128.5 (2 × Ph-C), 128.2 (2 × Ph-C), 126.0 (Ph-C), 45.9 ($\mathit{C}(CH_3)_2$), 39.3 (CH_2), 30.8 (CH_2), 21.4 ($C(\mathit{C}H_3)_2$). Spectroscopic data for this compound (^{1}H NMR, ^{13}C NMR, IR) is consistent with that shown in the literature.[17]

3-((Benzyloxy)methyl)-3-methyloxetane (S20)

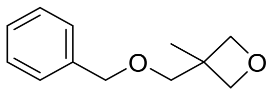

Sodium hydride (440 mg, 11.0 mmol, as a 60% dispersion in mineral oil) was slowly added to a solution of (3-methyloxetan-3-yl)methanol (998 µL, 10.0 mmol) in THF (35 mL) at 0 °C. After 10 minutes at 0 °C, benzyl bromide (1.31 mL, 11.0 mmol) was added dropwise and the reaction was stirred at 25 °C overnight. The reaction was quenched by addition of saturated aqueous ammonium chloride solution (35 mL) and the aqueous layer was extracted with CH_2Cl_2 (3 × 35 mL). The combined organic layers were dried ($MgSO_4$), filtered and solvent removed under reduced pressure. Purification by flash chromatography (20% EtOAc/pentane) afforded benzylated alcohol **S20** as a colourless oil (1.49 g, 77%). R_f 0.55 (20% EtOAc/pentane). IR (film)/cm^{-1} 2957, 2931, 2864 (m), 1453, 1361, 1093 (s), 976 (s). ^{1}H NMR (400 MHz, $CDCl_3$) δ 7.39–7.30 (m, 5H, Ar-H), 4.59 (s, 2H, CH_2), 4.54 (d, *J* = 5.7 Hz, 2H, 2 × OC(*H*)H), 4.38 (d, *J* = 5.7 Hz, 2H, 2 × OC(H)*H*), 3.54 (s, 2H, CH_2), 1.35 (s, 3H, CH_3). ^{13}C NMR (101 MHz, $CDCl_3$) δ 138.3 (Ph-C_q), 128.4 (2 × Ph-C), 127.63 (Ph-C), 127.55 (2 × Ph-C), 80.2 (2 × CH_2), 75.4 (CH_2), 73.3 (CH_2), 39.9 (*C*(CH_3)), 21.4 (CH_3). Spectroscopic data for this compound (^{1}H NMR),[18] (^{13}C NMR)[19] and (IR)[18] is consistent with that shown in the literature.

3-(Benzyloxy)-2-(methoxymethyl)-2-methylpropan-1-ol (S21)

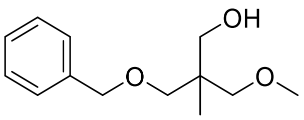

5 drops of H_2SO_4 (98%) was added to a solution of 3-((benzyloxy)methyl)-3-methyloxetane **S20** (481 mg, 2.5 mmol) in MeOH (2.5 mL) at 25 °C. The reaction was heated to 65 °C in a sealed vial and stirred for 1 h. The reaction mixture was neutralised by slow addition of saturated aqueous sodium bicarbonate solution (5 mL) and the aqueous layer extracted with diethyl ether (3 × 10 mL). The combined organic extracts were dried ($MgSO_4$), filtered and solvent removed under reduced pressure. Purification by flash chromatography (20% EtOAc/pentane) afforded alcohol **S21** as a pale yellow oil (377 mg, 67%). R_f 0.48 (20% diethyl ether/pentane). IR (film)/cm^{-1} 3448 (br, OH), 2875, 1453, 1363, 1197, 1094. ^{1}H NMR (400 MHz, $CDCl_3$) δ 7.38–7.28 (m, 5H, Ph-H), 4.53 (s, 2H, CH_2), 3.59 (d, *J* = 5.8 Hz, 2H, CH_2), 3.50–3.36 (m, 4H, 2 × CH_2), 3.34 (s, 3H, OCH_3), 2.86 (t, *J* = 6.0 Hz, 1H, OH), 0.89 (s, 3H, CH_3). ^{13}C NMR (101 MHz, $CDCl_3$) δ 138.3 (Ph-C_q), 128.4 (2 × Ph-C), 127.6 (Ph-C), 127.4 (2 × Ph-C), 77.5 (CH_2), 74.7 (CH_2), 73.5 (CH_2), 69.3 (CH_2), 59.4 (OCH_3), 40.5 (*C*(CH_3)), 17.5 (CH_3). HRMS (ESI) m/z Calcd. for $C_{13}H_{21}O_3$ [M+H]$^+$: 225.1491; Found: 225.1491.

3-(Benzyloxy)-2-(methoxymethyl)-2-methylpropanal (S22)

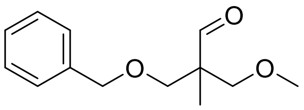

General procedure G was followed using 3-(benzyloxy)-2-(methoxymethyl)-2-methylpropan-1-ol **S21** (224 mg, 1.00 mmol) to afford aldehyde **S22** as a colourless oil (127 mg, 57%). R_f 0.23 (10% diethyl ether/pentane). IR (film)/cm^{-1} 2861, 1728 (s, C=O), 1453, 1365, 1203, 1098 (s). ^{1}H NMR (400 MHz, $CDCl_3$) δ 9.65 (s, 1H, CHO), 7.37–7.29 (m, 5H, Ar-H), 4.52 (s, 2H, CH_2), 3.65–3.48 (m, 4H, 2 × CH_2), 3.33 (s, 3H, OCH_3), 1.11 (s, 3H, CH_3).^{13}C NMR (101 MHz, $CDCl_3$) δ 204.6 (CHO), 138.1 (Ph-C_q), 128.3(2 × Ph-C), 127.6 (Ph-C), 127.4 (2 × Ph-C), 74.0 (CH_2), 73.4 (CH_2), 71.3 (CH_2), 59.4 (OCH_3), 51.7 (*C*(CH_3)), 14.9 (CH_3). HRMS (pNSI) m/z Calcd. for $C_{13}H_{19}O_3$ [M+H]$^+$: 223.1329; Found: 223.1328.

C(sp^3)–H arylation of aldehydes with a transient directing group (compounds 3-20)

General Procedure J

Aldehyde (0.40 mmol), *N*-(2-aminoethyl)-4-methylbenzenesulfonamide (43 mg, 0.20 mmol), aryl iodide (2.6 equiv), palladium pivalate (6.2 mg, 5 mol%), silver trifluoroacetate (176 mg, 0.80 mmol), DMSO (28.4 µL, 0.40 mmol), acetic acid (0.4 mL) and HFIP (0.4 mL) were combined in a flame dried microwave vial. The vial was purged with argon, sealed and heated to 130 °C for 3 h. The reaction was allowed to cool to room temperature, dissolved in CH_2Cl_2, filtered through a short plug of silica, washed with CH_2Cl_2 and concentrated under reduced pressure. Purification by flash chromatography (SiO_2) afforded the arylated aldehydes.

3-(4-Methoxyphenyl)-2,2-dimethylpropanal (3a), 2-(4-methoxybenzyl)-3-(4-methoxyphenyl)-2-methylpropanal (3b) and 2,2-Bis(4-methoxybenzyl)-3-(4-methoxyphenyl)propanal (3c)

Prepared according to general procedure J using pivaldehyde (44 µL, 0.40 mmol) and 1-iodo-4-methoxybenzene (244 mg, 1.04 mmol). Purification by flash column chromatography (7.5% diethyl ether/pentane) afforded monoarylated aldehyde **3a** as a colourless oil (19 mg, 25%) followed by diarylated aldehyde **3b** as a pale yellow wax (19 mg, 16%) followed by triarylated aldehyde **3c** as a pale orange oil (19 mg, 12%).

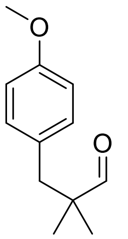

3a

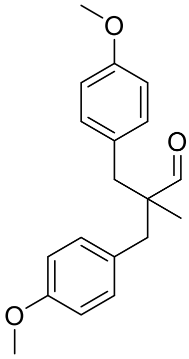

3b

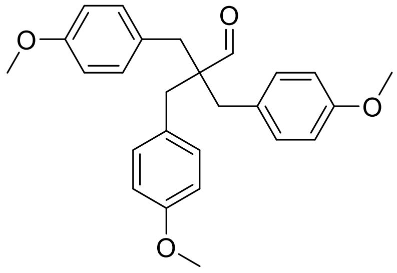

3c

3a: R_f 0.29 (10% diethyl ether/pentane). IR (film)/cm-1 2963, 2932, 2836, 1722 (s, C=O), 1611, 1511 (s), 1465, 1244 (s), 1178, 1034. ^{1}H NMR (400 MHz, $CDCl_3$) δ 9.59 (s, 1H, CHO), 7.04–7.00 (m, 2H, Ar-H), 6.84–6.80 (m, 2H, Ar-H), 3.79 (s, 3H, OCH_3), 2.73 (s, 2H, CH_2), 1.05 (s, 6H, C(CH_3)$_2$). ^{13}C NMR (101 MHz, $CDCl_3$) δ 206.2 (C=O), 158.3 (Ar-C_q), 131.2 (2 × Ar-C), 128.9 (Ar-C_q), 113.6 (2 × Ar-C), 55.2 (OCH_3), 47.0 (*C*(CH_3)$_2$), 42.4 (CH_2), 21.3 (C(*C*H_3)$_2$). Spectroscopic data for this compound (^{1}H NMR, ^{13}C NMR, IR) is consistent with that shown in the literature.[20]

3b: R_f 0.08 (10% diethyl ether/pentane). IR (film)/cm^{-1} 2915, 2836, 1760, 1720 (C=O), 1610, 1509, 1462, 1369, 1244, 1215, 1192, 1177, 1031. ^{1}H NMR (400 MHz, $CDCl_3$) δ 9.67 (s, 1H, CHO), 7.02–7.00 (m, 4H, Ar-H), 6.82–6.80 (m, 4H, Ar-H), 3.79 (s, 6H, OCH_3), 2.97 (d, *J* = 13.8 Hz, 2H, *CH*(H)), 2.64 (d, *J* = 13.8 Hz, 2H, CH(*H*)), 0.97 (s, 3H, CH_3). ^{13}C NMR (101 MHz, $CDCl_3$) δ 206.6 (C=O), 158.3 (2 × Ar-C_q), 131.3 (4 × Ar-C), 128.6 (2 × Ar-C_q), 113.6 (4 × Ar-C), 55.3 (2 × OCH_3), 51.4 (C_q(CH_3)), 41.9 (2 × CH_2), 18.0 (CH_3). HRMS (ESI) m/z Calcd. for $C_{19}H_{26}O_3N$ [M+NH$_4$]$^+$: 316.1907; Found: 316.1910.

3c: R_f 0.05 (10% diethyl ether/pentane). IR (film)/cm^{-1} 2934, 1720 (C=O), 1610, 1582, 1509, 1463, 1440, 1300, 1242 (s), 1176, 1114, 1031. ^{1}H NMR (400 MHz, $CDCl_3$) δ 9.76 (s, 1H, CHO), 7.05–7.02 (m, 6H, Ar-H), 6.84–6.80 (m, 6H, Ar-H), 3.80 (s, 9H, OCH_3), 2.85 (s, 6H, CH_2). ^{13}C NMR (101 MHz, $CDCl_3$) δ 207.4 (C=O), 158.2 (3 × Ar–C_q), 131.5 (6 × Ar-C), 128.6 (3 × Ar-C_q), 113.6 (6 × Ar-C), 55.2 (3 × OCH_3), 53.8 (C_q(CHO)), 39.2 (3 × CH_2). HRMS (ESI) m/z Calcd. for $C_{26}H_{29}O_4$ [M+H]$^+$: 405.2060; Found: 405.2046.

Resubjecting aldehyde 3a and 3b to the reaction conditions

According to general procedure J, 3-(4-methoxyphenyl)-2,2-dimethylpropanal **3a** (19 mg, 0.10 mmol) or 2-(4-methoxybenzyl)-3-(4-methoxyphenyl)-2-methylpropanal **3b** (19 mg, 0.06 mmol) with 1-iodo-4-methoxybenzene (2.6 equiv) were subjected to the arylation conditions. Yields of the arylated aldehyde products were calculated by ^{1}H NMR using *gem*-dimethyl (mono: 1.05 ppm), methyl (di: 0.97 ppm) and methylene (tri: 2.85 ppm) signals in comparison to a known amount of 1,3,5-trimethoxybenzene as an internal standard.

3-(3-Methoxyphenyl)-2,2-dimethylpropanal (4a)

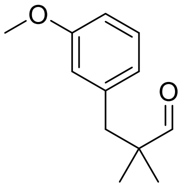

Prepared according to general procedure J using pivaldehyde (44 µL, 0.40 mmol) and 1-iodo-3-methoxybenzene (244 mg, 1.04 mmol). Purification by flash column chromatography (5% diethyl ether/pentane) afforded monoarylated aldehyde **4a** as a colourless oil (15 mg, 20%). R_f 0.31 (10% diethyl ether/pentane). IR (film)/cm^{-1} 2958, 2835, 2710, 1723 (s, C=O), 1600, 1583, 1488, 1465, 1436, 1261 (s), 1154, 1049. ^{1}H NMR (400 MHz, $CDCl_3$) δ 9.60 (s, 1H, CHO), 7.20 (t, *J* = 8.0 Hz, 1H, Ar-H), 6.79–6.76 (m, 1H, Ar-H), 6.69 (d, *J* = 8.0 Hz, 1H, Ar-H), 6.66–6.65 (m, 1H, Ar-H), 3.80 (s, 3H, OCH_3), 2.77 (s, 2H, CH_2), 1.07 (s, 6H, $C(CH_3)_2$). ^{13}C NMR (101 MHz, $CDCl_3$) δ 205.9 (C=O), 159.3 (Ar-C_q), 138.5 (Ar-C_q), 129.1 (Ar-C), 122.7 (Ar-C), 116.2 (Ar-C), 111.6 (Ar-C), 55.1 (OCH_3), 46.9 ($\mathit{C}(CH_3)_2$), 43.2 (CH_2), 21.5 ($C(\mathit{C}H_3)_2$). HRMS (ASAP(SOLID)) m/z Calcd. for $C_{12}H_{17}O_2$ $[M+H]^+$: 193.1229; Found: 193.1225.

3-(2-Methoxyphenyl)-2,2-dimethylpropanal (5a)

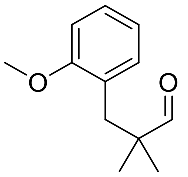

Prepared according to general procedure J using pivaldehyde (44 µL, 0.40 mmol) and 1-iodo-2-methoxybenzene (244 mg, 1.04 mmol). Purification by flash column chromatography (5% diethyl ether/pentane) afforded monoarylated aldehyde **5a** as a colourless oil (7 mg, 9%). R_f 0.21 (10% diethyl ether/pentane). IR (film)/cm^{-1} 2962, 1722 (C=O), 1493, 1463, 1243, 1178, 1028. ^{1}H NMR (400 MHz, $CDCl_3$) δ 9.53 (s, 1H, CHO), 7.21 (ddd, *J* = 8.1, 7.5, 1.7 Hz, 1H, Ar-H), 7.06 (dd, *J* = 7.5, 1.7 Hz, 1H, Ar-H), 6.88 (td, *J* = 7.5, 1.0 Hz, 1H, Ar-H), 6.83 (dd, *J* = 8.1, 1.0 Hz, 1H, Ar-H), 3.77 (s, 3H, OCH_3), 2.82 (s, 2H, CH_2), 1.04 (s, 6H, $C(CH_3)_2$). ^{13}C NMR (101 MHz, $CDCl_3$) δ 205.7 (C=O), 157.4 (Ar-C_q), 132.1 (Ar-C), 128.0 (Ar-C), 125.7 (Ar-C_q), 120.2 (Ar-C), 110.4 (Ar-C), 54.9 (OCH_3), 46.9 ($\mathit{C}(CH_3)_2$), 38.0 (CH_2), 21.6 ($C(\mathit{C}H_3)_2$). HRMS (ASAP(SOLID)) m/z Calcd. for $C_{12}H_{17}O_2$ $[M+H]^+$: 193.1229; Found: 193.1226.

3-(Benzo[d][1,3]dioxol-5-yl)-2,2-dimethylpropanal (6a)

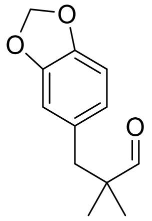

Prepared according to general procedure J using pivaldehyde (44 µL, 0.40 mmol) and 5-iodobenzo[d][1,3]dioxole (153 µL, 1.04 mmol). Purification by flash column chromatography (7.5% diethyl ether/pentane) afforded monoarylated aldehyde **6a** as a pale yellow oil (9 mg, 11%). R_f 0.29 (10% diethyl ether/pentane). IR (film)/cm^{-1} 2965, 2704, 1721 (C=O), 1489, 1440, 1360, 1240, 1190, 1036. ^{1}H NMR (400 MHz, $CDCl_3$) δ 9.57 (s, 1H, CHO), 6.72 (d, *J* = 7.9 Hz, 1H, Ar-H), 6.59 (d, *J* = 1.7 Hz, 1H, Ar-H), 6.55 (dd, *J* = 7.9, 1.7 Hz, 1H, Ar-H), 5.93 (s, 2H, OCH_2O), 2.71 (s, 2H, CH_2), 1.05 (s, 6H, $C(CH_3)_2$). ^{13}C NMR (101 MHz, $CDCl_3$) δ 206.0 (C=O), 147.4 (Ar-C_q), 146.2 (Ar-C_q), 130.6 (Ar-C_q), 123.2 (Ar-C), 110.5 (Ar-C), 108.0 (Ar-C), 100.9 (OCH_2O), 47.0 ($\mathit{C}(CH_3)_2$), 42.9 (CH_2), 21.4 ($C(\mathit{C}H_3)_2$). HRMS (ASAP(DCM)) m/z Calcd. for $C_{12}H_{13}O_3$ $[M-H]^+$: 205.0865; Found: 205.0859.

2,2-Dimethyl-3-(4-nitrophenyl)propanal (7a) and 2-methyl-2-(4-nitrobenzyl)-3-(4-nitrophenyl)propanal (7b)

Prepared according to general procedure J using pivaldehyde (44 µL, 0.40 mmol) and 1-iodo-4-nitrobenzene (259 mg, 1.04 mmol). Purification by flash column chromatography (10%–50% diethyl ether/pentane) afforded monoarylated aldehyde **7a** as an orange oil (24 mg, 29%) followed by diarylated aldehyde **7b** as a pale yellow amorphous solid (21 mg, 16%).

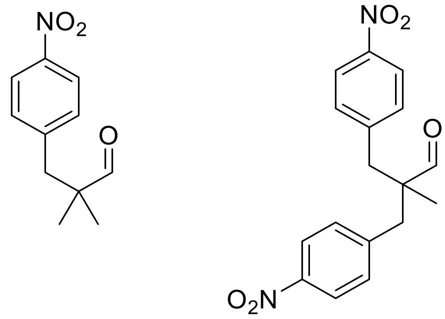

7a **7b**

7a: R_f 0.16 (10% diethyl ether/pentane). IR (film)/cm^{-1} 2967, 1722 (C=O), 1597, 1514, 1341, 1109. ^{1}H NMR (400 MHz, $CDCl_3$) δ 9.58 (s, 1H, CHO), 8.17–8.14 (m, 2H, Ar-H), 7.32–7.28 (m, 2H, Ar-H), 2.92 (s, 2H, CH_2), 1.10 (s, 6H, $C(CH_3)_2$). ^{13}C NMR (101 MHz, $CDCl_3$) δ 204.7 (C=O), 146.8 (Ar-C_q), 145.0 (Ar-C_q), 131.1 (2 × Ar-C), 123.3 (2 × Ar-C), 46.9 (*C*$(CH_3)_2$), 42.4 (CH_2), 21.5 (C(*C*$H_3)_2$). HRMS (ASAP(DCM)) m/z Calcd. for $C_{11}H_{14}NO_3$ [M+H]$^+$: 208.0974; Found: 208.0973.

7b: R_f 0.08 (30% diethyl ether/pentane). IR (film)/cm^{-1}2970, 1723 (C=O), 1598, 1515, 1339, 1107, 1089. ^{1}H NMR (400 MHz, $CDCl_3$) δ 9.65 (s, 1H, CHO), 8.19–8.15 (m, 4H, Ar-H), 7.31–7.27 (m, 4H, Ar-H), 3.16 (d, *J* = 13.5 Hz, 2H, 2 × C(H)*H*), 2.84 (d, *J* = 13.5 Hz, 2H, 2 × C(*H*)H), 1.08 (s, 3H, CH_3).^{13}C NMR (101 MHz, $CDCl_3$) δ 204.0 (C=O), 147.0 (2 × Ar-C_q), 143.7 (2 × Ar-C_q), 131.2 (4 × Ar-C), 123.5 (4 × Ar-C), 51.1 (*C*(CH_3)), 42.2 (2 × CH_2), 18.5 (CH_3). HRMS (ASAP(SOLID)) m/z Calcd. for $C_{17}H_{17}N_2O_5$ [M+H]$^+$: 329.1137; Found: 329.1138.

3-(4-Chlorophenyl)-2,2-dimethylpropanal (8a), 2-(4-chlorobenzyl)-3-(4-chlorophenyl)-2-methylpropanal (8b) and 2,2-bis(4-chlorobenzyl)-3-(4-chlorophenyl)propanal (8c)

Prepared according to general procedure J using pivaldehyde (44 µL, 0.40 mmol) and 1-chloro-4-iodobenzene (248 mg, 1.04 mmol). Purification by flash column chromatography (2.5% diethyl ether/pentane) afforded monoarylated aldehyde **8a** as a colourless oil (24 mg, 31%) followed by a 5.2:1 mixture of diarylated aldehyde **8b** (29 mg, 24%) and triarylated aldehyde **8c** (7 mg, 4%) as an off-white solid.

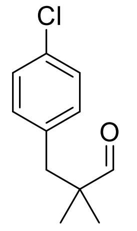

8a

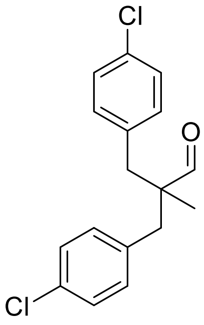

8b

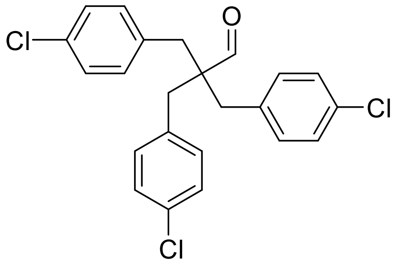

8c

8a: R_f 0.32 (10% diethyl ether/pentane). IR (film)/cm^{-1} 2966, 2929, 2704, 1723 (s, C=O), 1491, 1467, 1198, 1090, 1015. ^{1}H NMR (400 MHz, $CDCl_3$) δ 9.57 (s, 1H, CHO), 7.27–7.23 (m, 2H, Ar-H), 7.05–7.01 (m, 2H, Ar-H), 2.76 (s, 2H, CH_2), 1.05 (s, 6H, $C(CH_3)_2$). ^{13}C NMR (101 MHz, $CDCl_3$) δ 205.6 (CHO), 135.4 (Ar-C_q), 132.5 (Ar-C_q), 131.5 (2 × Ar-C), 128.3 (2 × Ar-C), 46.8 ($\mathit{C}(CH_3)_2$), 42.3 (CH_2), 21.4 ($C(\mathit{C}H_3)_2$). HRMS (ASAP(DCM)) m/z Calcd. for $C_{11}H_{13}O_1Cl_1$ $[M+CH_3]^+$: 196.0655; Found: 196.0651.

8b: R_f 0.20 (10% diethyl ether/pentane). IR (film)/cm^{-1} 2921, 1724 (s, C=O) 1491 (s), 1409, 1093, 1015. ^{1}H NMR (400 MHz, $CDCl_3$) δ 9.63 (s, 1H , CHO), 7.27–7.23 (m, 4H, Ar-H), 7.02–7.00 (m, 4H, Ar-H), 2.98 (d, *J* = 13.7 Hz, 2H, 2 × C(*H*)H), 2.66 (d, *J* = 13.7 Hz, 2H, 2 × C(H)*H*), 0.98 (s, 3H, CH_3). ^{13}C NMR (101 MHz, $CDCl_3$) δ 205.5 (CHO), 134.8 (2 × Ar-C_q), 132.7 (2 × Ar-C_q), 131.6 (4 × Ar-C), 128.4 (4 × Ar-C), 51.0 ($\mathit{C}(CH_3)$), 41.9 (2 × CH_2), 18.2 (CH_3). HRMS (ASAP(DCM)) m/z Calcd. for $C_{17}H_{17}OCl_2$ $[M+H]^+$: 307.0656; Found: 307.0651.

8c: R_f 0.20 (10% diethyl ether/pentane). IR (film)/cm^{-1} 2921, 1724 (s, C=O) 1491 (s), 1409, 1093, 1015. ^{1}H NMR (400 MHz, $CDCl_3$) δ 9.73 (s, 1H, CHO), 7.27–7.23 (m, 6H, Ar-H), 7.02–7.00 (m, 6H, Ar-H), 2.86 (s, 6H, 3 × CH_2). ^{13}C NMR (101 MHz, $CDCl_3$) δ 205.4 (CHO), 134.5 (3 × Ar-C_q), 132.8 (3 × Ar-C_q), 131.8 (6 × Ar-C), 128.5 (6 × Ar-C), 53.5 (C(CHO)), 39.6 (3 × CH_2). HRMS (ASAP(DCM)) m/z Calcd. for $C_{23}H_{20}OCl_3$ $[M+H]^+$: 417.0580; Found: 417.0577.

3-(4-Fluorophenyl)-2,2-dimethylpropanal (9a), 2-(4-fluorobenzyl)-3-(4-fluorophenyl)-2-methylpropanal (9b) and 2,2-bis(4-fluorobenzyl)-3-(4-fluorophenyl)propanal (9c)

Prepared according to general procedure J using pivaldehyde (44 µL, 0.40 mmol) and 1-fluoro-4-iodobenzene (124 µL, 1.04 mmol). Purification by flash column chromatography (2.5% diethyl ether/pentane) afforded monoarylated aldehyde **9a** as a colourless oil (19 mg, 26%) followed by a 4.5:1 mixture of diarylated aldehyde **9b** (26 mg, 24%) and triarylated aldehyde **9c** (8 mg, 5%) as a pale yellow oil.

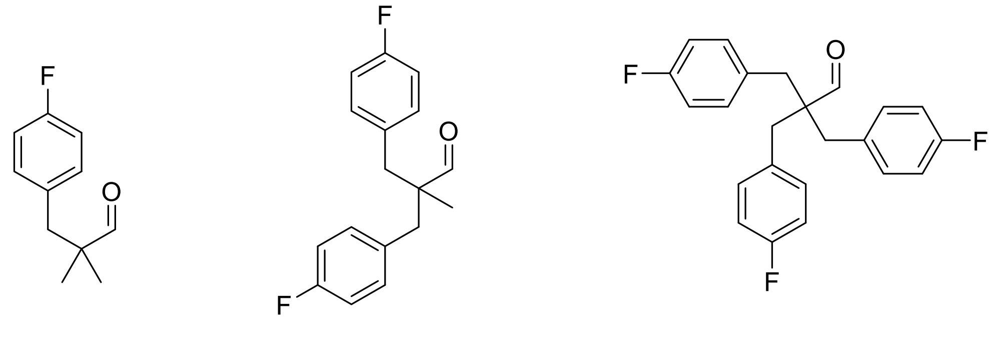

9a **9b** **9c**

9a: R_f 0.32 (10% diethyl ether/pentane). IR (film)/cm^{-1} 2970, 2701, 1723 (C=O), 1605, 1508 (s), 1468, 1221, 1159. ^{1}H NMR (400 MHz, $CDCl_3$) δ 9.58 (s, 1H, CHO), 7.08–7.04 (m, 2H, Ar-H), 6.99–6.94 (m, 2H, Ar-H), 2.76 (s, 2H, CH_2), 1.05 (s, 6H, $C(CH_3)_2$). ^{13}C NMR (101 MHz, $CDCl_3$) δ 205.7 (C=O), 161.7 (d, $^1J_{C-F}$ = 244.8 Hz, Ar-C_q), 132.6 (d, $^4J_{C-F}$ = 3.1 Hz, Ar-C_q), 131.6 (d, $^3J_{C-F}$ = 7.9 Hz, 2 × Ar-C), 115.0 (d, $^2J_{C-F}$ = 21.1 Hz, 2 × Ar-C), 46.9 (*C*(CH_3)$_2$), 42.2 (CH_2), 21.3 (C(*C*H_3)$_2$). ^{19}F NMR (377 MHz, $CDCl_3$) δ –116.6.

9b: R_f 0.20 (10% diethyl ether/pentane). IR (film)/cm^{-1} 2922, 1722 (C=O), 1603, 1507, 1219, 1158. ^{1}H NMR (400 MHz, $CDCl_3$) δ 9.65 (s, 1H, CHO), 7.08–7.03 (m, 4H, Ar-H), 7.00–6.95 (m, 4H, Ar-H), 2.99 (d, *J* = 13.8 Hz, 2H, 2 × C(H)*H*), 2.67 (d, *J* = 13.8 Hz, 2H, 2 × C(H)*H*), 0.98 (s, 3H, CH_3). ^{13}C NMR (101 MHz, $CDCl_3$) δ 205.8 (C=O), 161.8 (d, $^1J_{C-F}$ = 245.2 Hz, 2 x Ar-C_q), 132.1 (d, $^4J_{C-F}$ = 3.2 Hz, 2 × Ar-C_q), 131.7 (d, $^3J_{C-F}$ = 7.9 Hz, 4 × Ar-C), 115.1 (d, $^2J_{C-F}$ = 21.3 Hz, 4 × Ar-C), 51.1 (*C*(CH_3)), 41.8 (2 × CH_2), 18.1 (CH_3). ^{19}F NMR (377 MHz, $CDCl_3$) δ –116.2. HRMS (ASAP(SOLID)) m/z Calcd. for $C_{17}H_{17}OF_2$ [M+H]$^+$: 275.1247; Found: 275.1246.

9c: R_f 0.20 (10% diethyl ether/pentane). IR (film)/cm^{-1} 2922, 1722 (C=O), 1603, 1507, 1219, 1158. ^{1}H NMR (400 MHz, $CDCl_3$) δ 9.75 (s, 1H, CHO), 7.08–7.03 (m, 6H, Ar-H), 7.00–6.95 (m, 6H, Ar-H), 2.87 (s, 6H, 3 × CH_2). ^{13}C NMR (101 MHz, $CDCl_3$) δ 205.9 (C=O), 161.8 (d, $^1J_{C-F}$ = 245.2 Hz, 3 × Ar-C_q), 132.1 (d, $^4J_{C-F}$ = 3.2 Hz, 3 × Ar-C_q), 131.9 (d, $^3J_{C-F}$ = 7.8 Hz, 6 × Ar-C), 115.2 (d, $^2J_{C-F}$ = 21.0 Hz, 6 × Ar-C), 53.5 (*C*(CHO)), 39.4 (3 × CH_2). ^{19}F NMR (377 MHz, $CDCl_3$) δ -116.0. HRMS (ASAP(SOLID)) m/z Calcd. for $C_{23}H_{20}OF_3$ [M+H]$^+$: 369.1466; Found: 369.1464.

3-(2-Fluorophenyl)-2,2-dimethylpropanal (10a), 2-(2-fluorobenzyl)-3-(2-fluorophenyl)-2-methylpropanal (10b) and 2,2-bis(2-fluorobenzyl)-3-(2-fluorophenyl)propanal (10c)

Prepared according to general procedure J using pivaldehyde (44 µL, 0.40 mmol) and 1-fluoro-2-iodobenzene (121 µL, 1.04 mmol). Purification by flash column chromatography (2.5% diethyl ether/pentane) afforded monoarylated aldehyde **10a** as a colourless oil (10 mg, 12%) followed by a 8.2:1 mixture of diarylated aldehyde **10b** (6 mg, 5%) and triarylated aldehyde **10c** (1 mg, 1%) as an off-white solid.

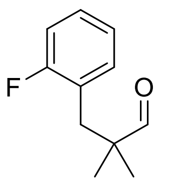

10a

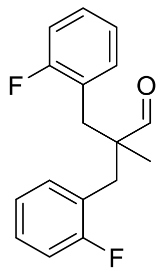

10b

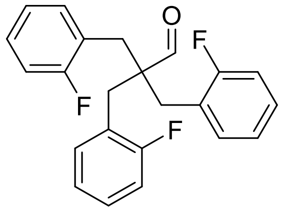

10c

10a: R_f 0.35 (10% diethyl ether/pentane). IR (film)/cm^{-1} 2970, 1725 (C=O), 1492, 1453, 1229, 1183. ^{1}H NMR (400 MHz, $CDCl_3$) δ 9.60 (d, $J_{H\text{-}F}$ = 1.7 Hz, 1H, CHO), 7.25–7.19 (m, 1H, Ar-H), 7.13–7.00 (m, 3H, Ar-H), 2.84 (d, $J_{H\text{-}F}$ = 1.6 Hz, 2H, CH_2), 1.08 (d, $J_{H\text{-}F}$ = 0.6 Hz, 6H, $C(CH_3)_2$). ^{13}C NMR (101 MHz, $CDCl_3$) δ 205.4 (C=O), 161.3 (d, $^1J_{C\text{-}F}$ = 244.8 Hz, Ar-C_q), 132.6 (d, $^3J_{C\text{-}F}$ = 4.4 Hz, Ar-C), 128.5 (d, $^3J_{C\text{-}F}$ = 8.3 Hz, Ar-C), 124.0 (d, $^2J_{C\text{-}F}$ = 15.6 Hz, Ar-C_q), 123.8 (d, $^4J_{C\text{-}F}$ = 3.4 Hz, Ar-C), 115.4 (d, $^2J_{C\text{-}F}$ = 23.1 Hz, Ar-C), 47.1 ($\mathit{C}(CH_3)_2$), 35.7 (CH_2), 21.2 ($C(\mathit{C}H_3)_2$). ^{19}F NMR (377 MHz, $CDCl_3$) δ –115.3. HRMS (EI) m/z Calcd. for $C_{11}H_{13}OF$ $[M+H]^+$: 180.0950; Found: 180.0957.

10b: R_f 0.27 (10% diethyl ether/pentane). IR (film)/cm^{-1} 2927, 1724 (C=O), 1584, 1491, 1455, 1228, 1182. ^{1}H NMR (400 MHz, $CDCl_3$) δ 9.69 (t, $J_{H\text{-}F}$ = 2.1 Hz, 1H, CHO), 7.25–7.20 (m, 2H, Ar-H), 7.13–7.01 (m, 6H, Ar-H), 3.08 (dd, *J* = 13.8, $1.2_{(H\text{-}F)}$ Hz, 2H, 2 × C(H)*H*), 2.84 (dd, *J* = 13.8, $1.5_{(H\text{-}F)}$ Hz, 2H, 2 × C(*H*)H), 0.99 (t, $J_{H\text{-}F}$ = 1.1 Hz, 3H, CH_3). ^{13}C NMR (101 MHz, $CDCl_3$) δ 204.9 (C=O), 161.2 (d, $^1J_{C\text{-}F}$ = 245.0 Hz, 2 × Ar-C_q), 132.6 (d, $^3J_{C\text{-}F}$ = 4.2 Hz, 2 × Ar-C), 128.7 (d, $^3J_{C\text{-}F}$ = 8.3 Hz, 2 × Ar-C), 123.9 (d, $^4J_{C\text{-}F}$ = 3.3 Hz, 2 × Ar-C), 123.6 (d, $^2J_{C\text{-}F}$ = 15.9 Hz, 2 × Ar-C_q), 115.5 (d, $^2J_{C\text{-}F}$ = 22.7 Hz, 2 × Ar-C), 51.6 ($\mathit{C}(CH_3)$), 35.4 (2 × CH_2), 17.3 (CH_3). ^{19}F NMR (377 MHz, $CDCl_3$) δ –114.9. HRMS (ASAP(SOLID)) m/z Calcd. for $C_{17}H_{17}OF_2$ $[M+H]^+$: 275.1247; Found: 275.1251.

10c: R_f 0.27 (10% diethyl ether/pentane). IR (film)/cm^{-1} 2927, 1724 (C=O), 1584, 1491, 1455, 1228, 1182. ^{1}H NMR (400 MHz, $CDCl_3$) δ 9.65 (s, 1H, CHO), 7.25–7.20 (m, 3H, Ar-H), 7.13–7.01 (m, 9H, Ar-H), 2.99 (s, 6H, 3 × CH_2). ^{19}F NMR (377 MHz, $CDCl_3$) δ –114.1. HRMS (ASAP(SOLID)) m/z Calcd. for $C_{23}H_{20}OF_3$ $[M+H]^+$: 369.1466; Found: 369.1463.

3-(4-Bromophenyl)-2,2-dimethylpropanal (11a), 2-(4-bromobenzyl)-3-(4-bromophenyl)-2-methylpropanal (11b) and 2,2-bis(4-bromobenzyl)-3-(4-bromophenyl)propanal (11c)

Prepared according to general procedure J using pivaldehyde (44 µL, 0.40 mmol) and 1-bromo-4-iodobenzene (294 mg, 1.04 mmol). Purification by flash column chromatography (2.5% diethyl ether/pentane) afforded monoarylated aldehyde **11a** as a colourless oil (29 mg, 30%) followed by a 5:1 mixture of diarylated aldehyde **11b** (36 mg, 23%) and triarylated aldehyde **11c** (10 mg, 5%) as a colourless oil.

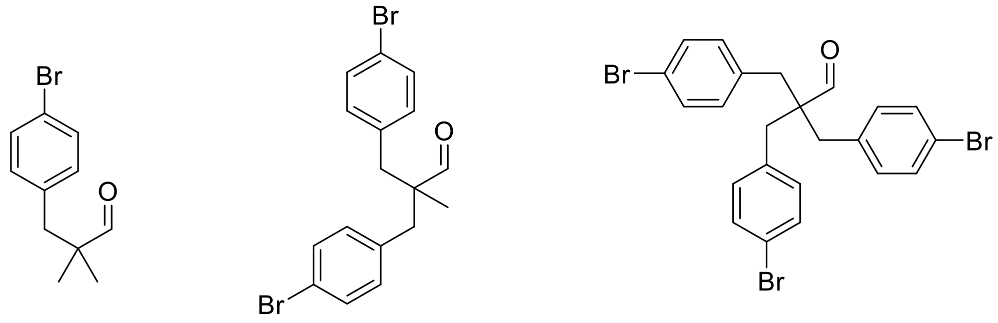

11a **11b** **11c**

11a: R_f 0.31 (10% diethyl ether/pentane). IR (film)/cm^{-1} 2967, 2702, 1722 (s, C=O), 1487, 1467, 1404, 1197. ^{1}H NMR (400 MHz, $CDCl_3$) δ 9.56 (s, 1H, CHO), 7.42–7.38 (m, 2H, Ar-H), 7.00–6.96 (m, 2H, Ar-H), 2.74 (s, 2H, CH_2), 1.05 (s, *J* = 5.0 Hz, 6H, $C(CH_3)_2$). ^{13}C NMR (101 MHz, $CDCl_3$) δ 205.5 (C=O), 135.9 (Ar-C_q), 131.9 (2 × Ar-C), 131.2 (2 × Ar-C), 120.5 (Ar-C_q), 46.8 ($\mathit{C}(CH_3)_2$), 42.4 (CH_2), 21.4 ($C(\mathit{C}H_3)_2$). Spectroscopic data for this compound (^{1}H NMR, ^{13}C NMR, IR) is consistent with that shown in the literature.[21]

11b: R_f 0.19 (10% diethyl ether/pentane). IR (film)/cm^{-1} 2971, 2919, 1722 (C=O), 1487, 1405, 1072, 1010. ^{1}H NMR (400 MHz, $CDCl_3$) δ 9.62 (s, 1H, CHO), 7.41–7.39 (m, 4H, Ar-H), 6.97–6.95 (m, 4H, Ar-H), 2.96 (d, *J* = 13.7 Hz, 2H, 2 × C(H)*H*), 2.65 (d, *J* = 13.7 Hz, 2H, 2 × C(*H*)H), 0.98 (s, 3H, CH_3). ^{13}C NMR (101 MHz, $CDCl_3$) δ 205.4 (C=O), 135.3 (2 × Ar-C_q), 132.0 (4 × Ar-C), 131.4 (4 × Ar-C), 120.8 (2 × Ar-C_q), 50.9 ($\mathit{C}(CH_3)$), 41.9 (2 × CH_2), 18.2 (CH_3). HRMS (ASAP(SOLID)) m/z Calcd. for $C_{18}H_{19}OBr_2$ $[M+CH_3]^+$: 408.9803; Found: 408.9805.

11c: R_f 0.19 (10% diethyl ether/pentane). IR (film)/cm^{-1} 2971, 2919, 1722 (C=O), 1487, 1405, 1072, 1010. ^{1}H NMR (400 MHz, $CDCl_3$) δ 9.72 (s, 1H, CHO), 7.42–7.39 (m, 6H, Ar-H), 6.97–6.95 (m, 6H, Ar-H), 2.84 (s, 6H, 3 × CH_2). ^{13}C NMR (101 MHz, $CDCl_3$) δ 205.3 (C=O), 135.0 (3 × Ar-C_q), 132.12 (6 × Ar-C), 131.5 (6 × Ar-C), 120.9 (3 × Ar-C_q), 53.3 (*C*(CHO)), 39.6 (3 × CH_2). HRMS (ASAP(SOLID)) m/z Calcd. for $C_{23}H_{20}OBr_3$ $[M+CH_3]^+$: 548.9064; Found: 548.9066.

5-Iodo-1-tosyl-1H-indole (S23)

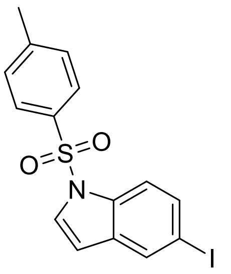

Tosyl chloride (836 mg, 4.4 mmol) in DMF (2.2 mL) was added dropwise to a stirred suspension of 5-iodo-1-tosyl-1H-indole (936 mg, 4.0 mmol) and sodium hydride (192 mg, 4.8 mmol, 60% dispersion in mineral oil) in DMF (10 mL) at 0 °C. The reaction was stirred at 0 °C for 30 minutes then allowed to warm to rt and stirred for 2 h. The reaction was quenched by addition of water and the product extracted with EtOAc. The combined organic extracts were washed with saturated sodium bicarbonate solution, dried ($MgSO_4$) and solvent removed under reduced pressure. Purification by flash column chromatography (SiO_2, 0-20% diethyl ether/hexane) afforded tosyl indole **S23** (1.38 g, 87%) as a white solid. R_f 0.17 (10% diethyl ether/hexane). mp = 132–137 °C (lit = 136–138 °C)[22]. IR (film)/cm^{-1} 1595, 1437, 1361, 1252, 1193, 1168, 1130, 1092. ^{1}H NMR (400 MHz, $CDCl_3$) δ 7.87 (s, 1H, Ar-H), 7.77–7.73 (m, 3H Ar-H), 7.58 (d, J = 8.7 Hz, 1H, Ar-H), 7.53 (d, J = 3.6 Hz, 1H, Ar-H), 7.24 (d, J = 8.3 Hz, 2H, Ar-H), 6.58 (d, J = 3.6 Hz, 1H, Ar-H), 2.36 (s, 3H, Ar-CH_3). ^{13}C NMR (101 MHz, $CDCl_3$) δ 145.2 (Ar-C_q), 135.0 (Ar-C_q) 134.1 (Ar-C_q), 133.02 (Ar-C), 133.00 (Ar-C_q) 130.2 (Ar-C), 130.0 (2 × Ar-C), 127.2 (Ar-C), 126.8 (2 × Ar-C), 115.3 (Ar-C), 108.0 (Ar-C), 87.4 (Ar-C_q), 21.6 (Ar-CH_3). Spectroscopic data for this compound (^{1}H NMR, ^{13}C NMR, IR) is consistent with that shown in the literature.[23]

2,2-Dimethyl-3-(1-tosyl-1H-indol-5-yl)propanal (12a)

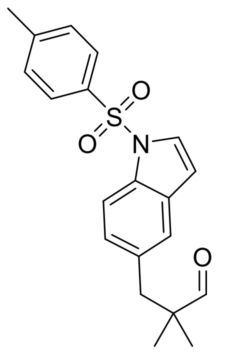

Prepared according to general procedure J using pivaldehyde (44 µL, 0.40 mmol) and 5-iodo-1-tosyl-1H-indole **S23** (294 mg, 1.04 mmol). Purification by flash column chromatography (15% diethyl ether/hexane) afforded monoarylated aldehyde **12a** as an amourphous off white solid (26 mg, 18%). R_f 0.41 (40% diethyl ether/hexane). IR (film)/cm^{-1}2966, 1722 (C=O), 1459, 1370, 1173, 1129, 1092. ^{1}H NMR (400 MHz, $CDCl_3$) δ 9.59 (s, 1H, CHO), 7.87 (d, J = 8.6 Hz, 1H, Ar-H), 7.77 (d, J = 8.6 Hz, 2H, Ar-H), 7.54 (d, J = 3.6 Hz, 1H, Ar-H), 7.24 (d, J = 8.3 Hz, 3H, Ar-H), 7.04 (dd, J = 8.6, 1.6 Hz, 1H, Ar-H), 6.59 (dt, J = 5.3, 2.7 Hz, 1H, Ar-H), 2.84 (s, 2H, CH_2), 2.35 (s, 3H, Ar-CH_3), 1.05 (s, 6H, $C(CH_3)_2$). ^{13}C NMR (101 MHz, $CDCl_3$) δ 206.1 (CHO), 144.9 (Ar-C_q), 135.3 (Ar-C_q), 133.6 (Ar-C_q), 131.9 (Ar-C_q), 130.8 (Ar-C_q), 129.9 (2 × Ar-C), 126.9 (Ar-C), 126.8 (2 × Ar-C), 126.5 (Ar-C), 122.7 (Ar-C), 113.0 (Ar-C), 108.7 (Ar-C), 47.1 ($\mathit{C}(CH_3)_2$), 43.0 (CH_2), 21.6 (Ar-CH_3), 21.4 ($C(\mathit{C}H_3)_2$). HRMS (ESI) m/z Calcd. for $C_{20}H_{20}NO_3S$ $[M-H]^-$: 354.1164; Found: 354.1171.

2-(4-Methoxybenzyl)-2-methyloctanal (13a) and 2,2-bis(4-methoxybenzyl)octanal (13b)

Prepared according to general procedure J using 2,2-dimethyloctanal **S7** (63 mg, 0.40 mmol) and 1-iodo-4-methoxybenzene (244 mg, 1.04 mmol). Purification by flash column chromatography (2.5–5% diethyl ether/pentane) afforded monoarylated aldehyde **13a** as a colourless oil (33 mg, 31%) followed by diarylated aldehyde **13b** as a pale yellow oil (33 mg, 22%).

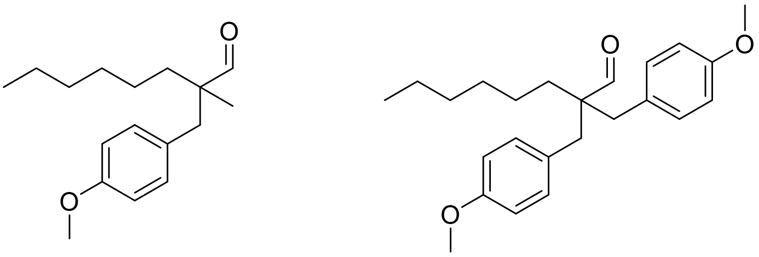

13a **13b**

13a: R_f 0.34 (10% diethyl ether/pentane). IR (film)/cm^{-1} 2929, 2856, 1725 (C=O), 1611, 1511, 1463, 1372, 1246, 1178. ^{1}H NMR (400 MHz, $CDCl_3$) δ 9.56 (s, 1H, CHO), 7.02–6.98 (m, 2H, Ar-H), 6.83–6.79 (m, 2H, Ar-H), 3.79 (s, 3H, OCH_3), 2.82 (d, *J* = 13.8 Hz, 1H, C(H)*H*), 2.66 (d, *J* = 13.8 Hz, 1H, C(*H*)H), 1.61–1.54 (m, 1H, CH_2C(H)*H*), 1.44–1.38 (m, 1H, CH_2C(*H*)H), 1.32–1.17 (m, 8H, 4 × CH_2), 0.99 (s, 3H, CH_3), 0.89 (t, *J* = 6.8 Hz, 3H, CH_3). ^{13}C NMR (101 MHz, $CDCl_3$) δ 206.7 (C=O), 158.2 (Ar-C_q), 131.1 (2 × Ar-C), 128.9 (Ar-C_q), 113.5 (2 × Ar-C), 55.2 (OCH_3), 50.4 (*C*(CH_3)), 41.1 (CH_2), 35.6 (CH_2), 31.6 (CH_2), 29.9 (CH_2), 24.1 (CH_2), 22.5 (CH_2), 18.2 (CH_3), 14.0 (CH_3). HRMS (ASAP(SOLID)) m/z Calcd. For $C_{17}H_{27}O_2$ [M+H]$^+$: 263.2011; Found: 263.2005.

13b: R_f 0.14 (10% diethyl ether/pentane). IR (film)/cm^{-1} 2930, 2855, 1722 (C=O), 1611, 1510, 1463, 1245, 1177, 1034. ^{1}H NMR (400 MHz, $CDCl_3$) δ 9.62 (s, 1H, CHO), 7.03–6.99 (m, 4H, Ar-H), 6.83–6.80 (m, 4H, Ar-H), 3.80 (s, 6H, 2 × OCH_3), 2.89 (d, *J* = 14.2 Hz, 2H, 2 × C(H)*H*), 2.75 (d, *J* = 14.2 Hz, 2H, 2 × C(*H*)H), 1.47– 1.35 (m, 4H, 2 × CH_2), 1.33–1.24 (m, 6H, 3 × CH_2), 0.89 (t, *J* = 6.8 Hz, 3H, CH_3). ^{13}C NMR (101 MHz, $CDCl_3$) δ 207.6 (C=O), 158.2 (2 × Ar-C_q), 131.2 (4 × Ar-C), 128.7 (2 × Ar-C_q), 113.6 (4 × Ar-C), 55.2 (2 × OCH_3), 54.0 (*C*(CHO)), 38.8 (2 × CH_2), 31.7 (CH_2), 31.0 (CH_2), 29.8 (CH_2), 23.7 (CH_2), 22.6 (CH_2), 14.0 (CH_3). HRMS (ASAP(SOLID)) m/z Calcd. For $C_{24}H_{33}O_3$ [M+H]$^+$: 369.2430; Found: 369.2422.

2-(4-Methoxybenzyl)-2-methyldecanal (14a) and 2,2-bis(4-methoxybenzyl)decanal (14b)

Prepared according to general procedure J using 2,2-dimethyldecanal **S10** (74 mg, 0.40 mmol) and 1-iodo-4-methoxybenzene (244 mg, 1.04 mmol). Purification by flash column chromatography (2.5–5% diethyl ether/pentane) afforded monoarylated aldehyde **14a** as a colourless oil (35 mg, 30%) followed by diarylated aldehyde **14b** as a pale yellow oil (38 mg, 24%).

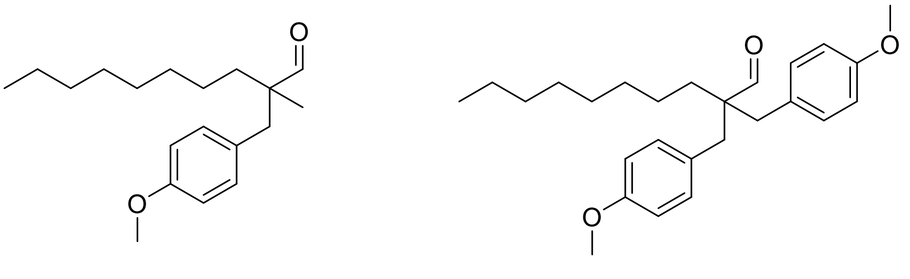

14a **14b**

14a: R_f 0.38 (10% diethyl ether/pentane). IR (film)/cm^{-1} 2925, 2853, 1723 (C=O), 1611, 1512, 1463, 1246, 1036. ^{1}H NMR (400 MHz, $CDCl_3$) δ 9.56 (s, 1H, CHO), 7.02–6.98 (m, 2H, Ar-H), 6.83–6.79 (m, 2H, Ar-H), 3.79 (s, 3H, OCH_3), 2.82 (d, *J* = 13.8 Hz, 1H, C(H)*H*), 2.66 (d, *J* = 13.8 Hz, 1H, C(*H*)H), 1.56–1.53 (m, 1H, CH_2C(H)*H*), 1.44–1.37 (m, 1H CH_2C(*H*)H) 1.32–1.19 (m, 12H, 6 × CH_2), 0.99 (s, 3H, CH_3), 0.89 (t, *J* = 6.9 Hz, 3H, CH_3). ^{13}C NMR (101 MHz, $CDCl_3$) δ 206.7 (C=O), 158.2 (Ar-C_q), 131.1 (2 × Ar-C), 128.9 (Ar-C_q), 113.5 (2 × Ar-C), 55.2 (OCH_3), 50.4 (*C*(CH_3)), 41.1 (CH_2), 35.6 (CH_2), 31.8 (CH_2), 30.2 (CH_2), 29.4 (CH_2), 29.2 (CH_2), 24.2 (CH_2), 22.6 (CH_2), 18.2 (CH_3), 14.1 (CH_3). HRMS (ASAP(SOLID)) m/z Calcd. For $C_{19}H_{31}O_2$ [M+H]$^+$: 291.2324; Found: 291.2317.

14b: R_f 0.19 (10% diethyl ether/pentane). IR (film)/cm^{-1} 2926, 2853, 1721 (C=O), 1611, 1510, 1246, 1177, 1034. ^{1}H NMR (400 MHz, $CDCl_3$) δ 9.62 (s, 1H, CHO), 7.02–7.00 (m, 4H, Ar-H), 6.82–6.80 (m, 4H, Ar-H), 3.80 (s, 6H, 2 × OCH_3), 2.89 (d, *J* = 14.2 Hz, 2H, 2 × C(H)*H*), 2.74 (d, *J* = 14.2 Hz, 2H, 2 × C(*H*)H), 1.46–1.37 (m, 4H, 2 × CH_2) 1.33–1.27 (m, 10H, 5 × CH_2), 0.89 (t, *J* = 6.9 Hz, 3H, CH_3). ^{13}C NMR (101 MHz, $CDCl_3$) δ 207.7 (C=O), 158.2 (2 × Ar-C_q), 131.2 (4 × Ar-C), 128.7 (2 × Ar-C_q), 113.6 (4 × Ar-C), 55.2 (2 × OCH_3), 54.0 (*C*(CHO)), 38.8 (2 × CH_2), 31.8 (CH_2), 31.0 (CH_2), 30.1 (CH_2), 29.4 (CH_2), 29.2 (CH_2), 23.7 (CH_2), 22.6 (CH_2), 14.1 (CH_3). HRMS (ASAP(SOLID)) m/z Calcd. For $C_{26}H_{37}O_3$ [M+H]$^+$: 397.2743; Found: 397.2735.

2-(4-Methoxybenzyl)-2-methyldodecanal (15a) and 2,2-bis(4-methoxybenzyl)dodecanal (15b)

Prepared according to general procedure J using 2,2-dimethyldodecanal **S13** (85 mg, 0.40 mmol) and 1-iodo-4-methoxybenzene (244 mg, 1.04 mmol). Purification by flash column chromatography (2.5–5% diethyl ether/pentane) afforded monoarylated aldehyde **15a** as a colourless oil (36 mg, 28%) followed by diarylated aldehyde **15b** as a pale yellow oil (23 mg, 14%).

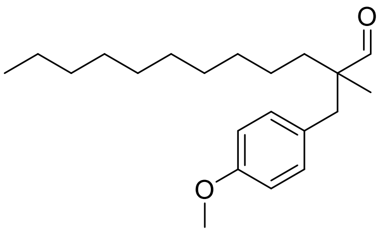

15a

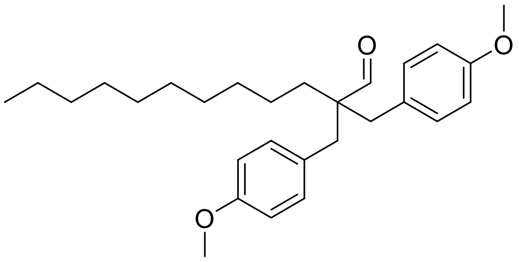

15b

15a: R_f 0.35 (10% diethyl ether/pentane). IR (film)/cm^{-1} 2924, 2853, 1725 (C=O), 1611, 1512, 1463, 1246, 1178, 1036. ^{1}H NMR (400 MHz, $CDCl_3$) δ 9.56 (s, 1H, CHO), 7.01–6.98 (m, 2H, Ar-H), 6.83–6.79 (m, 2H, Ar-H), 3.79 (s, 3H, OCH_3), 2.82 (d, *J* = 13.8 Hz, 1H, C(H)*H*), 2.66 (d, *J* = 13.8 Hz, 1H, C(*H*)H), 1.60–1.53 (m, 1H, CH_2C(H)*H*), 1.44–1.37 (m, 1H, CH_2C(*H*)H), 1.33–1.19 (m, 16H, 8 × CH_2), 0.99 (s, 3H, CH_3), 0.89 (t, *J* = 6.9 Hz, 3H, CH_3). ^{13}C NMR (101 MHz, $CDCl_3$) δ 206.8 (C=O), 158.2 (Ar-C_q), 131.2 (2 × Ar-C), 128.9 (Ar-C_q), 113.6 (2 × Ar-C), 55.2 (OCH_3), 50.4 (*C*(CH_3)), 41.1 (CH_2), 35.6 (CH_2), 31.9 (CH_2), 30.2 (CH_2), 29.6 (2 × CH_2), 29.5 (CH_2), 29.3 (CH_2), 24.2 (CH_2), 22.7 (CH_2), 18.2 (CH_3), 14.1 (CH_3). HRMS (ASAP(SOLID)) m/z Calcd. For $C_{21}H_{35}O_2$ [M+H]$^+$: 319.2637; Found: 319.2635.

15b: R_f 0.21 (10% diethyl ether/pentane). IR (film)/cm^{-1} 2926, 2853, 1723 (C=O), 1611, 1512, 1248, 1178, 1036. ^{1}H NMR (400 MHz, $CDCl_3$) δ 9.62 (s, 1H, CHO), 7.02–6.99 (m, 4H, Ar-H), 6.83–6.80 (m, 4H, Ar-H), 3.79 (s, 6H, 2 × OCH_3), 2.89 (d, *J* = 14.2 Hz, 2H, 2 × C(H)*H*), 2.74 (d, *J* = 14.2 Hz, 2H, 2 × C(*H*)H), 1.46–1.37 (m, 4H, 2 × CH_2), 1.33–1.26 (m, 14H, 7 × CH_2), 0.90 (t, *J* = 6.9 Hz, 3H, CH_3). ^{13}C NMR (101 MHz, $CDCl_3$) δ 207.7 (CHO), 158.2 (2 × Ar-C_q), 131.2 (4 × Ar-C), 128.7 (2 × Ar-C_q), 113.6 (4 × Ar-C), 55.2 (2 × OCH_3), 54.0 (*C*(CHO)), 38.8 (2 × CH_2), 31.9 (CH_2), 31.0 (CH_2), 30.1 (CH_2), 29.6 (2 × CH_2), 29.5 (CH_2), 29.3 (CH_2), 23.7 (CH_2), 22.7 (CH_2), 14.1 (CH_3). HRMS (ASAP(SOLID)) m/z Calcd. For $C_{26}H_{41}O_3$ [M+H]$^+$: 425.3056; Found: 425.3053.

1-(4-Methoxybenzyl)cyclohexane-1-carbaldehyde (16a)

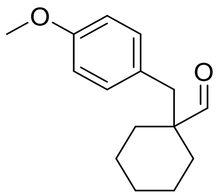

Prepared according to general procedure J using 1-methylcyclohexane-1-carbaldehyde (50.5 mg, 0.40 mmol) and 4-iodoanisole (244 mg, 1.20 mmol). Purification by flash column chromatography (10% diethyl ether/pentane) afforded monoarylated aldehyde **16a** as a colourless oil (28 mg, 30%). R_f 0.26 (10% diethyl ether/pentane). IR (film)/cm^{-1} 2929, 2854, 1718 (C=O), 1610, 1510 (s), 1451, 1300, 1243 (s), 1176, 1109, 1033. ^{1}H NMR (400 MHz, $CDCl_3$) δ 9.51 (s, 1H, CHO), 7.00–6.96 (m, 2H, Ar-H), 6.82–6.78 (m, 2H, Ar-H), 3.79 (s, 3H, OCH_3), 2.67 (s, 2H, CH_2), 1.92–1.86 (m, 2H), 1.65–1.54 (m, 3H), 1.34–1.24 (m, 5H). ^{13}C NMR (101 MHz, $CDCl_3$) δ 207.6 (CHO), 158.3 (Ar-C_q), 131.1 (2 × Ar-C), 128.2 (Ar-C_q), 113.5 (2 × Ar-C), 55.2 (OCH_3), 50.8 (*C*(CHO)), 42.7 (CH_2), 31.1 (2 × CH_2), 25.6 (CH_2), 22.7 (2 × CH_2). HRMS (ESI) m/z Calcd. for $C_{15}H_{21}O_2$ [M+H]$^+$: 233.1536; Found: 233.1534.

3-(Benzyloxy)-2-(4-methoxybenzyl)-2-methylpropanal (17a) and 3-(benzyloxy)-2,2-bis(4-methoxybenzyl)propanal (17b)

Prepared according to general procedure J using 3-(benzyloxy)-2,2-dimethylpropanal (77 mg, 0.40 mmol) **S15** and 1-iodo-4-methoxybenzene (244 mg, 1.04 mmol). Purification by flash column chromatography (2.5–10% diethyl ether/pentane) afforded monoarylated aldehyde **17a** as a colourless oil (39 mg, 33%) followed by diarylated aldehyde **17b** as a pale yellow oil (33 mg, 21%).

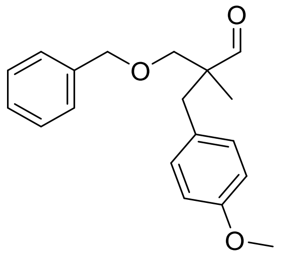

17a

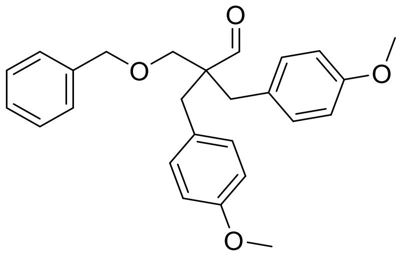

17b

17a: R_f 0.15 (10% diethyl ether/pentane). IR (film)/cm^{-1} 2903, 2858, 2721, 1722 (s, C=O), 1610, 1510, 1492, 1251, 1072 (s). ^{1}H NMR (400 MHz, $CDCl_3$) δ 9.68 (s, 1H, CHO), 7.39–7.31 (m, 5H, Ph-H), 7.03–6.99 (m, 2H, Ar-H), 6.81–6.77 (m, 2H, Ar-H), 4.51 (s, 2H, CH_2), 3.79 (s, 3H, OCH_3), 3.41 (s, 2H, CH_2), 2.92 (d, *J* = 13.7 Hz, 1H, C(*H*)H), 2.78 (d, *J* = 13.7 Hz, 1H, C(H)*H*), 1.00 (s, 3H, CH_3). ^{13}C NMR (101 MHz, $CDCl_3$) δ 205.5 (CHO), 158.3 (Ar-C_q), 138.0 (Ph-C_q), 131.3 (2 × Ar-C), 128.4 (2 × Ph-C), 128.3 (Ar-C_q), 127.7 (Ph-C), 127.6 (2 × Ph-C), 113.6 (2 × Ar-C), 73.3 (CH_2), 72.5 (CH_2), 55.2 (OCH_3), 51.2 (*C*(CH_3)), 37.3 (CH_2), 16.7 (CH_3). HRMS (pNSI) m/z Calcd. for $C_{19}H_{26}O_3N$ $[M+NH_4]^+$: 316.1907; Found: 316.1910.

17b: R_f 0.09 (10% diethyl ether/pentane). IR (film)/cm^{-1} 3003, 2939, 2858, 2721, 1733 (s, C=O), 1610, 1510, 1251, 1444, 1072 (s), 1035. ^{1}H NMR (400 MHz, $CDCl_3$) δ 9.68 (s, 1H, CHO), 7.42–7.33 (m, 5H, Ph-H), 7.03–6.99 (m, 4H, Ar-H), 6.80– 6.76 (m, 4H, Ar-H), 4.48 (s, 2H, OCH_2), 3.79 (s, 6H, 2 × OCH_3), 3.35 (s, 2H, OCH_2), 3.01 (d, *J* = 13.9 Hz, 2H, 2 × C(*H*)H), 2.88 (d, *J* = 13.9 Hz, 2H, 2 × C(H)*H*). ^{13}C NMR (101 MHz, $CDCl_3$) δ 205.7 (CHO), 158.3 (2 × Ar-C_q), 138.0 (Ph-C_q), 131.2 (4 × Ar-C), 128.4 (2 × Ph-C), 128.3 (2 × Ar-C_q), 127.8 (2 × Ph-C), 127.7 (Ph-C), 113.7 (4 × Ar-C), 73.3 (OCH_2), 68.3 (OCH_2), 55.8 (*C*(CHO)), 55.2 (2 × OCH_3), 37.7 (2 × CH_2). HRMS (pNSI) m/z Calcd. for $C_{26}H_{32}O_4N$ $[M+NH_4]^+$: 422.2326; Found: 422.2322.

3-((2-Chlorobenzyl)oxy)-2-(4-methoxybenzyl)-2-methylpropanal (18a) and 2-(4-methoxybenzyl)-3-(4-methoxyphenyl)acrylaldehyde (18b)

Prepared according to general procedure J using 3-((2-chlorobenzyl)oxy)-2,2-dimethylpropanal (92 mg, 0.40 mmol) **S17** and 1-iodo-4-methoxybenzene (244 mg, 1.04 mmol). Purification by flash column chromatography (20% diethyl ether/pentane) afforded monoarylated aldehyde **18a** as a colourless oil (40 mg, 30%) followed by diarylated aldehyde **18b** as an off-white solid (43 mg, 24%).

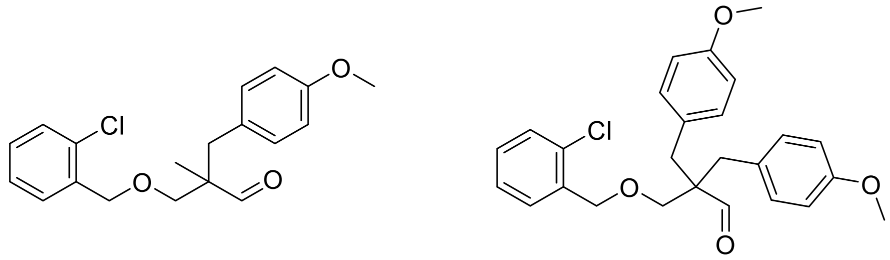

18a **18b**

18a: R_f 0.32 (7.5% EtOAc/pentane). IR (film)/cm^{-1} 2932, 2836, 2704, 1725 (m, C=O), 1611, 1511, 1441, 1245, 1178, 1101, 1034. ^{1}H NMR (400 MHz, $CDCl_3$) δ 9.71 (s, 1H, CHO), 7.47 (dd, *J* = 7.4, 1.7 Hz, 1H, Ar-H), 7.37 (dd, *J* = 7.4, 1.7 Hz, 1H, Ar-H), 7.32–7.23 (m, 2H, Ar-H), 7.06–7.02 (m, 2H, Ar-H), 6.83–6.79 (m, 2H, Ar-H), 4.60 (s, 2H, OCH_2), 3.79 (s, 3H, OCH_3), 3.53–3.48 (m, 2H, OCH_2), 2.95 (d, *J* = 13.7 Hz, 1H C(*H*)H), 2.80 (d, *J* = 13.7 Hz, 1H C(H)*H*), 1.04 (s, 3H, CH_3). ^{13}C NMR (101 MHz, $CDCl_3$) δ 205.4 (CHO), 158.3 (Ar-C_q), 135.7 (Ar-C_q), 132.7 (Ar-C_q), 131.3 (2 × Ar-C), 129.2 (Ar-C), 128.8 (Ar-C), 128.7 (Ar-C), 128.2 (Ar-C_q), 126.8 (Ar-C), 113.6 (2 × Ar-C), 73.0 (OCH_2), 70.3 (OCH_2), 55.2 (OCH_3), 51.3 (*C*(CH_3)), 37.3 (CH_2), 16.7 (CH_3). HRMS (pNSI) m/z Calcd. for $C_{19}H_{25}O_3N_1Cl_1$ $[M+NH_4]^+$: 350.1517; Found: 350.1521.

18b: R_f 0.20 (7.5% EtOAc/pentane). mp = 69–73 °C. IR (film)/cm^{-1} 2836, 2900, 2836, 1714 (m, C=O), 1611, 1510, 1441, 1243 (s), 1177, 1094, 1037. ^{1}H NMR (400 MHz, $CDCl_3$) δ 9.71 (s, 1H, CHO), 7.53 (dd, *J* = 7.2, 1.8 Hz, 1H, Ar-H), 7.41 (dd, *J* = 7.2, 1.8 Hz, 1H, Ar-H), 7.34–7.26 (m, 2H, Ar-H), 7.04–7.01 (m, 4H, Ar-H), 6.80–6.77 (m, 4H, Ar-H), 4.56 (s, 2H, OCH_2), 3.78 (s, 6H, 2 × OCH_3), 3.42 (s, 2H, OCH_2), 3.03 (d, *J* = 13.9 Hz, 2H, 2 × C(*H*)H), 2.90 (d, *J* = 13.9 Hz, 2H, 2 × C(H)*H*). ^{13}C NMR (101 MHz, $CDCl_3$) δ 205.6 (CHO), 158.3 (2 × Ar-C_q), 135.8 (Ar-C_q), 132.9 (Ar-C_q), 131.2 (4 × Ar-C), 129.3 (Ar-C), 129.2 (Ar-C), 128.8 (Ar-C), 128.1 (Ar-C), 126.8 (2 × Ar-C_q), 113.7 (4 × Ar-C), 70.2 (OCH_2), 68.7 (OCH_2), 55.8 (*C*(CHO)), 55.2 (2 × OCH_3), 37.7 (2 × CH_2). HRMS (pNSI) m/z Calcd. for $C_{26}H_{31}O_4N_1Cl_1$ $[M+NH_4]^+$: 456.1936; Found: 456.1923.

2-(4-Methoxybenzyl)-2-methyl-4-phenylbutanal (19a) and 2,2-bis(4-methoxybenzyl)-4-phenylbutanal (19b)

Prepared according to general procedure J using 2,2-dimethyl-4-phenylbutanal **S19** (70 mg, 0.40 mmol) and 1-iodo-4-methoxybenzene (244 mg, 1.04 mmol). Purification by flash column chromatography (5% diethyl ether/pentane) afforded monoarylated aldehyde **19a** as an amorphous white solid (21 mg, 19%) followed by diarylated aldehyde **19b** as a yellow oil (20 mg, 13%).

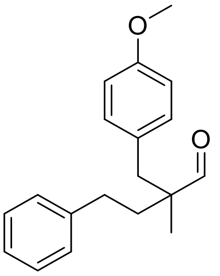

19a

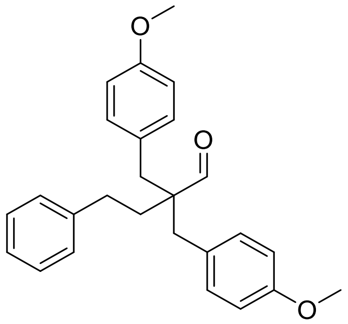

19b

19a: R_f 0.11 (5% diethyl ether/pentane). IR (film)/cm^{-1} 2931, 1725 (C=O), 1609, 1510, 1457, 1298, 1246, 1176, 1032. ^{1}H NMR (400 MHz, $CDCl_3$) δ 9.62 (s, 1H, CHO), 7.31–7.28 (m, 2H, Ph-H), 7.22–7.16 (m, 3H, Ph-H), 7.05–7.02 (m, 2H, Ar-H), 6.84–6.80 (m, 2H, Ar-H), 3.79 (s, 3H, OCH_3), 2.88 (d, *J* = 13.9 Hz, 1H, C(*H*)H), 2.75 (d, *J* = 13.9 Hz, 1H, C(H)*H*), 2.63–2.49 (m, 2H, CH_2), 1.91 (ddd, *J* = 13.9, 12.2, 5.2 Hz, 1H, C(*H*)H), 1.73 (ddd, *J* = 13.9, 12.2, 5.6 Hz, 1H, C(H)*H*), 1.11 (s, 3H, CH_3). ^{13}C NMR (101 MHz, $CDCl_3$) δ 206.2 (CHO), 158.3 (Ar-C_q), 141.8 (Ar-C_q), 131.2 (2 × Ar-C), 128.5 (2 × Ph-C), 128.2 (2 × Ph-C + Ph-C_q), 126.0 (Ph-C), 113.6 (2 × Ar-C), 55.2 (OCH_3), 50.4 (*C*(CH_3)), 41.2 (CH_2), 37.4 (CH_2), 30.7 (CH_2), 18.3 (CH_3). HRMS (ASAP(SOLID)) m/z Calcd. for $C_{19}H_{23}O_2$ $[M+H]^+$: 283.1698; Found: 283.1693.

19b: R_f 0.07 (5% diethyl ether/pentane). IR (film)/cm^{-1} 2934, 1720 (C=O), 1611, 1510, 1454, 1301, 1246, 1178, 1032. ^{1}H NMR (400 MHz, $CDCl_3$) δ 9.72 (s, 1H, CHO), 7.31–7.27 (m, 2H, Ph-H), 7.22–7.19 (m, 1H, Ph-H), 7.14–7.08 (m, 6H, 4 × Ar-H + 2 × Ph-H), 6.87–6.84 (m, 4H, Ar-H), 3.82 (s, 6H, 2 × OCH_3), 3.04 (d, *J* = 14.3 Hz, 2H, 2 × C(*H*)H), 2.87 (d, *J* = 14.3 Hz, 2H, 2 × C(H)*H*), 2.74–2.70 (m, 2H, CH_2), 1.82–1.78 (m, 2H, CH_2).^{13}C NMR (101 MHz, $CDCl_3$) δ 207.1 (CHO), 158.4 (2 × Ar-C_q), 141.8 (2 × Ar-C_q), 131.2 (4 × Ar-C), 128.44 (2 × Ph-C), 128.36 (Ph-C_q), 128.2 (2 × Ph-C), 126.0 (Ph-C), 113.8 (4 × Ar-C), 55.2 (2 × OCH_3), 54.0 (*C*(CHO)), 39.0 (2 × CH_2), 32.9 (CH_2), 30.1 (CH_2). HRMS (pNSI) m/z Calcd. for $C_{26}H_{32}O_3N_1$ $[M+NH_4]^+$: 406.2377; Found: 406.2375.

3-(Benzyloxy)-2-(4-methoxybenzyl)-2-(methoxymethyl)propanal (20a)

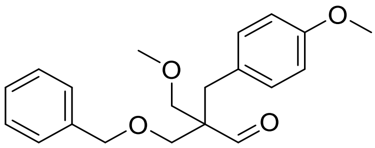

Prepared according to general procedure J using 3-(benzyloxy)-2-(methoxymethyl)-2-methylpropanal (89 mg, 0.40 mmol) and 1-iodo-4-methoxybenzene (244 mg, 1.20 mmol). Purification by flash column chromatography (15% diethyl ether/pentane) afforded arylated aldehyde **20a** as a colourless oil (34 mg, 40%). R_f 0.30 (15% diethyl ether/pentane). IR (film)/cm^{-1} 2836, 1727 (s, C=O), 1611, 1511, 1454, 1364, 1246, 1178, 1099, 1031. ^{1}H NMR (400 MHz, CDCl3) δ 9.71 (s, 1H, CHO), 7.39–7.30 (m, 5H, Ph-H), 7.05–7.01 (m, 2H, Ar-H), 6.82–6.78 (m, 2H, Ar-H), 4.51 (s, 2H, OCH_2), 3.79 (s, 3H, ArOCH_3), 3.53 (d, *J* = 2.7 Hz, 2H, OCH_2), 3.44 (s, 2H, OCH_2), 3.33 (s, 3H, OCH_3), 2.92 (s, 2H, CH_2). ^{13}C NMR (101 MHz, $CDCl_3$) δ 204.5 (C=O), 158.3 (Ar-C_q), 138.0 (Ph-C_q), 131.2 (2 × Ar-C), 128.4 (2 × Ph-C), 127.8 (Ar-C_q), 127.7 (Ph-C), 127.6 (2 × Ph-C), 113.6 (2 × Ar-C), 73.4 (OCH_2), 71.2 (OCH_2), 68.7 (OCH_2), 59.1 (OCH_3), 55.9 (*C*(CHO)), 55.1 (ArOCH_3), 33.2 (CH_2). HRMS (pNSI) m/z Calcd. for $C_{20}H_{28}O_4N_1$ $[M+NH_4]^+$: 346.2013; Found: 346.2014.

Synthesis of palladacycle dimer (2a-Pd-dimer)

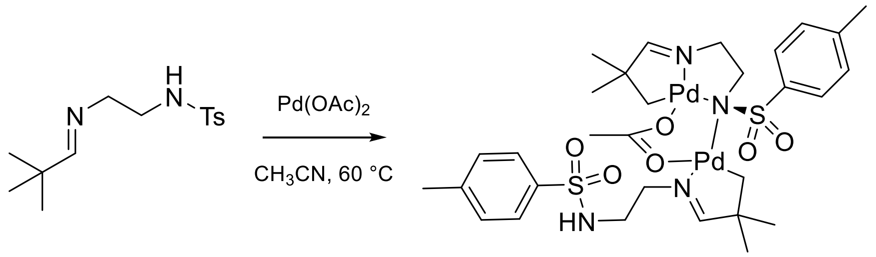

Palladium acetate (673 mg, 3.00 mmol) was added to a stirred solution of *N*-{2-[(*E*)-(2,2-dimethylpropylidene)amino]ethyl}-4-methylbenzene-1-sulfonamide **2a** (846 mg, 3.00 mmol) in CH_3CN (6 mL) and the reaction was stirred at 60 °C for 3 h. Toluene (25 mL) was added and the reaction was filtered through Celite and concentrated to afford the crude palladacycle **2a-Pd-dimer** (1.39 g, 56%). A sample was purified by recrystallisation in CH_2Cl_2/pentane at -20 °C to obtain analytical data. mp = decomposition occurs at 136 °C. IR (film)/cm^{-1} 3155, 2956, 2925, 1557, 1401, 1322, 1299, 1157, 1143, 1088. ^{1}H NMR (400 MHz, $CDCl_3$) δ 8.19 (d, *J* = 8.1 Hz, 2H, Ar-H), 7.97 (d, *J* = 8.1 Hz, 2H, Ar-H), 7.53 (s, 1H, N=CH), 7.47 (s, 1H, N=CH), 7.36 (d, *J* = 8.1 Hz, 2H, Ar-H), 7.30 (d, *J* = 8.1 Hz, 2H, Ar-H), 6.69 (t, *J* = 4.7 Hz, 1H, NH), 3.95 (t, *J* = 11.8 Hz, 1H, C(H)*H*), 3.65–3.56 (m, 2H, CH_2), 3.50–3.46 (m, 1H, C(*H*)H), 3.19–3.12 (m, 2H, C(*H*)H + C(H)*H*), 2.70 (dd, *J* = 12.0, 3.8 Hz, 1H, C(*H*)H), 2.44–2.41 (m, 7H, C(*H*)H + 2 × Ar-CH_3), 2.36 (d, *J* = 8.8 Hz, 1H, PdC(H)*H*), 2.26 (d, *J* = 8.5 Hz, 1H, PdC(H)*H*), 2.09–2.07 (m, 4H, PdC(*H*)H + CO_2CH_3), 1.65 (d, *J* = 8.5 Hz, 1H, PdC(*H*)H), 1.16–1.07 (m, 12H, 2 × $C(CH_3)_2$). ^{13}C NMR (101 MHz, $CDCl_3$) δ 189.0 (C=N), 183.7 (C=N), 181.2 (C=O), 143.1 (Ar-C_q), 142.1 (Ar-C_q), 137.2 (2 × Ar-C_q), 129.7 (2 × Ar-H), 129.4 (2 × Ar-H), 128.7 (2 × Ar-H), 127.4 (2 × Ar-H), 58.6 (CH_2), 57.3 (CH_2), 54.0 (*C*$(CH_3)_2$), 50.8 (CH_2), 50.1 (*C*$(CH_3)_2$), 44.4 (CH_2), 34.4 ($PdCH_2$), 33.8 ($PdCH_2$), 28.4 (CH_3), 26.3 (CH_3), 26.2 (CH_3), 24.5 (CH_3), 24.0 (CO_2*C*H_3), 21.5 (2 × Ar-CH_3).

Arylation of palladacycle dimer (2a-Pd-dimer)

Palladacycle dimer **2a-Pd-dimer** (33.3 mg, 0.04 mmol), AgTFA (35.2 mg, 0.16 mmol), 1-iodo-4-methoxybenzene (55.7 mg, 0.24 mmol) and AcOH (0.13 mL) were combined in a flame dried microwave vial. The vial was purged with argon, sealed and heated to 120 °C for 24 h. The reaction was allowed to cool to room temperature, dissolved in CH_2Cl_2, filtered through a short plug of silica, washed with CH_2Cl_2 and concentrated under reduced pressure. Yields of the arylated aldehyde products were calculated by ^{1}H NMR using gem dimethyl (mono: 1.05 ppm), methyl (di: 0.97 ppm) and methylene (tri: 2.85 ppm) signals following in comparison to a known amount of 1,3,5-trimethoxybenzene as an internal standard.

Arylation of pivaldehyde using palladacycle dimer (2a-Pd-dimer) as the catalyst

Pivaldehyde (22 µL, 0.20 mmol), *N*-(2-aminoethyl)-4-methylbenzenesulfonamide **1a** (21.4 mg, 0.10 mmol), 1-iodo-4-methoxybenzene (122 mg, 0.52 mmol), **2a-Pd-dimer** (4.2 mg, 2.5 mol%), silver trifluoroacetate (88 mg, 0.40 mmol), DMSO (14.2 µL, 0.20 mmol), acetic acid (0.2 mL) and HFIP (0.2 mL) were combined in a flame dried microwave vial. The vial was purged with argon, sealed and heated to 130 °C for 3 h. The reaction was allowed to cool to room temperature, dissolved in CH_2Cl_2, filtered through a short plug of silica, washed with CH_2Cl_2 and concentrated under reduced pressure. Yields of the arylated aldehyde products were calculated by ^{1}H NMR using *gem*-dimethyl (mono: 1.05 ppm), methyl (di: 0.97 ppm) and methylene (tri: 2.85 ppm) signals in comparison to a known amount of 1,3,5-trimethoxybenzene as an internal standard.

Formation of palladacycle monomer as a solution in AcOD-*d₄* (2a-Pd-monomer)

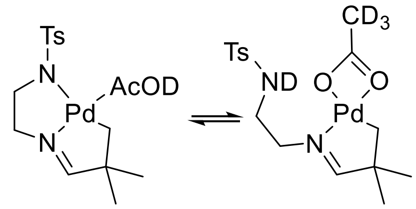

Prepared from dissolving dimer **2a-Pd-dimer** in AcOD-d_4. IR (film)/cm^{-1} 2962, 1563, 1402, 1267, 1157, 1090. ^{1}H NMR (400 MHz, AcOD-d_4) δ 7.80 (d, J = 8.1 Hz, 2H, Ar-H), 7.66 (s, 1H, N=CH), 7.38 (d, J = 8.1 Hz, 2H, Ar-H), 3.38 (bs, 2H, CH_2), 3.20 (bs, 1H, CH_2), 2.43 (s, 3H, Ar-CH_3), 2.33 (bs, 2H, $PdCH_2$), 2.07 (s, 3H, CO_2CH_3), 1.22 (s, 6H, $C(CH_3)_2$). ^{13}C NMR (101 MHz, AcOD-d_4) δ 191.5 (C=N), 178.4 (Ar-C_q), 144.8 (Ar-C_q), 138.2 (2 × Ar-C), 130.8 (2 × Ar-C), 128.1 (CH_2), 60.6 ($C(CH_3)_2$), 51.3 (CH_2), 43.0 ($PdCH_2$), 26.2 ($C(CH_3)_2$), 21.6 (Ar-CH_3).

NMR study of the formation of palladacycle monomer in MeCN-*d₃* at 60 °C

Palladium acetate (51 mg, 0.23 mmol), imine **2a** (63 mg, 0.23 mmol) and MeCN-d_3 (0.75 mL) were combined in a Young's NMR tube. The reaction was heated to 60 °C and a ^{1}H NMR spectrum was recorded every 5 min for 3 h.

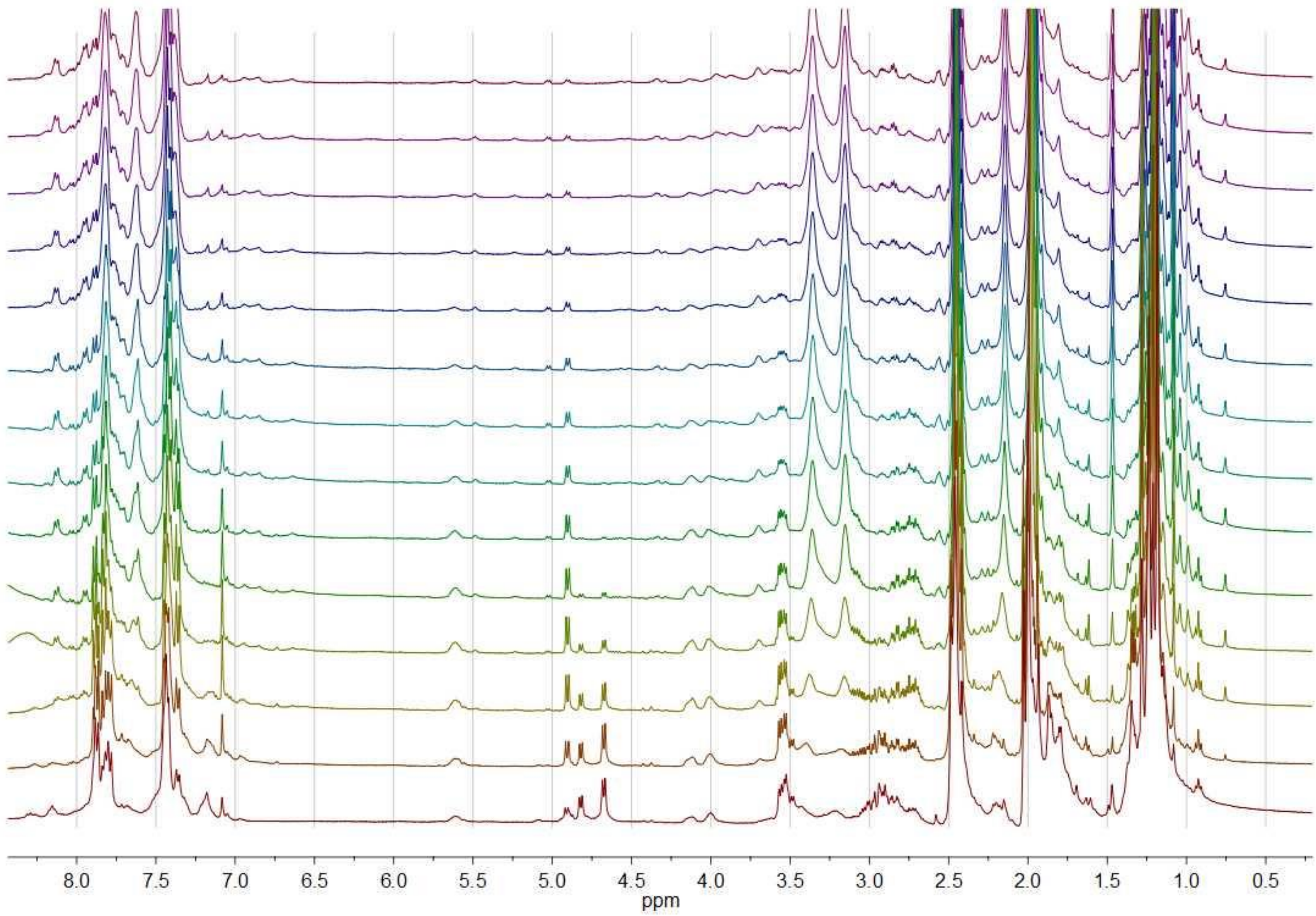

Figure S8: ^{1}H NMR spectra of cyclometallation reaction in 5 minute increments for the first 60 minutes (bottlom to top)

A representative selection of spectra are shown in figure S8 which indicates coordination of catalyst happens rapidly then cyclometallation to give cyclometallated monomer signals (CH_2's of the ethylene diamine chain at 3.12 and 3.36 ppm and Pd-CH_2 at 2.17 ppm) occurs gradually at 60 °C reaching maximum conversion (see Figure S9) at approximately 80 minutes, at which time the spectra is comparable to the monomer **2a-Pd-monomer** formed when dissolving dimer **2a-Pd-dimer** in AcOD-d_4. The disappearance of the broad signal at 5.6 ppm may correspond to the deprotonation and binding of the sulfonamide through N. Sharp peaks between 4.5–5.0 ppm may be indicative of a coordinated, charged intermediate Pd species.

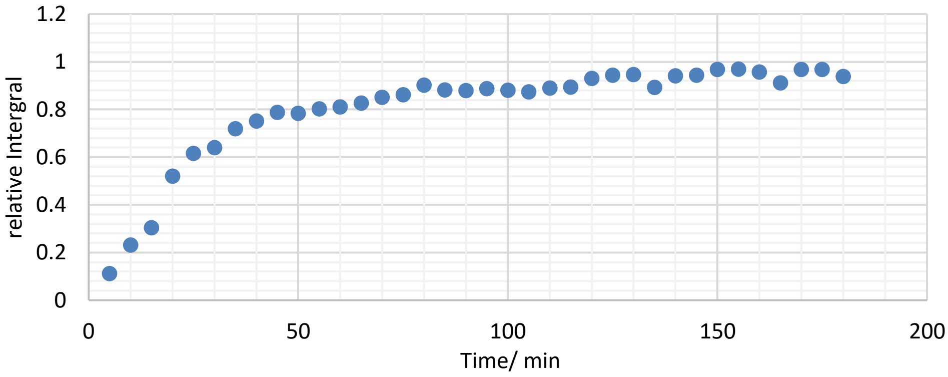

Figure S9: Change in relative integral of Pd-CH_2 signal (2.17 ppm) over time

X-Ray crystal structure of metallacycle 2a-Pd-dimer

Crystals suitable for X-ray analysis were grown by vapour diffusion of pentane into a concentrated CH_2Cl_2 solution at 25 °C.

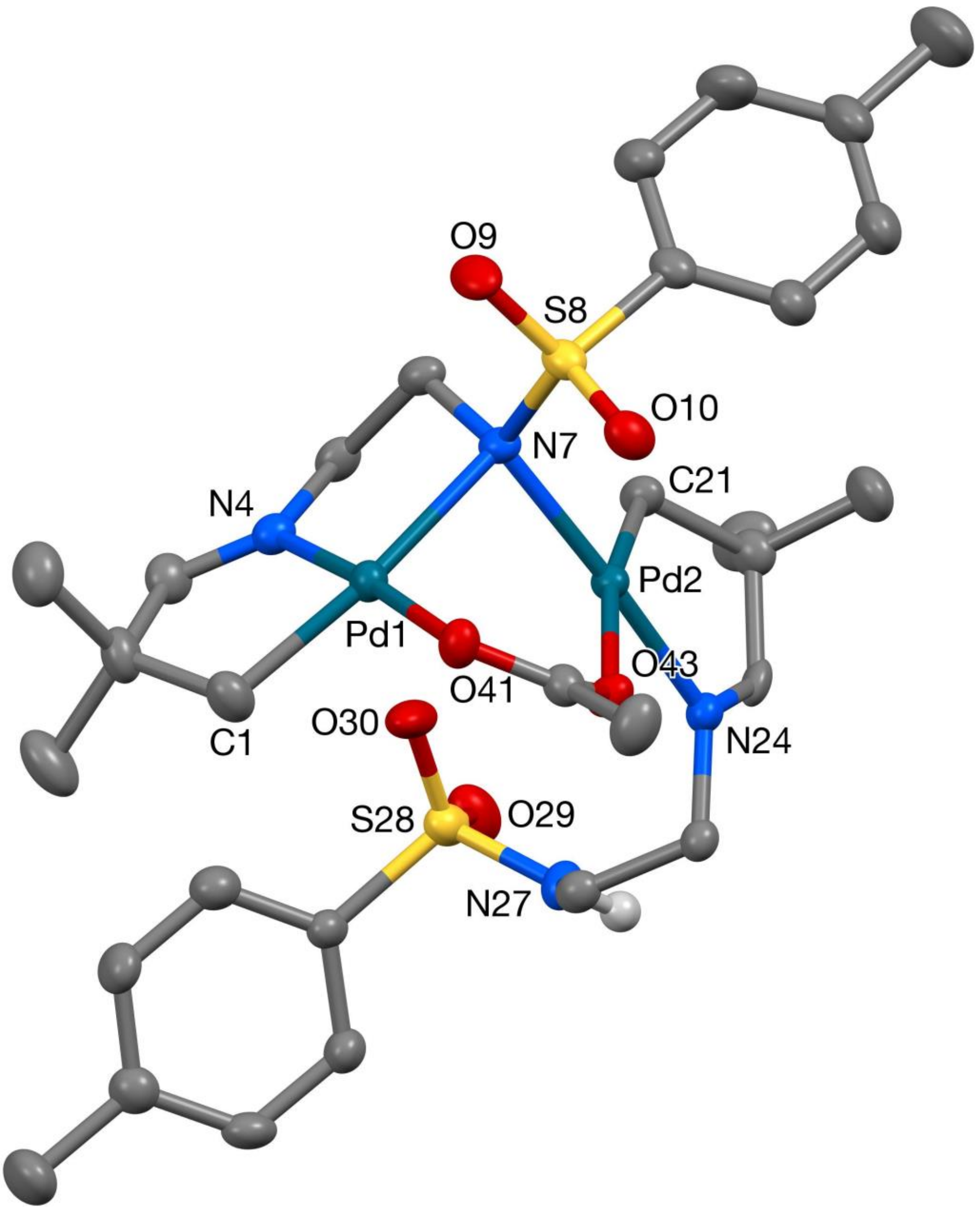

Fig. S10 The crystal structure of **2a-Pd-dimer** (50% probability ellipsoids).

The X-ray crystal structure of 2a-Pd-dimer

Crystal data for **2a-Pd-dimer**: $C_{30}H_{44}N_4O_6Pd_2S_2$, M = 833.61, monoclinic, $P2_1/n$ (no. 14), a = 13.3652(4), b = 18.9012(7), c = 14.5561(5) Å, β = 104.314(4)°, V = 3563.0(2) Å^3, Z = 4, D_c = 1.554 g cm^{-3}, μ(Mo-Kα) = 1.171 mm^{-1}, T = 173 K, colourless tablets, Agilent Xcalibur 3 E diffractometer; 7079 independent measured reflections (R_{int} = 0.0243), F^2 refinement,[X1,X2] R_1(obs) = 0.0345, wR_2(all) = 0.0736, 5616 independent observed absorption-corrected reflections [$|F_o| > 4\sigma(|F_o|)$, $2\theta_{max}$ = 57°], 408 parameters. CCDC 1534094.

The N27–H hydrogen atom in the structure of **2a-Pd-dimer** was located from a Δ*F* map and refined freely subject to an N–H distance constraint of 0.90 Å.

References

[X1] SHELXTL v5.1, Bruker AXS, Madison, WI, 1998.

[X2] SHELX-2013, G.M. Sheldrick, *Acta Cryst.*, 2015, **C71**, 3-8.

Bond lengths [Å] and angles [°] for 2a-Pd-dimer

Bond lengths [Å]

Pd(1)-N(4)	1.952(3)
Pd(1)-C(1)	2.009(3)
Pd(1)-O(41)	2.046(2)
Pd(1)-N(7)	2.253(3)
Pd(1)-Pd(2)	3.1098(3)
Pd(2)-C(21)	2.003(3)
Pd(2)-N(24)	2.018(3)
Pd(2)-N(7)	2.085(3)
Pd(2)-O(43)	2.244(2)
C(1)-C(2)	1.538(5)
C(2)-C(3)	1.501(5)
C(2)-C(19)	1.527(5)
C(2)-C(18)	1.535(5)
C(3)-N(4)	1.271(4)
N(4)-C(5)	1.462(4)
C(5)-C(6)	1.509(5)
C(6)-N(7)	1.495(4)
N(7)-S(8)	1.628(3)
S(8)-O(10)	1.434(2)
S(8)-O(9)	1.443(2)
S(8)-C(11)	1.776(3)
C(11)-C(12)	1.375(5)
C(11)-C(16)	1.379(5)
C(12)-C(13)	1.384(5)
C(13)-C(14)	1.383(6)
C(14)-C(15)	1.377(6)
C(14)-C(17)	1.521(5)
C(15)-C(16)	1.379(5)
C(21)-C(22)	1.536(5)
C(22)-C(23)	1.489(5)
C(22)-C(39)	1.532(5)
C(22)-C(38)	1.541(5)
C(23)-N(24)	1.272(4)
N(24)-C(25)	1.459(4)
C(25)-C(26)	1.516(4)
C(26)-N(27)	1.451(4)
N(27)-S(28)	1.600(3)
S(28)-O(29)	1.427(3)
S(28)-O(30)	1.431(3)
S(28)-C(31)	1.763(3)
C(31)-C(36)	1.375(5)
C(31)-C(32)	1.389(5)
C(32)-C(33)	1.384(5)
C(33)-C(34)	1.401(5)
C(34)-C(35)	1.382(5)
C(34)-C(37)	1.508(5)
C(35)-C(36)	1.386(5)
O(41)-C(42)	1.273(4)
C(42)-O(43)	1.238(4)
C(42)-C(44)	1.508(5)

Bond Angles [°]

N(4)-Pd(1)-C(1)	81.83(13)	C(12)-C(11)-C(16)	120.3(3)
N(4)-Pd(1)-O(41)	175.12(11)	C(12)-C(11)-S(8)	120.2(3)
C(1)-Pd(1)-O(41)	93.47(12)	C(16)-C(11)-S(8)	119.4(3)
N(4)-Pd(1)-N(7)	82.65(11)	C(11)-C(12)-C(13)	119.3(4)
C(1)-Pd(1)-N(7)	164.10(13)	C(14)-C(13)-C(12)	121.4(4)
O(41)-Pd(1)-N(7)	101.96(9)	C(15)-C(14)-C(13)	118.0(4)
N(4)-Pd(1)-Pd(2)	93.23(8)	C(15)-C(14)-C(17)	121.4(4)
C(1)-Pd(1)-Pd(2)	135.67(12)	C(13)-C(14)-C(17)	120.6(4)
O(41)-Pd(1)-Pd(2)	89.22(6)	C(14)-C(15)-C(16)	121.5(4)
N(7)-Pd(1)-Pd(2)	42.08(7)	C(11)-C(16)-C(15)	119.5(4)
C(21)-Pd(2)-N(24)	81.27(12)	C(22)-C(21)-Pd(2)	109.5(2)
C(21)-Pd(2)-N(7)	94.03(12)	C(23)-C(22)-C(39)	110.3(3)
N(24)-Pd(2)-N(7)	172.89(11)	C(23)-C(22)-C(21)	107.3(3)
C(21)-Pd(2)-O(43)	168.51(11)	C(39)-C(22)-C(21)	111.6(3)
N(24)-Pd(2)-O(43)	88.99(10)	C(23)-C(22)-C(38)	107.2(3)
N(7)-Pd(2)-O(43)	96.22(9)	C(39)-C(22)-C(38)	110.0(3)
C(21)-Pd(2)-Pd(1)	126.27(9)	C(21)-C(22)-C(38)	110.3(3)
N(24)-Pd(2)-Pd(1)	133.23(8)	N(24)-C(23)-C(22)	118.0(3)
N(7)-Pd(2)-Pd(1)	46.41(7)	C(23)-N(24)-C(25)	119.1(3)
O(43)-Pd(2)-Pd(1)	65.08(6)	C(23)-N(24)-Pd(2)	116.0(2)
C(2)-C(1)-Pd(1)	110.0(2)	C(25)-N(24)-Pd(2)	124.0(2)
C(3)-C(2)-C(19)	106.4(3)	N(24)-C(25)-C(26)	109.1(3)
C(3)-C(2)-C(18)	111.2(3)	N(27)-C(26)-C(25)	111.3(3)
C(19)-C(2)-C(18)	109.5(3)	C(26)-N(27)-S(28)	122.6(2)
C(3)-C(2)-C(1)	107.3(3)	O(29)-S(28)-O(30)	119.02(17)
C(19)-C(2)-C(1)	111.0(3)	O(29)-S(28)-N(27)	105.98(15)
C(18)-C(2)-C(1)	111.2(3)	O(30)-S(28)-N(27)	110.24(15)
N(4)-C(3)-C(2)	116.9(3)	O(29)-S(28)-C(31)	108.31(16)
C(3)-N(4)-C(5)	126.0(3)	O(30)-S(28)-C(31)	106.27(16)
C(3)-N(4)-Pd(1)	118.8(2)	N(27)-S(28)-C(31)	106.38(15)
C(5)-N(4)-Pd(1)	114.8(2)	C(36)-C(31)-C(32)	120.0(3)
N(4)-C(5)-C(6)	108.6(3)	C(36)-C(31)-S(28)	119.1(3)
N(7)-C(6)-C(5)	110.6(3)	C(32)-C(31)-S(28)	120.8(3)
C(6)-N(7)-S(8)	112.7(2)	C(33)-C(32)-C(31)	119.9(3)
C(6)-N(7)-Pd(2)	119.0(2)	C(32)-C(33)-C(34)	120.8(3)
S(8)-N(7)-Pd(2)	114.37(14)	C(35)-C(34)-C(33)	117.9(3)
C(6)-N(7)-Pd(1)	102.00(18)	C(35)-C(34)-C(37)	121.6(3)
S(8)-N(7)-Pd(1)	114.65(13)	C(33)-C(34)-C(37)	120.5(4)
Pd(2)-N(7)-Pd(1)	91.51(10)	C(34)-C(35)-C(36)	121.7(3)
O(10)-S(8)-O(9)	117.72(16)	C(31)-C(36)-C(35)	119.6(3)
O(10)-S(8)-N(7)	108.44(15)	C(42)-O(41)-Pd(1)	114.5(2)
O(9)-S(8)-N(7)	109.52(14)	O(43)-C(42)-O(41)	125.8(3)
O(10)-S(8)-C(11)	106.44(16)	O(43)-C(42)-C(44)	118.9(3)
O(9)-S(8)-C(11)	105.79(16)	O(41)-C(42)-C(44)	115.4(3)
N(7)-S(8)-C(11)	108.57(15)	C(42)-O(43)-Pd(2)	134.6(2)

References

1 D. H. Peacock and U. C. Dutta, *J. Chem. Soc.*, 1934, 1303–1305.
2 J. Wu, X.-L. Hou and L.-X. Dai, *J. Org. Chem.*, 2000, **65**, 1344–1348.
3 R. N. Salvatore, A. S. Nagle and K. W. Jung, *J. Org. Chem.*, 2002, **67**, 674–683.
4 J. Tan, W. Tang, Y. Sun, Z. Jiang, F. Chen, L. Xu, Q. Fan and J. Xiao, *Tetrahedron*, 2011, **67**, 6206–6213.
5 M. S. Abdel-Maksoud, M.-R. Kim, M. I. El-Gamal, M. M. Gamal El-Din, J. Tae, H. S. Choi, K.-T. Lee, K. H. Yoo and C.-H. Oh, *Eur. J. Med. Chem.*, 2015, **95**, 453–463.
6 S. K. Mangawa, S. K. Bagh, K. Sharma and S. K. Awasthi, *Tetrahedron Lett.*, 2015, **56**, 1960–1963.
7 R. M. Lopez and G. C. Fu, *Tetrahedron*, 1997, **53**, 16349–16354.
8 P.-X. Ling, S.-L. Fang, X.-S. Yin, K. Chen, B.-Z. Sun and B.-F. Shi, *Chem. Eur. J.*, 2015, **21**, 17503–17507.
9 W. J. Ryan, W. K. Banner, J. L. Wiley, B. R. Martin and R. K. Razdan, *J. Med. Chem.*, 1997, **40**, 3617–3625.
10 H. Fujioka, A. Goto, K. Otake, O. Kubo, Y. Sawama and T. Maegawa, *Chem. Commun.*, 2011, **47**, 9894.
11 S. Takano, M. Akiyama, S. Sato and K. Ogasawara, *Chem. Lett.*, 1983, **12**, 1593–1596.
12 E. L. Eliel, L. Clawson and D. E. Knox, *J. Org. Chem.*, 1985, **50**, 2707–2711.
13 M. A. Blanchette, M. S. Malamas, M. H. Nantz, J. C. Roberts, P. Somfai, D. C. Whritenour, S. Masamune, M. Kageyama and T. Tamura, *J. Org. Chem.*, 1989, **54**, 2817–2825.
14 Y. Yang, J. Wang and M. Kayser, *Tetrahedron: Asymmetry*, 2007, **18**, 2021–2029.
15 S. H. Christensen, T. Holm and R. Madsen, *Tetrahedron*, 2014, **70**, 4942–4946.
16 L.-P. B. Beaulieu, D. S. Roman, F. Vallée and A. B. Charette, *Chem. Commun.*, 2012, **48**, 8249.
17 P. C. Too, G. H. Chan, Y. L. Tnay, H. Hirao and S. Chiba, *Angew. Chem. Int. Ed.*, 2016, **55**, 3719–3723.
18 M. Motoi, H. Suda, K. Shimamura, S. Nagahara, M. Takei and S. Kanoh, *Bull. Chem. Soc. Jpn.*, 1988, **61**, 1653–1659.
19 N. Harada, J. Yasuhara, Y. Motoyama, O. Fujimura, T. Tsuji, T. Takahashi, Y. Takahashi and H. Nagashima, *Bull. Chem. Soc. Jpn.*, 2011, **84**, 26–39.
20 Y. Nishimoto, M. Yasuda and A. Baba, *Org. Lett.*, 2007, **9**, 4931–4934.
21 J. Peng, C. Chen and C. Xi, *Chem. Sci.*, 2016, **7**, 1383–1387.
22 A. R. Martin and L. A. Gharat, *Heterocycles*, 1996, **43**, 185.
23 Y.-F. Zhang, H.-W. Zhao, H. Wang, J.-B. Wei and Z.-J. Shi, *Angew. Chem. Int. Ed.*, 2015, **54**, 13686–13690.

^{1}H and ^{13}C NMR spectra of selected compounds

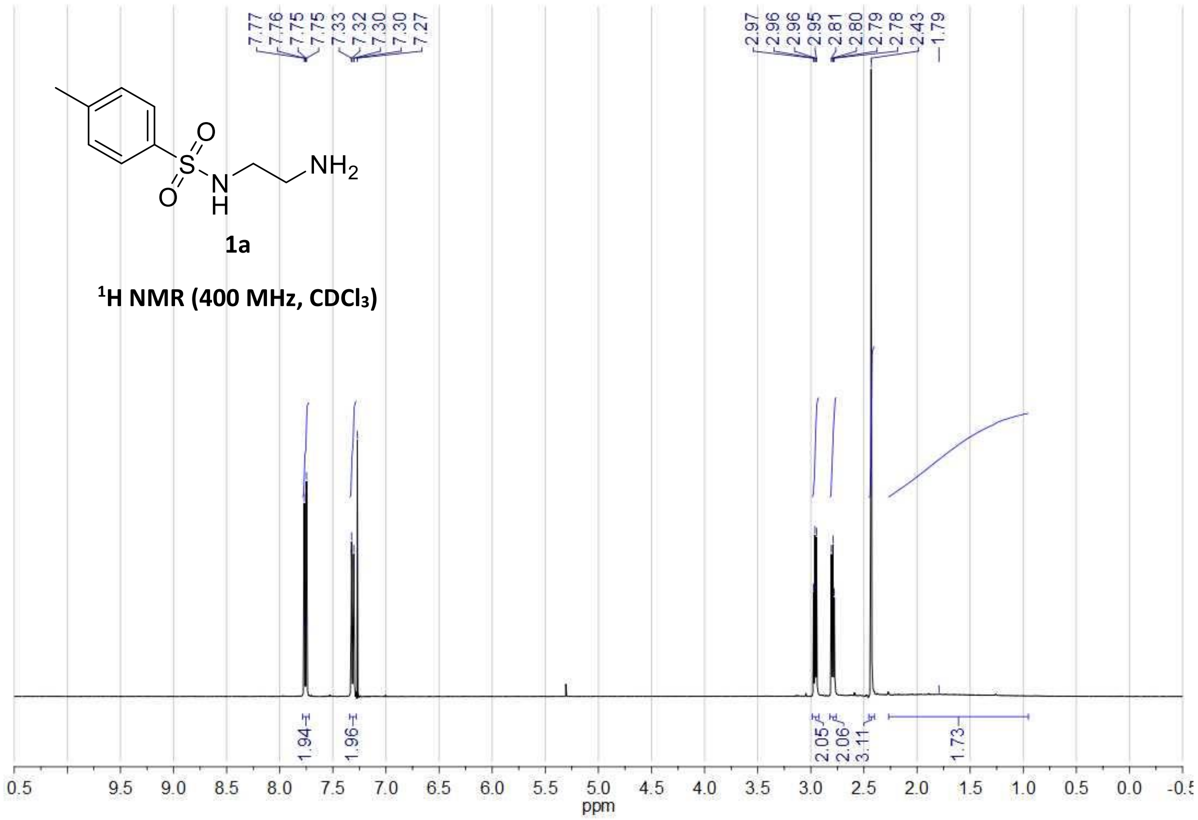
7.77
7.76
7.75
7.75
7.33
7.32
7.30
7.30
7.27
2.97
2.96
2.96
2.95
2.81
2.80
2.79
2.78
2.43
1.79
O
S
O
N
H
NH2
1a
1H NMR (400 MHz, CDCl3)
1.94
1.96
2.05
2.06
3.11
1.73
ppm

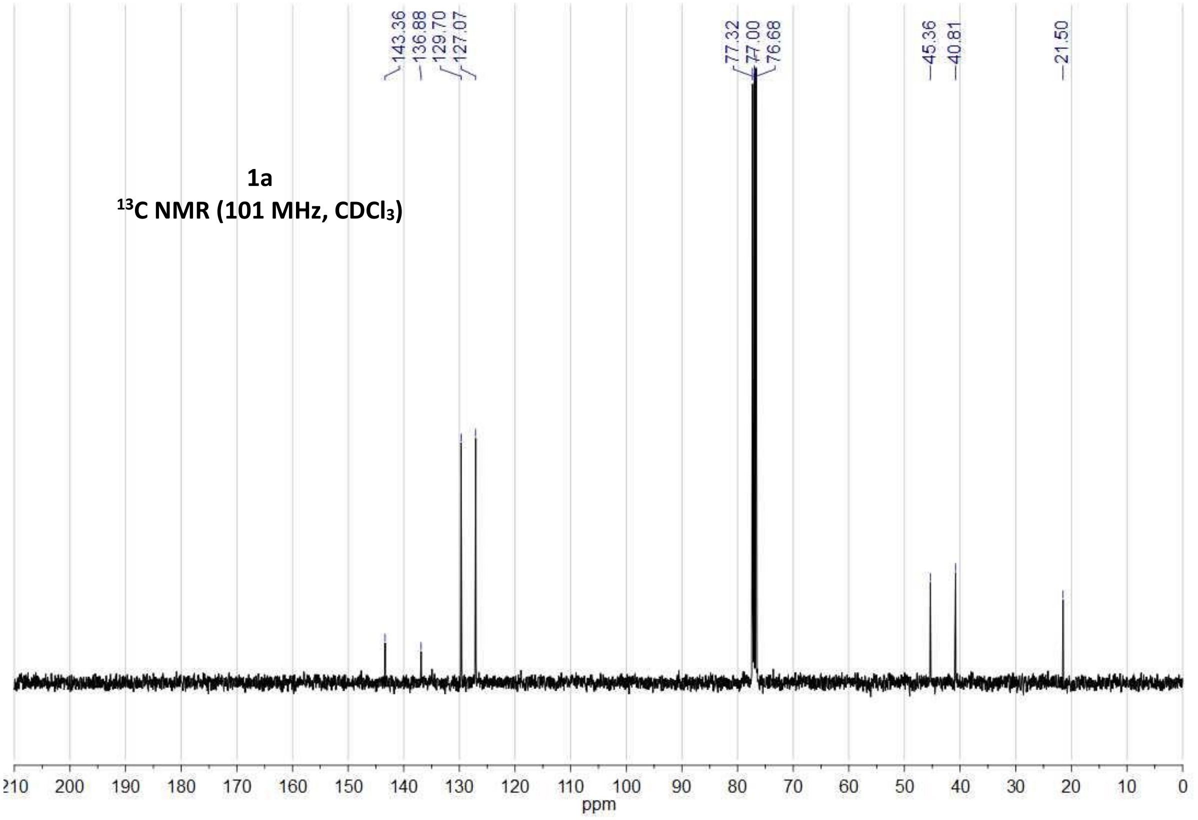
143.36
136.88
129.70
127.07
77.32
77.00
76.68
45.36
40.81
21.50
1a
13C NMR (101 MHz, CDCl3)
ppm

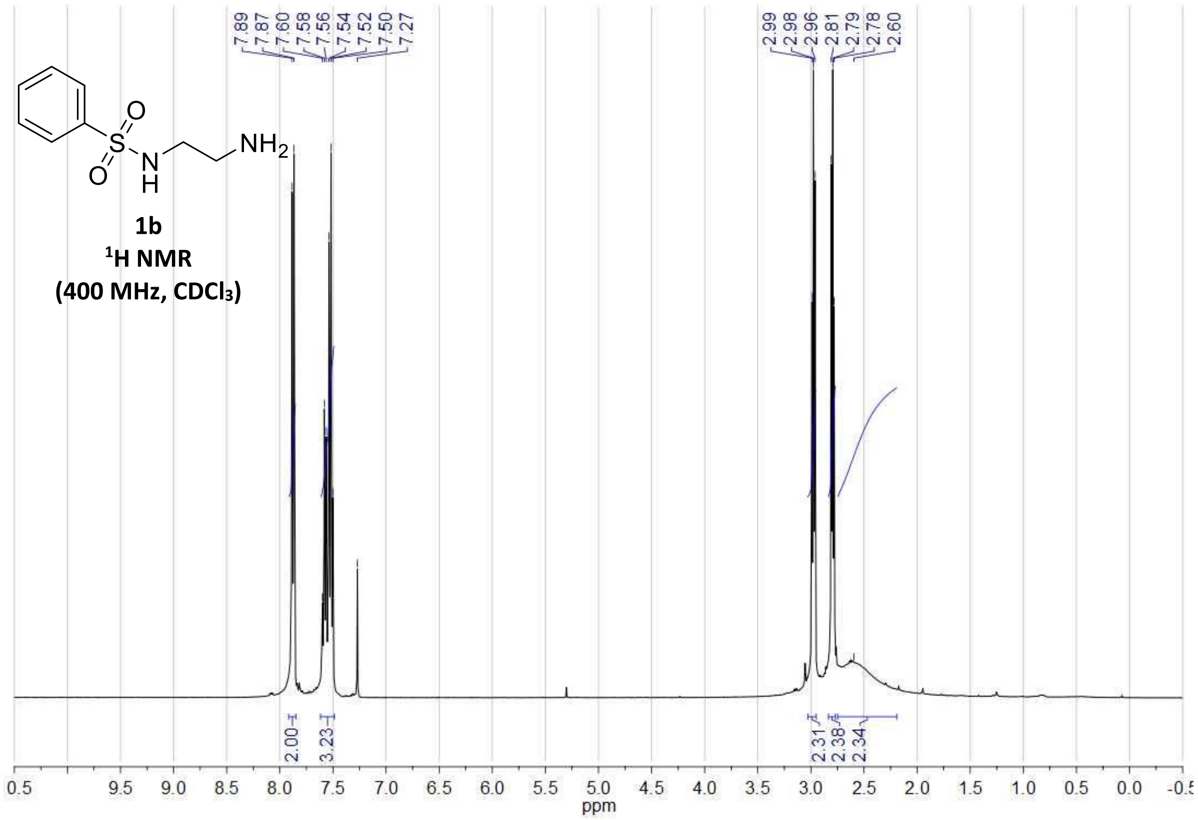
7.89
7.87
7.60
7.58
7.56
7.54
7.52
7.50
7.27
2.99
2.98
2.96
2.81
2.79
2.78
2.60
O
S
O
N
H
NH2
1b
1H NMR
(400 MHz, CDCl3)
2.00
3.23
2.31
2.38
2.34
ppm

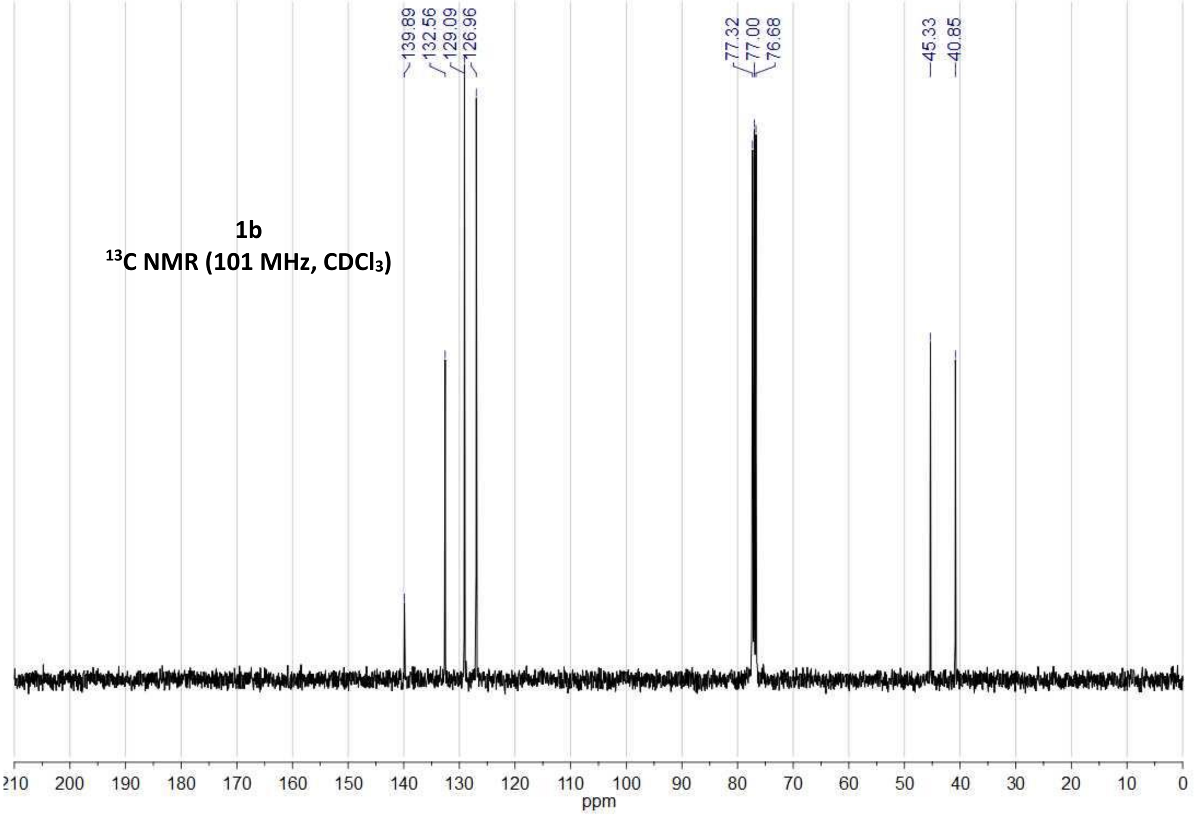
139.89
132.56
129.09
126.96
77.32
77.00
76.68
45.33
40.85
1b
13C NMR (101 MHz, CDCl3)
ppm

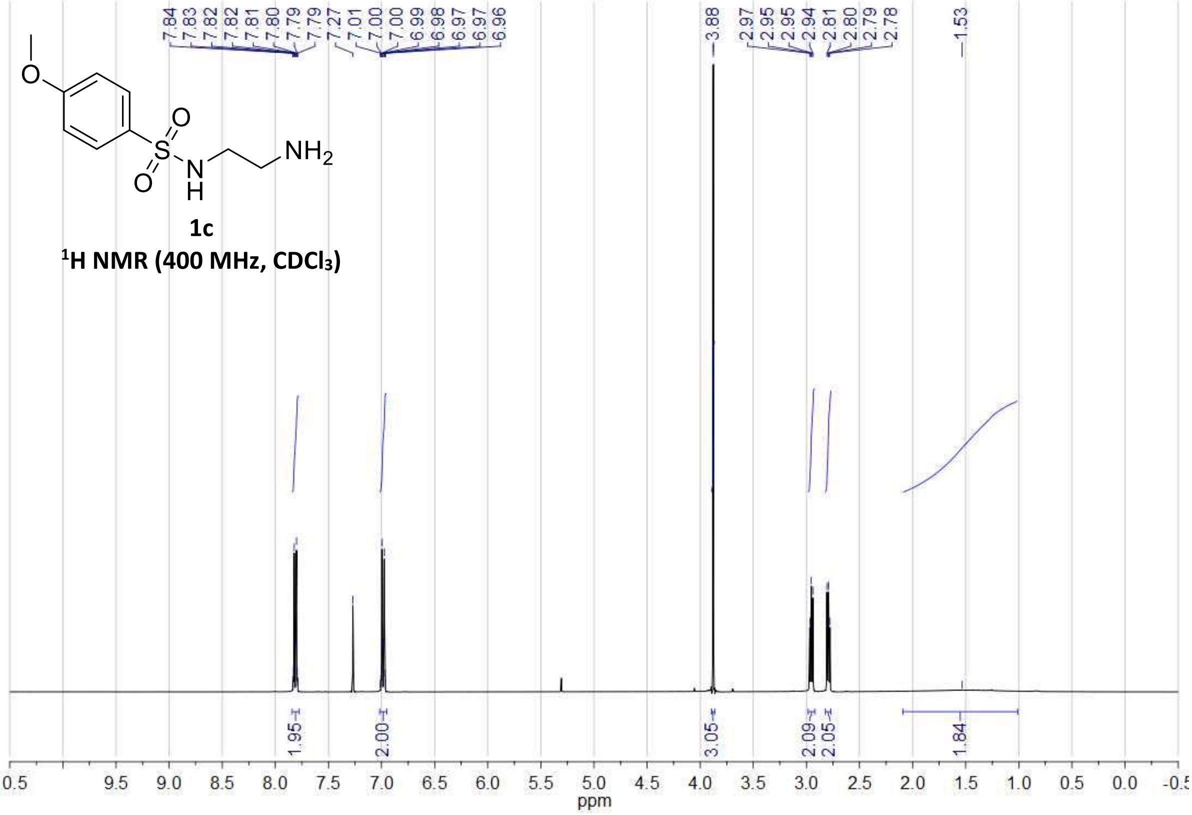
1c
^{1}H NMR (400 MHz, $CDCl_3$)
7.84 7.83 7.82 7.82 7.81 7.80 7.79 7.79 7.27 7.01 7.00 7.00 6.99 6.98 6.97 6.97 6.96
3.88
2.97 2.95 2.95 2.94 2.81 2.80 2.79 2.78
1.53
1.95 2.00 3.05 2.09 2.05 1.84
ppm

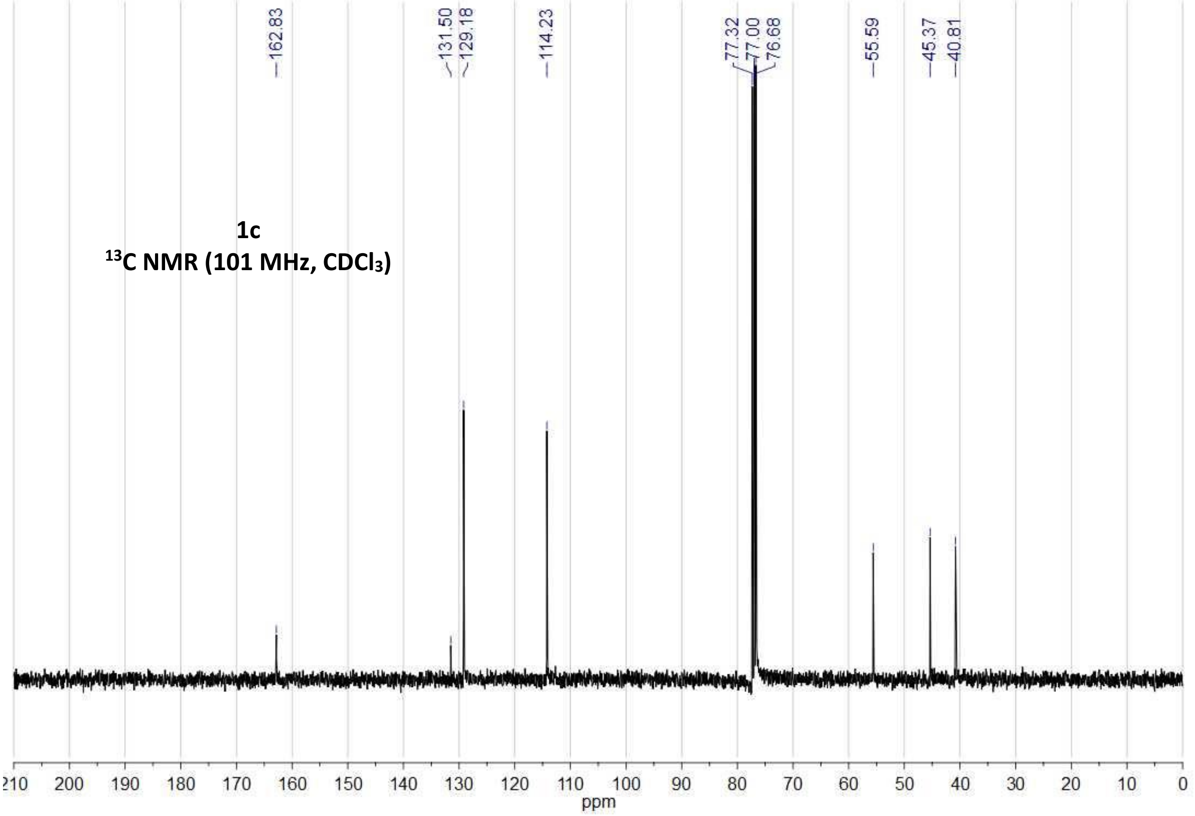
1c
^{13}C NMR (101 MHz, $CDCl_3$)
162.83
131.50 129.18
114.23
77.32 77.00 76.68
55.59
45.37 40.81
ppm

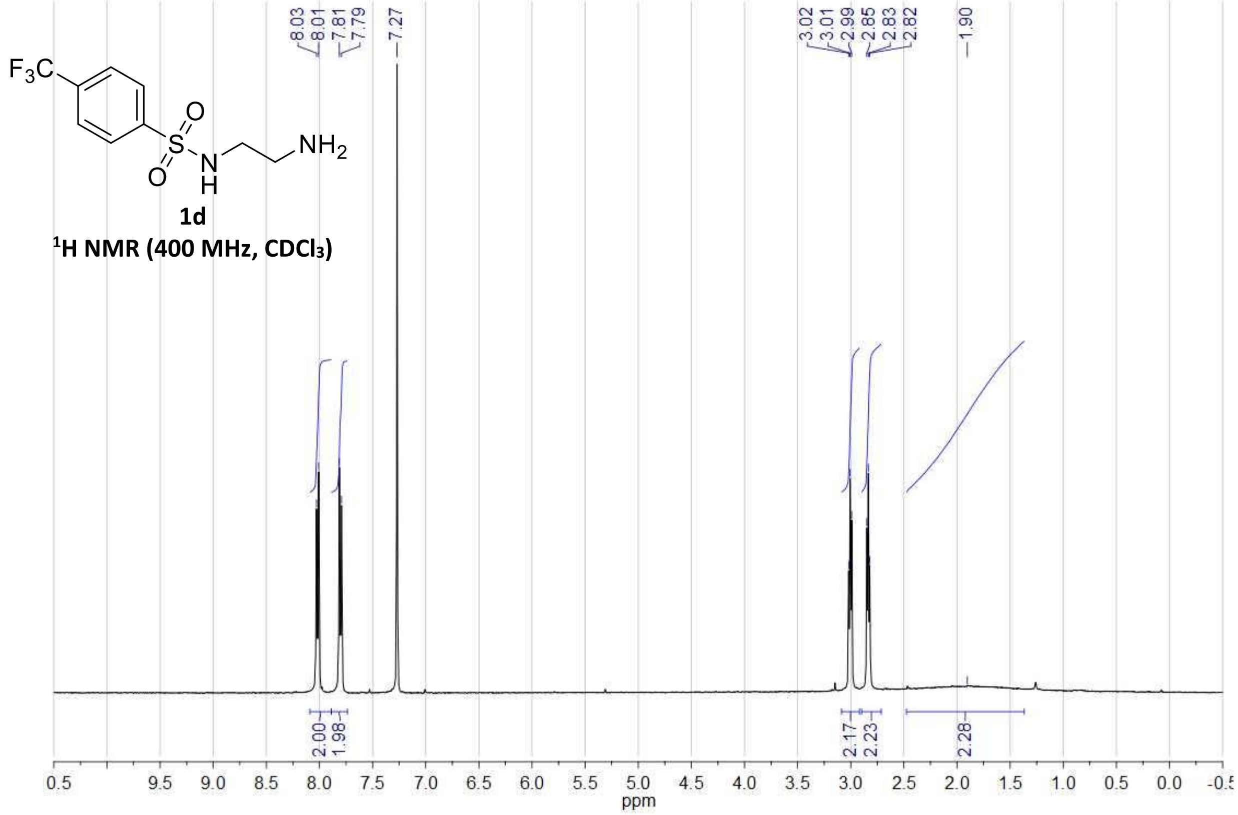

1d

^{1}H NMR (400 MHz, $CDCl_3$)

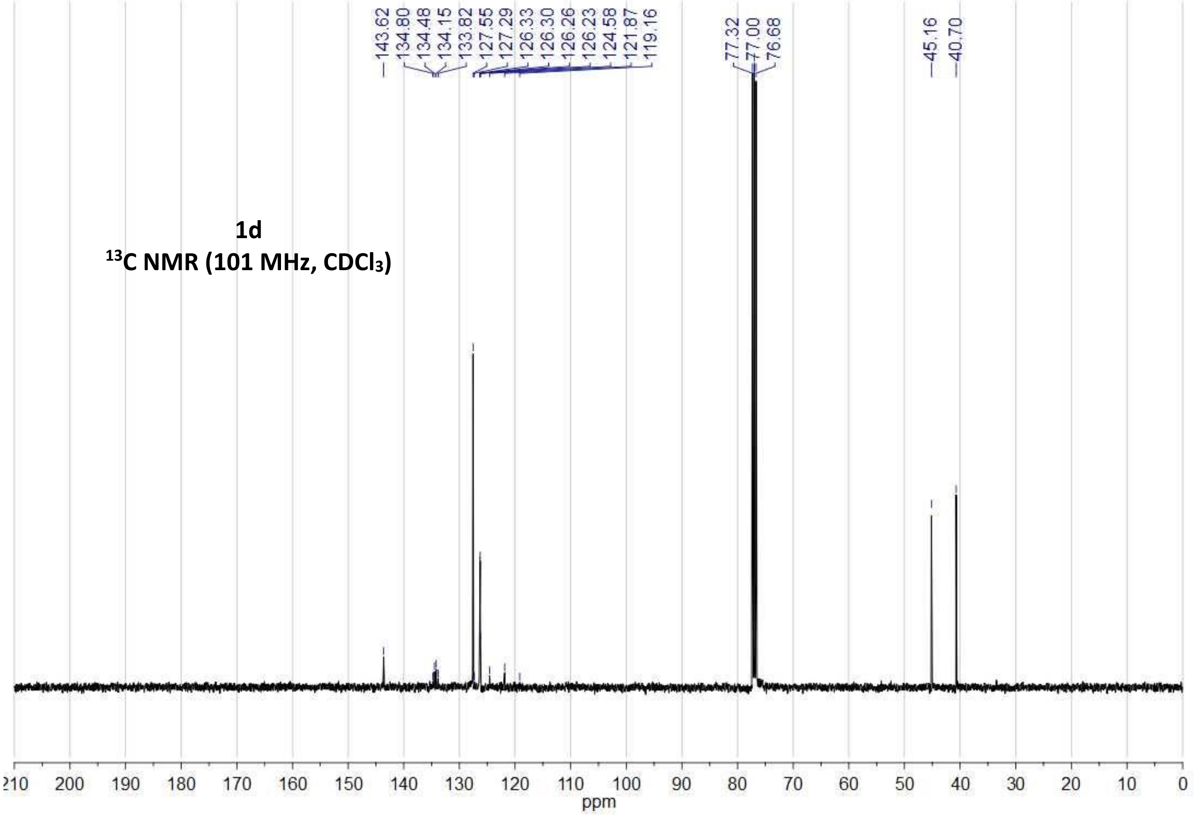

1d

^{13}C NMR (101 MHz, $CDCl_3$)

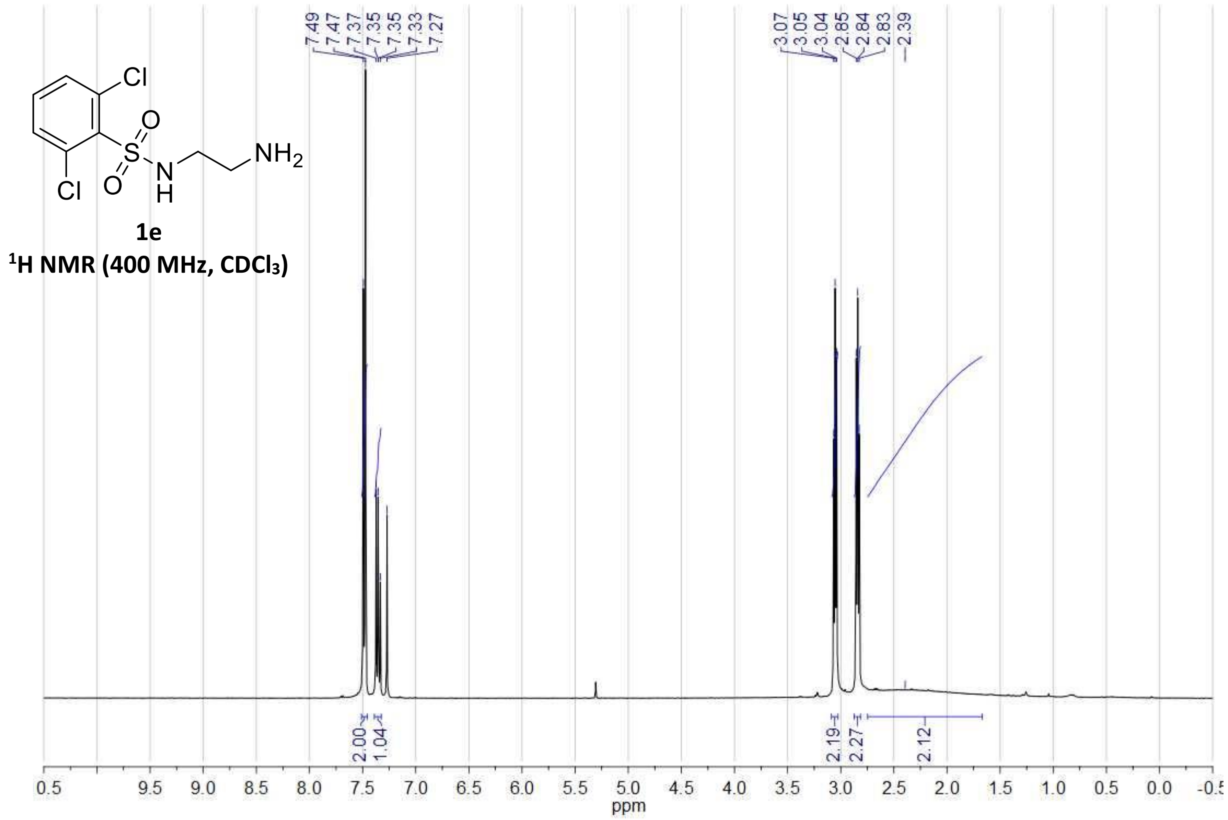

1e
^{1}H NMR (400 MHz, CDCl$_3$)

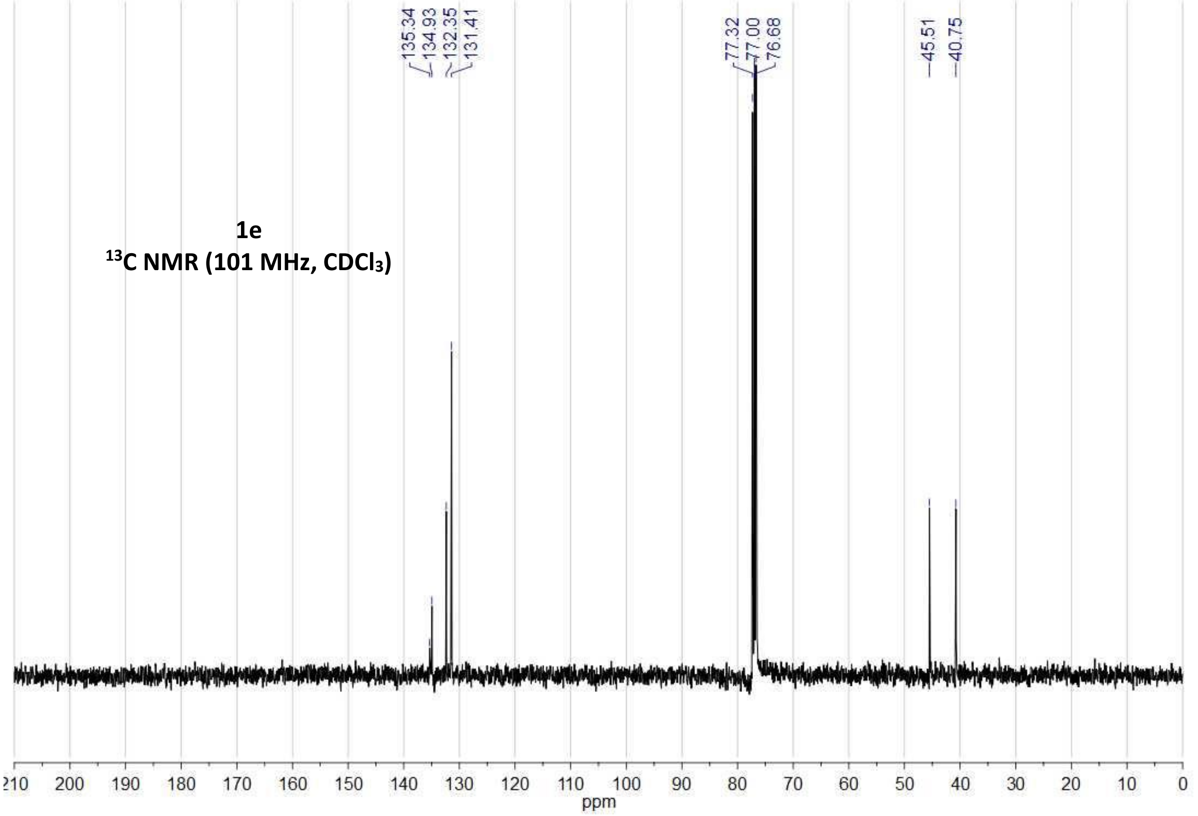

1e
^{13}C NMR (101 MHz, CDCl$_3$)

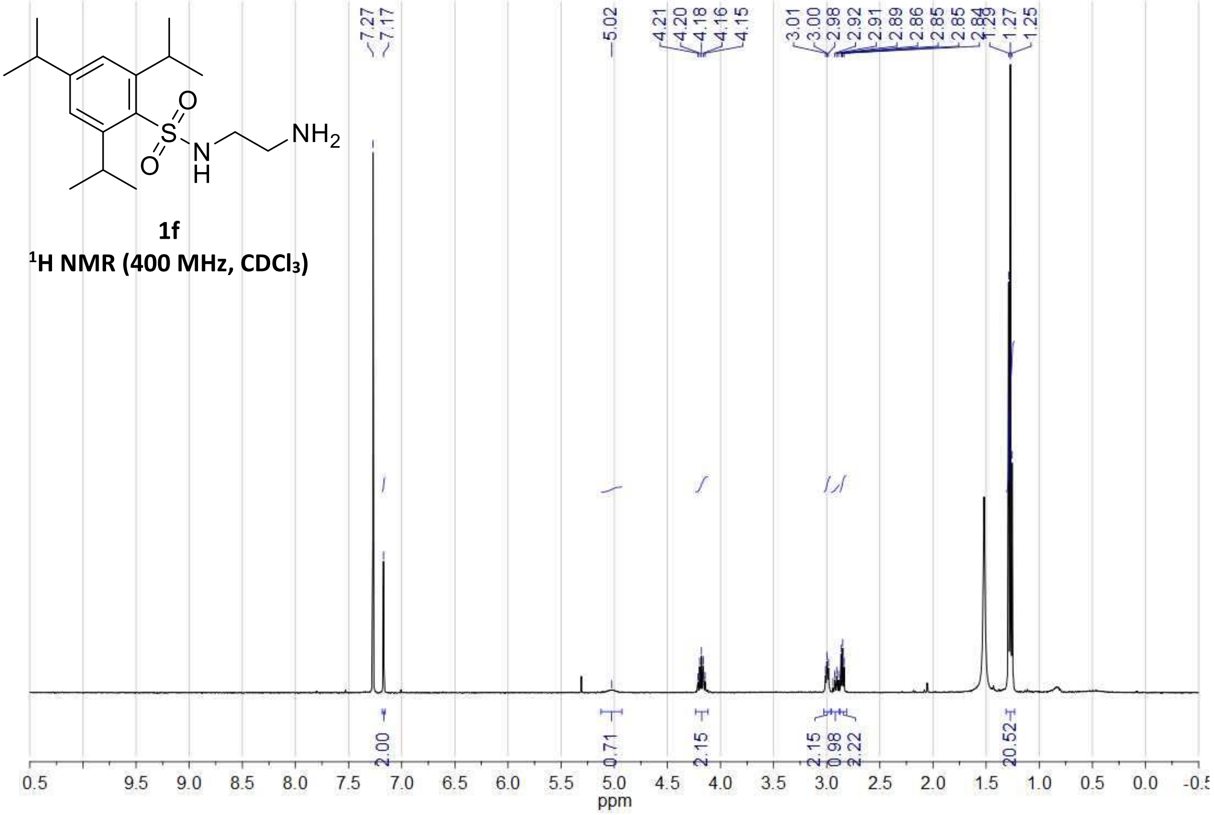

1f
^{1}H NMR (400 MHz, CDCl$_3$)

1f
^{13}C NMR (101 MHz, CDCl$_3$)

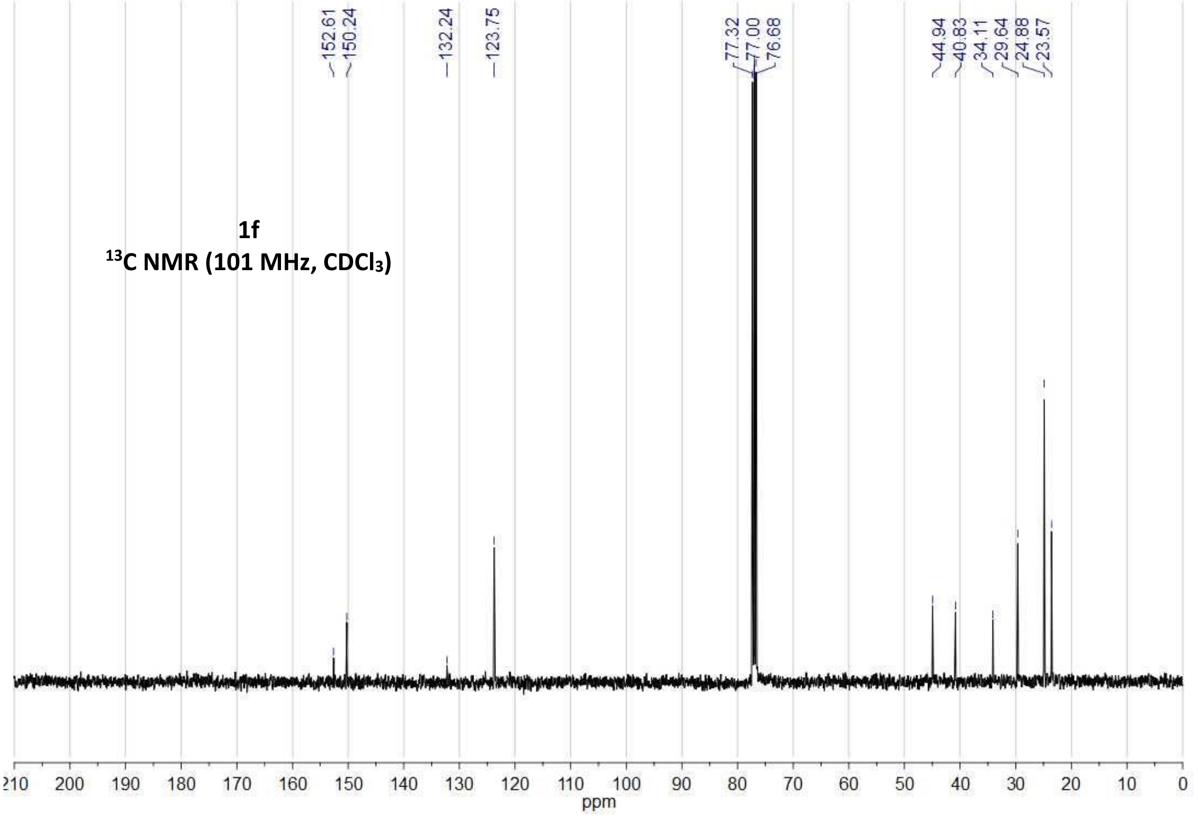

1g

^{1}H NMR (400 MHz, $CDCl_3$)

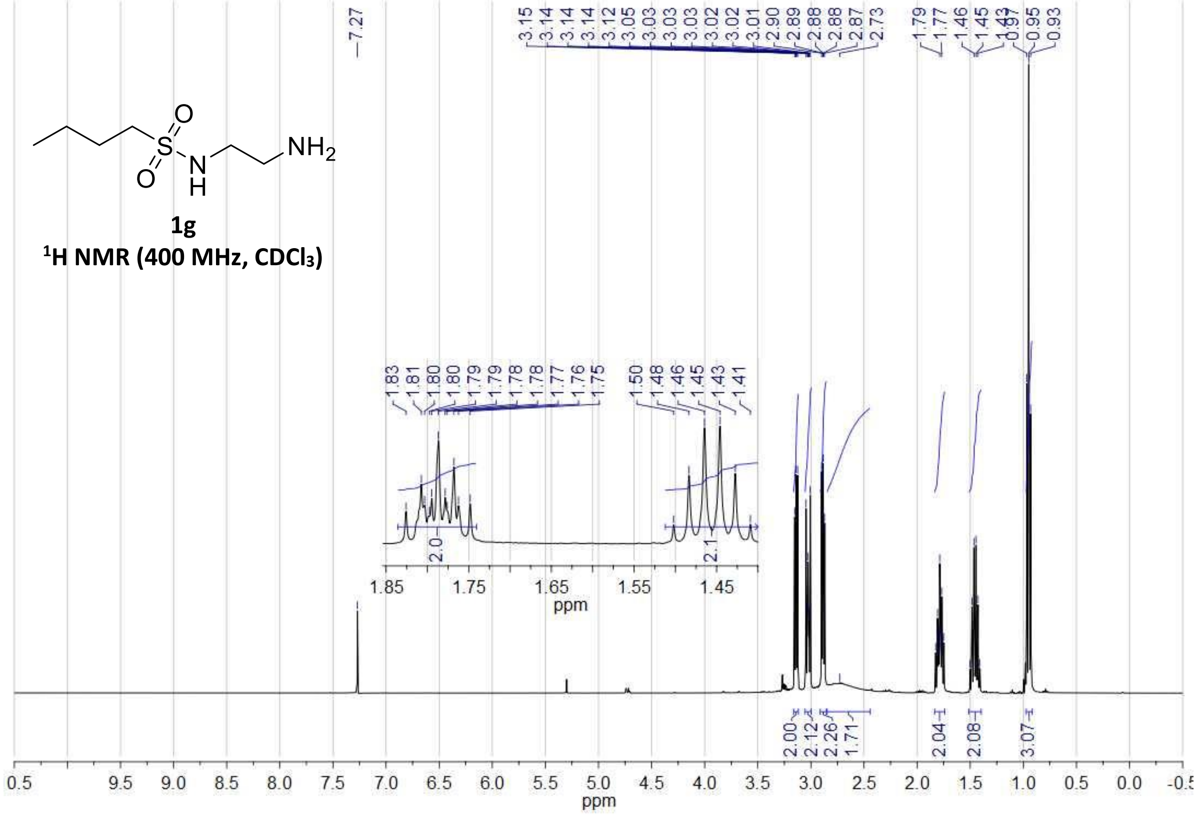

1g

^{13}C NMR (101 MHz, $CDCl_3$)

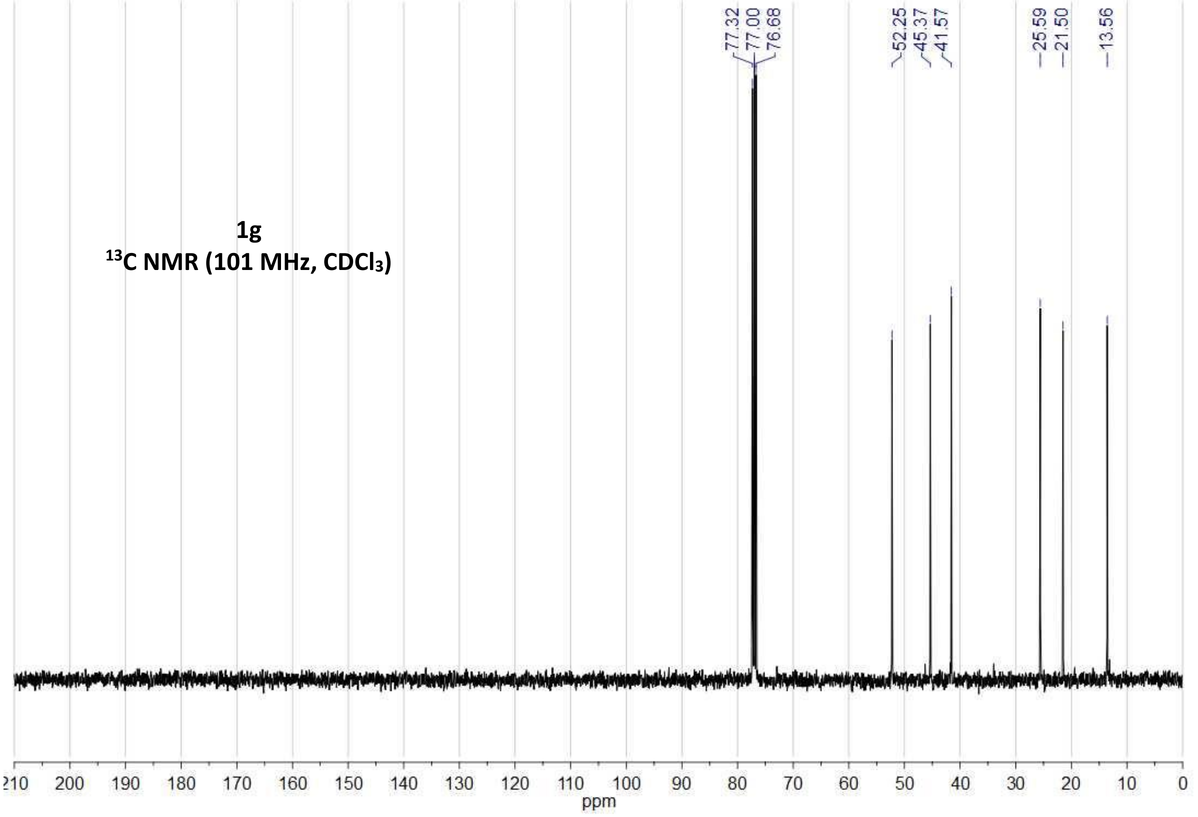

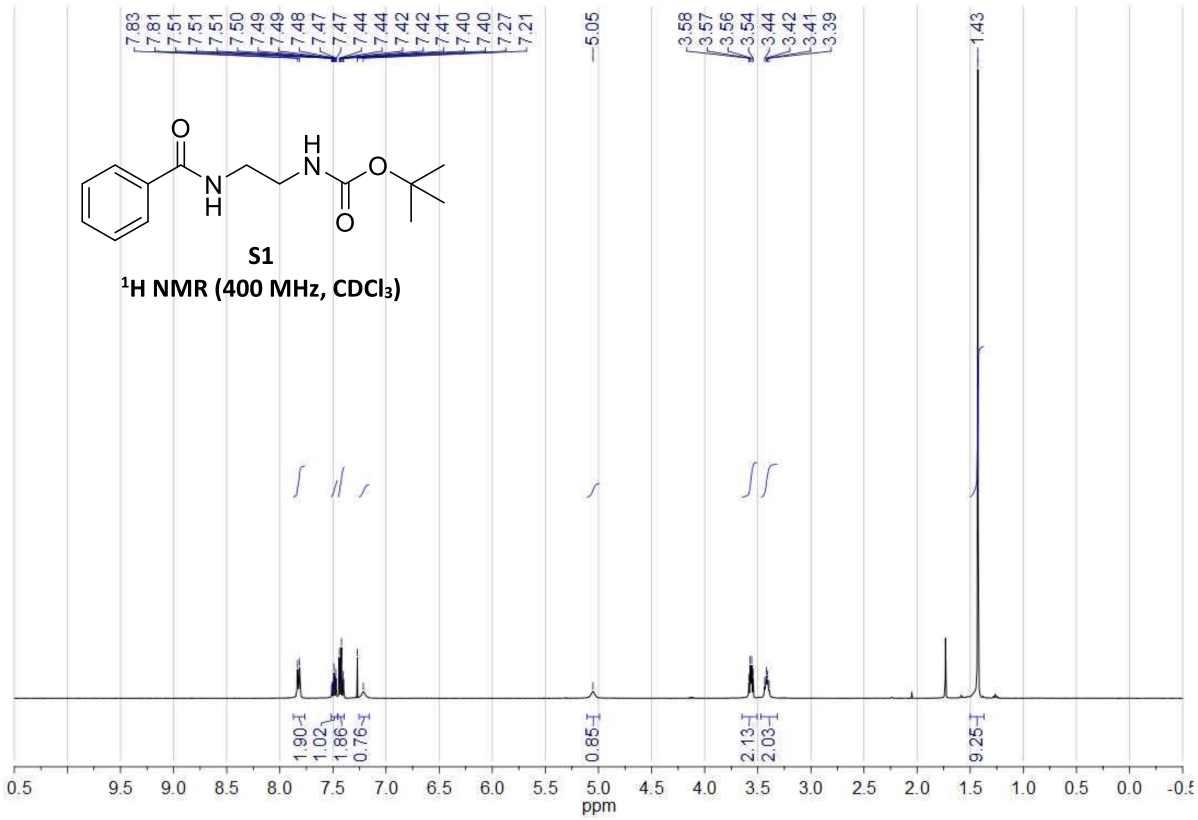
S1
^{1}H NMR (400 MHz, CDCl$_3$)
7.83 7.81 7.51 7.51 7.51 7.50 7.49 7.49 7.48 7.47 7.47 7.44 7.44 7.42 7.42 7.41 7.40 7.40 7.27 7.21
5.05
3.58 3.57 3.56 3.54 3.44 3.42 3.41 3.39
1.43
1.90 1.02 1.86 0.76 0.85 2.13 2.03 9.25
ppm

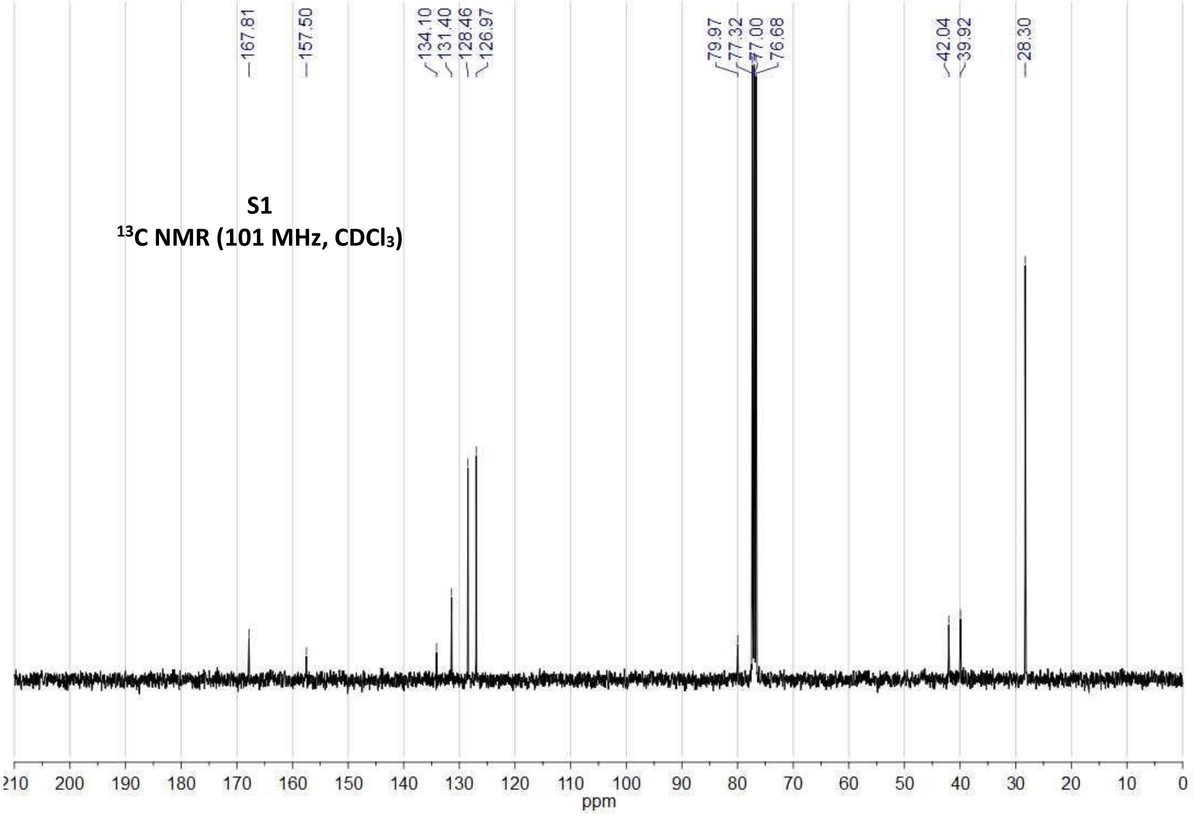
S1
^{13}C NMR (101 MHz, CDCl$_3$)
167.81
157.50
134.10 131.40 128.46 126.97
79.97 77.32 77.00 76.68
42.04 39.92
28.30
ppm

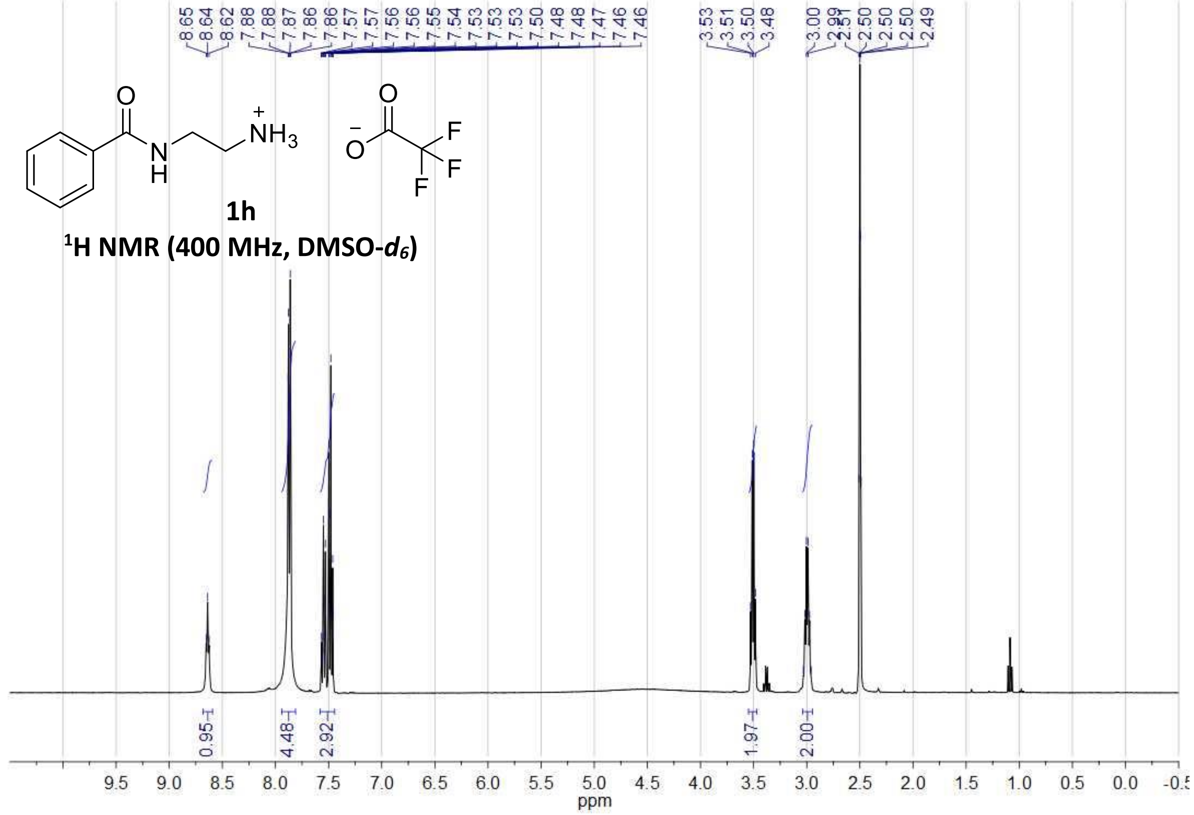
8.65 8.64 8.62 7.88 7.88 7.87 7.86 7.86 7.57 7.57 7.56 7.56 7.55 7.54 7.53 7.53 7.53 7.50 7.48 7.48 7.47 7.46 7.46
3.53 3.51 3.50 3.48
3.00 2.99 2.51 2.50 2.50 2.50 2.49
1h
1H NMR (400 MHz, DMSO-d6)
0.95
4.48
2.92
1.97
2.00
ppm

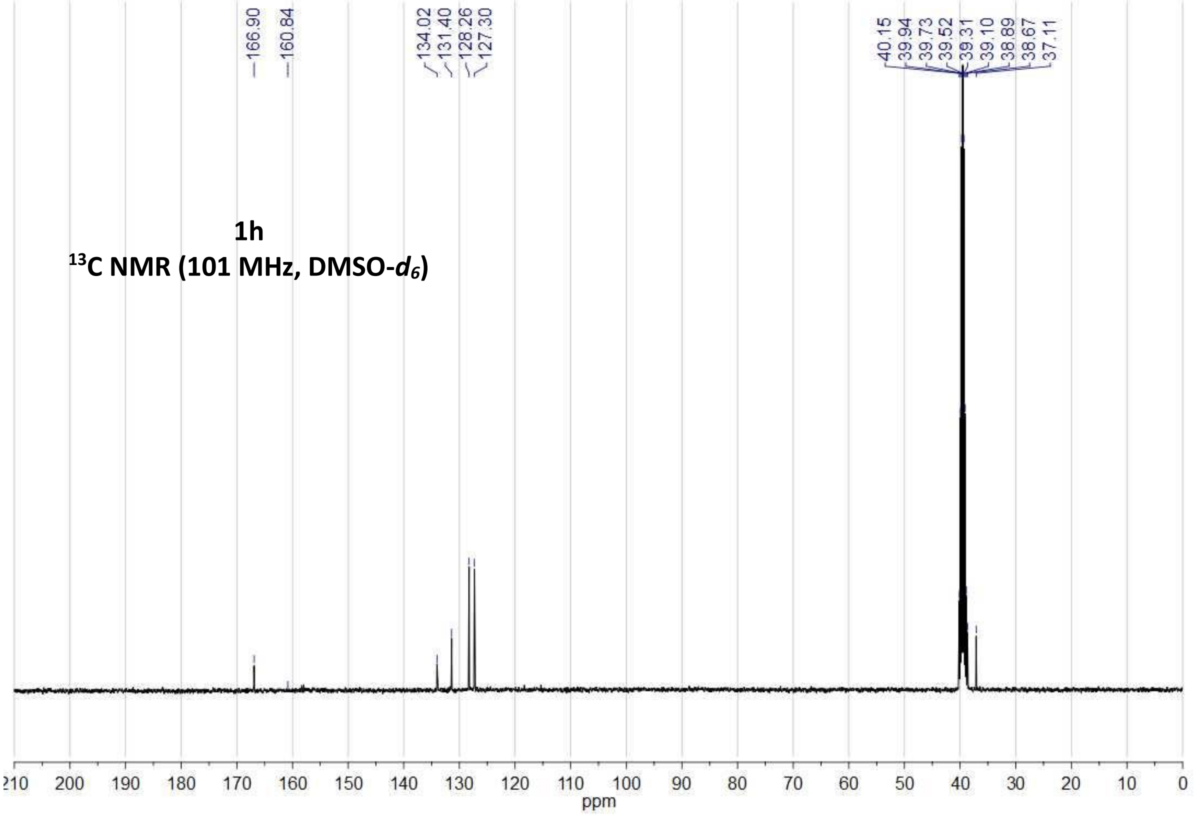
166.90
160.84
134.02 131.40 128.26 127.30
40.15 39.94 39.73 39.52 39.31 39.10 38.89 38.67 37.11
1h
13C NMR (101 MHz, DMSO-d6)
ppm

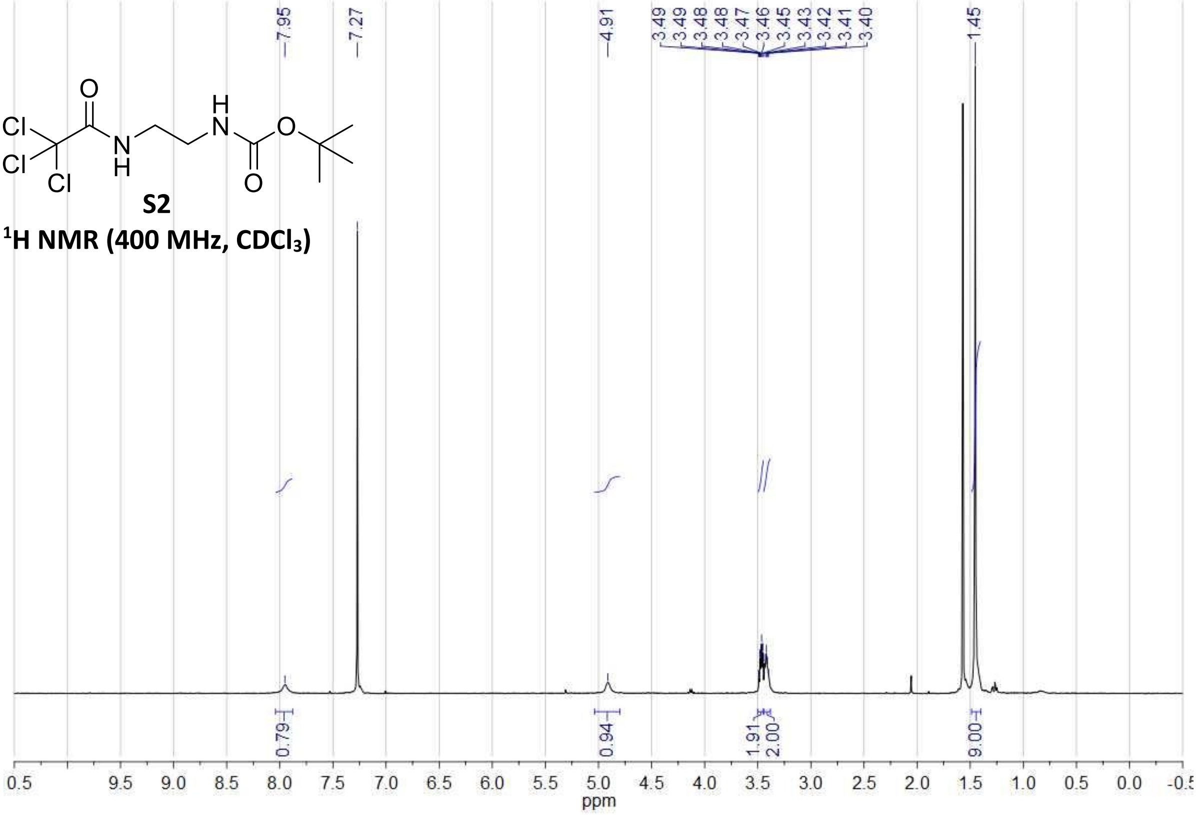
7.95
7.27
4.91
3.49
3.49
3.48
3.47
3.46
3.45
3.43
3.42
3.41
3.40
1.45
Cl
Cl
Cl
O
N
H
H
N
O
O
S2
¹H NMR (400 MHz, CDCl₃)
0.79
0.94
1.91
2.00
9.00
ppm

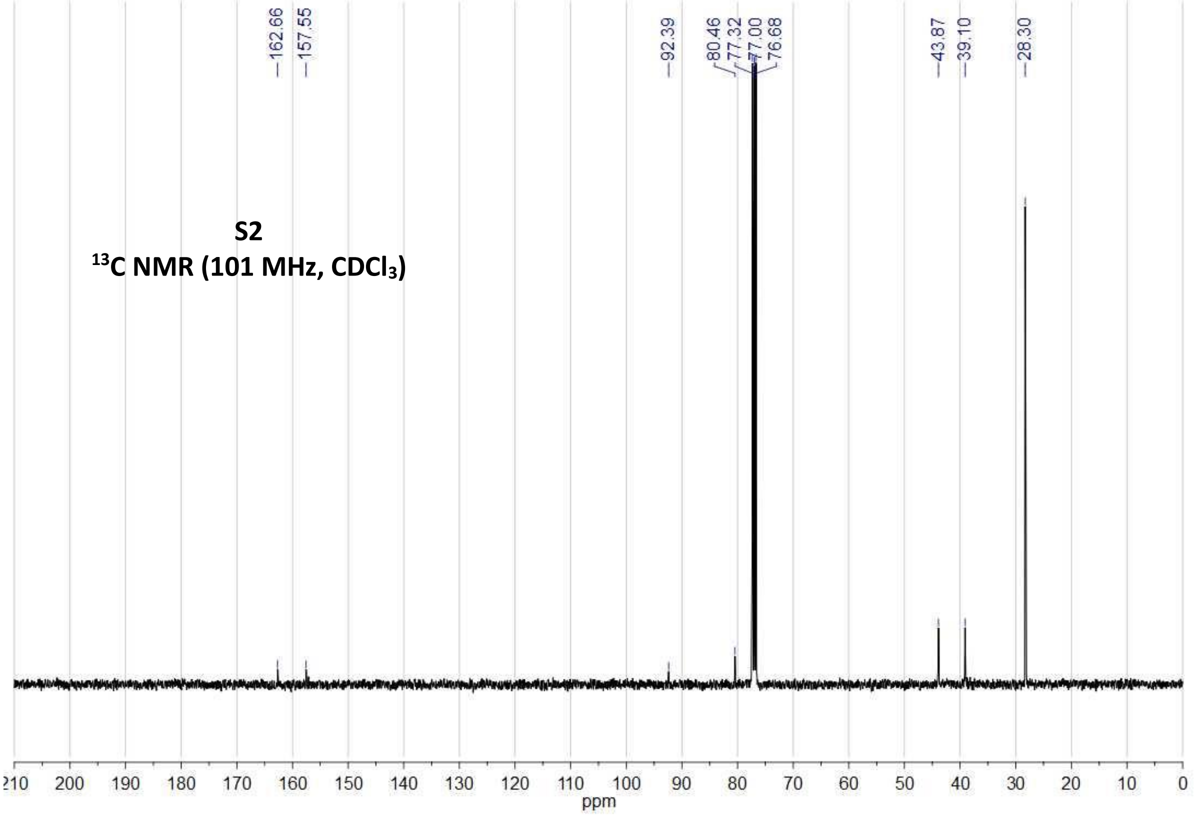
162.66
157.55
92.39
80.46
77.32
77.00
76.68
43.87
39.10
28.30
S2
¹³C NMR (101 MHz, CDCl₃)
ppm

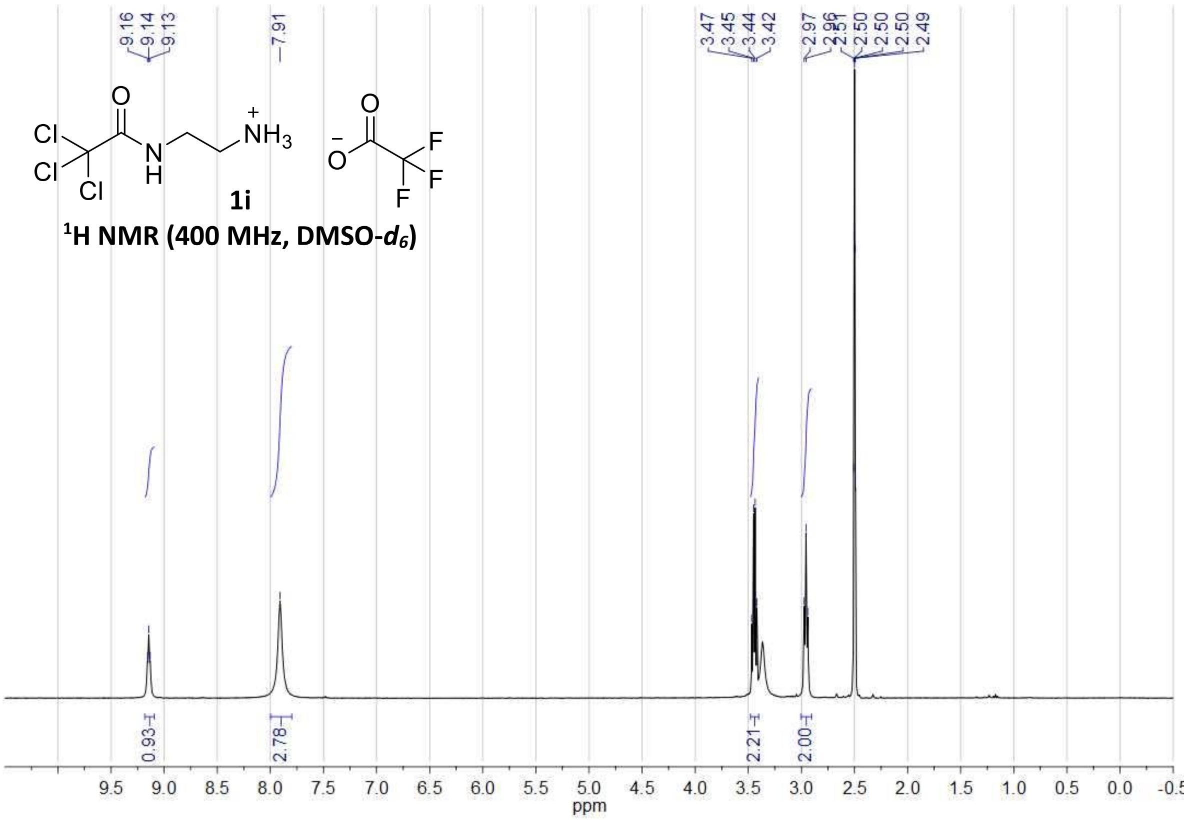

1i
^{1}H NMR (400 MHz, DMSO-*d*$_6$)

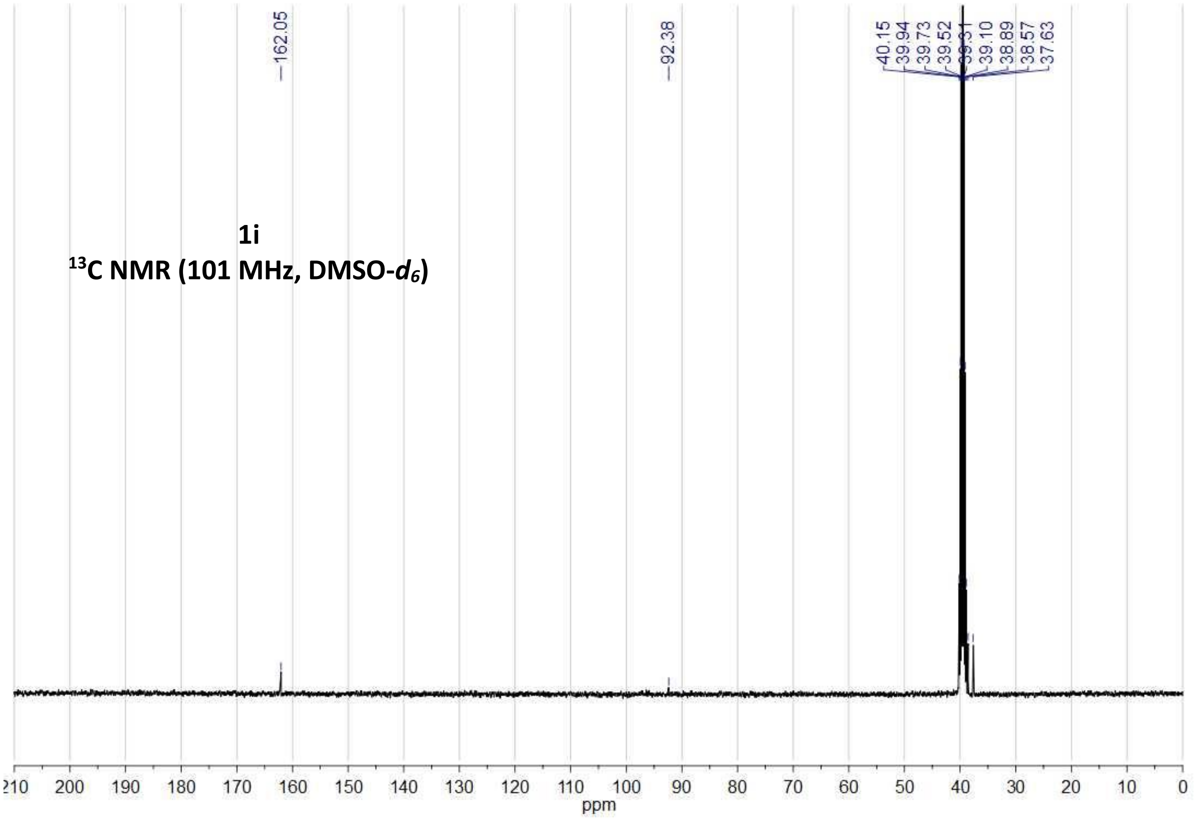

1i
^{13}C NMR (101 MHz, DMSO-*d*$_6$)

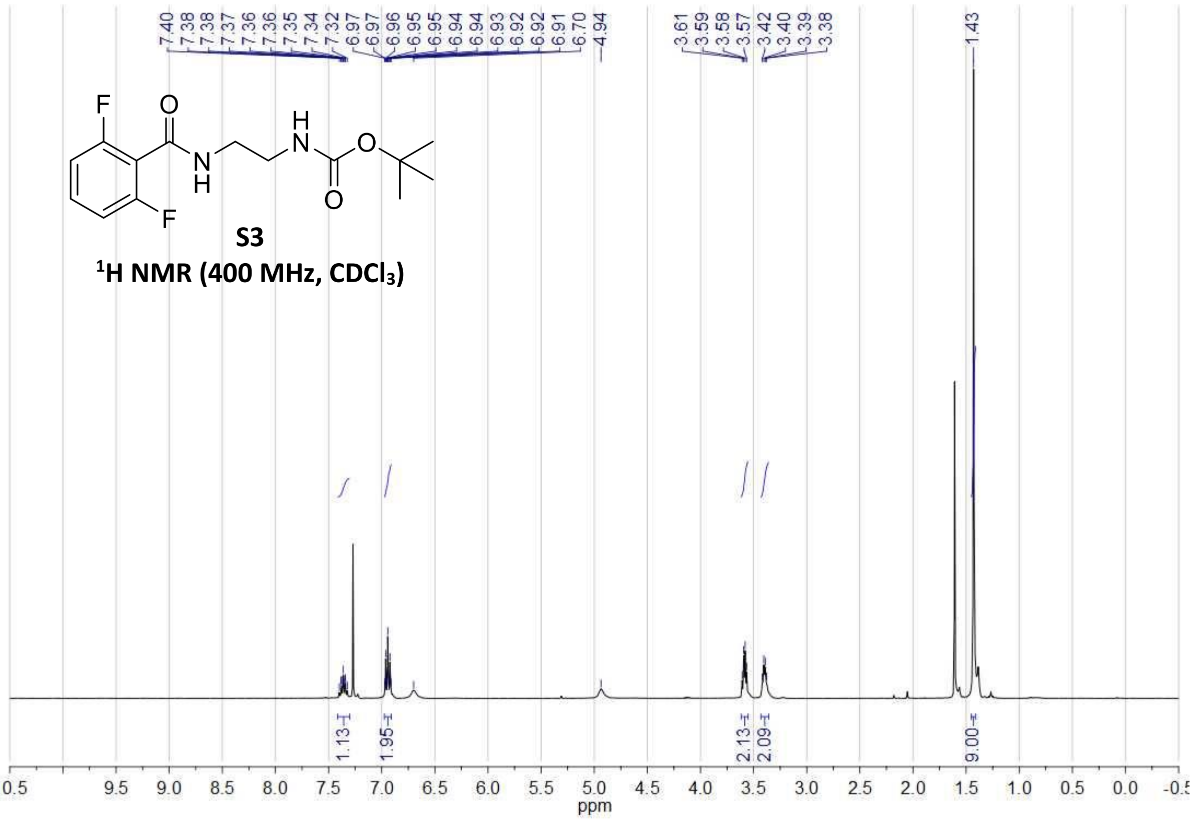

S3
^{1}H NMR (400 MHz, $CDCl_3$)

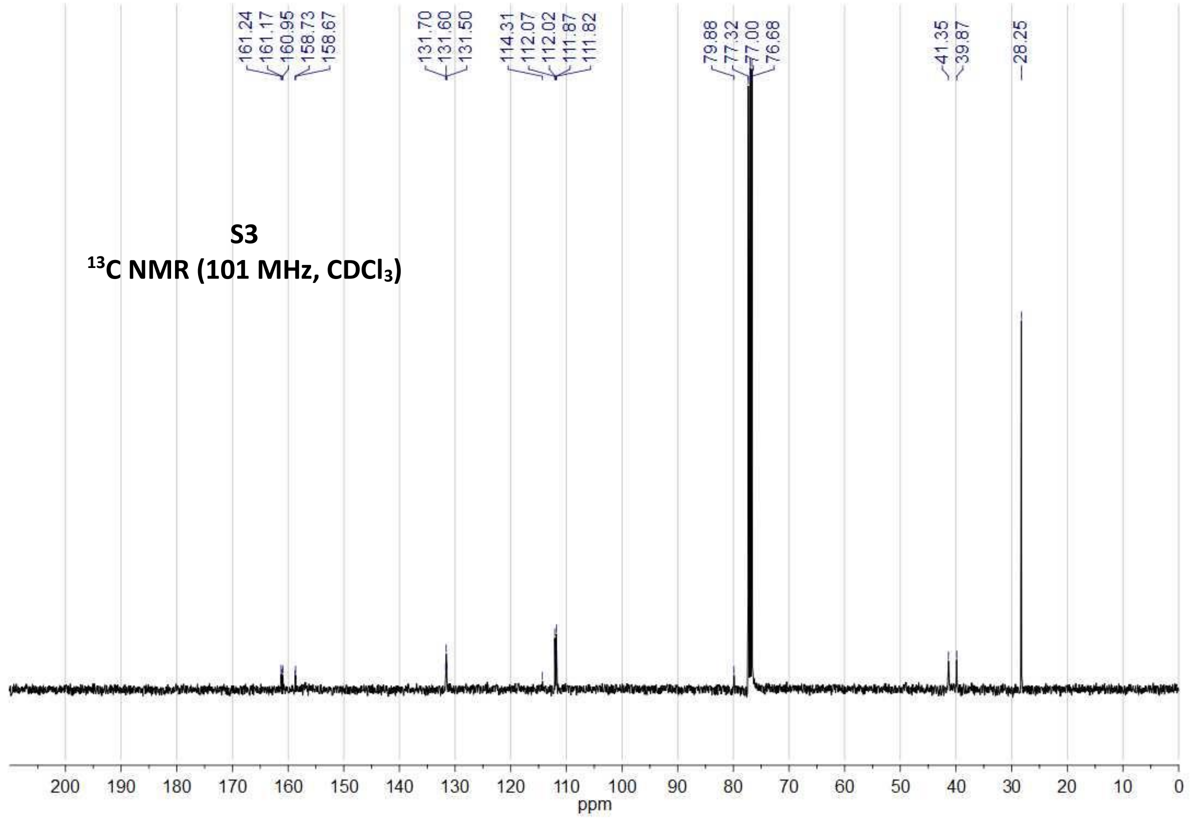

S3
^{13}C NMR (101 MHz, $CDCl_3$)

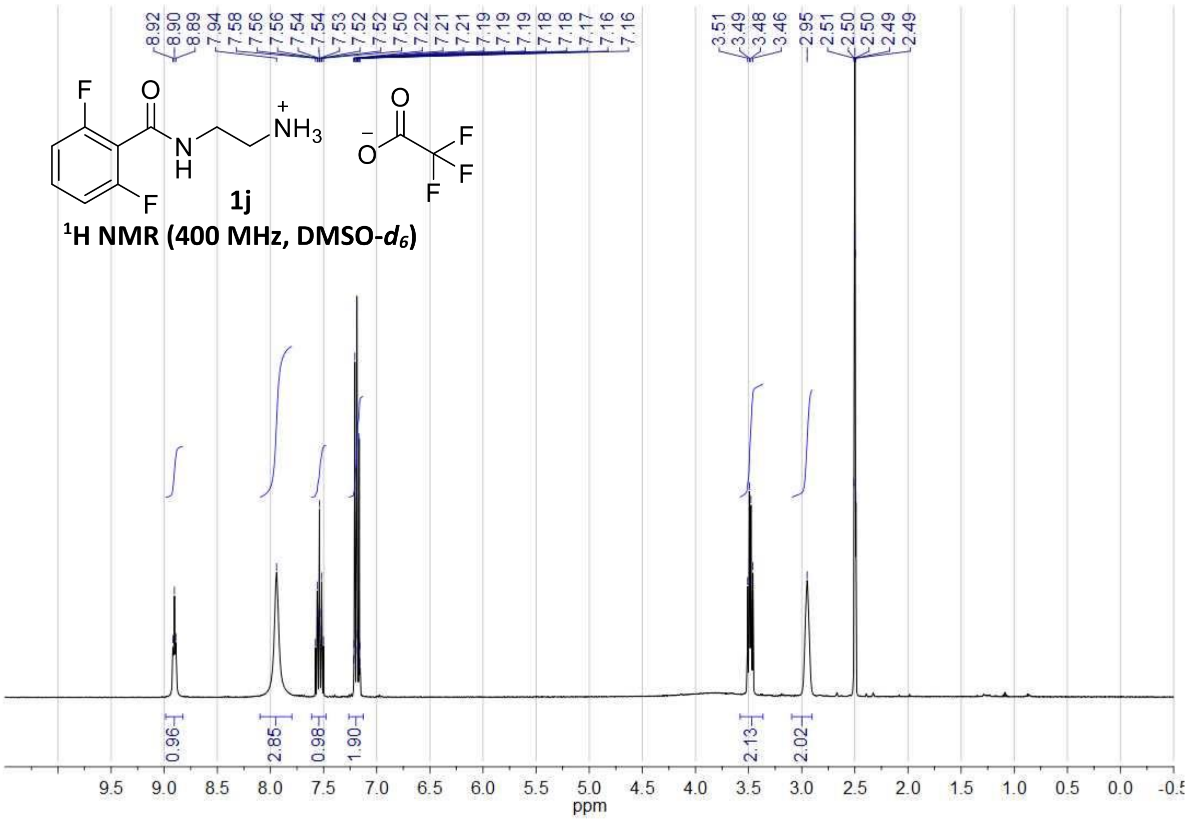
1j
^{1}H NMR (400 MHz, DMSO-d_6)
ppm

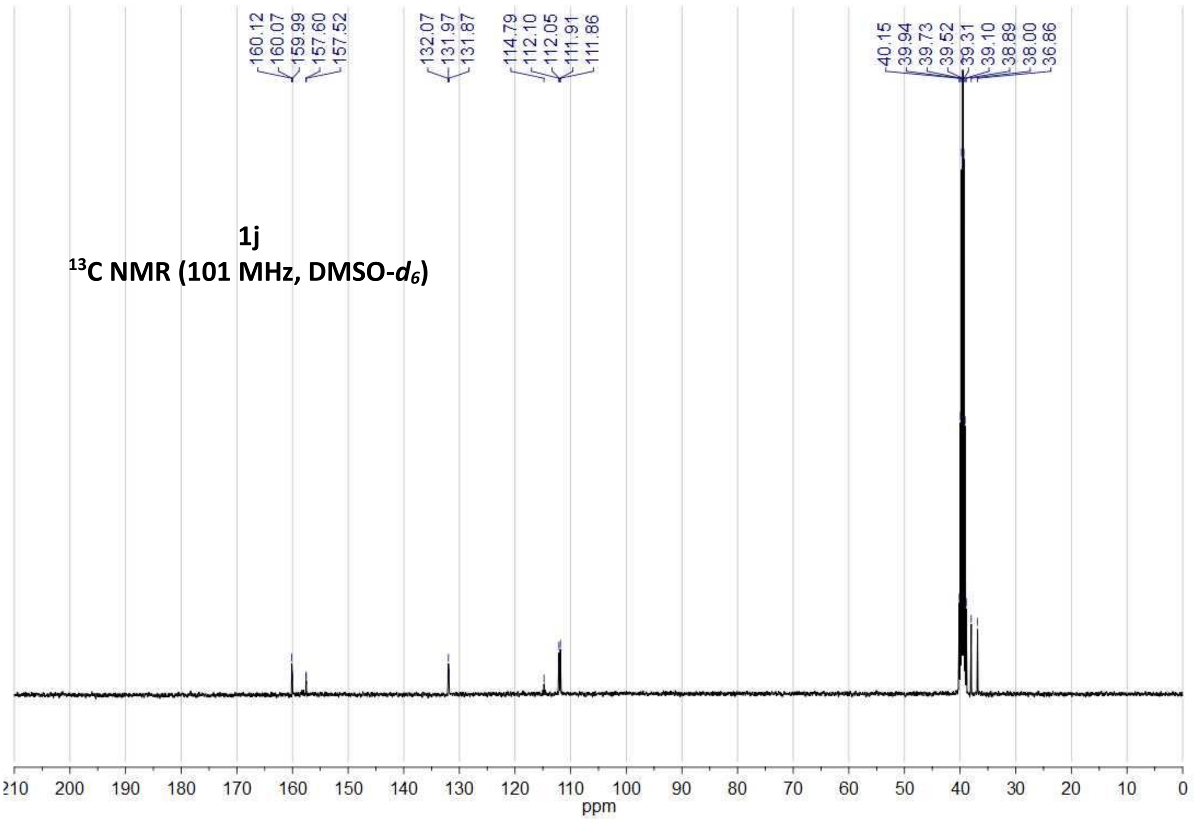
1j
^{13}C NMR (101 MHz, DMSO-d_6)
ppm

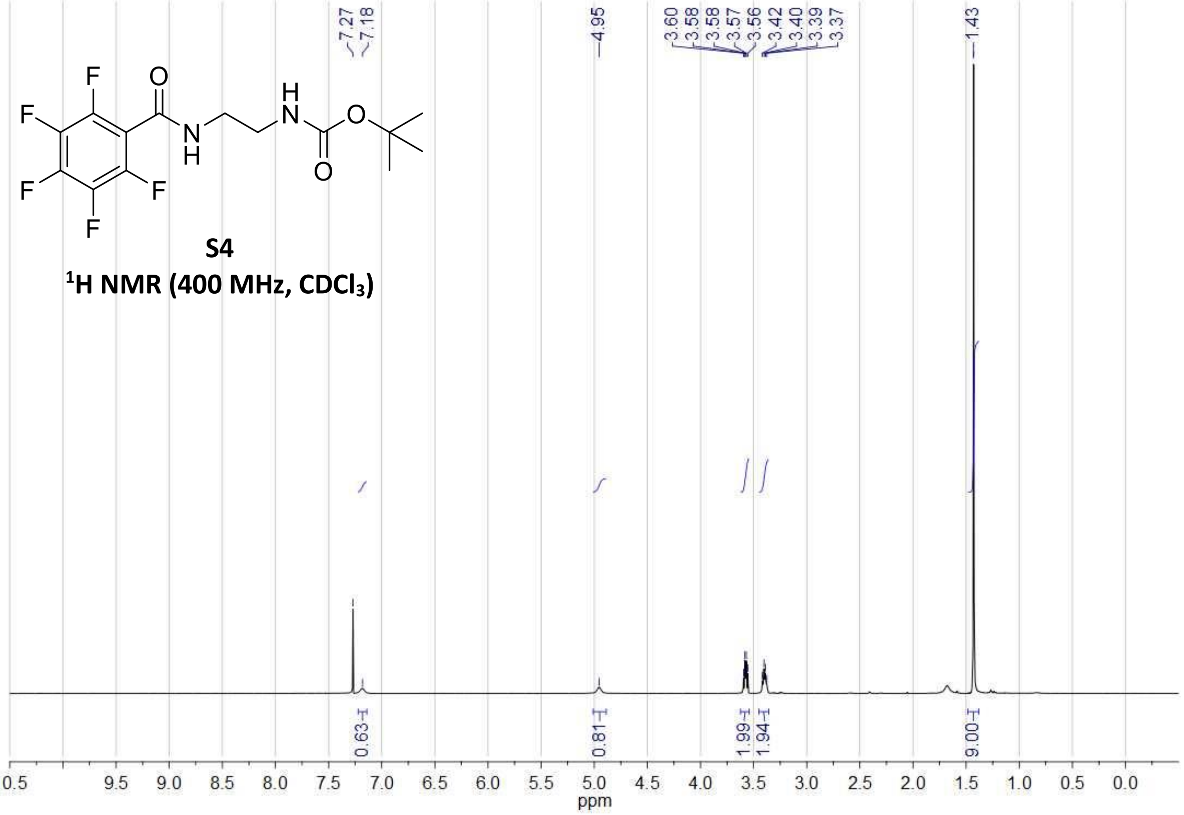
7.27
7.18
4.95
3.60
3.58
3.58
3.57
3.56
3.42
3.40
3.39
3.37
1.43
S4
1H NMR (400 MHz, CDCl3)
0.63
0.81
1.99
1.94
9.00
ppm

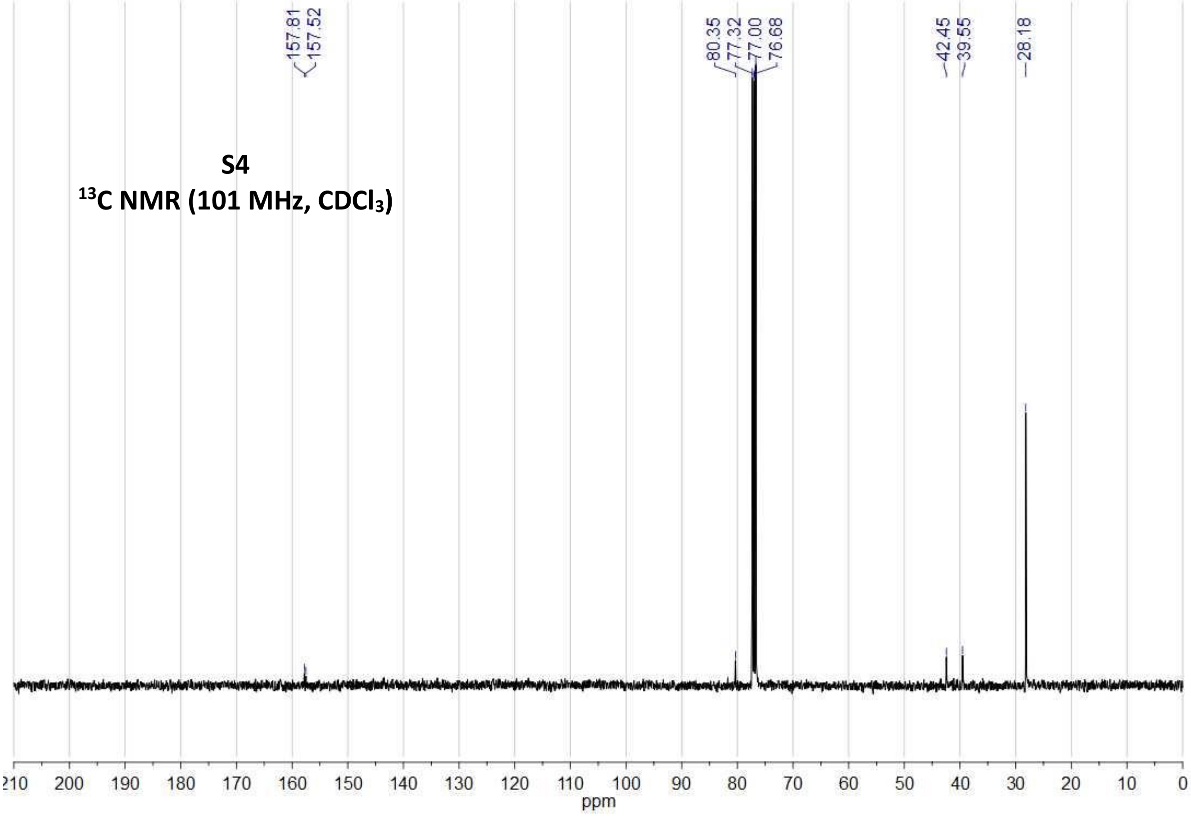
157.81
157.52
80.35
77.32
77.00
76.68
42.45
39.55
28.18
S4
13C NMR (101 MHz, CDCl3)
ppm

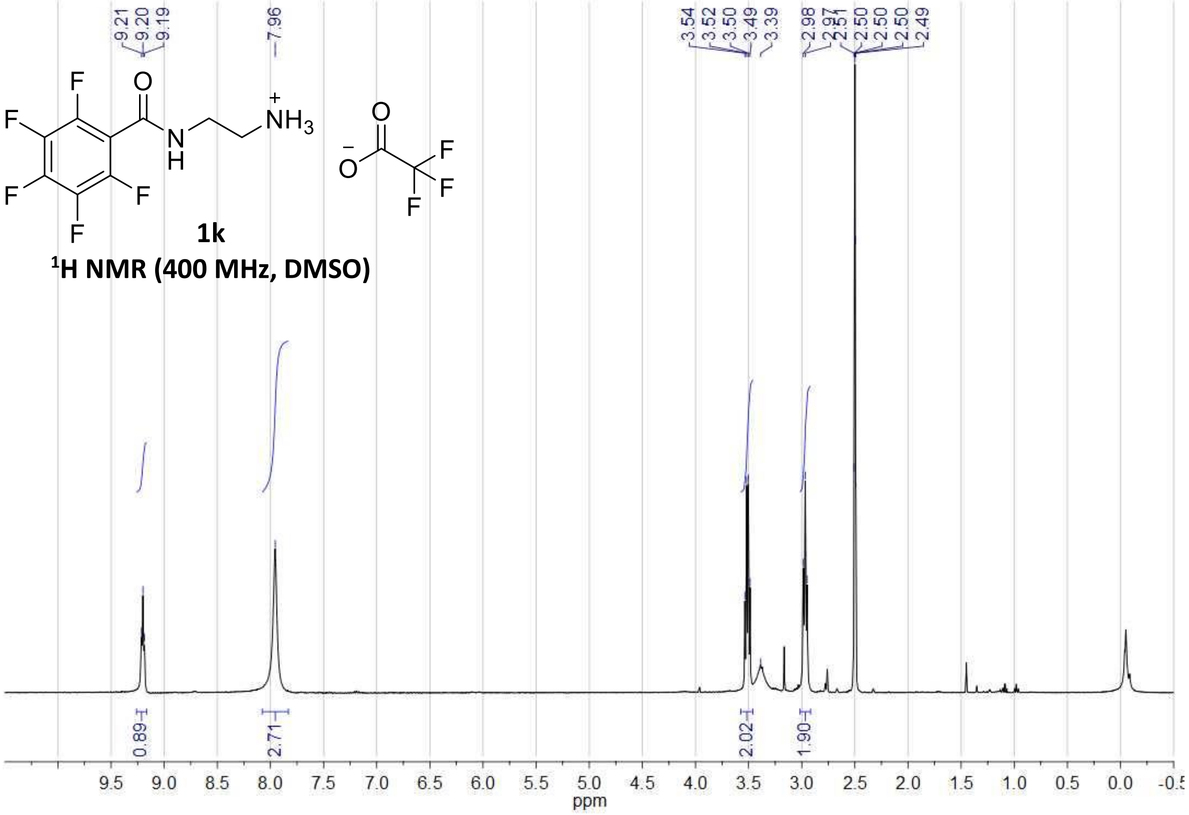
1k
1H NMR (400 MHz, DMSO)
9.21
9.20
9.19
7.96
3.54
3.52
3.50
3.49
3.39
2.98
2.97
2.51
2.50
2.50
2.50
2.49
0.89
2.71
2.02
1.90
ppm

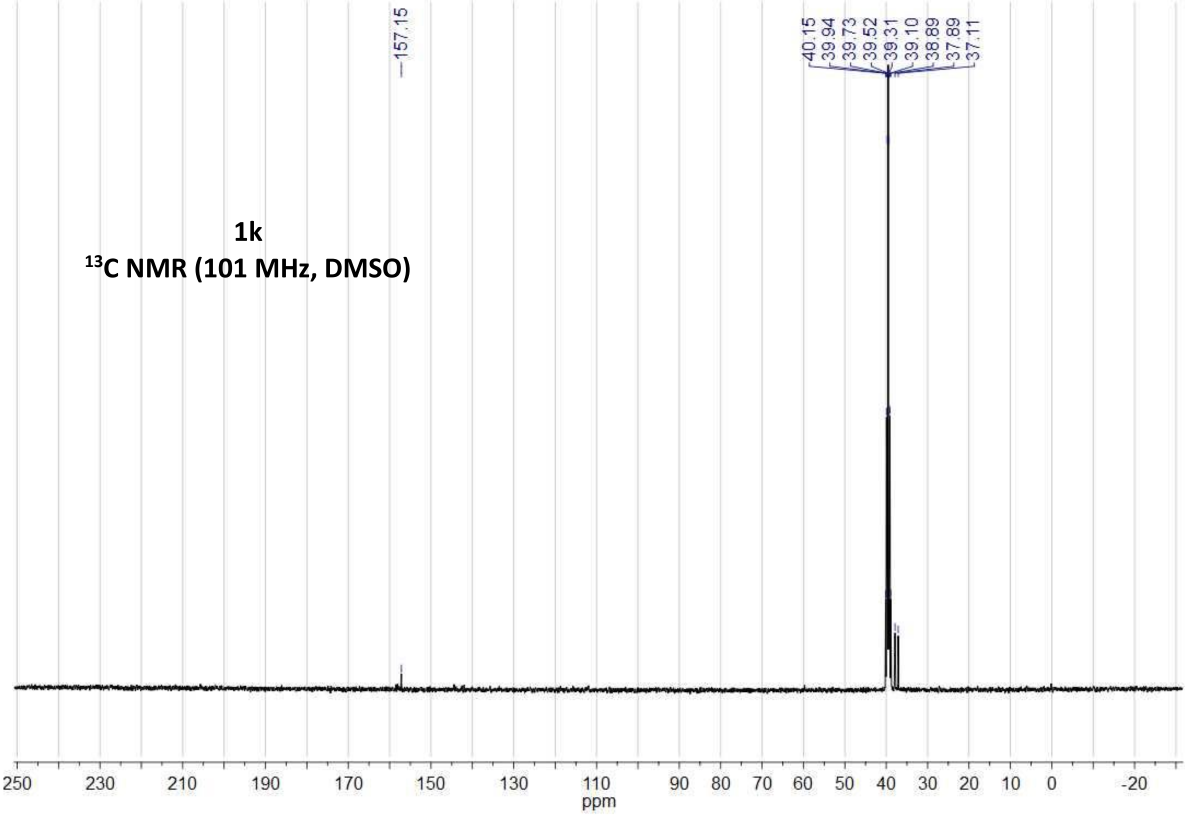
1k
13C NMR (101 MHz, DMSO)
157.15
40.15
39.94
39.73
39.52
39.31
39.10
38.89
37.89
37.11
ppm

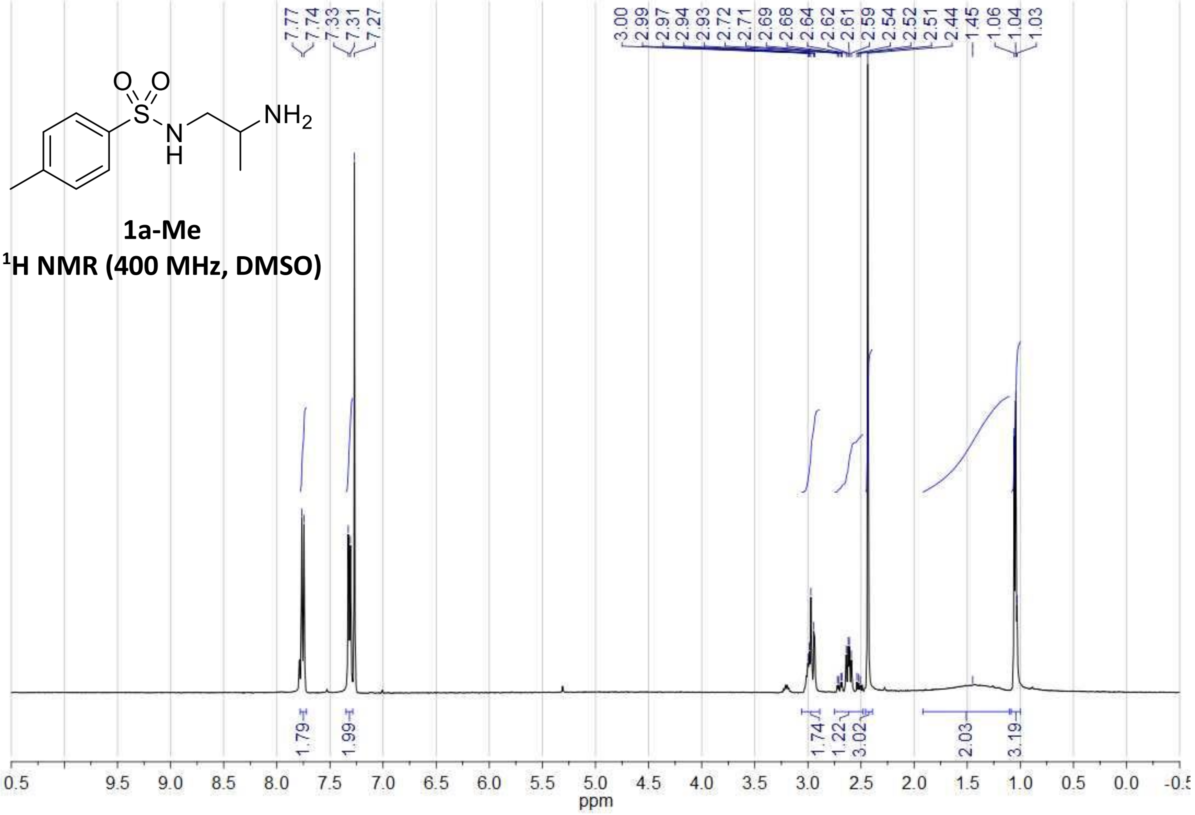
7.77
7.74
7.33
7.31
7.27
3.00
2.99
2.97
2.94
2.93
2.72
2.71
2.69
2.68
2.64
2.62
2.61
2.59
2.54
2.52
2.51
2.44
1.45
1.06
1.04
1.03
NH2
1a-Me
1H NMR (400 MHz, DMSO)
1.79
1.99
1.74
1.22
3.02
2.03
3.19
ppm

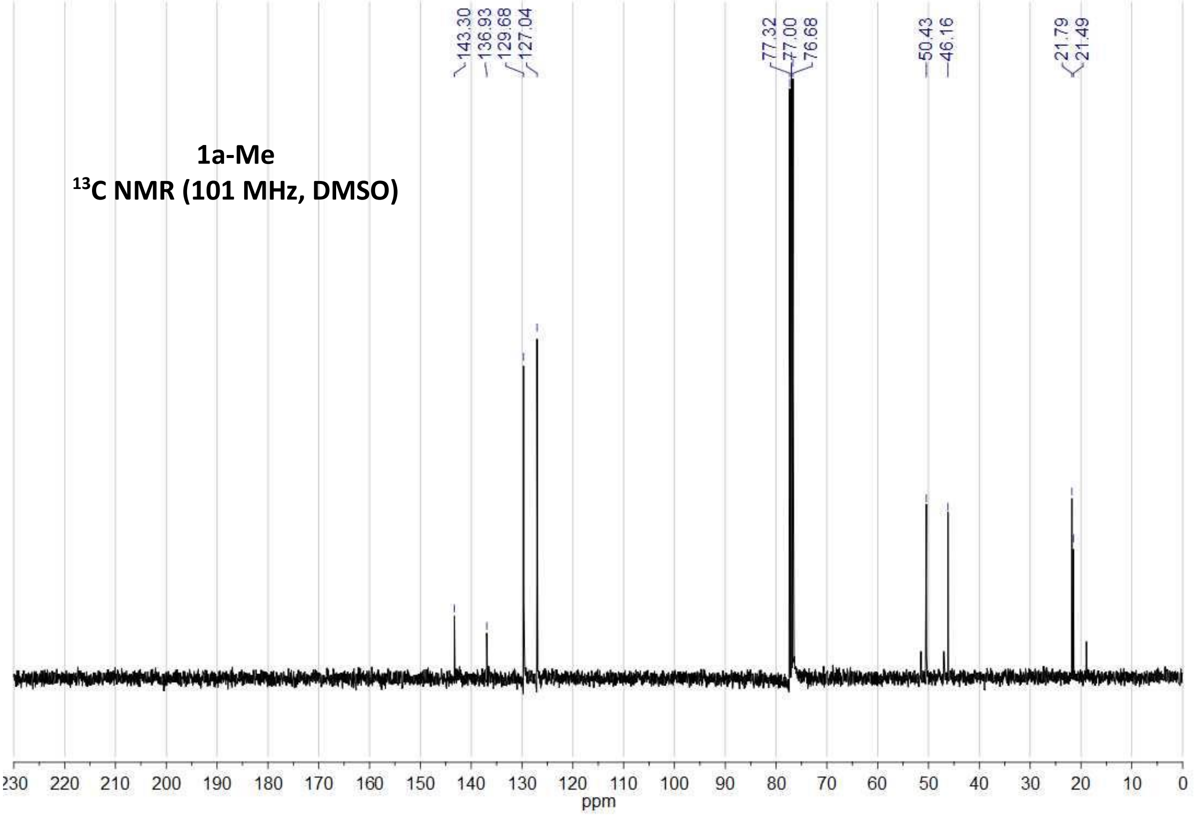
143.30
136.93
129.68
127.04
77.32
77.00
76.68
50.43
46.16
21.79
21.49
1a-Me
13C NMR (101 MHz, DMSO)
ppm

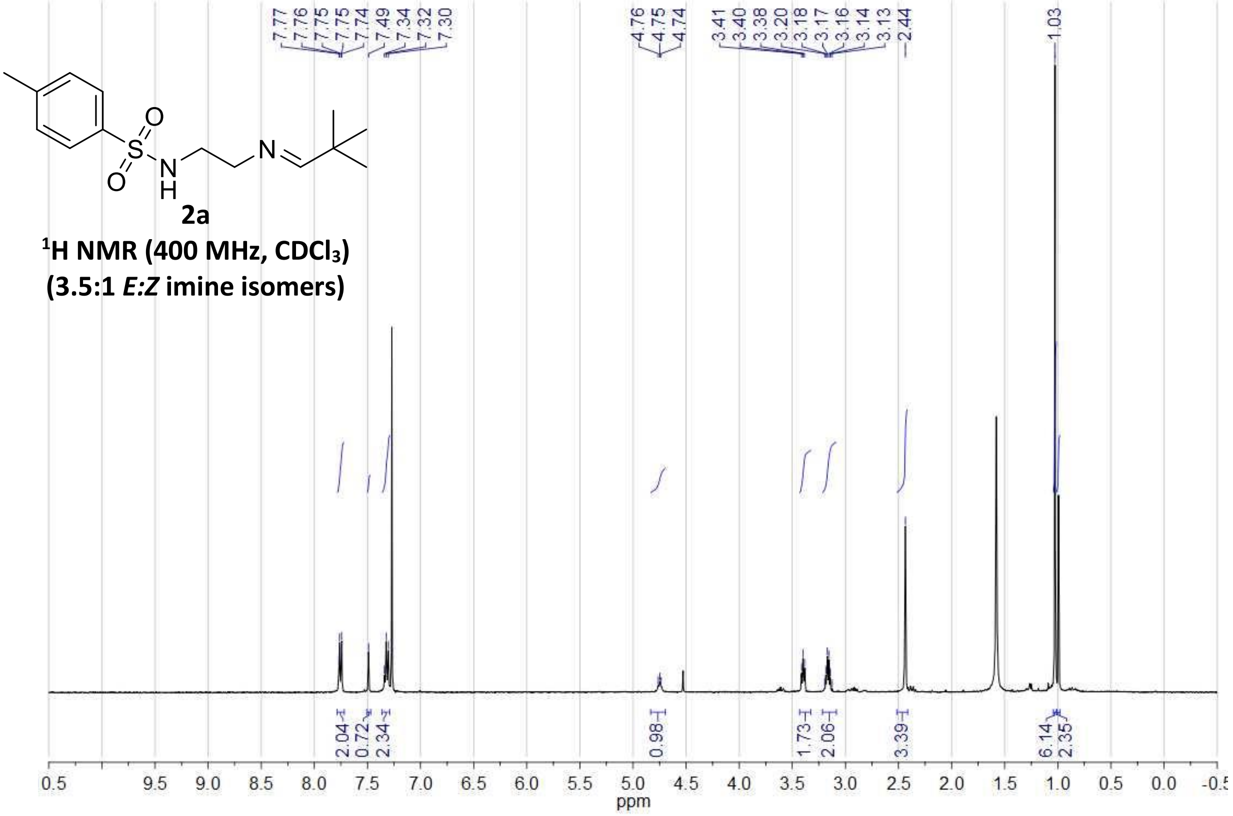
2a
1H NMR (400 MHz, CDCl3)
(3.5:1 E:Z imine isomers)
7.77 7.76 7.75 7.75 7.74 7.49 7.34 7.32 7.30
4.76 4.75 4.74
3.41 3.40 3.38 3.20 3.18 3.17 3.16 3.14 3.13 2.44
1.03
2.04 0.72 2.34 0.98 1.73 2.06 3.39 6.14 2.35
ppm

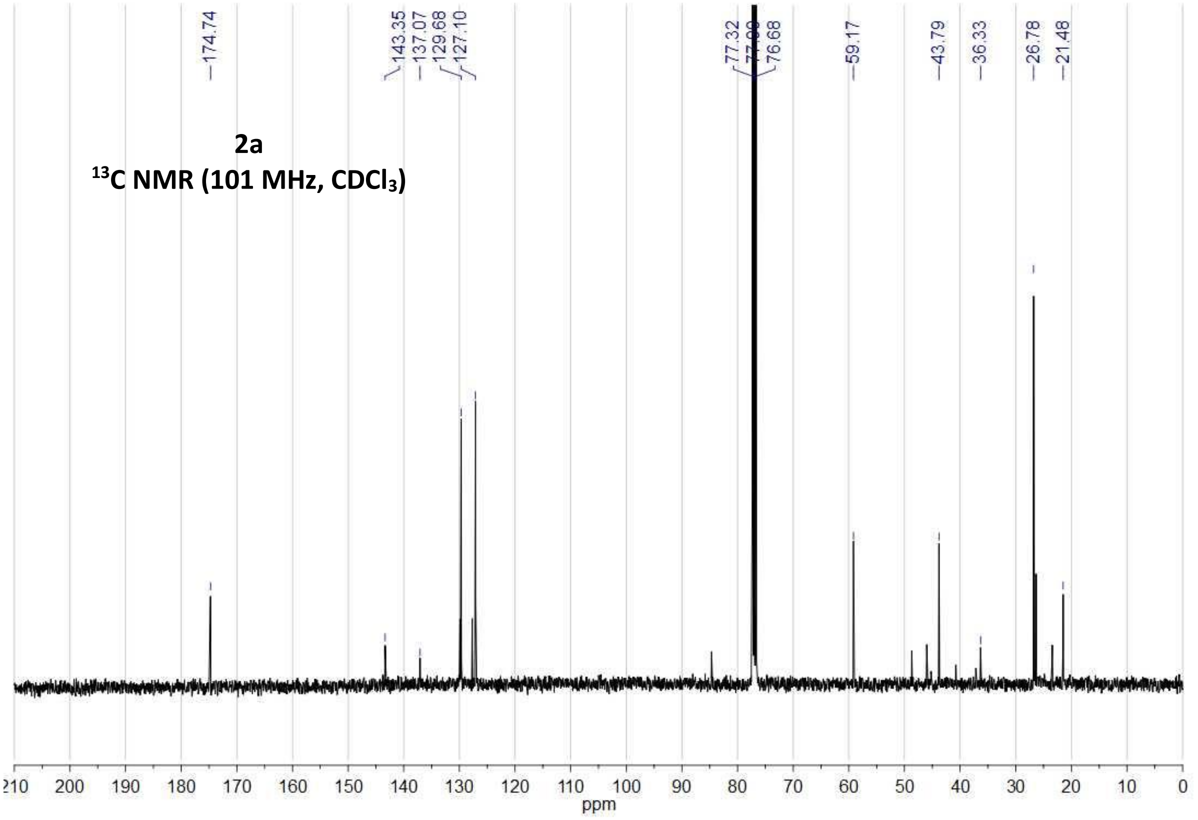
2a
13C NMR (101 MHz, CDCl3)
174.74
143.35 137.07 129.68 127.10
77.32 [illegible] 76.68
59.17
43.79
36.33
26.78
21.48
ppm

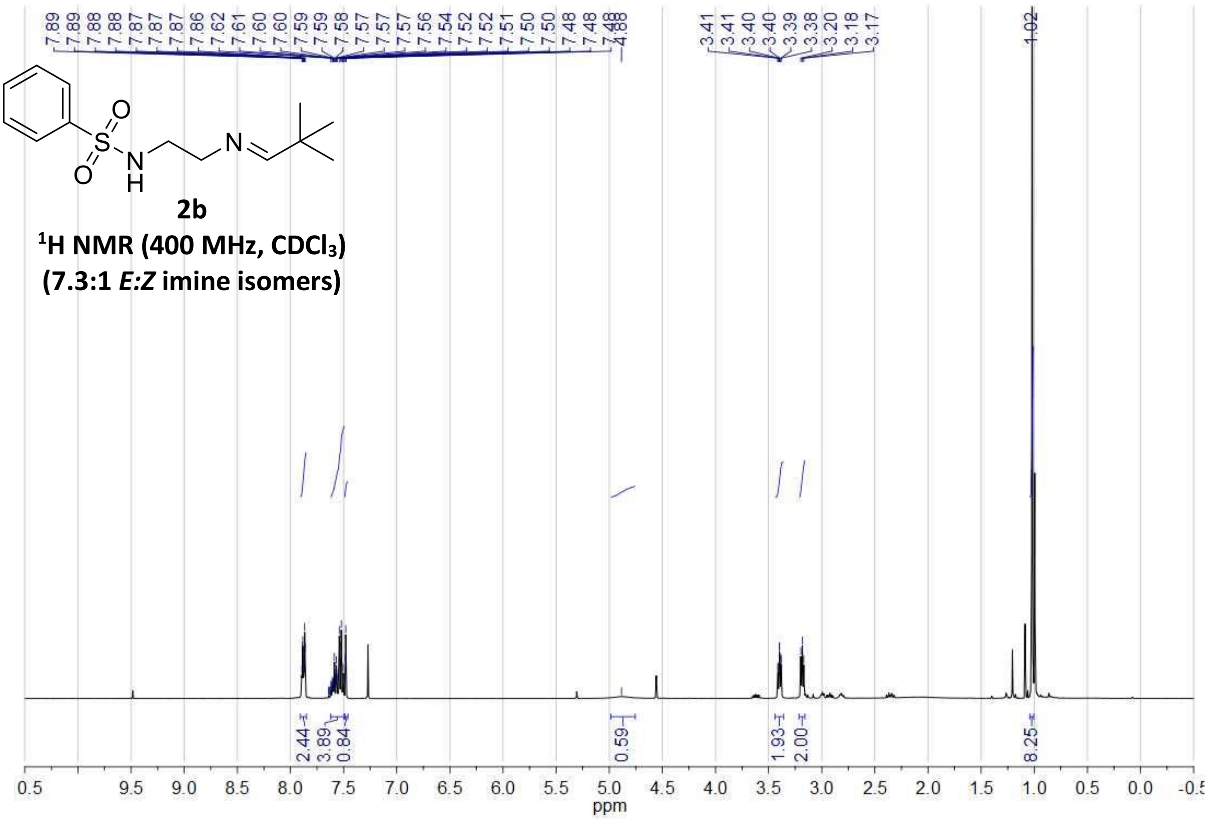
7.89 7.89 7.88 7.88 7.87 7.87 7.87 7.86 7.62 7.61 7.60 7.60 7.59 7.59 7.58 7.57 7.57 7.57 7.56 7.54 7.52 7.52 7.51 7.50 7.50 7.48 7.48 4.88
3.41 3.41 3.40 3.40 3.39 3.39 3.38 3.20 3.18 3.17
1.02
2b
^{1}H NMR (400 MHz, $CDCl_3$)
(7.3:1 *E:Z* imine isomers)
2.44 3.89 0.84 0.59 1.93 2.00 8.25
ppm

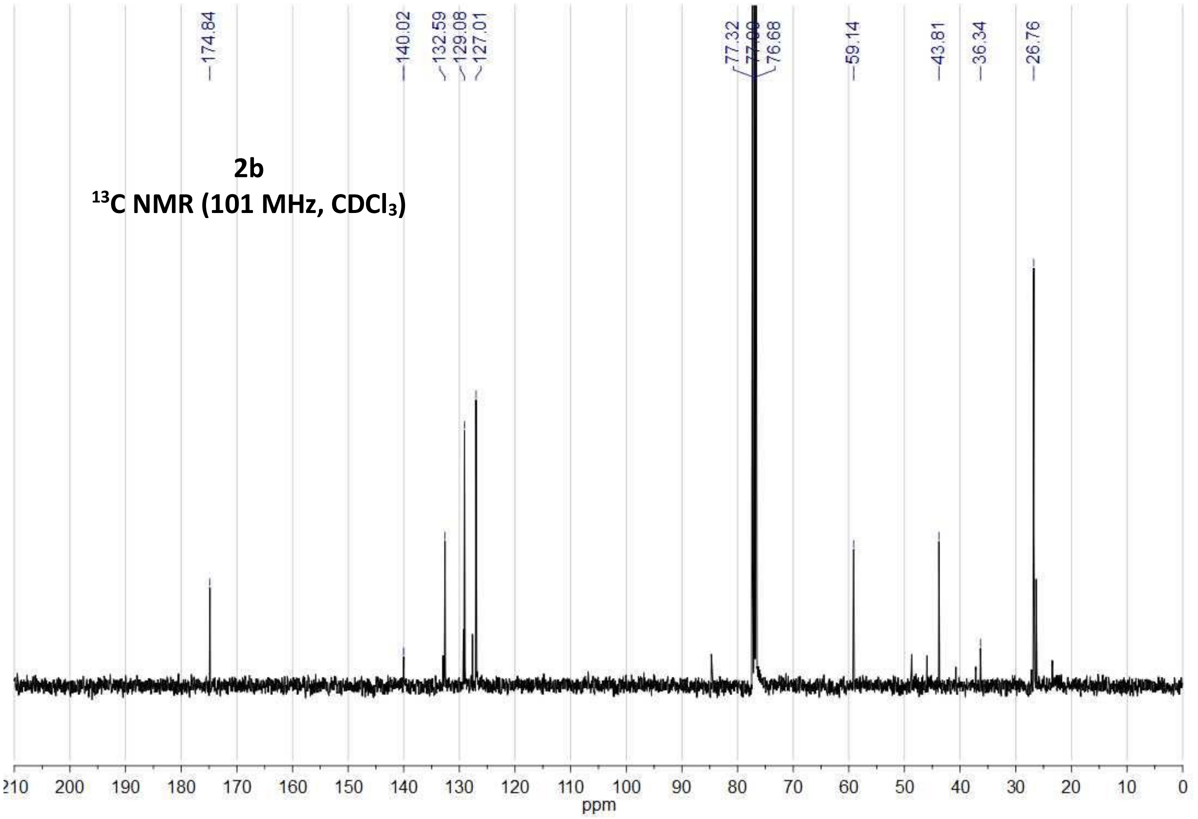
174.84
140.02 132.59 129.08 127.01
77.32 77.00 76.68
59.14
43.81
36.34
26.76
2b
^{13}C NMR (101 MHz, $CDCl_3$)
ppm

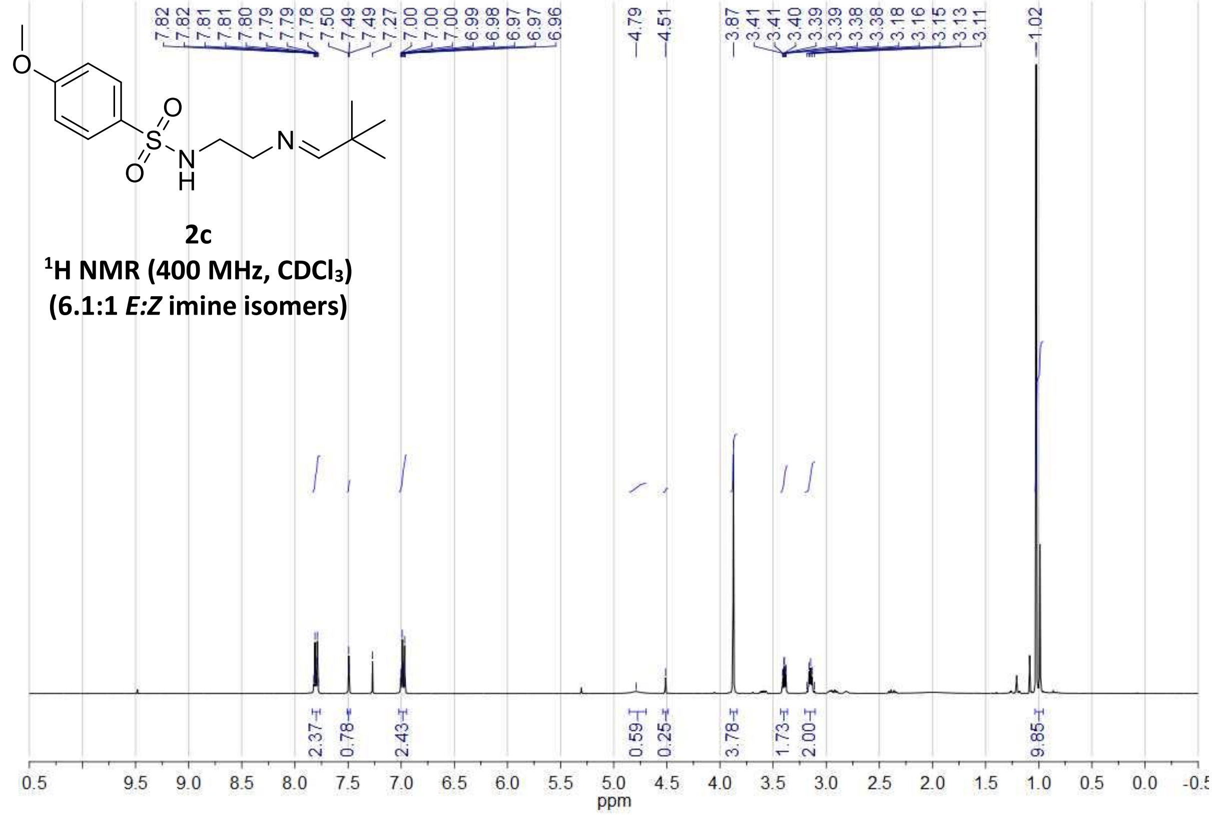
2c
^{1}H NMR (400 MHz, CDCl$_3$)
(6.1:1 *E:Z* imine isomers)
7.82 7.82 7.81 7.81 7.80 7.79 7.79 7.78 7.50 7.49 7.49 7.27 7.00 7.00 7.00 6.99 6.98 6.97 6.97 6.96
4.79 4.51
3.87 3.41 3.41 3.40 3.39 3.39 3.38 3.38 3.18 3.16 3.15 3.13 3.11
1.02
2.37 0.78 2.43 0.59 0.25 3.78 1.73 2.00 9.85
ppm

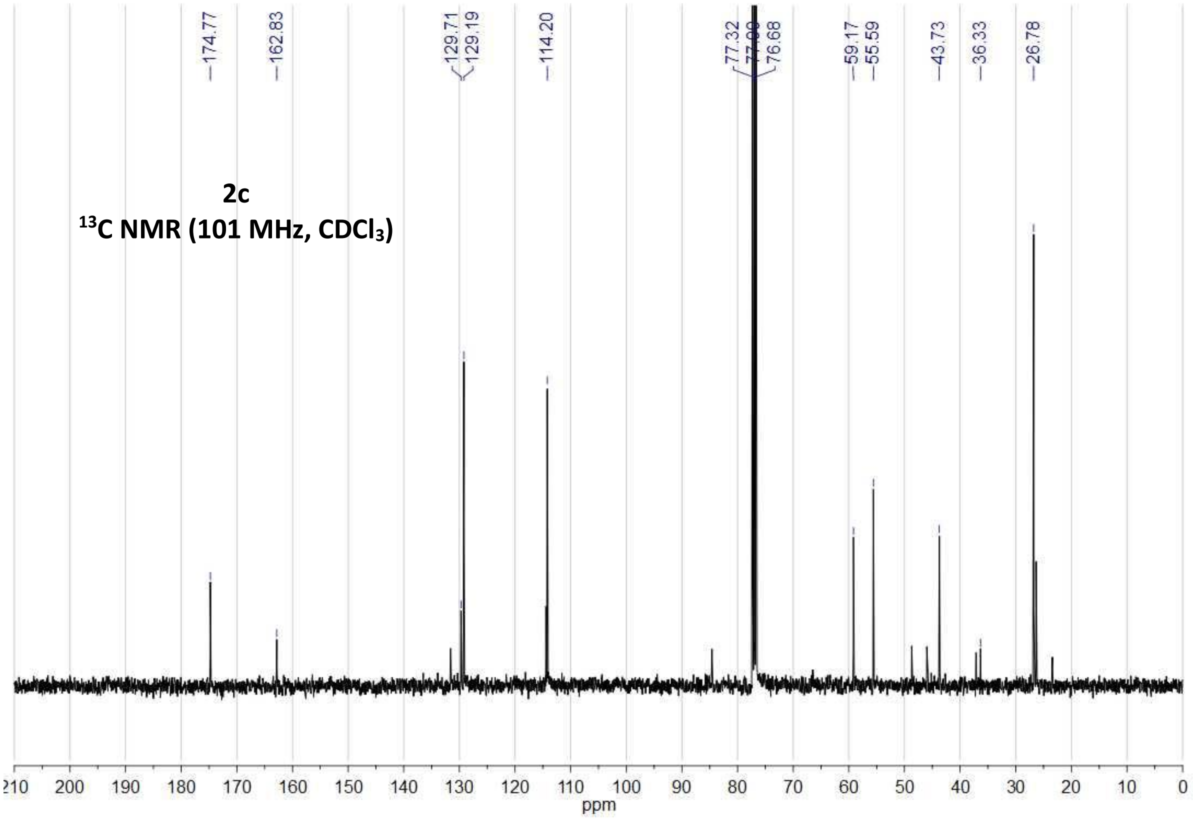
2c
^{13}C NMR (101 MHz, CDCl$_3$)
174.77 162.83 129.71 129.19 114.20 77.32 77.00 76.68 59.17 55.59 43.73 36.33 26.78
ppm

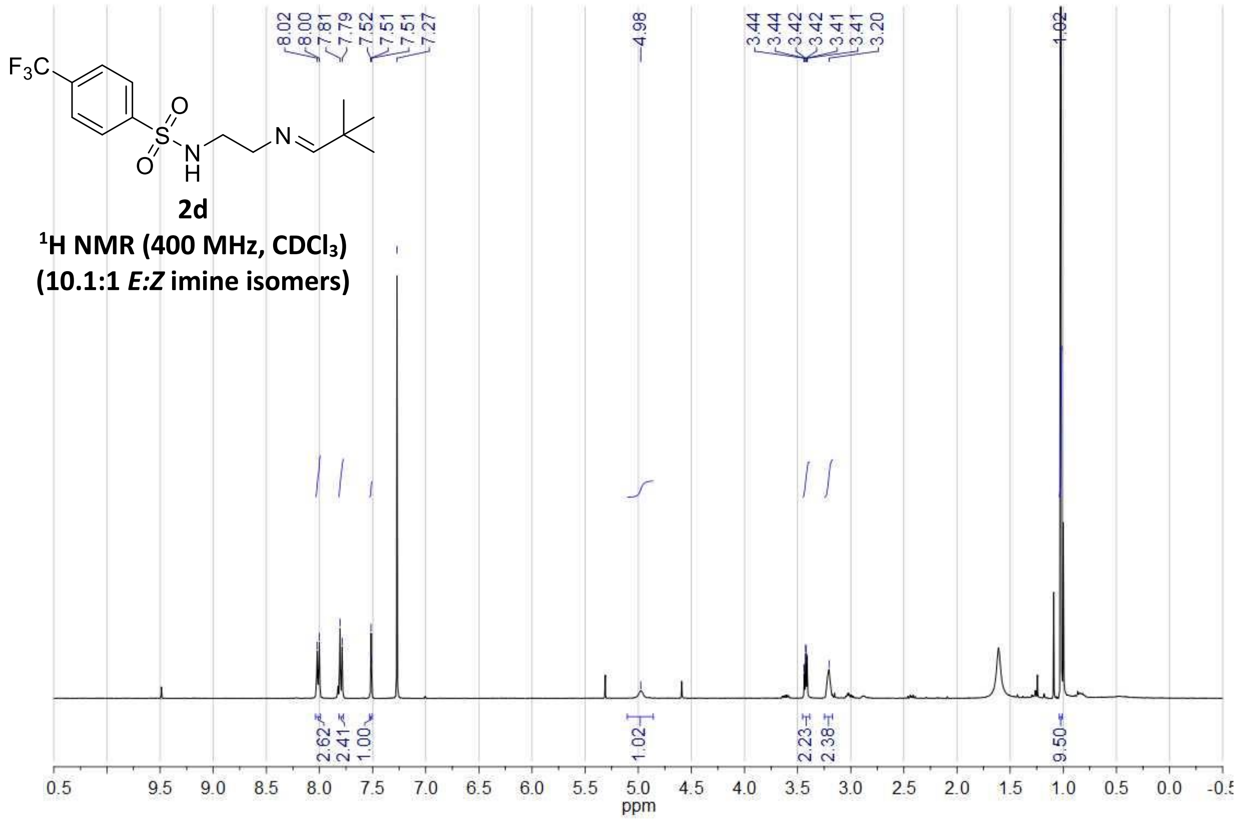

2d

^{1}H NMR (400 MHz, CDCl$_3$)

(10.1:1 *E:Z* imine isomers)

2d

^{13}C NMR (101 MHz, CDCl$_3$)

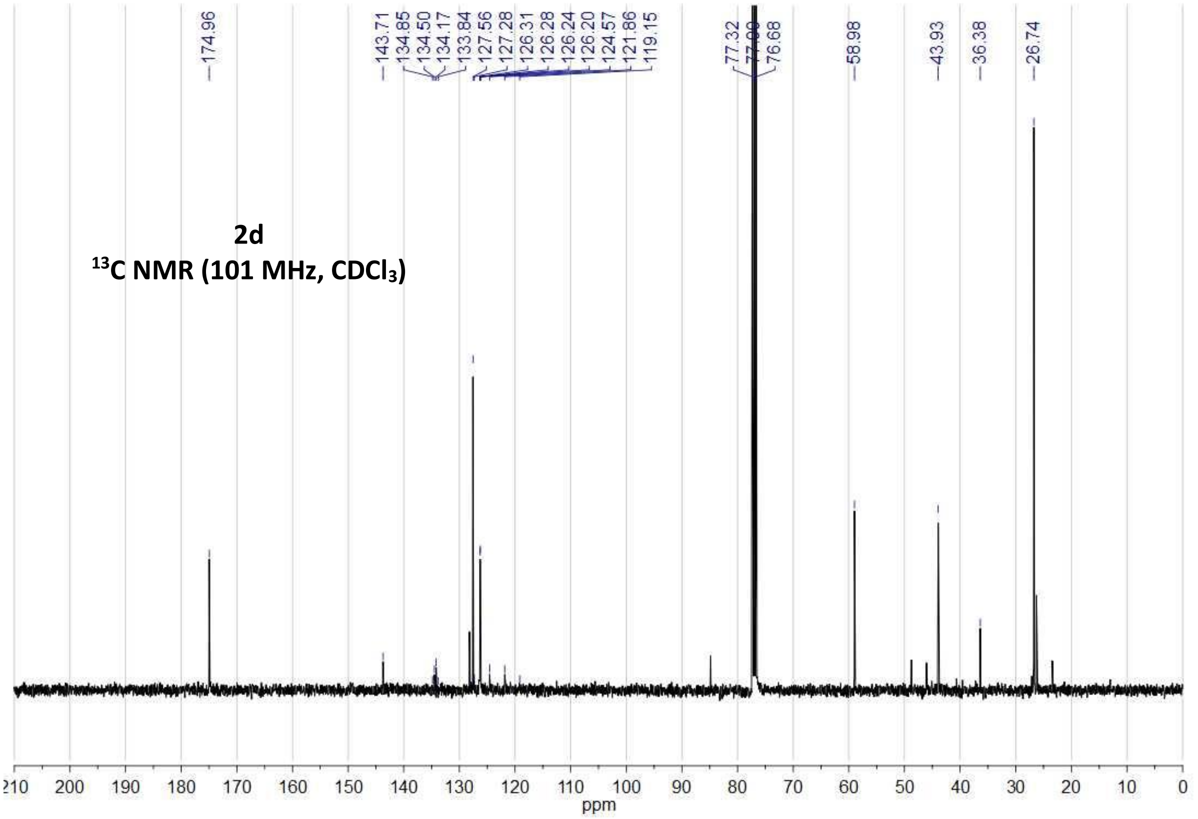

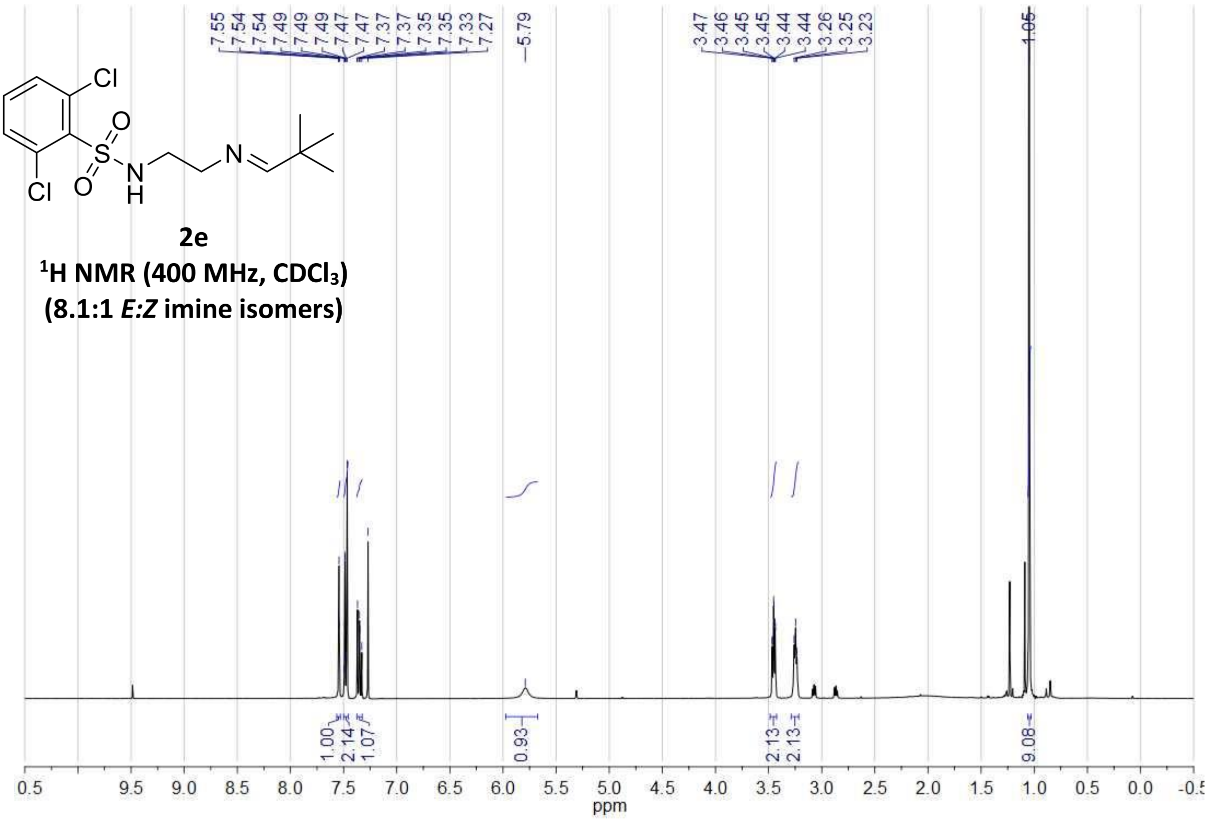
2e
^{1}H NMR (400 MHz, CDCl$_3$)
(8.1:1 *E:Z* imine isomers)
7.55 7.54 7.54 7.49 7.49 7.49 7.47 7.47 7.37 7.37 7.35 7.35 7.33 7.27
5.79
3.47 3.46 3.45 3.45 3.44 3.44 3.26 3.25 3.23
1.05
ppm

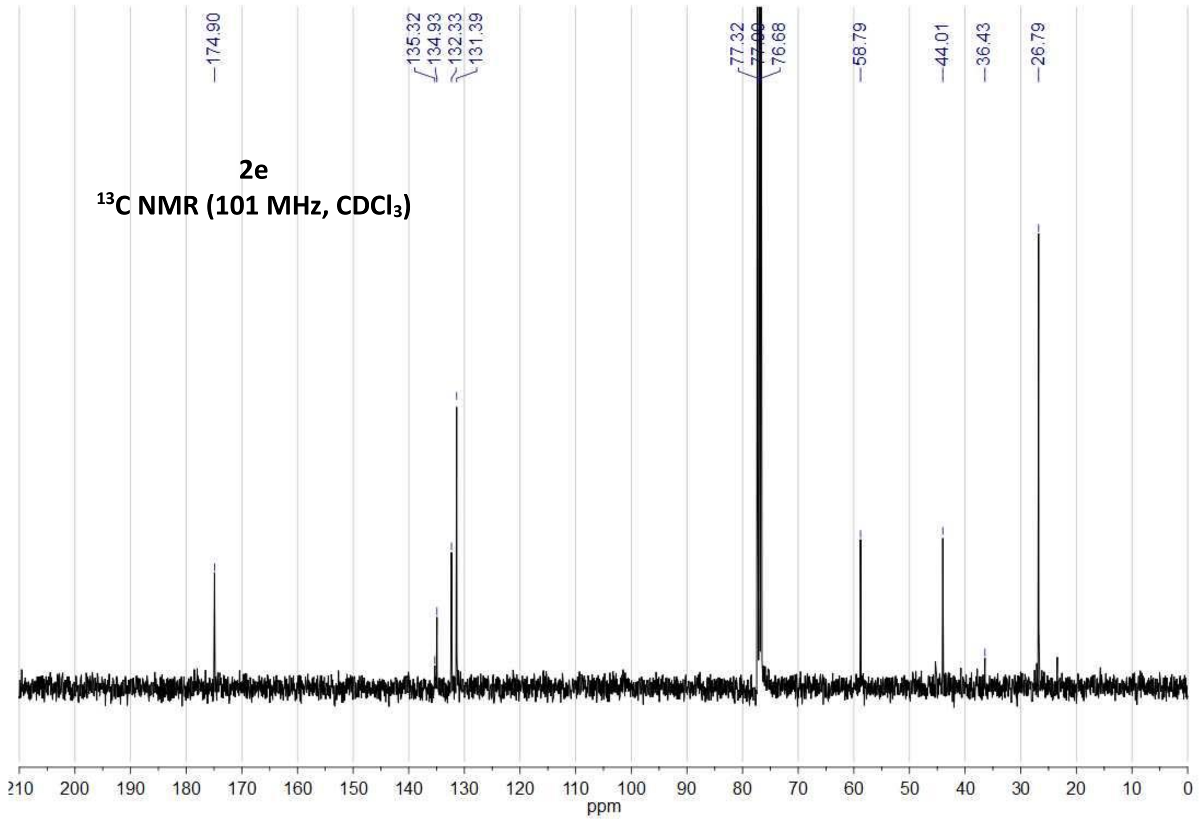
2e
^{13}C NMR (101 MHz, CDCl$_3$)
174.90
135.32 134.93 132.33 131.39
77.32 76.68
58.79
44.01
36.43
26.79
ppm

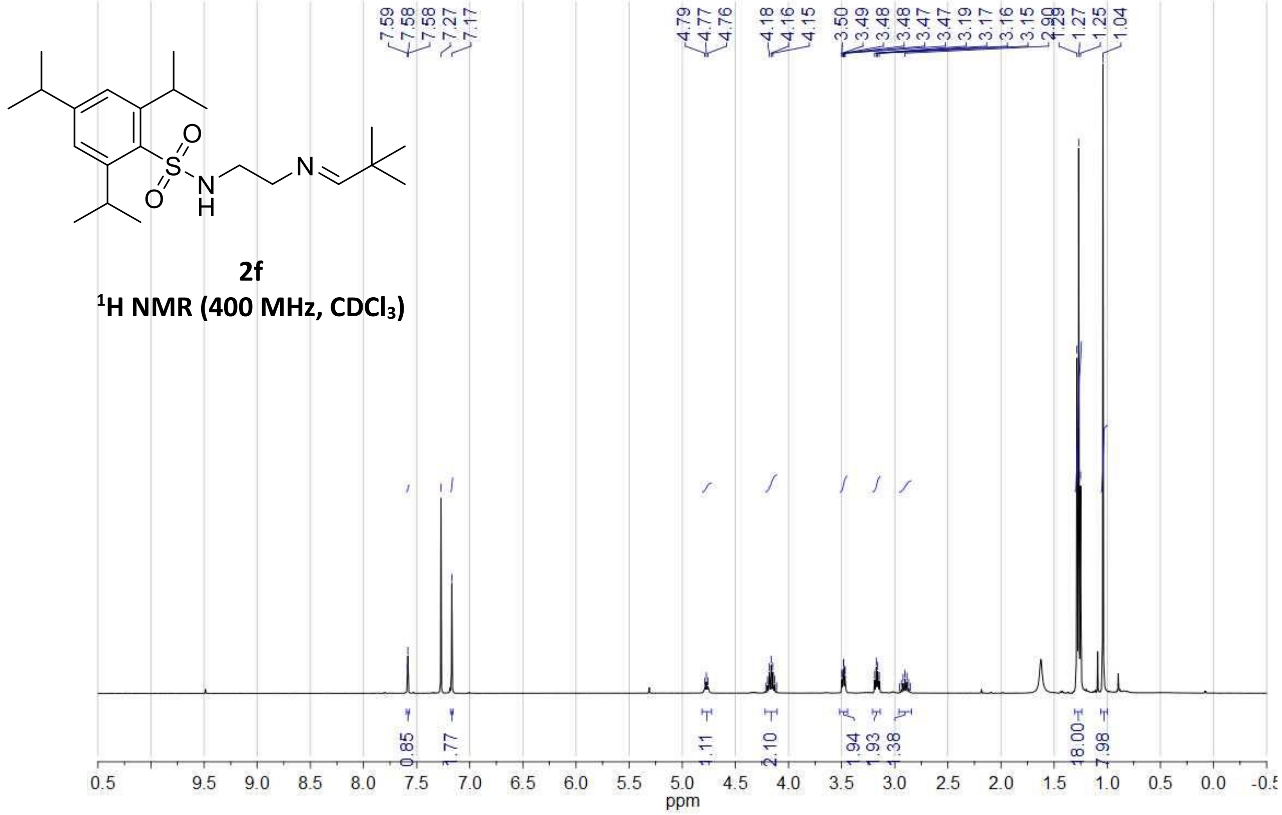

2f
^{1}H NMR (400 MHz, $CDCl_3$)

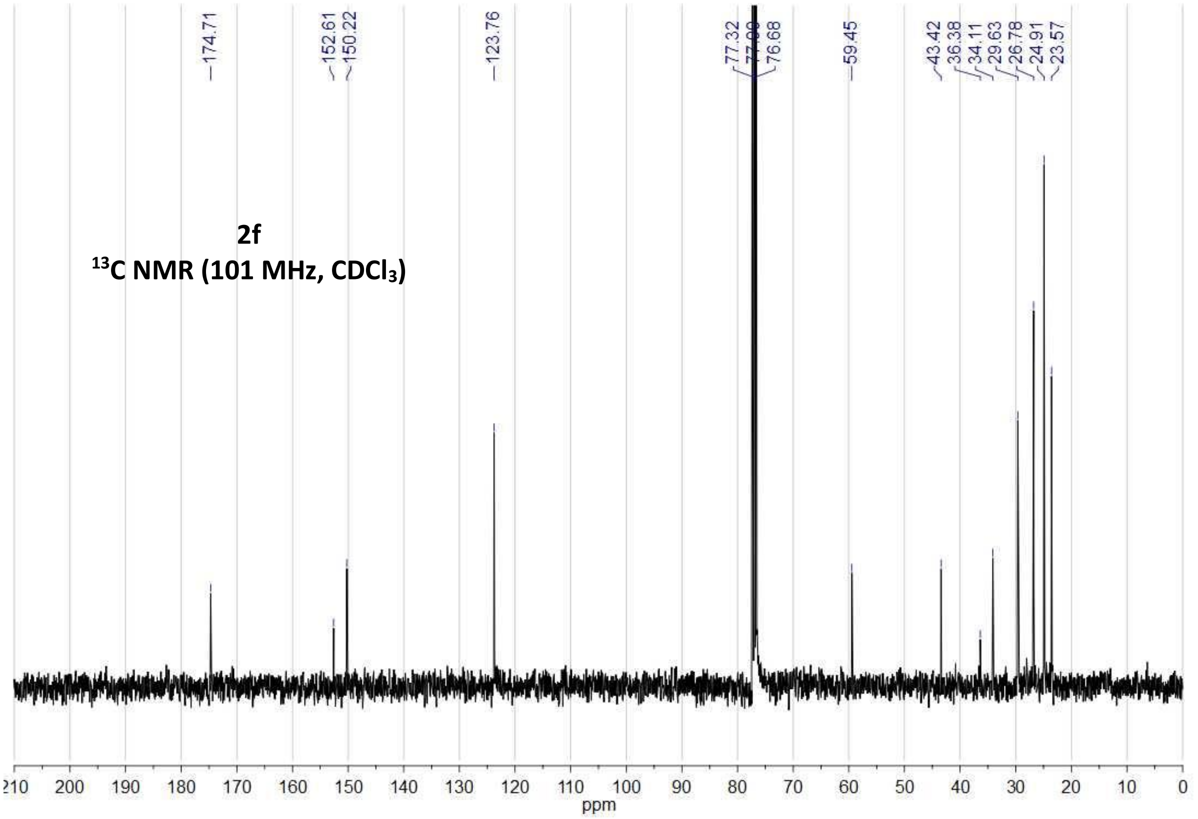

2f
^{13}C NMR (101 MHz, $CDCl_3$)

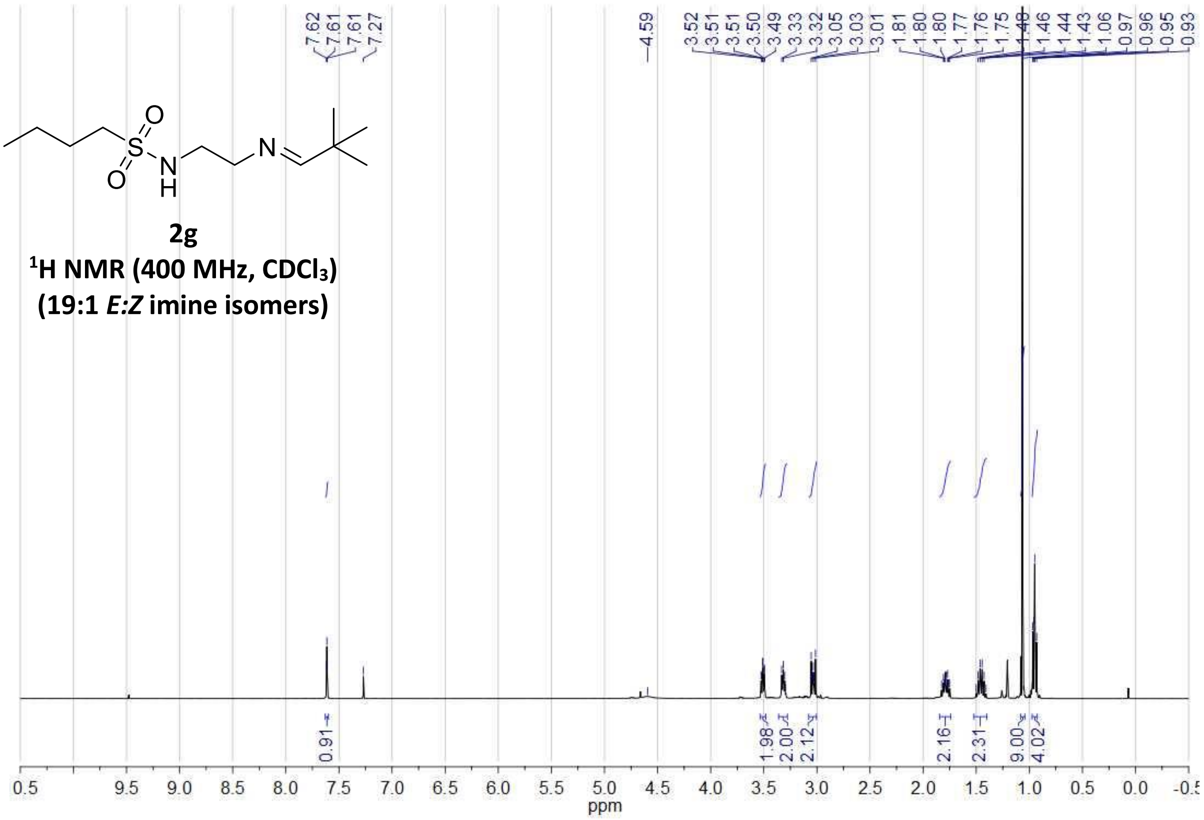
2g
^{1}H NMR (400 MHz, $CDCl_3$)
(19:1 *E:Z* imine isomers)
ppm

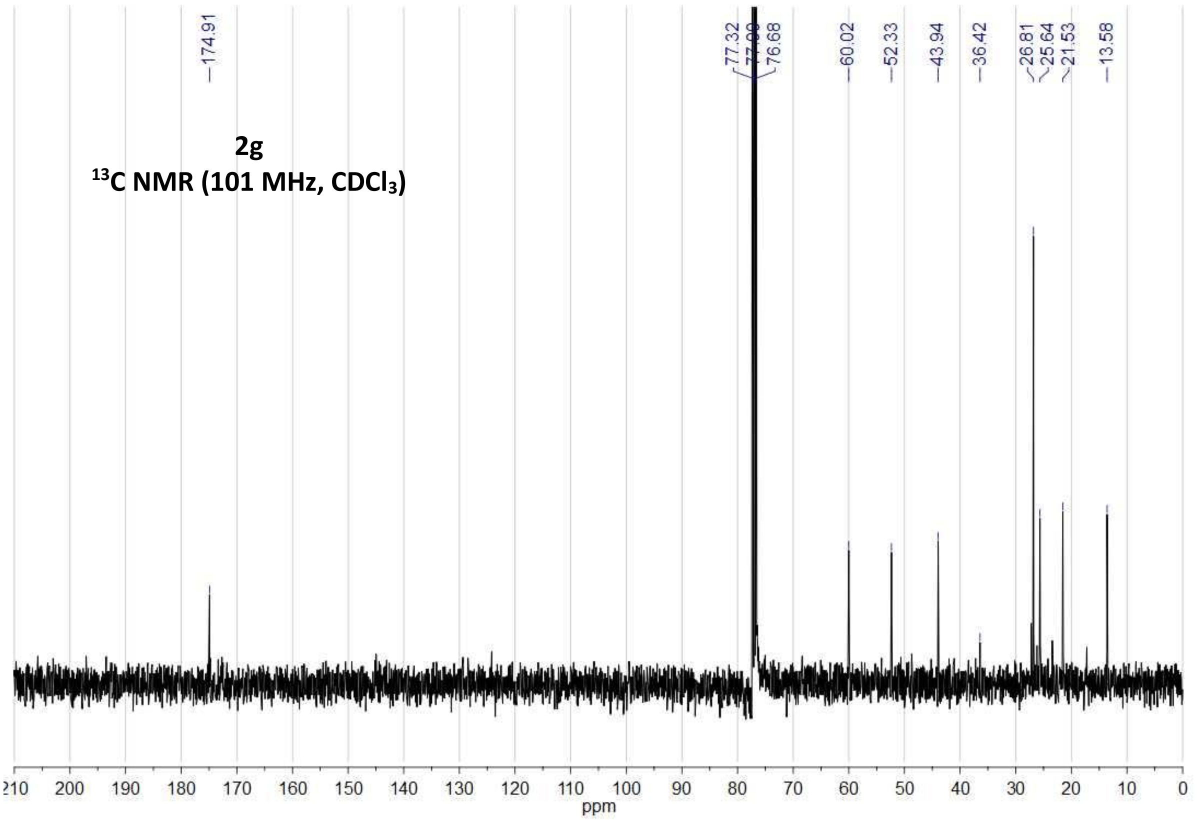
2g
^{13}C NMR (101 MHz, $CDCl_3$)
ppm

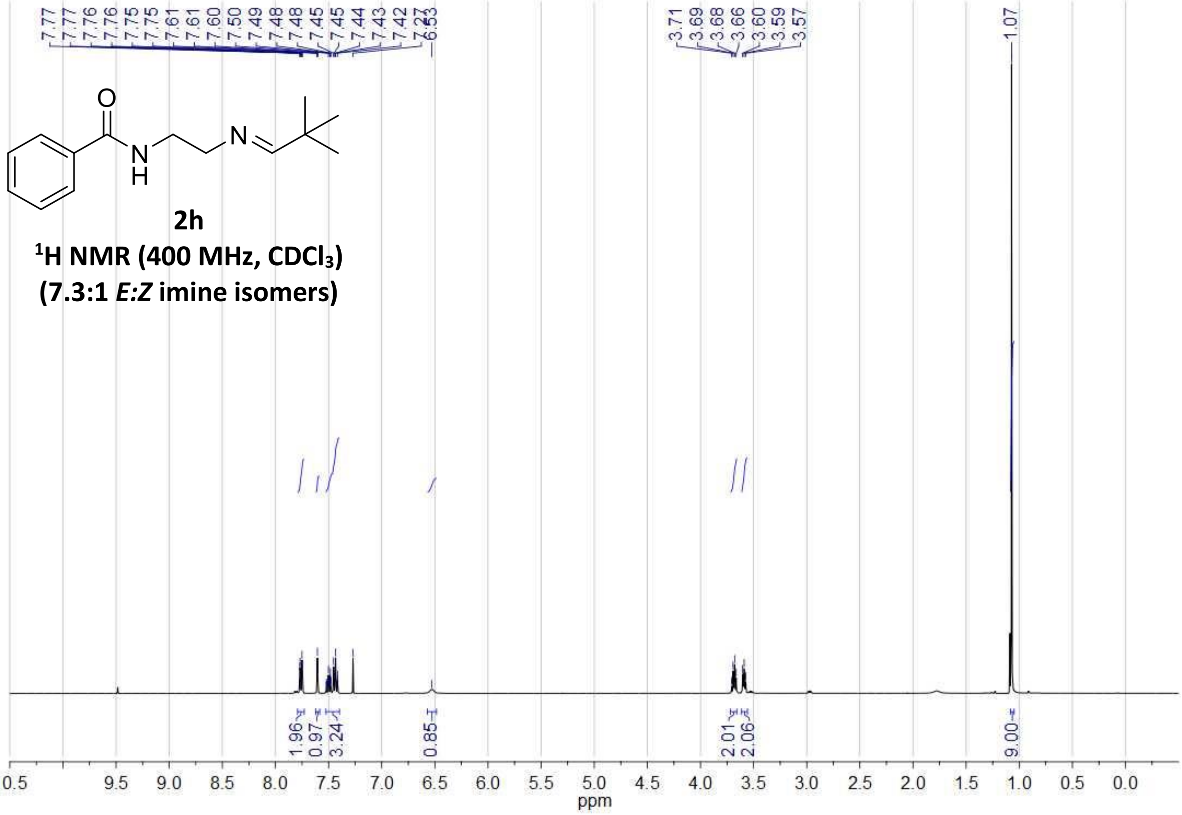
2h
^{1}H NMR (400 MHz, CDCl$_3$)
(7.3:1 *E:Z* imine isomers)
7.77 7.77 7.76 7.76 7.75 7.75 7.61 7.61 7.60 7.50 7.49 7.48 7.48 7.45 7.45 7.44 7.43 7.42 7.27 6.53
3.71 3.69 3.68 3.66 3.60 3.59 3.57
1.07
1.96 0.97 3.24 0.85 2.01 2.06 9.00
ppm

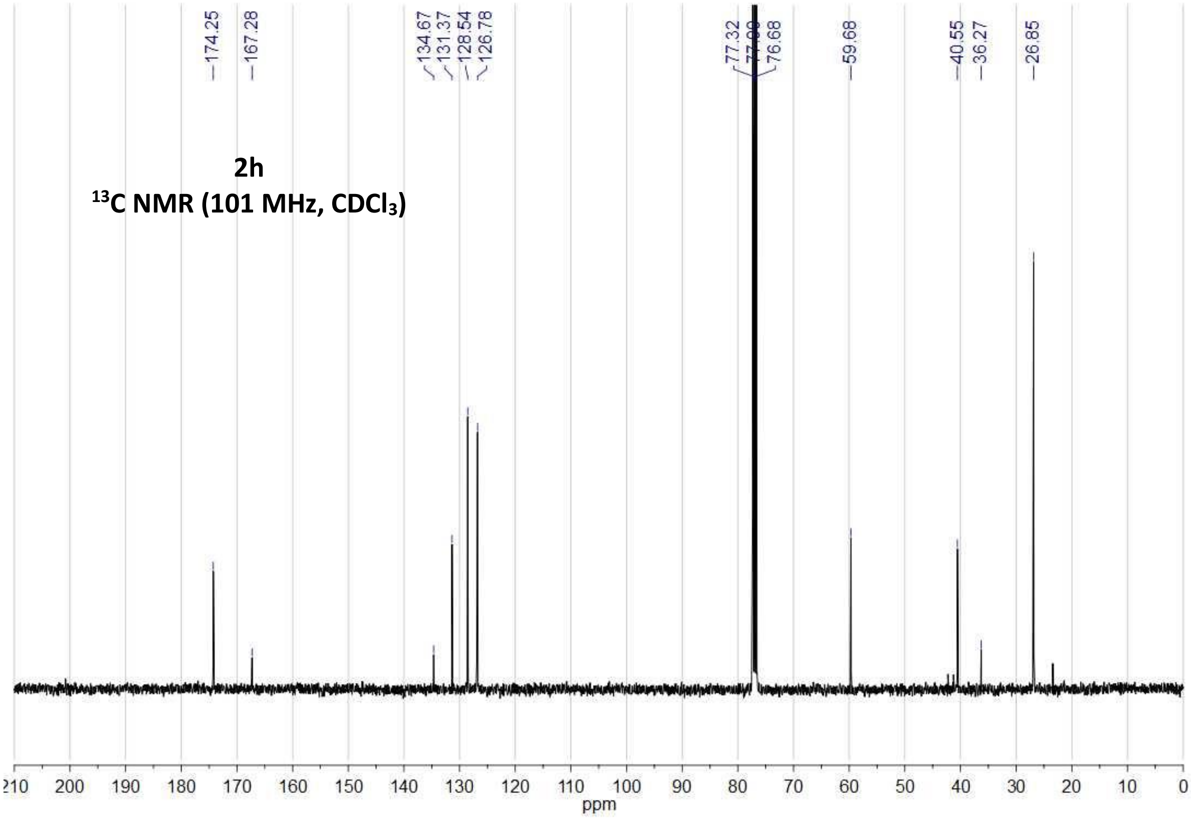
2h
^{13}C NMR (101 MHz, CDCl$_3$)
174.25 167.28
134.67 131.37 128.54 126.78
77.32 76.68
59.68
40.55 36.27
26.85
ppm

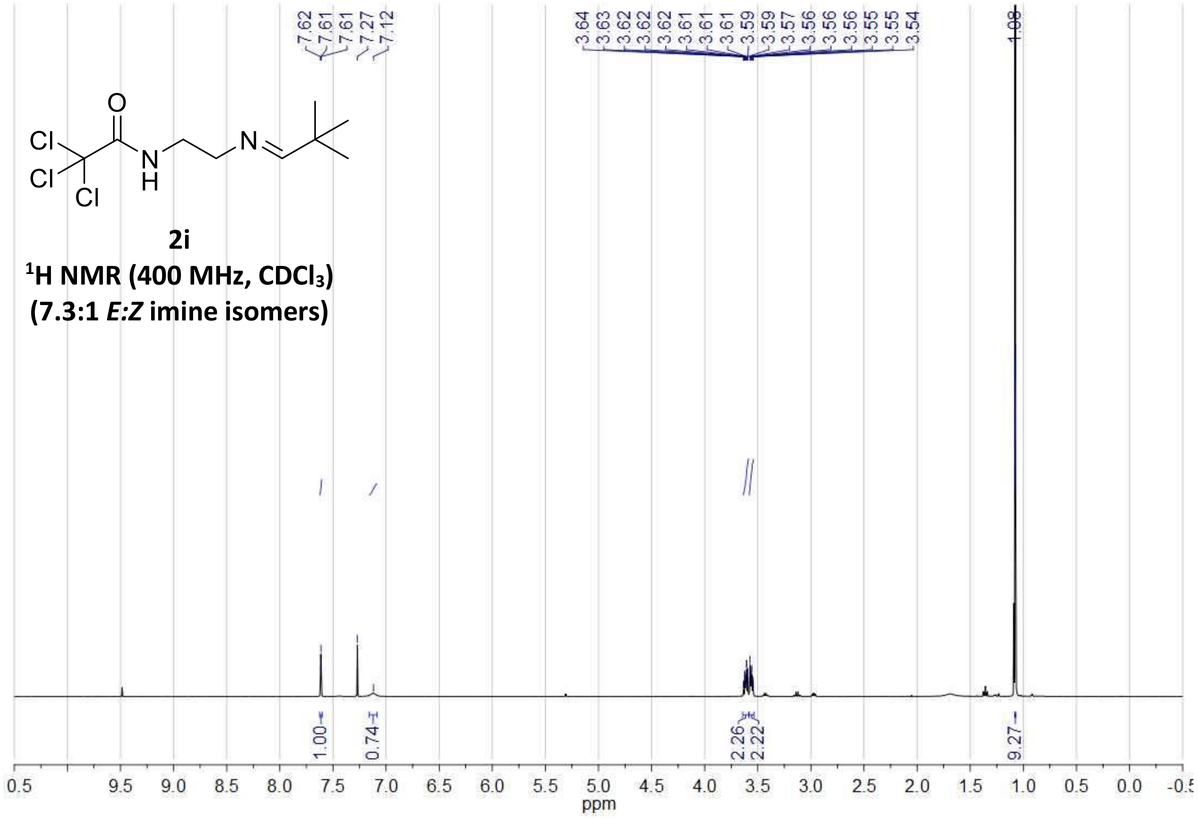

2i

^{1}H NMR (400 MHz, $CDCl_3$)

(7.3:1 *E*:*Z* imine isomers)

2i

^{13}C NMR (101 MHz, $CDCl_3$)

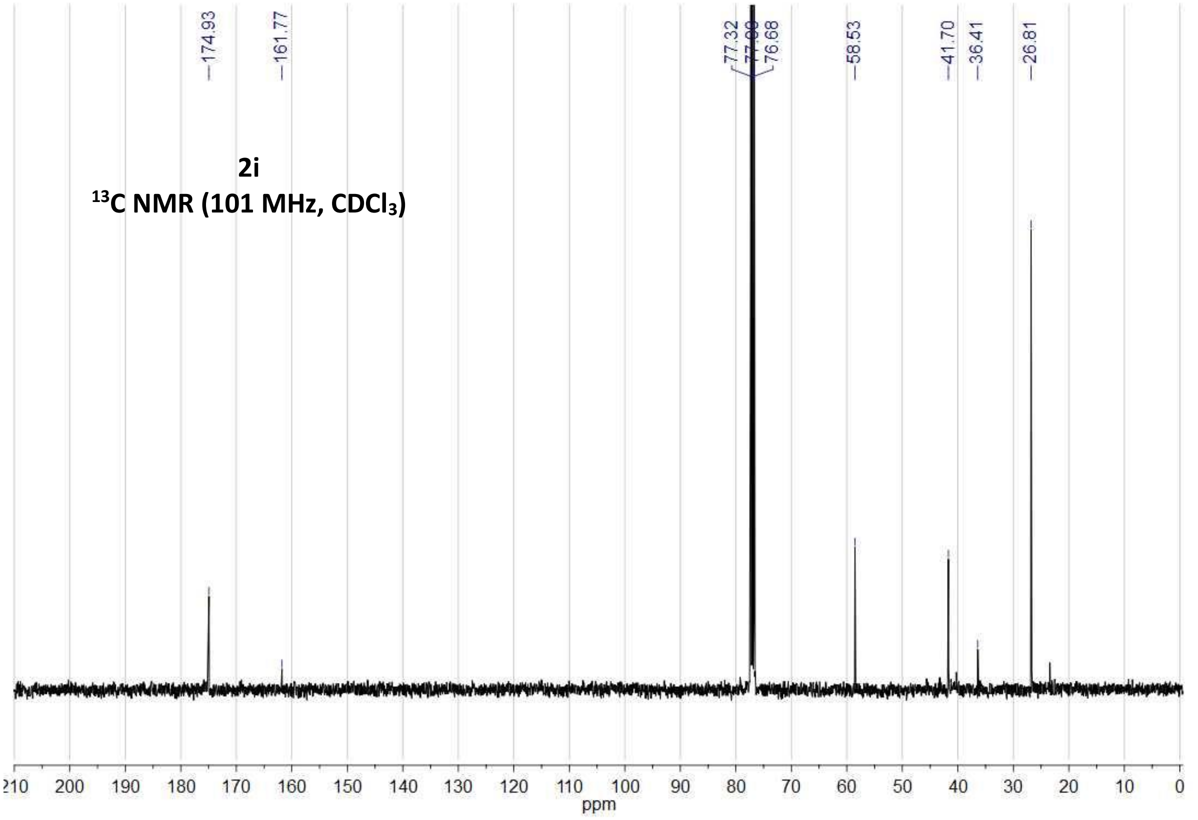

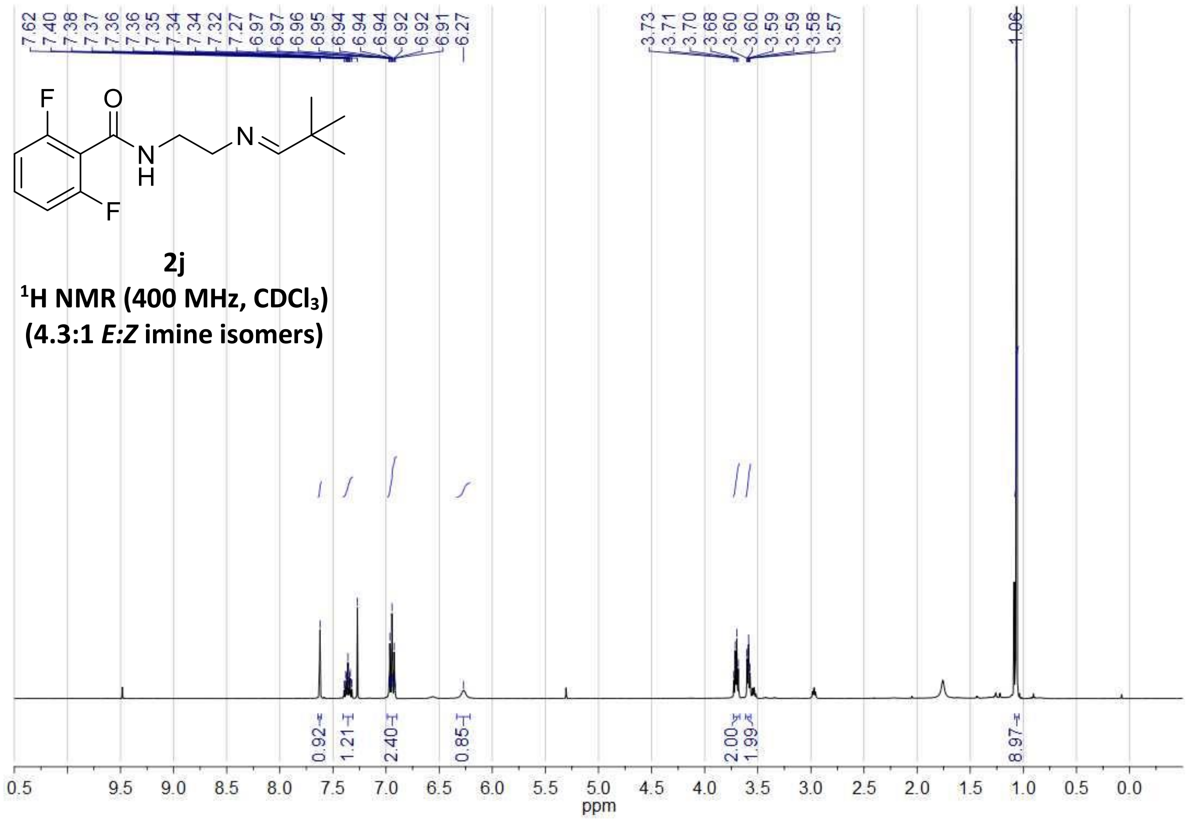
7.62 7.40 7.38 7.37 7.36 7.36 7.35 7.34 7.34 7.32 7.27 6.97 6.97 6.96 6.95 6.94 6.94 6.94 6.92 6.92 6.91 6.27
3.73 3.71 3.70 3.68 3.60 3.60 3.59 3.59 3.58 3.57
1.06
F
O
N
H
N
F
2j
^{1}H NMR (400 MHz, $CDCl_3$)
(4.3:1 *E:Z* imine isomers)
0.92 1.21 2.40 0.85 2.00 1.99 8.97
ppm

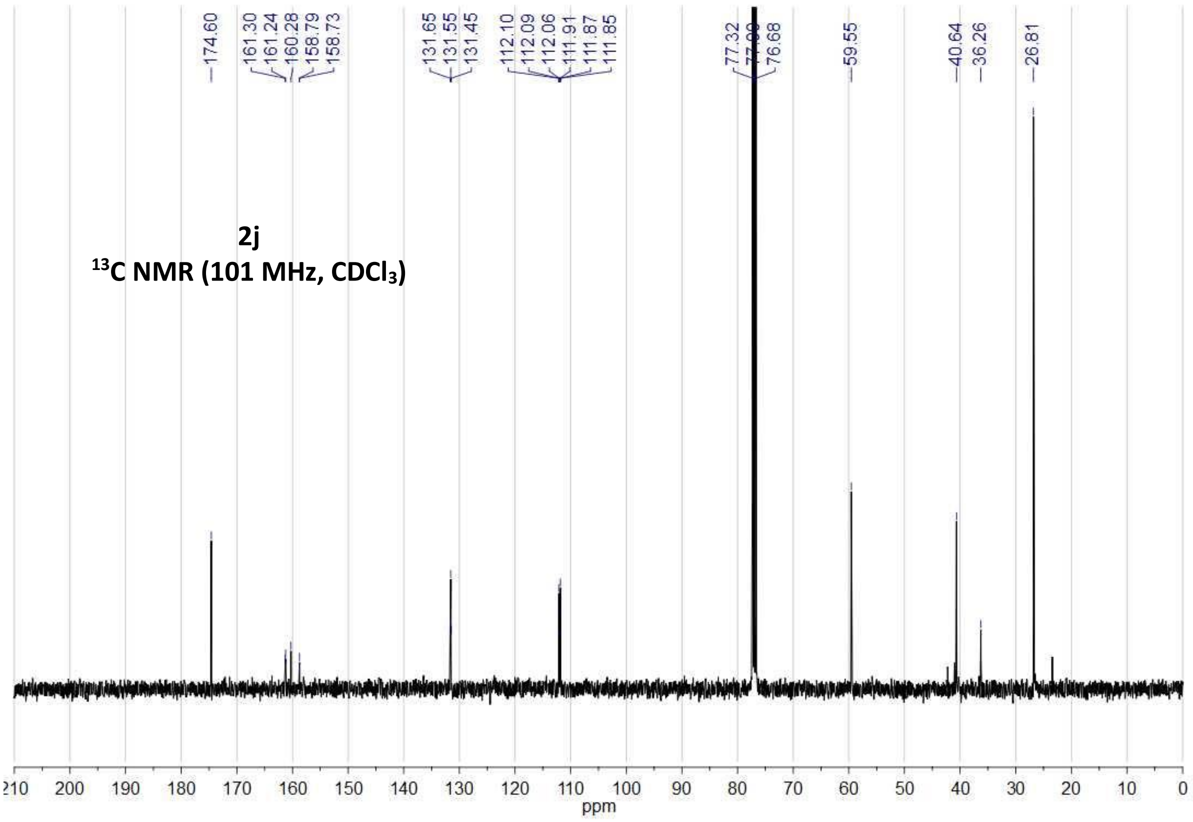
174.60
161.30 161.24 160.28 158.79 158.73
131.65 131.55 131.45
112.10 112.09 112.06 111.91 111.87 111.85
77.32 76.68
59.55
40.64 36.26
26.81
2j
^{13}C NMR (101 MHz, $CDCl_3$)
ppm

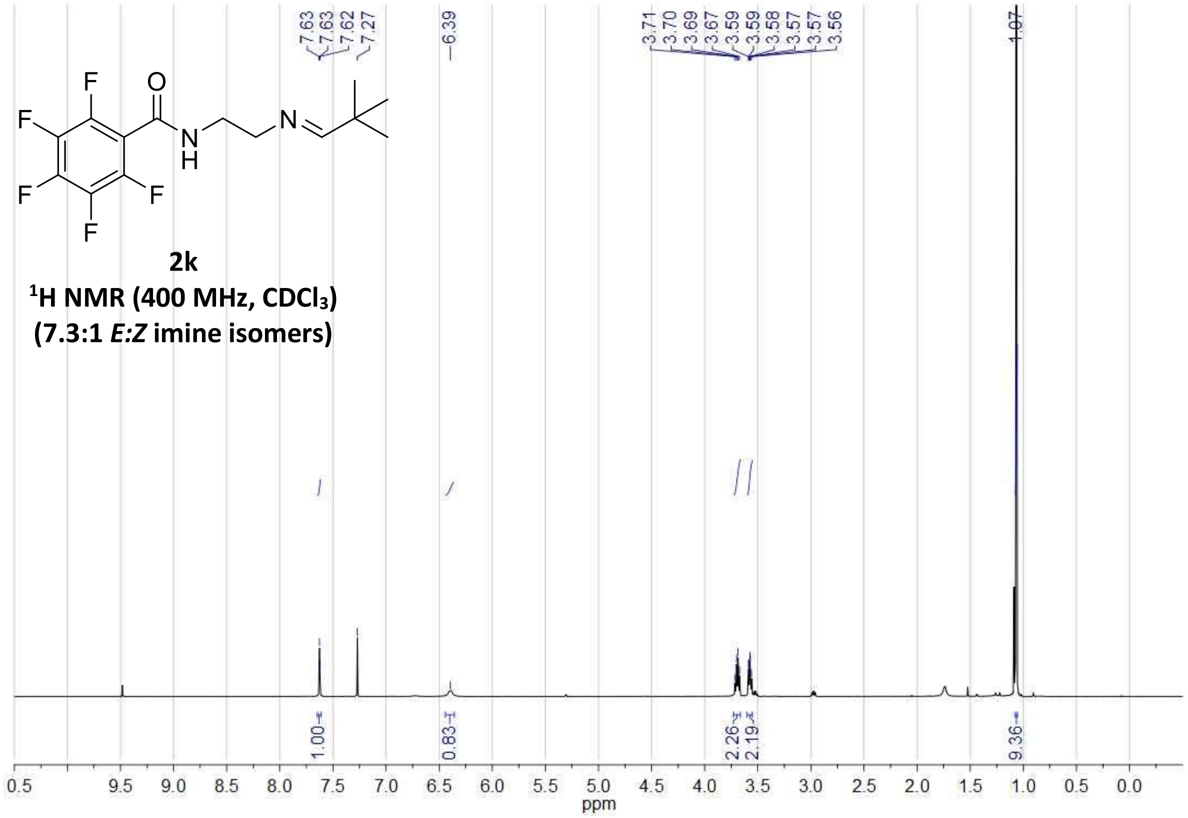

2k
^{1}H NMR (400 MHz, $CDCl_3$)
(7.3:1 *E:Z* imine isomers)

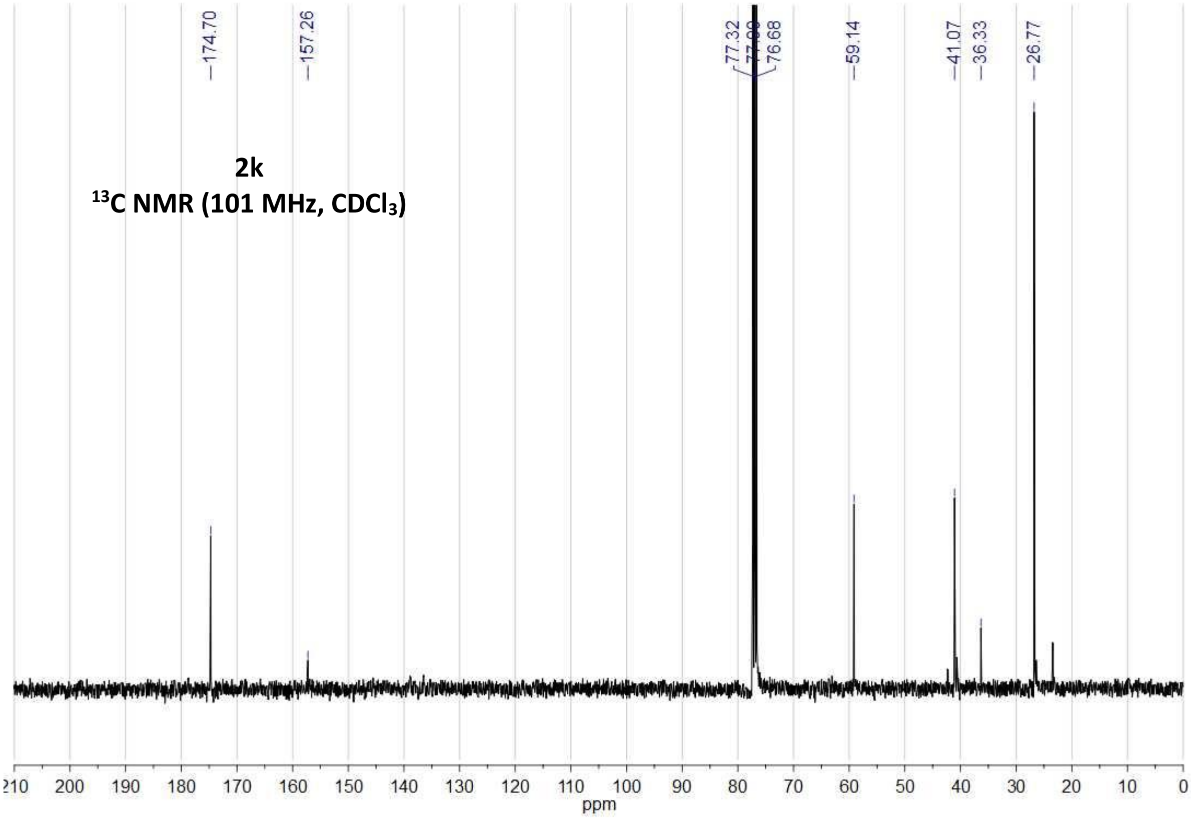

2k
^{13}C NMR (101 MHz, $CDCl_3$)

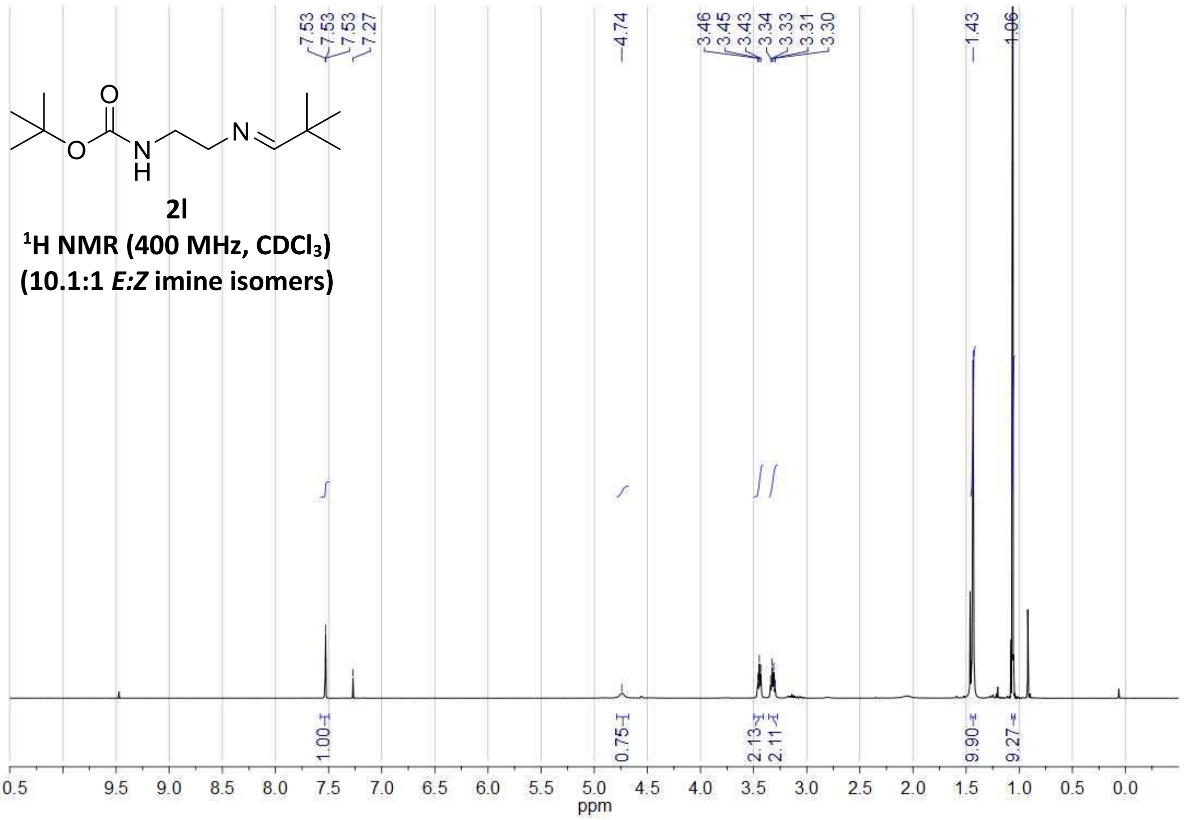
2l
^{1}H NMR (400 MHz, $CDCl_3$)
(10.1:1 *E:Z* imine isomers)
7.53 7.53 7.53 7.27
4.74
3.46 3.45 3.43 3.34 3.33 3.31 3.30
1.43
1.06
1.00
0.75
2.13
2.11
9.90
9.27
ppm

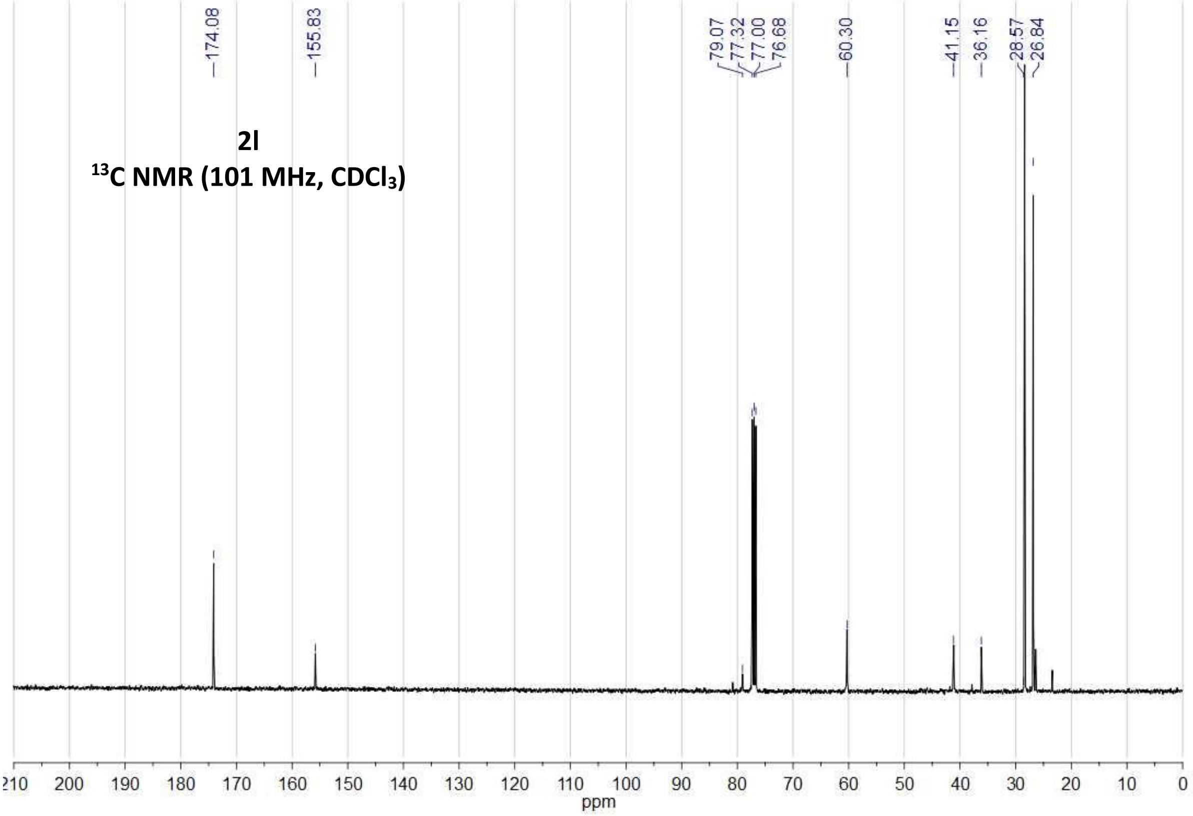
2l
^{13}C NMR (101 MHz, $CDCl_3$)
174.08
155.83
79.07 77.32 77.00 76.68
60.30
41.15
36.16
28.57
26.84
ppm

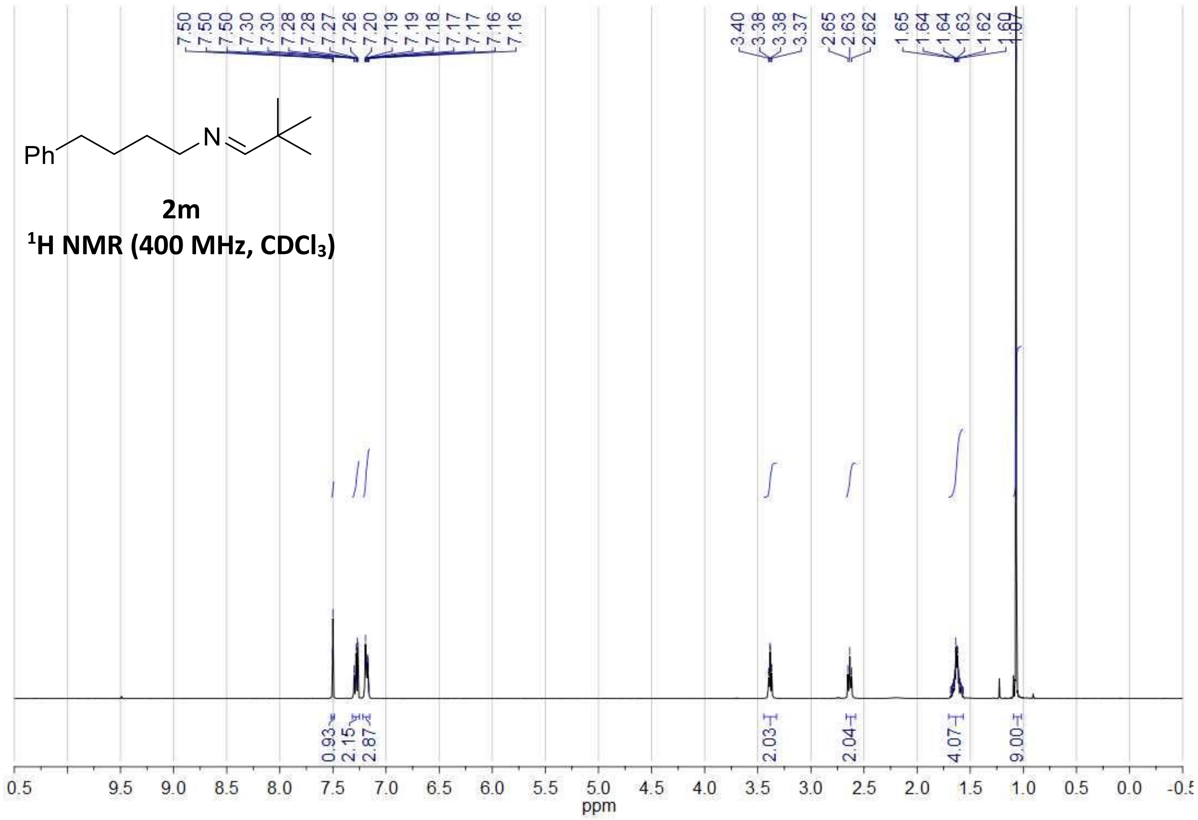
7.50
7.50
7.50
7.30
7.30
7.28
7.28
7.27
7.26
7.20
7.19
7.19
7.18
7.17
7.17
7.16
7.16
3.40
3.38
3.38
3.37
2.65
2.63
2.62
1.65
1.64
1.64
1.63
1.62
1.60
1.07
Ph
N
2m
1H NMR (400 MHz, CDCl3)
0.93
2.15
2.87
2.03
2.04
4.07
9.00
ppm

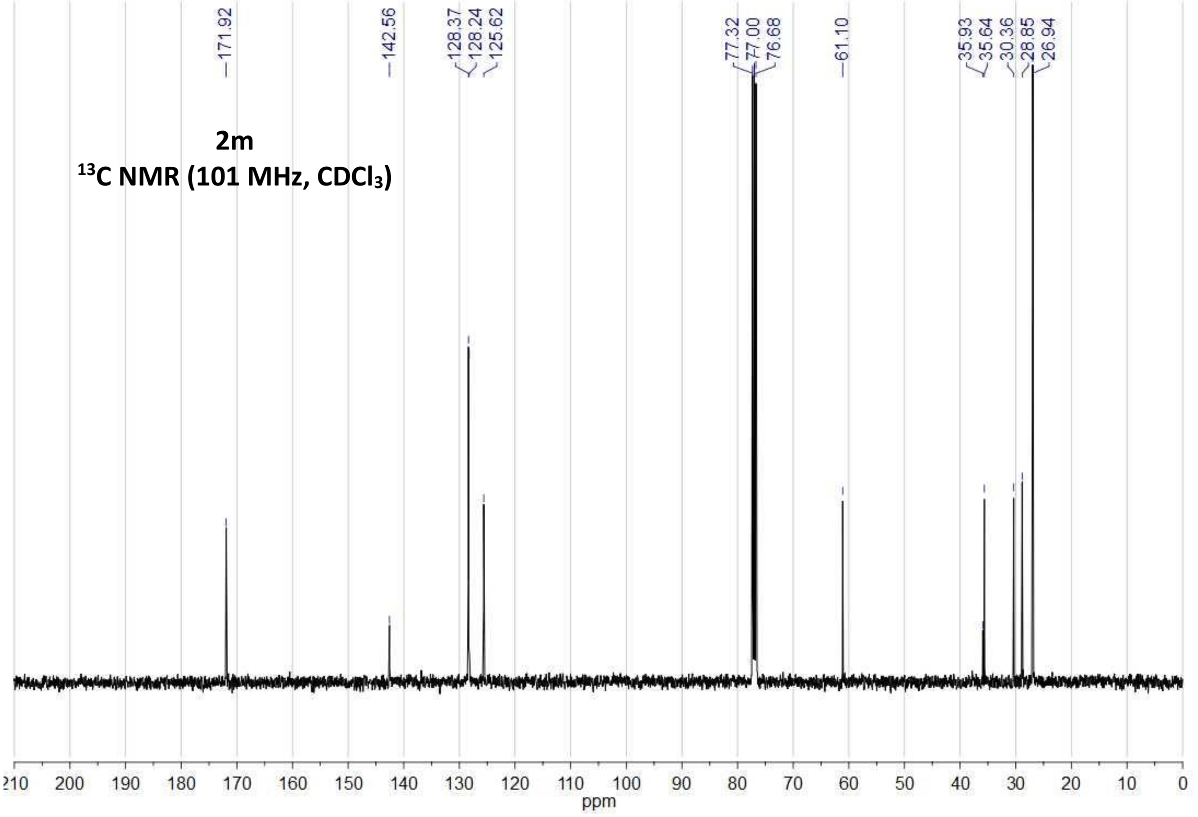
171.92
142.56
128.37
128.24
125.62
77.32
77.00
76.68
61.10
35.93
35.64
30.36
28.85
26.94
2m
13C NMR (101 MHz, CDCl3)
ppm

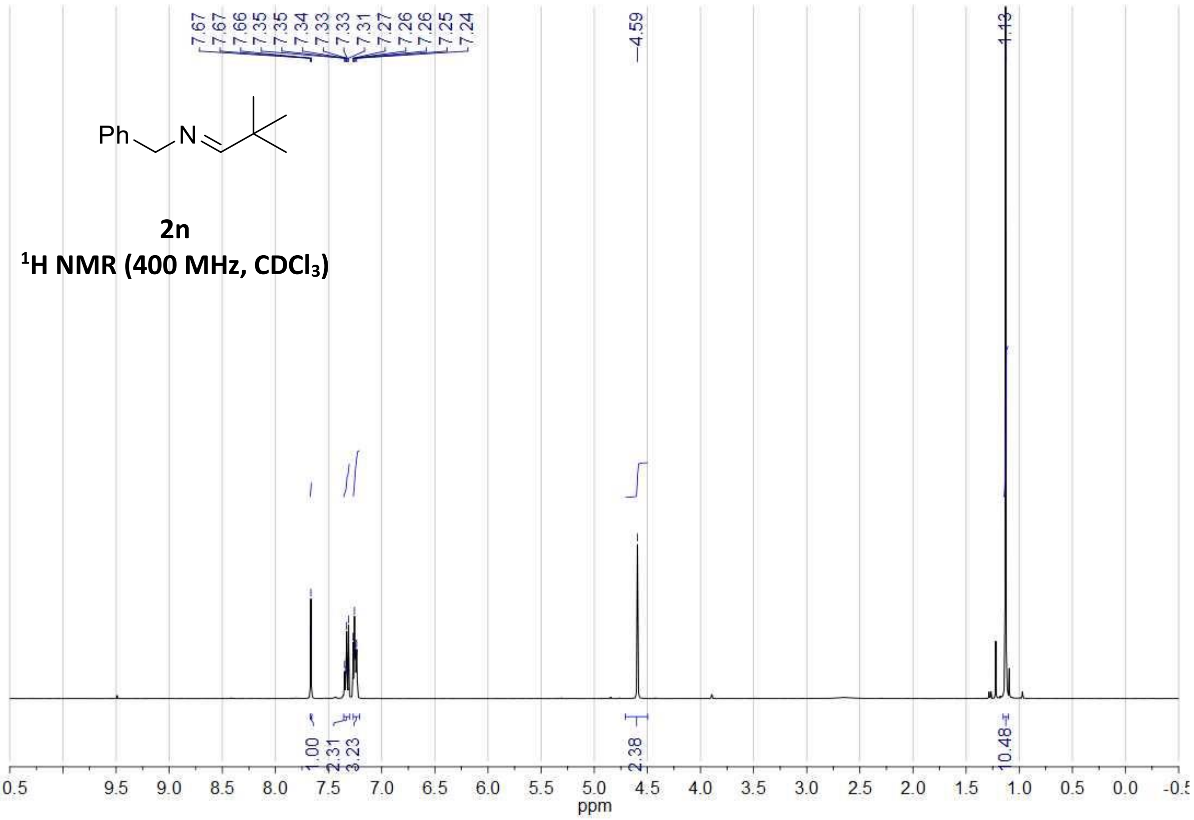

2n
^{1}H NMR (400 MHz, $CDCl_3$)

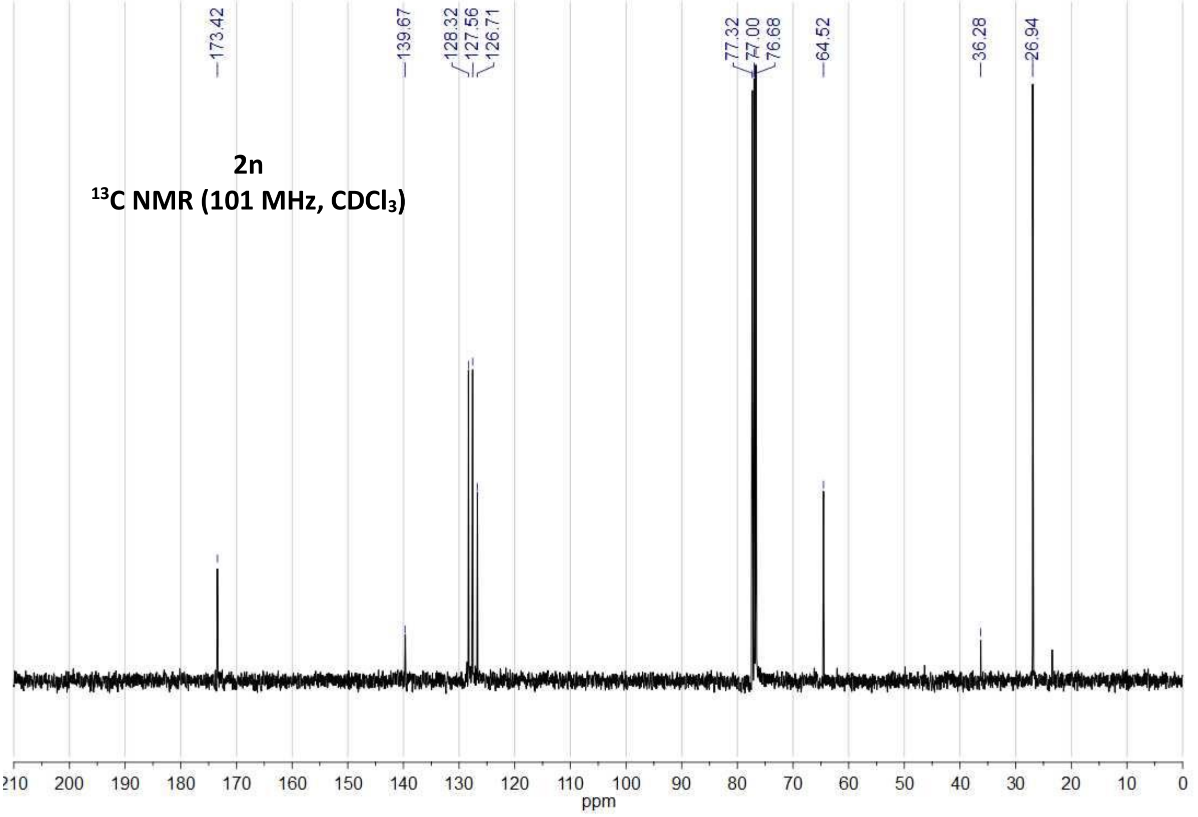

2n
^{13}C NMR (101 MHz, $CDCl_3$)

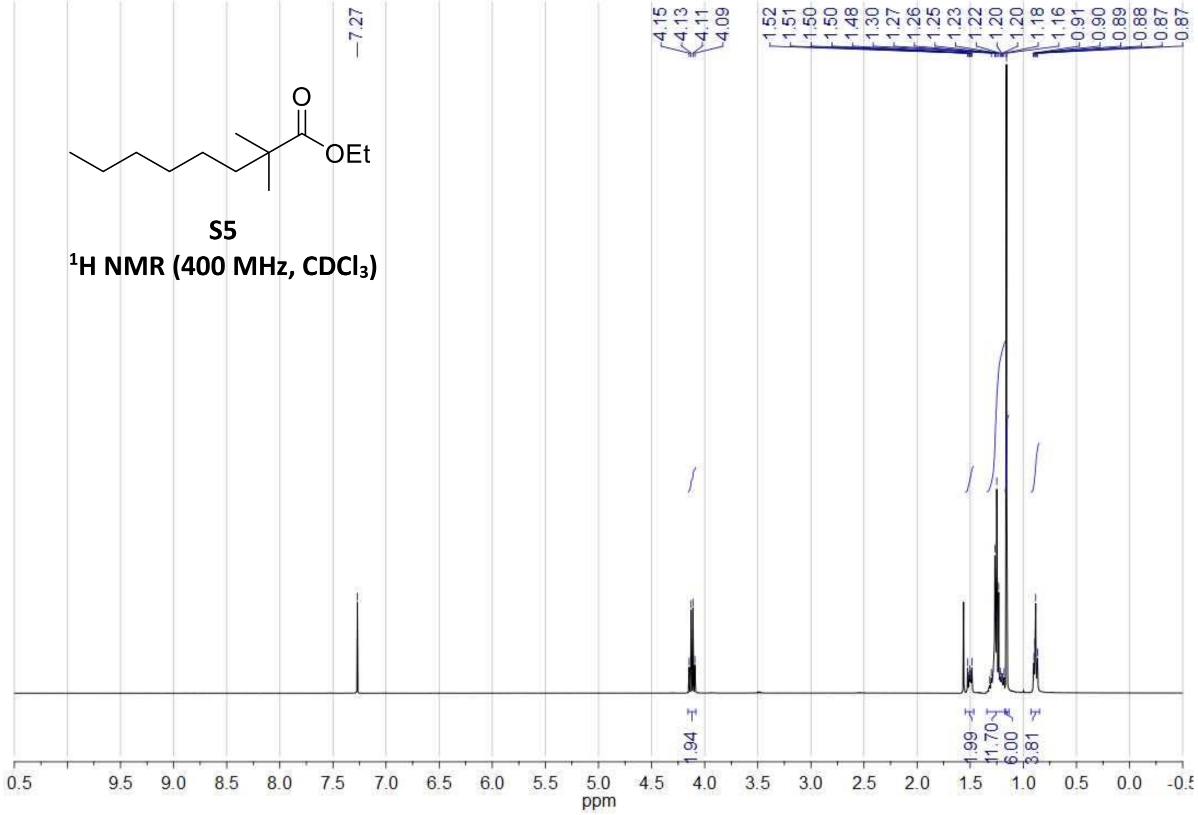
O
OEt
S5
^{1}H NMR (400 MHz, CDCl$_3$)
ppm

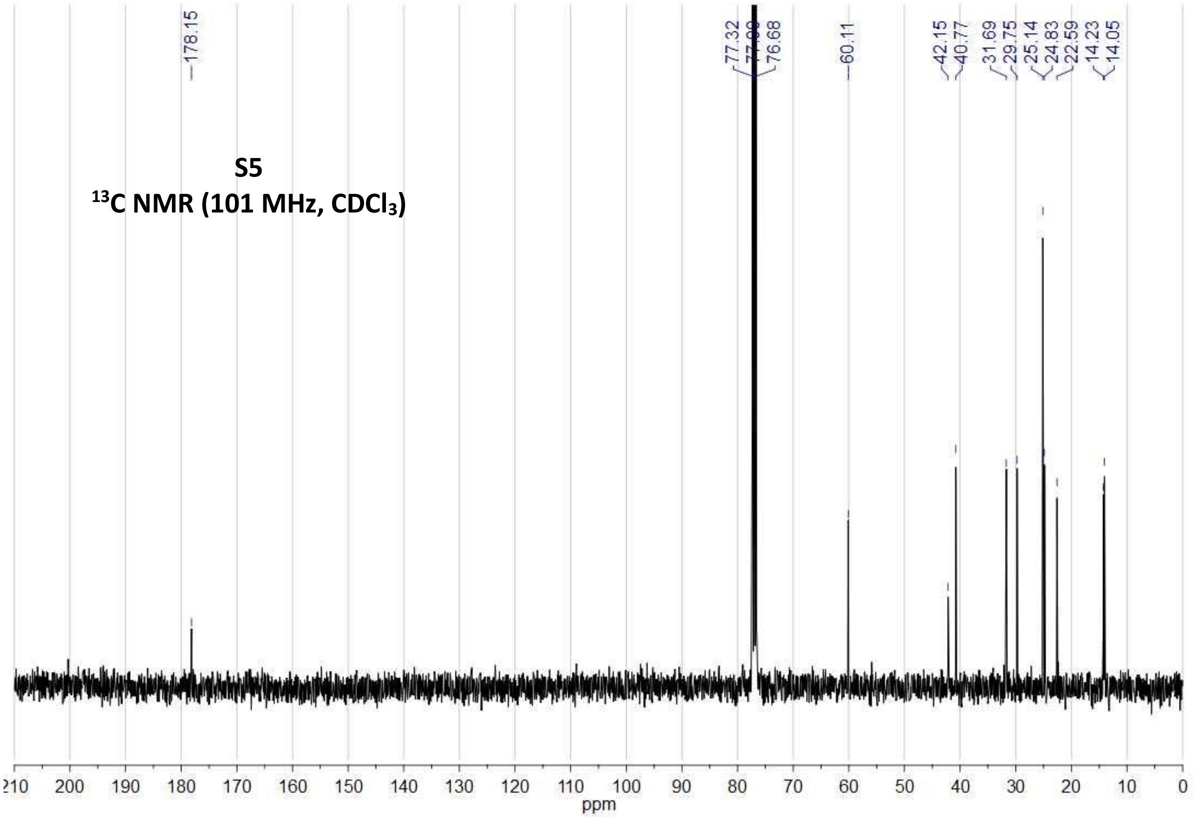
S5
^{13}C NMR (101 MHz, CDCl$_3$)
ppm

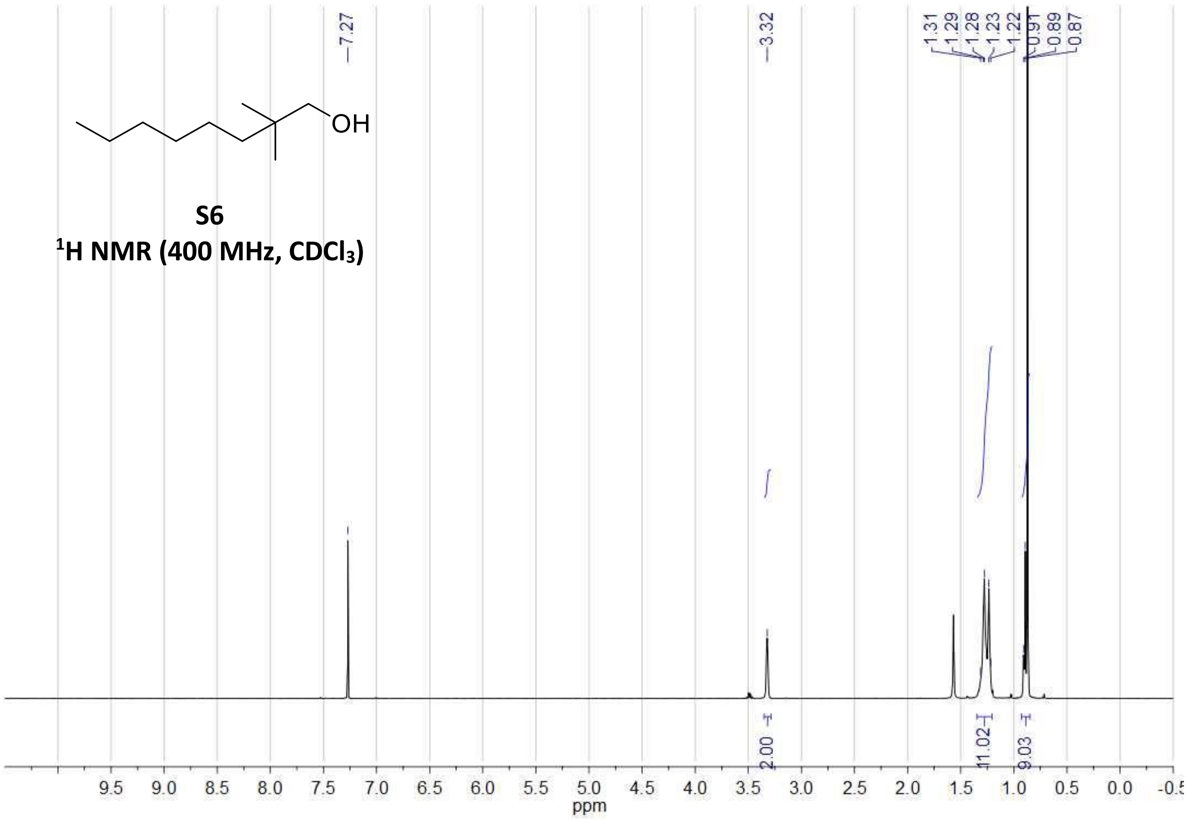

S6
^{1}H NMR (400 MHz, $CDCl_3$)

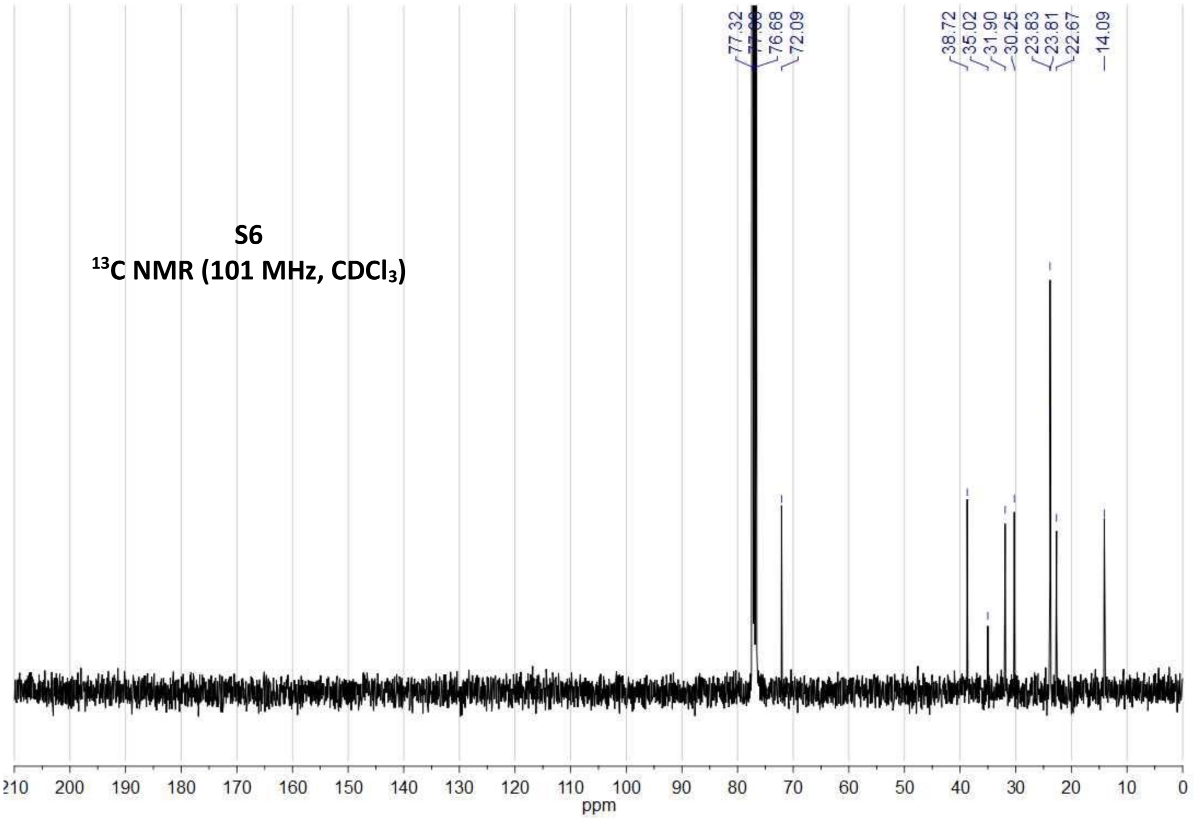

S6
^{13}C NMR (101 MHz, $CDCl_3$)

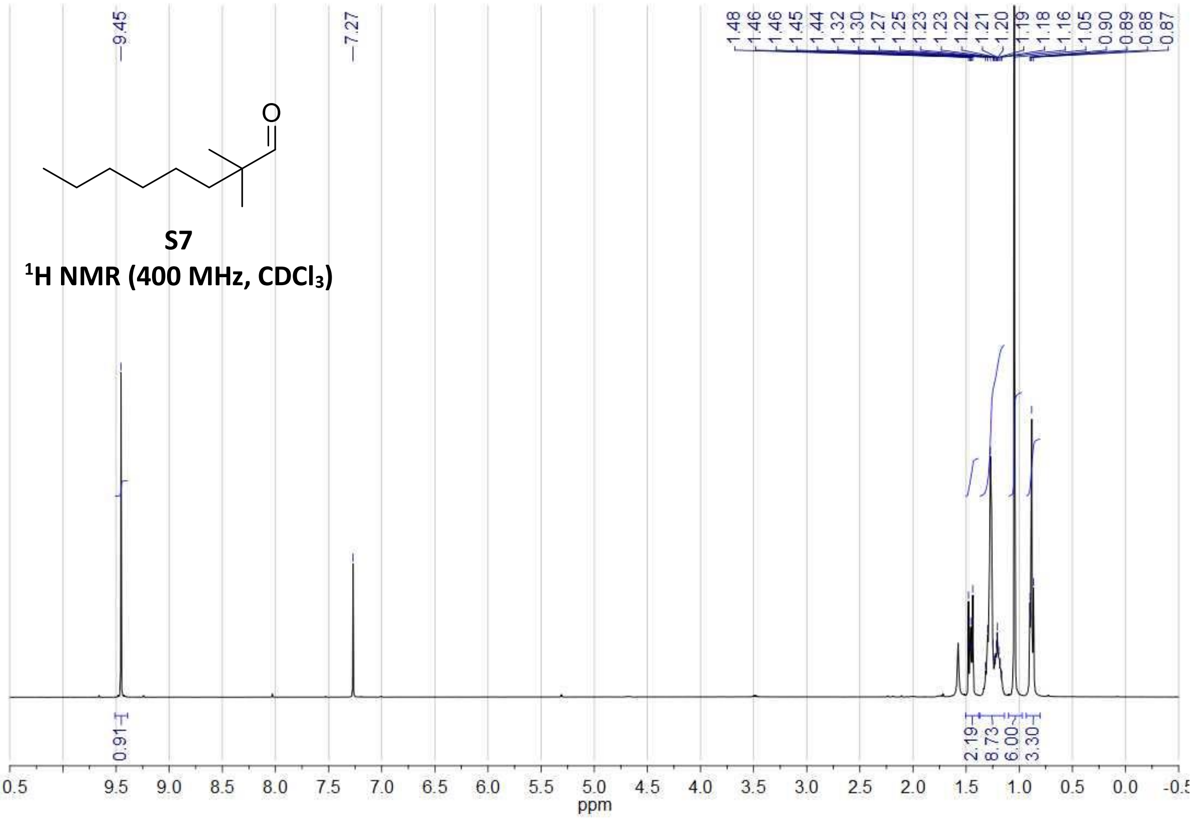
S7
^{1}H NMR (400 MHz, $CDCl_3$)
O
9.45
7.27
1.48 1.46 1.46 1.45 1.44 1.32 1.30 1.27 1.25 1.23 1.23 1.22 1.21 1.20 1.19 1.18 1.16 1.05 0.90 0.89 0.88 0.87
0.91
2.19 8.73 6.00 3.30
ppm

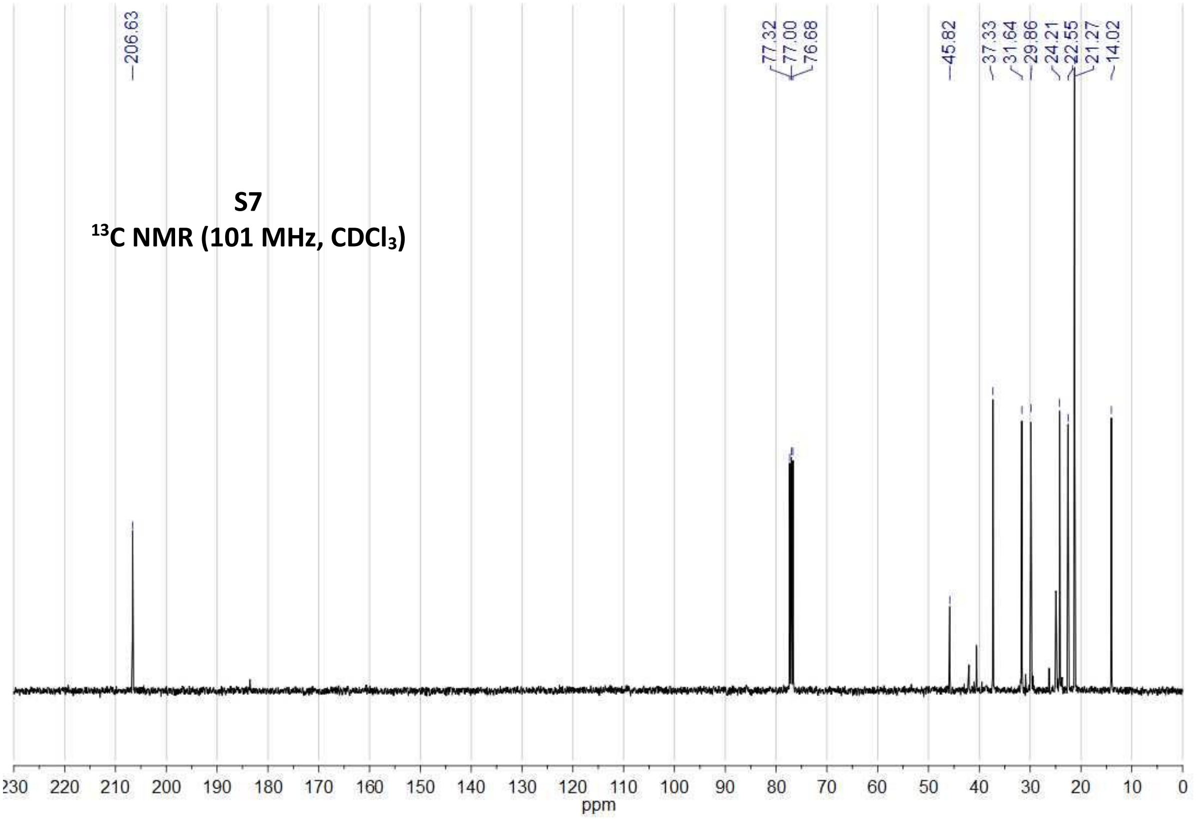
S7
^{13}C NMR (101 MHz, $CDCl_3$)
206.63
77.32 77.00 76.68
45.82
37.33 31.64 29.86 24.21 22.55 21.27 14.02
ppm

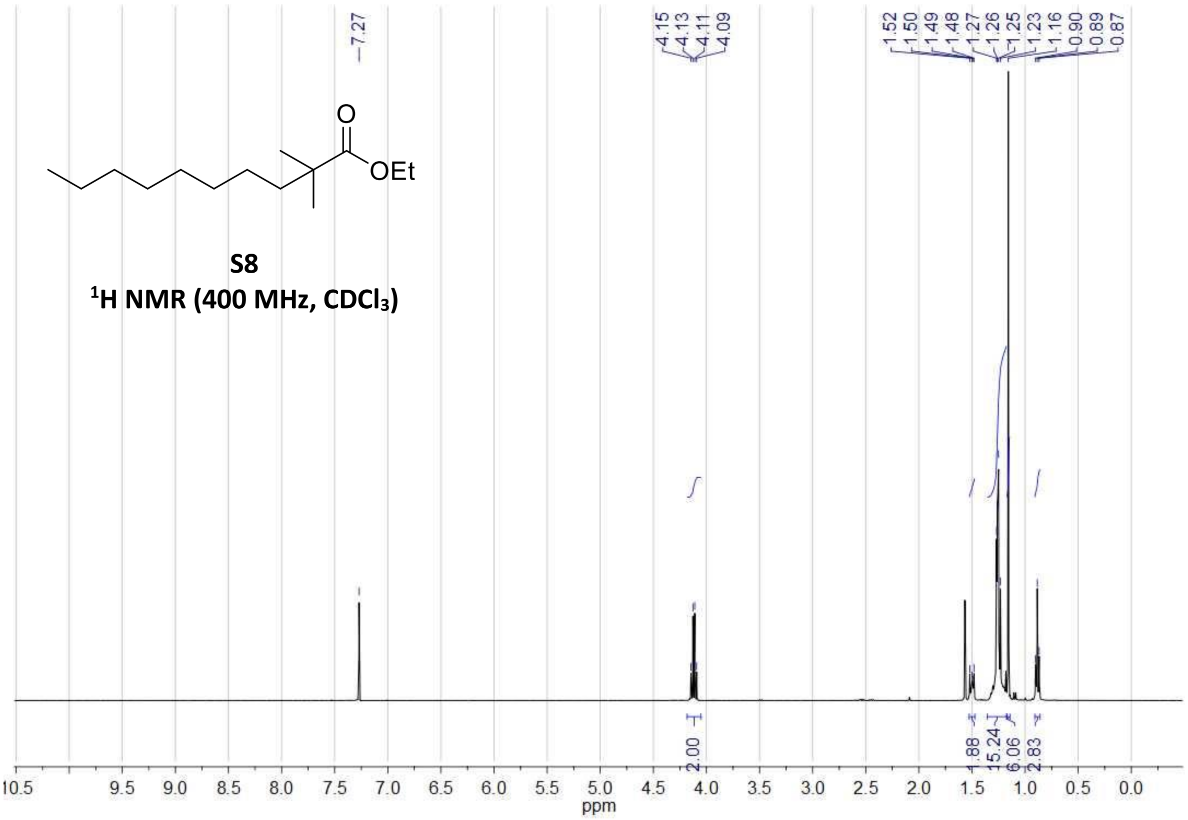

S8
^{1}H NMR (400 MHz, CDCl$_3$)

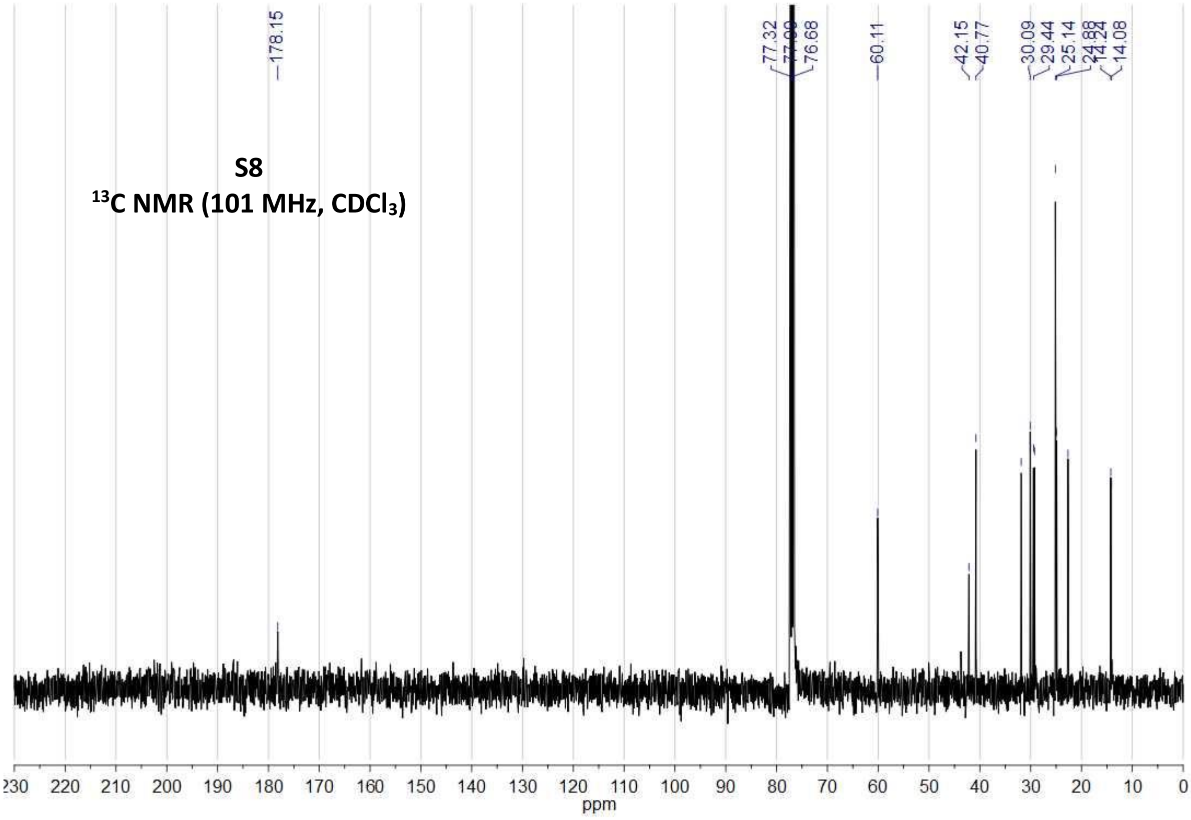

S8
^{13}C NMR (101 MHz, CDCl$_3$)

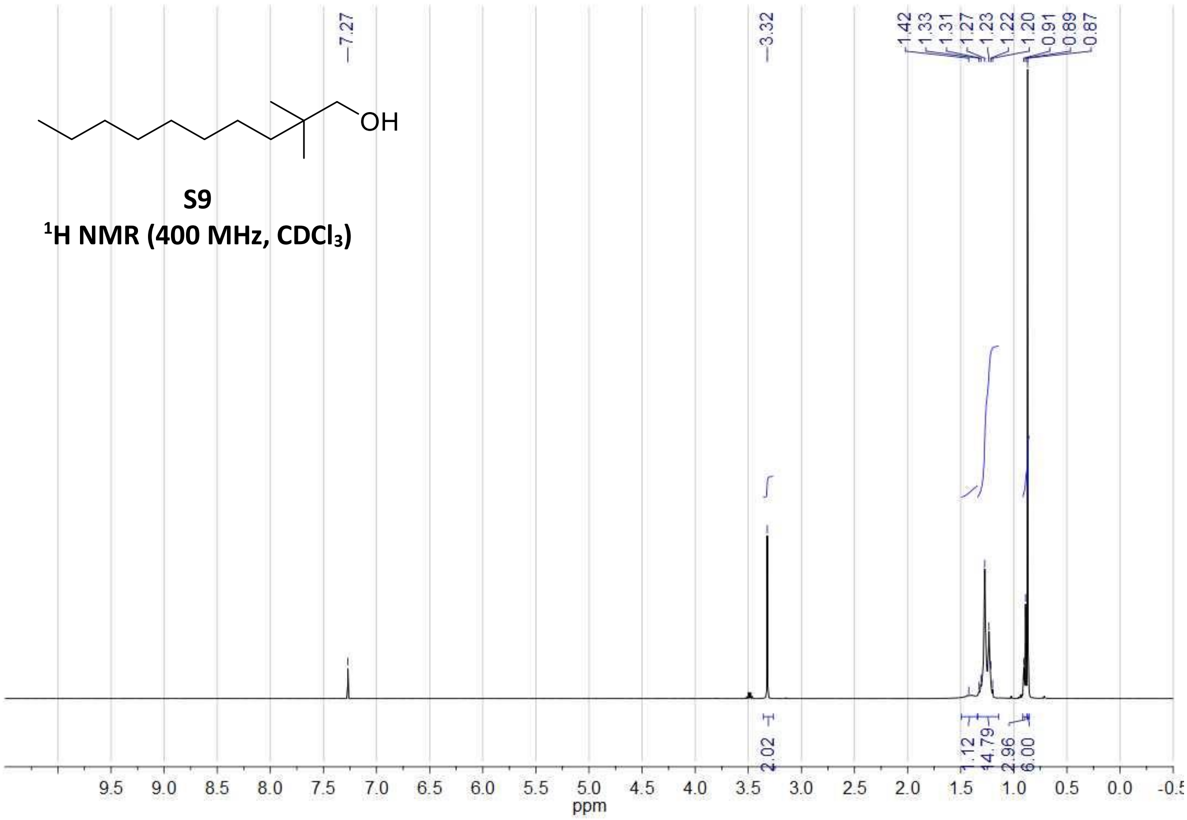

S9
^{1}H NMR (400 MHz, $CDCl_3$)

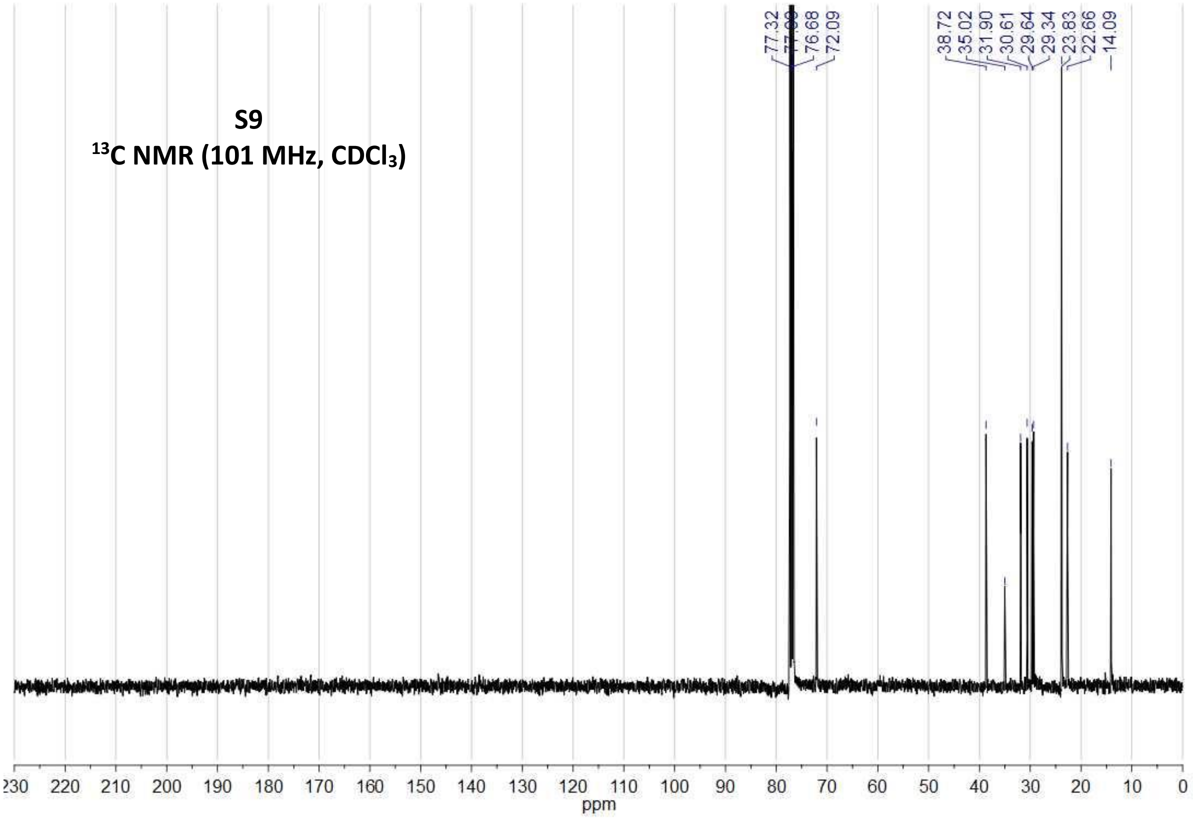

S9
^{13}C NMR (101 MHz, $CDCl_3$)

S10

^{1}H NMR (400 MHz, $CDCl_3$)

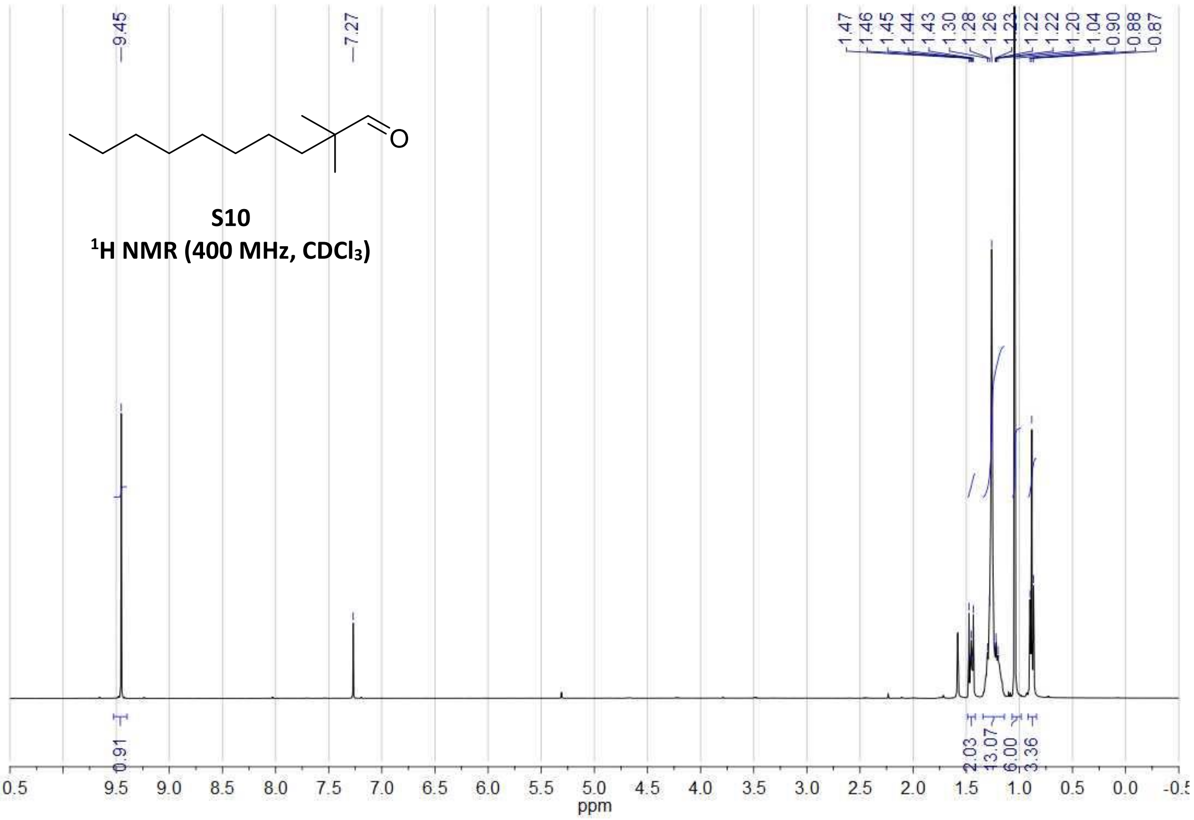

S10

^{13}C NMR (101 MHz, $CDCl_3$)

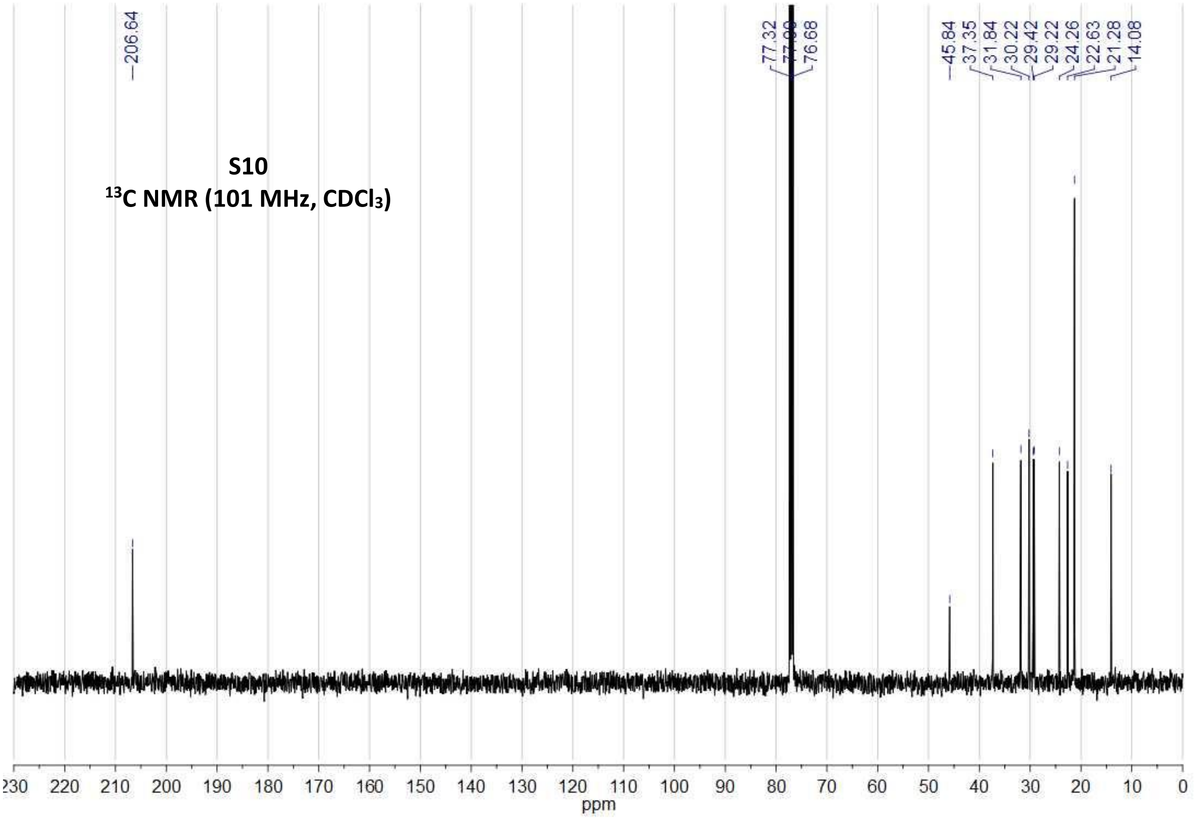

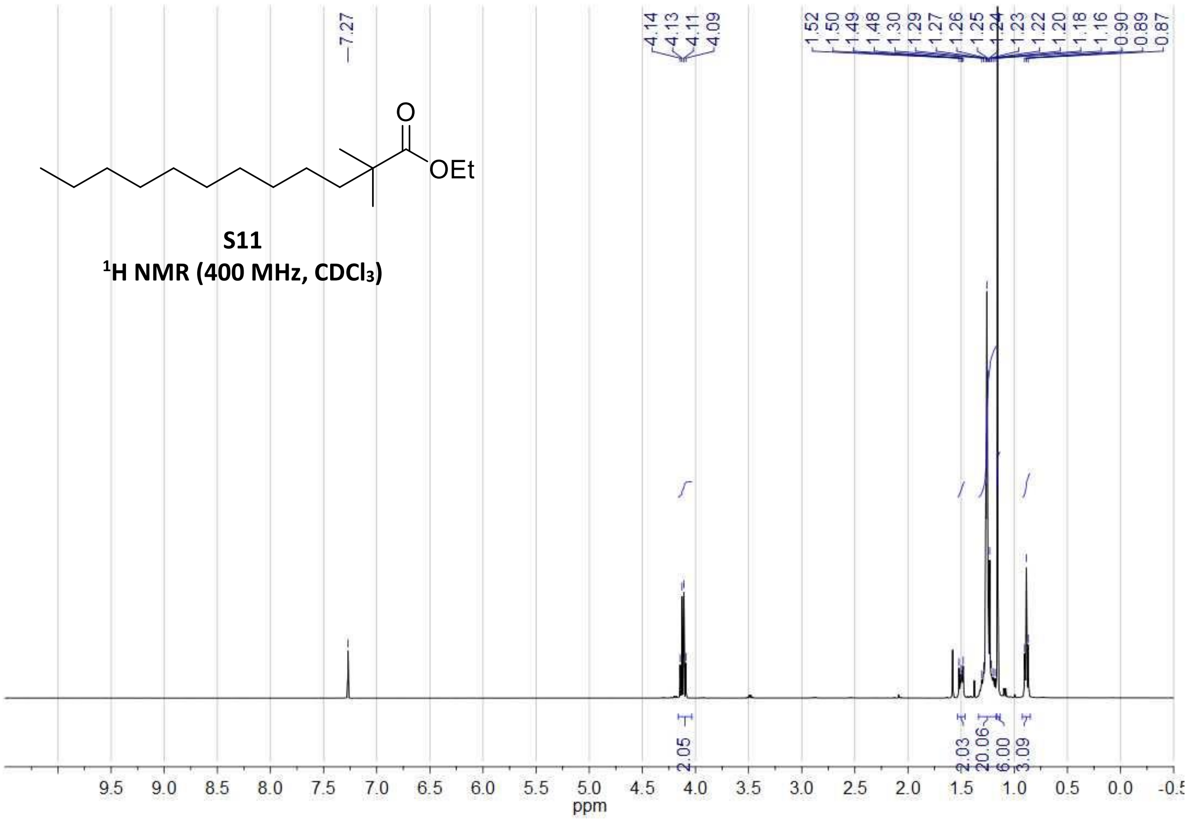

S11
^{1}H NMR (400 MHz, $CDCl_3$)

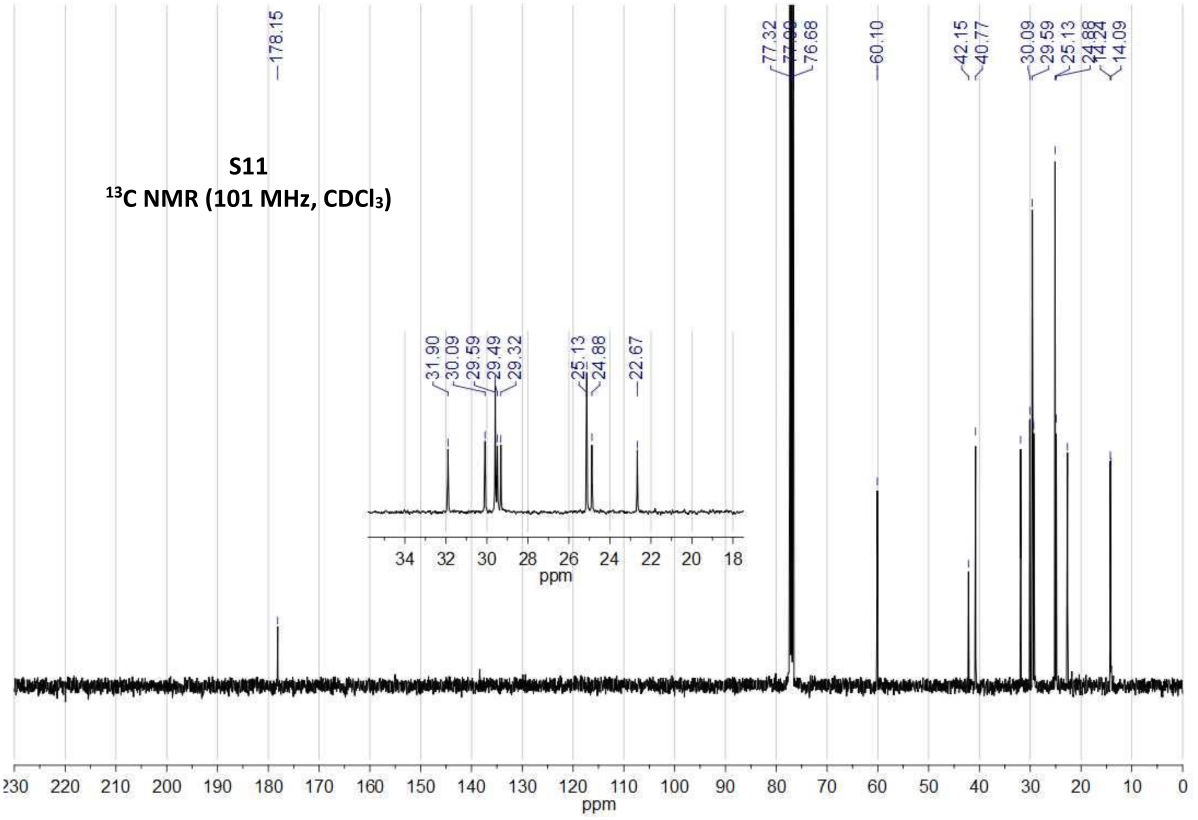

S11
^{13}C NMR (101 MHz, $CDCl_3$)

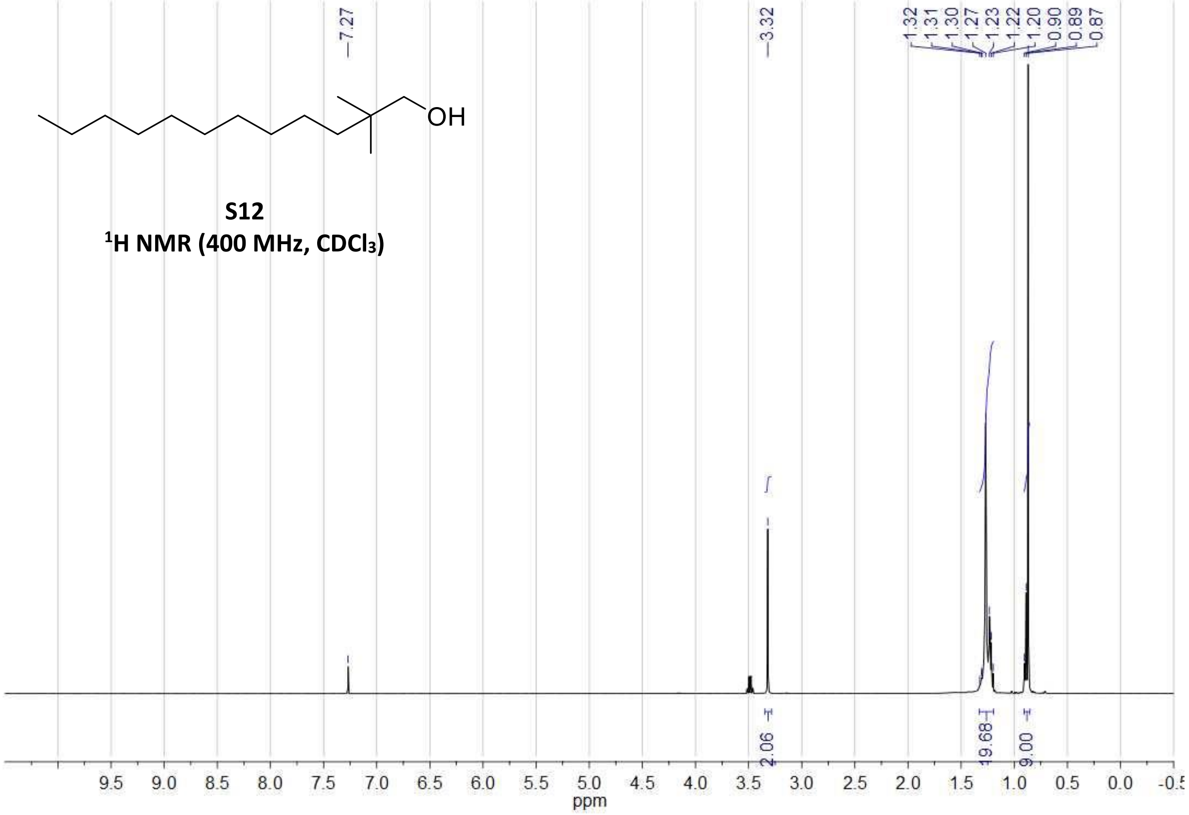

S12
^{1}H NMR (400 MHz, $CDCl_3$)

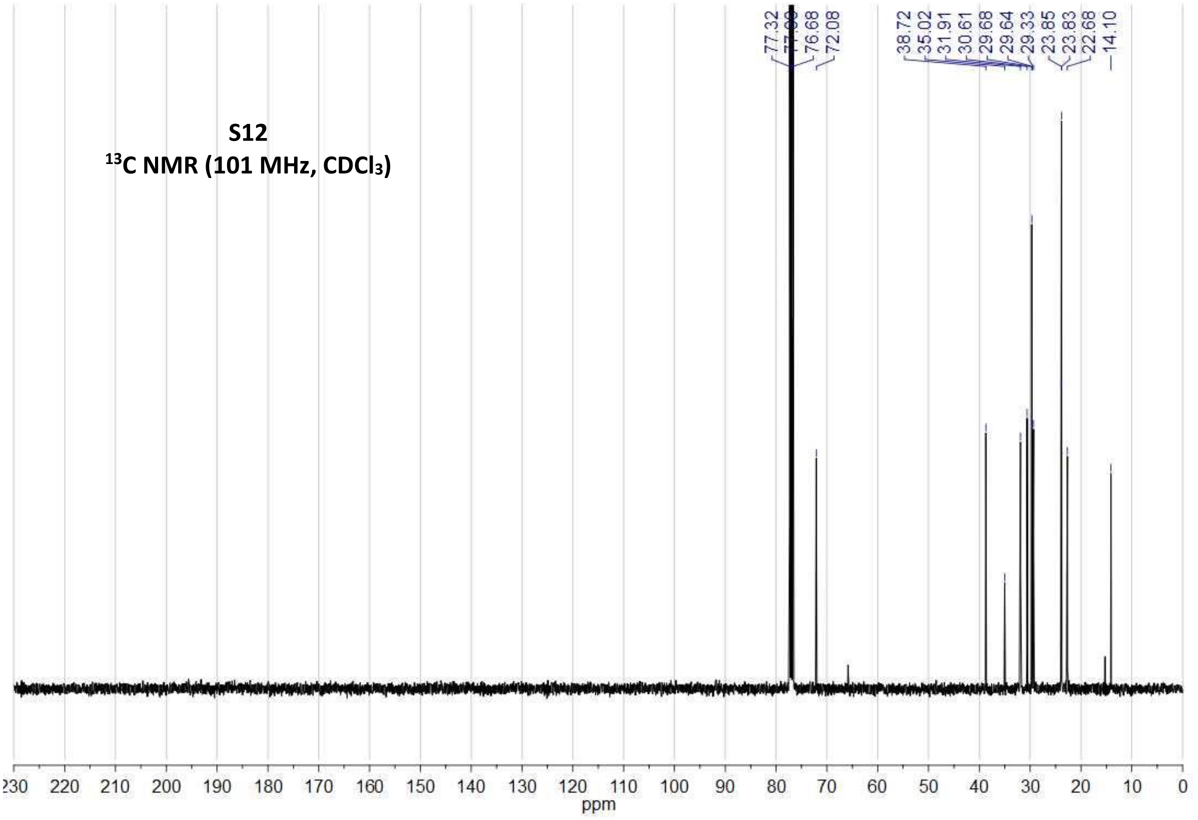

S12
^{13}C NMR (101 MHz, $CDCl_3$)

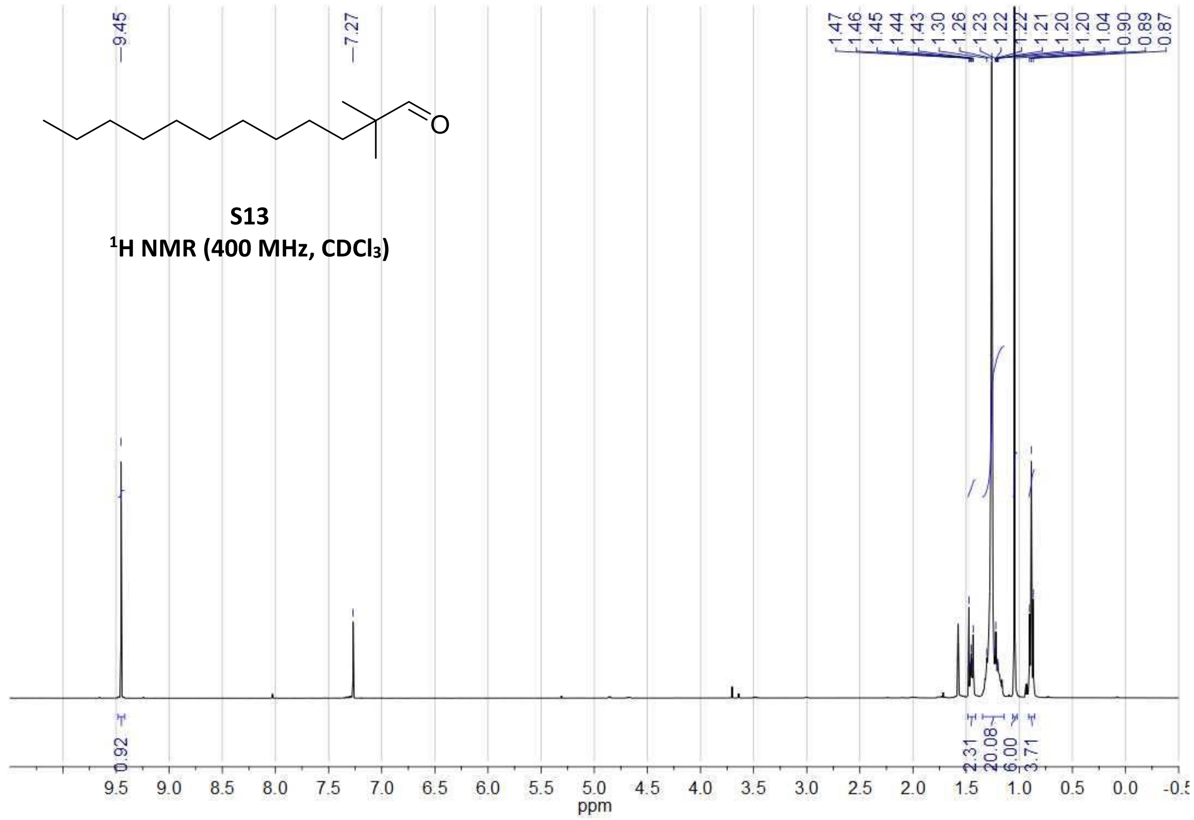
S13
1H NMR (400 MHz, CDCl3)
ppm

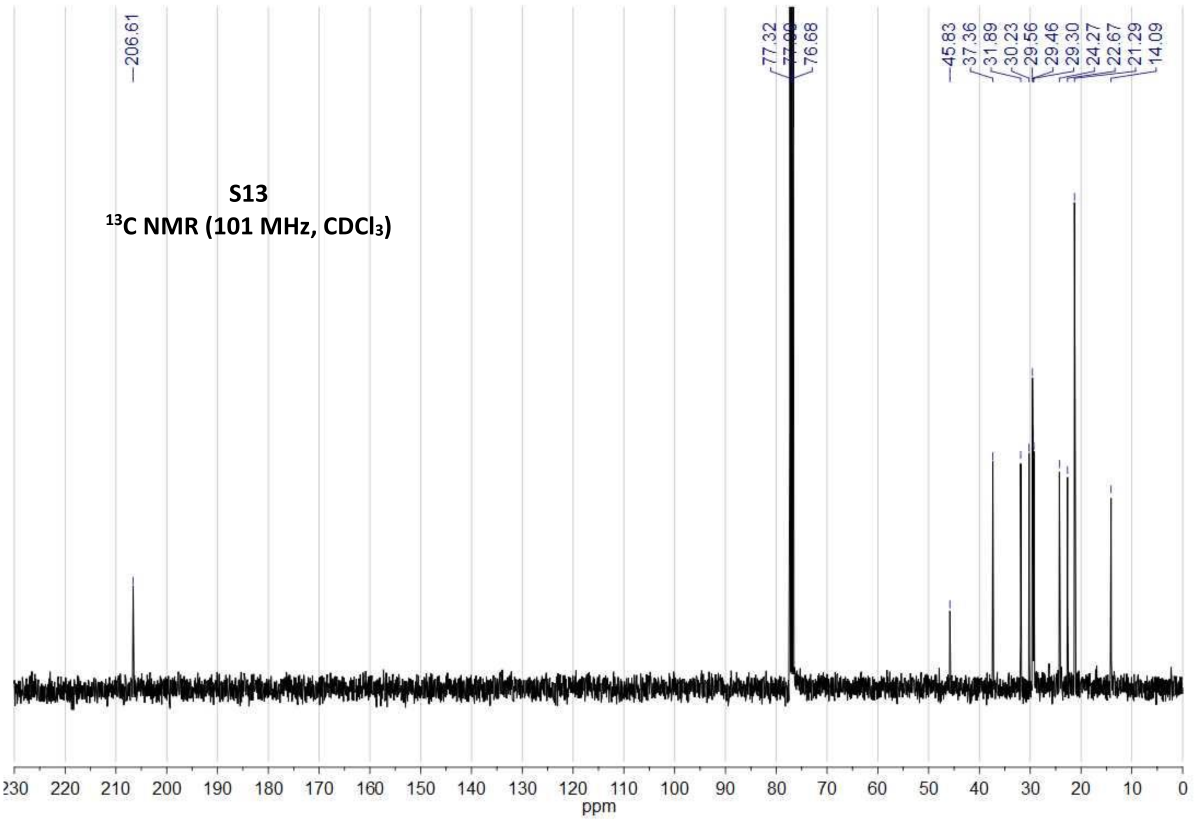
S13
13C NMR (101 MHz, CDCl3)
ppm

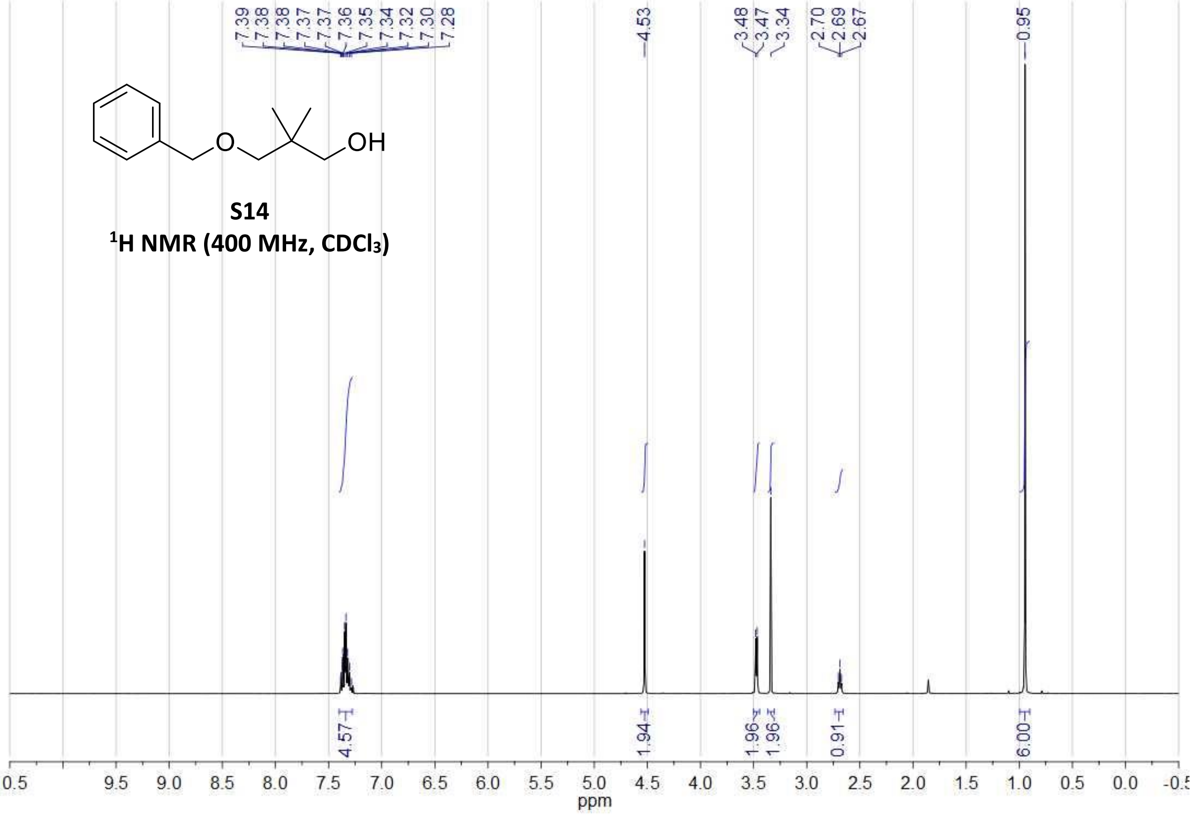
S14
1H NMR (400 MHz, CDCl3)
OH
O
ppm

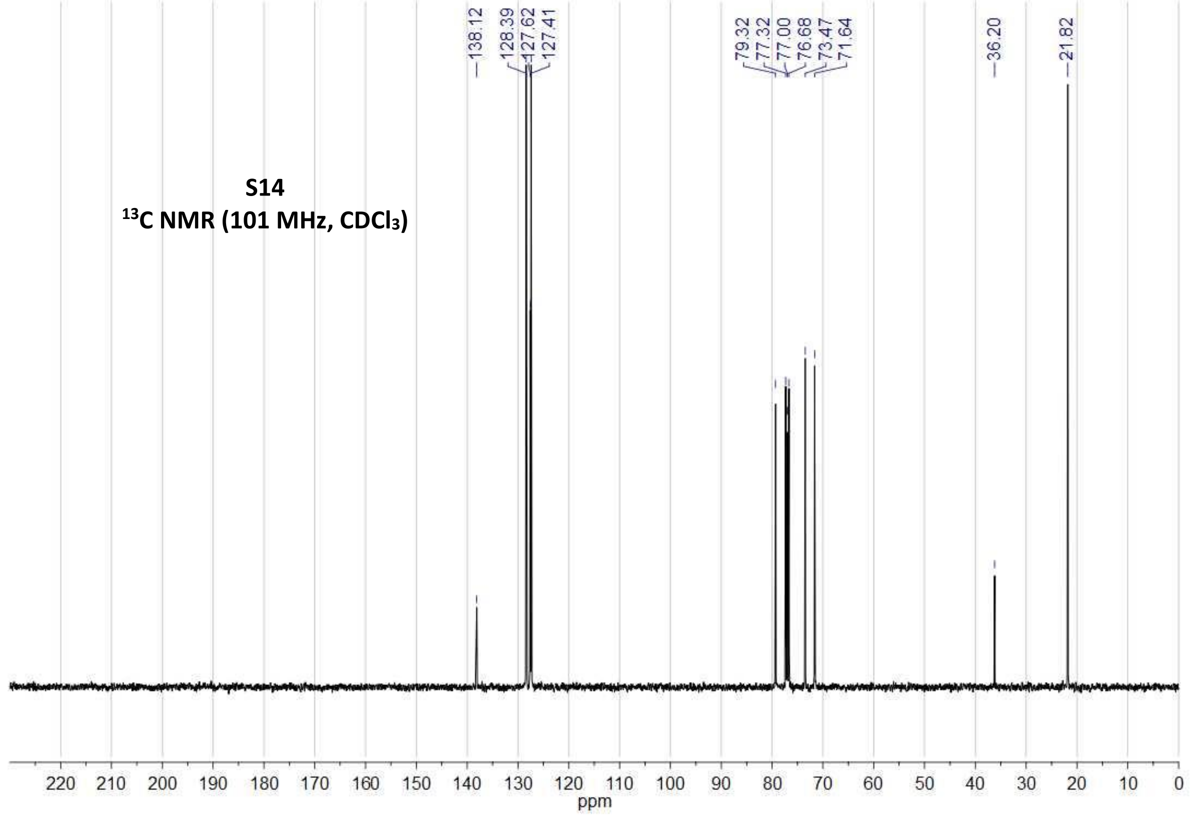
S14
13C NMR (101 MHz, CDCl3)
ppm

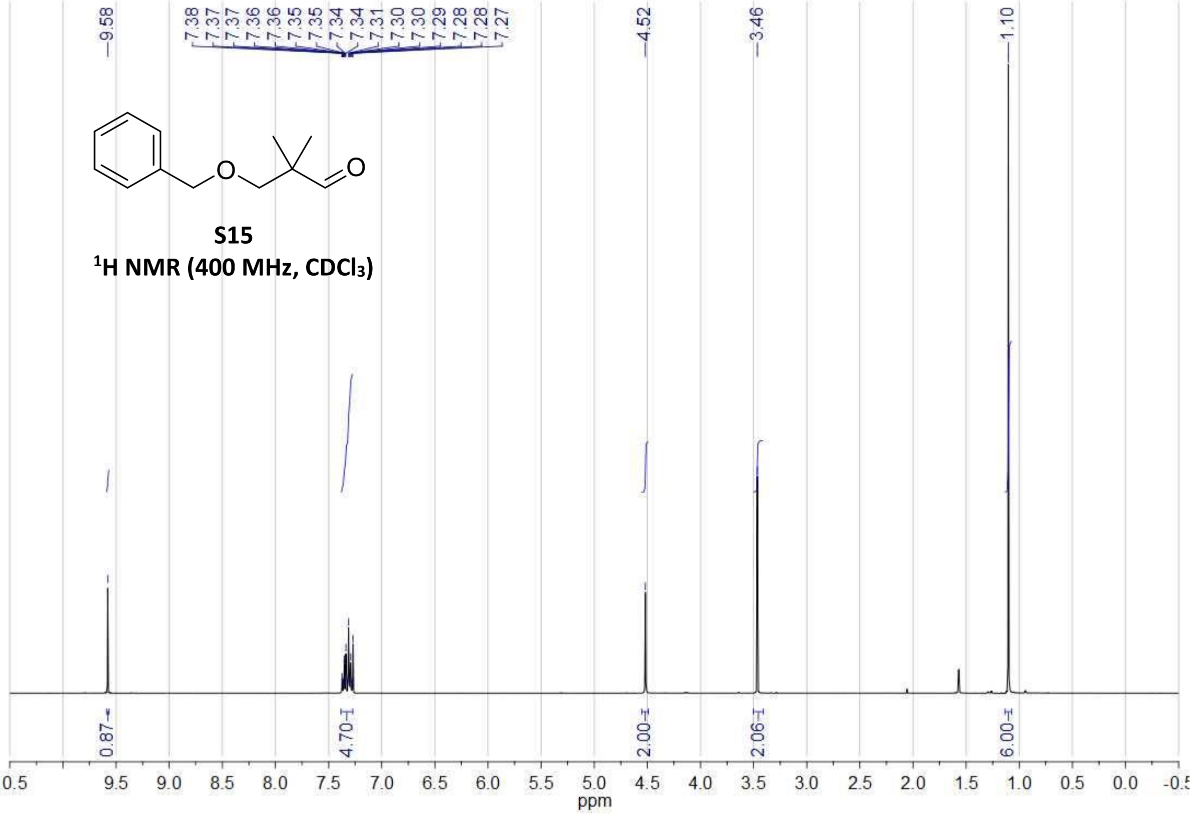
9.58
7.38
7.37
7.37
7.36
7.36
7.35
7.35
7.34
7.34
7.31
7.30
7.30
7.29
7.28
7.28
7.27
4.52
3.46
1.10
O
O
S15
^{1}H NMR (400 MHz, $CDCl_3$)
0.87
4.70
2.00
2.06
6.00
ppm

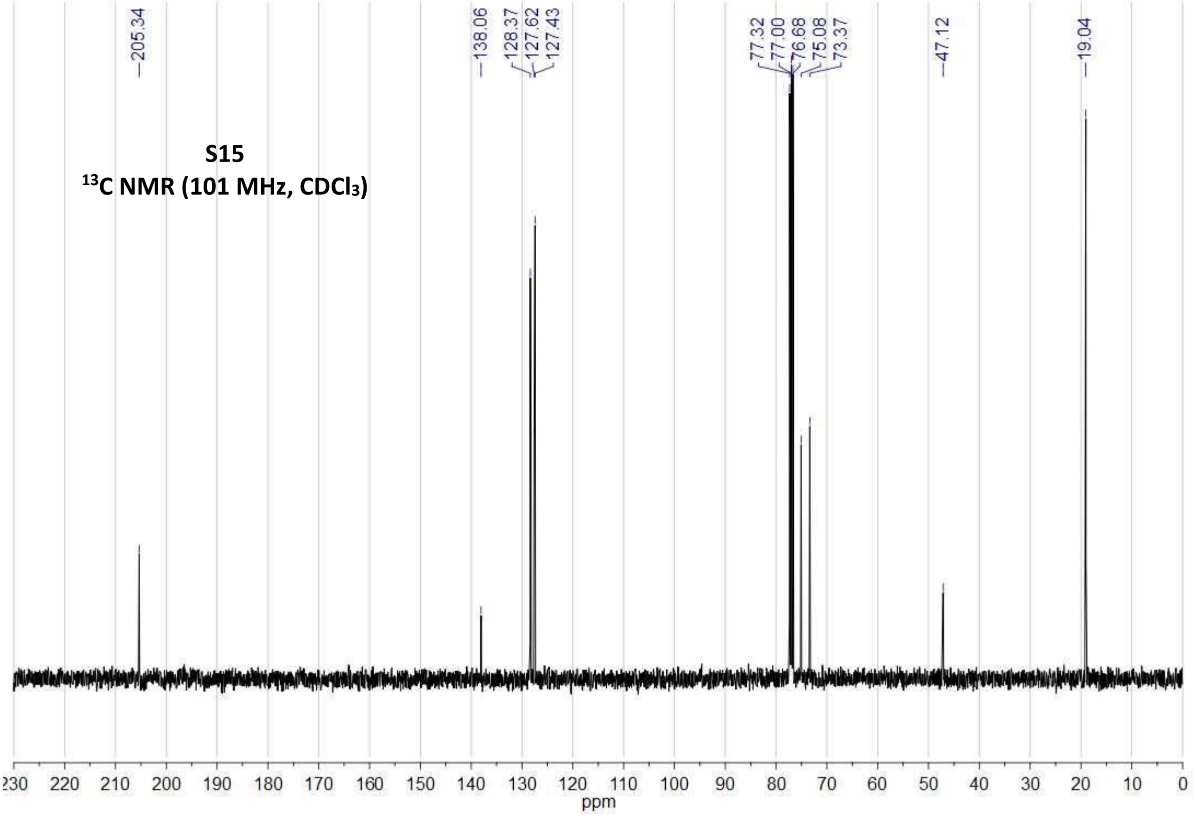
205.34
138.06
128.37
127.62
127.43
77.32
77.00
76.68
75.08
73.37
47.12
19.04
S15
^{13}C NMR (101 MHz, $CDCl_3$)
ppm

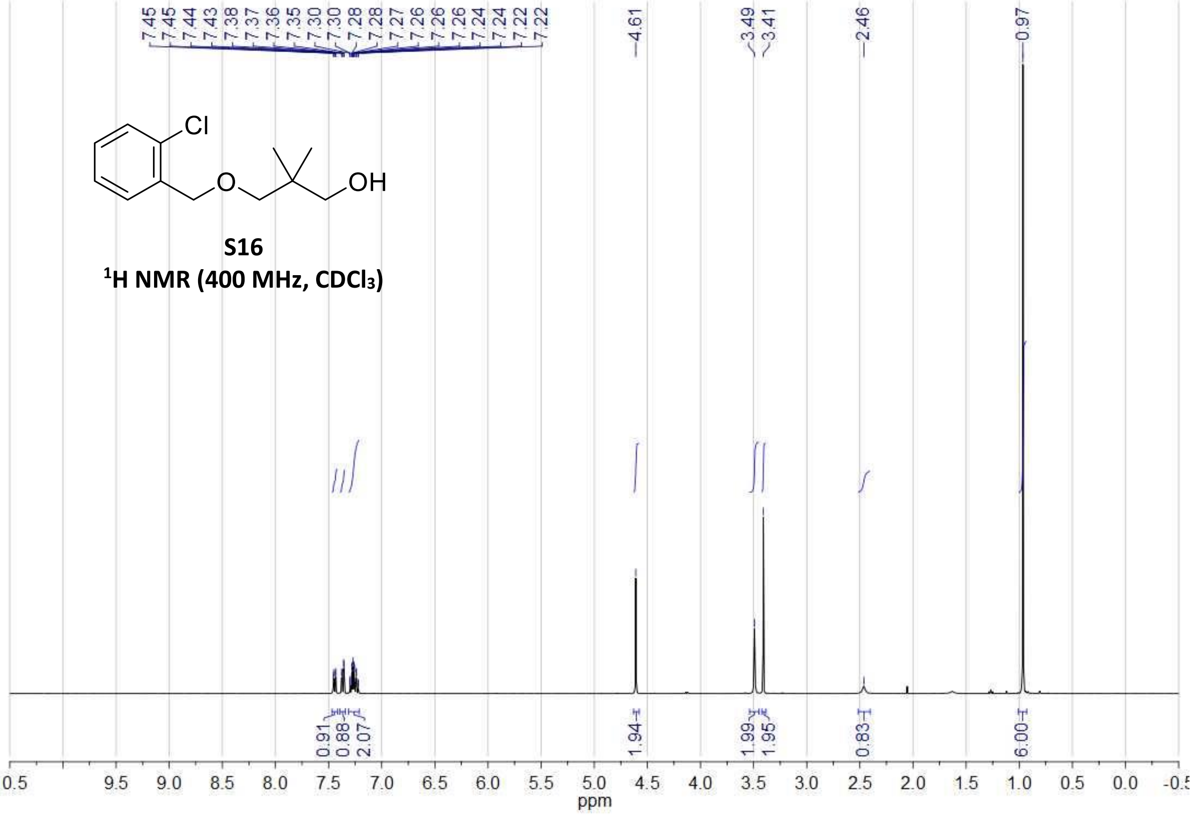

S16
^{1}H NMR (400 MHz, CDCl₃)

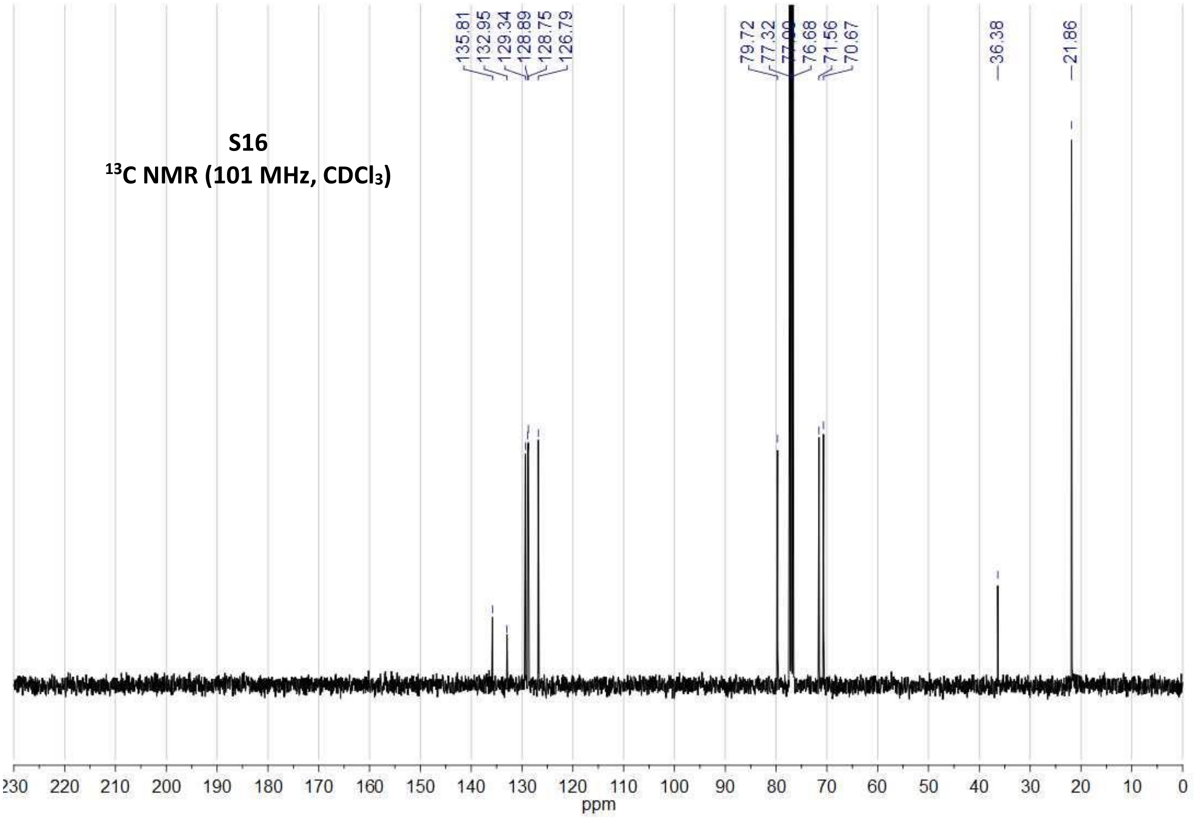

S16
^{13}C NMR (101 MHz, CDCl₃)

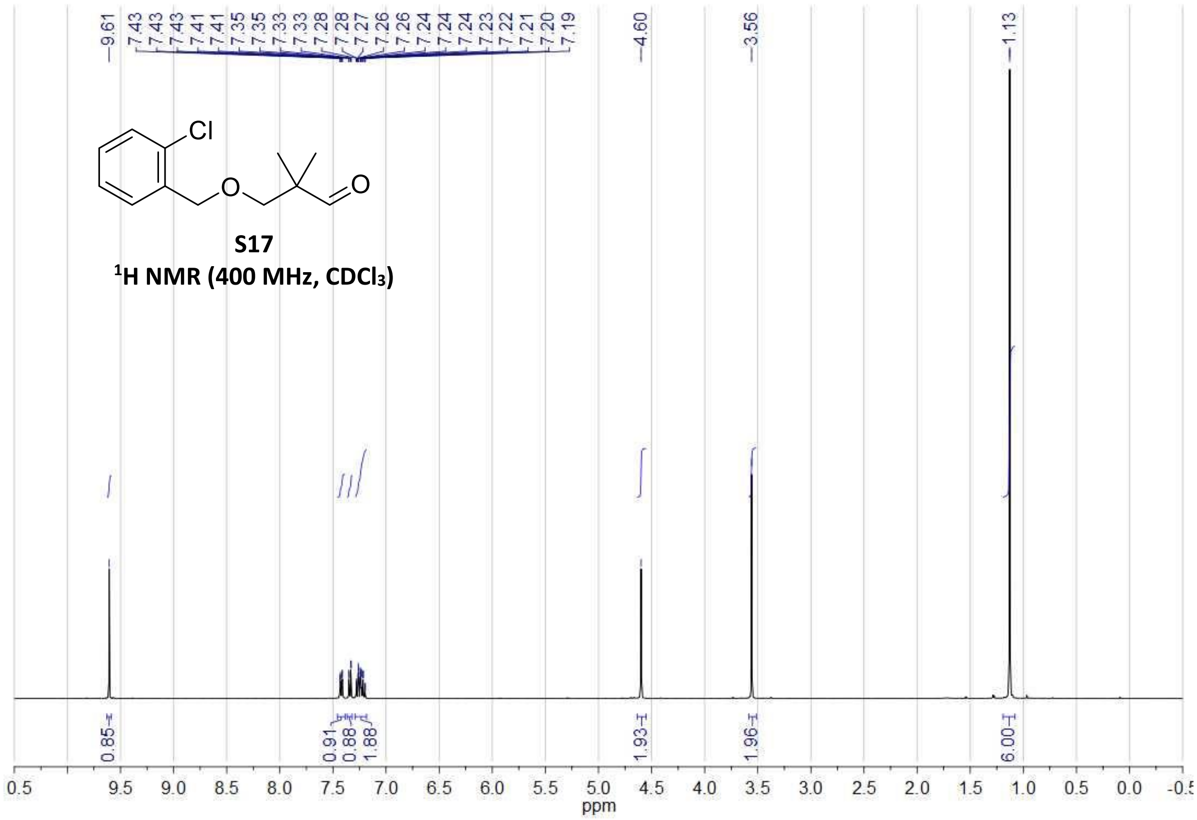

S17

^{1}H NMR (400 MHz, $CDCl_3$)

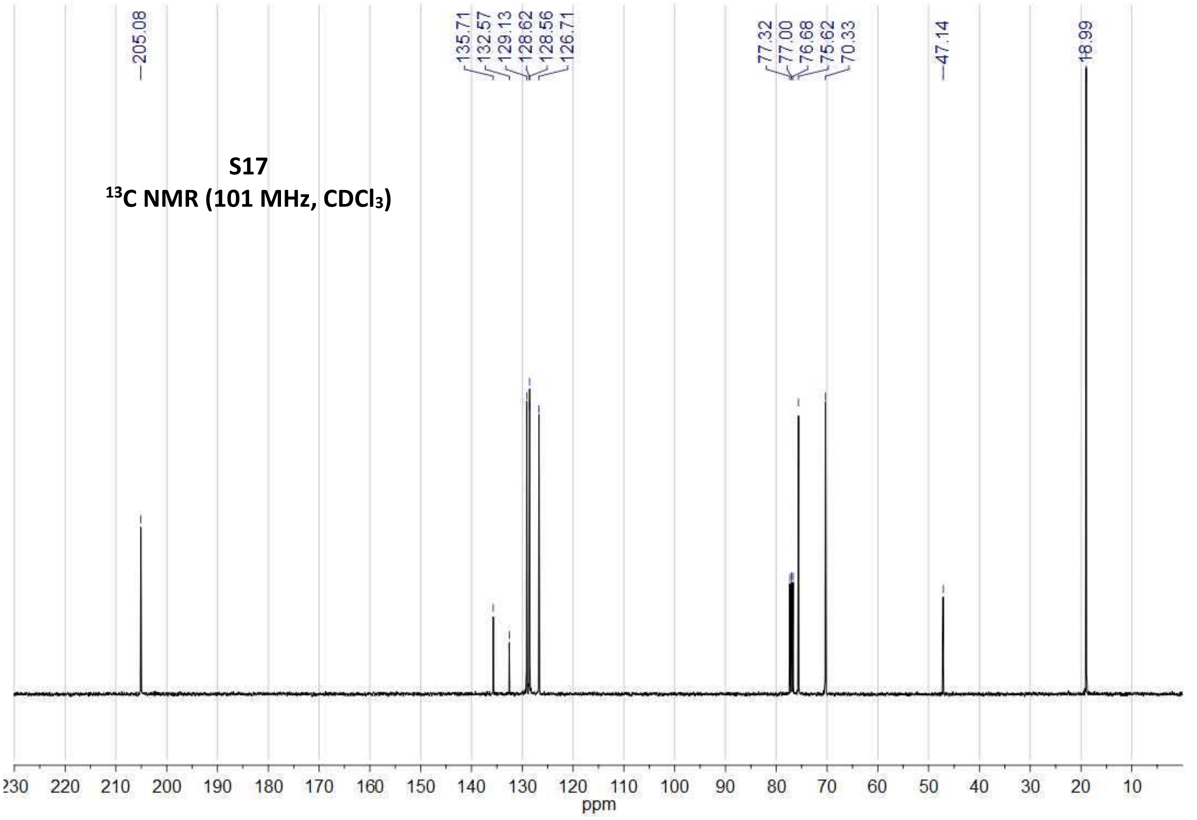

S17

^{13}C NMR (101 MHz, $CDCl_3$)

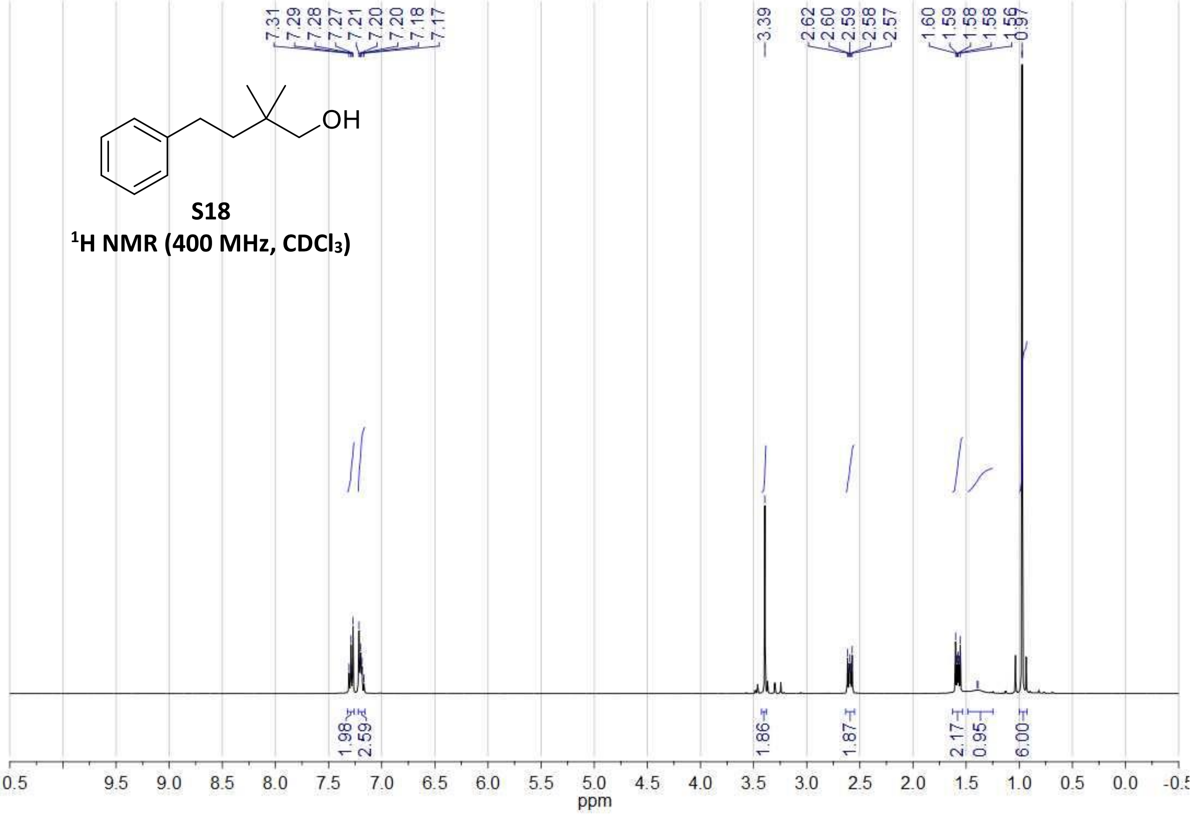

OH
S18
^{1}H NMR (400 MHz, CDCl3)
7.31 7.29 7.28 7.27 7.21 7.20 7.20 7.18 7.17
3.39
2.62 2.60 2.59 2.58 2.57
1.60 1.59 1.58 1.58 1.56 0.97
1.98 2.59 1.86 1.87 2.17 0.95 6.00
ppm

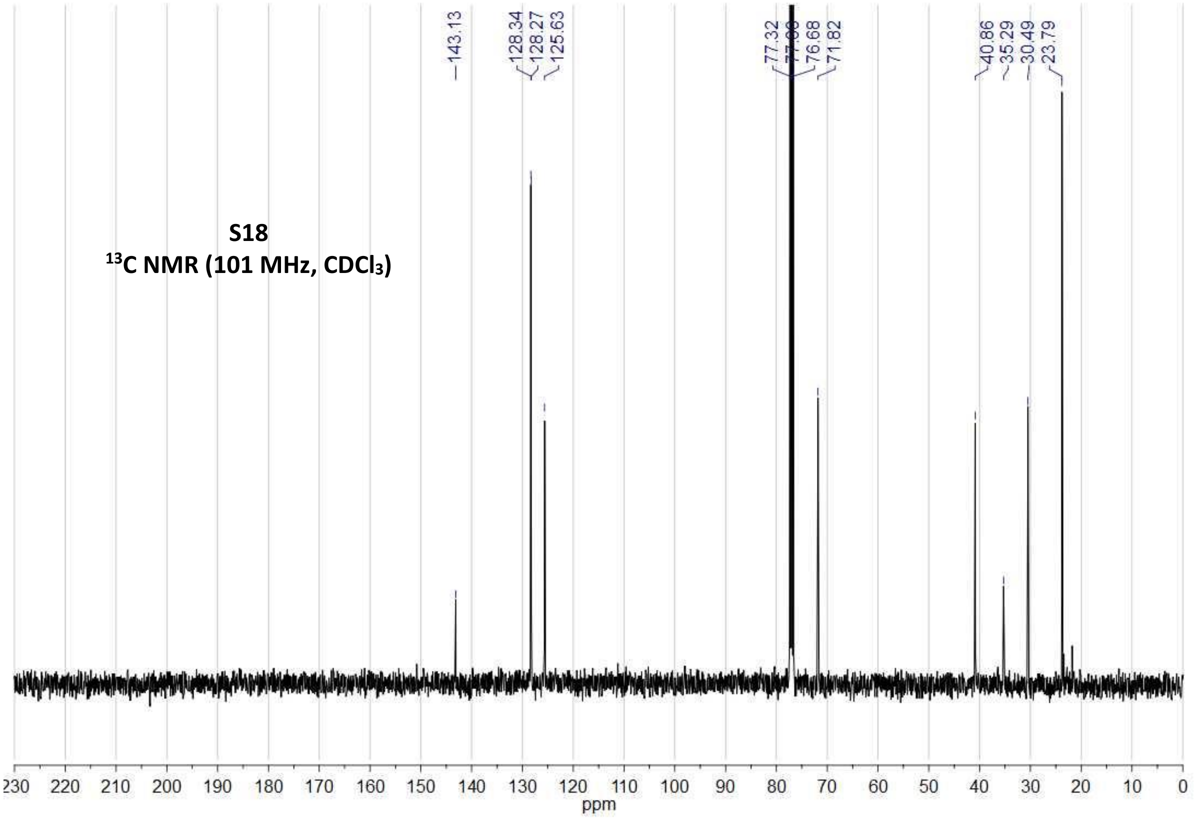

143.13
128.34 128.27 125.63
77.32 76.68 71.82
40.86 35.29 30.49 23.79
S18
^{13}C NMR (101 MHz, CDCl3)
ppm

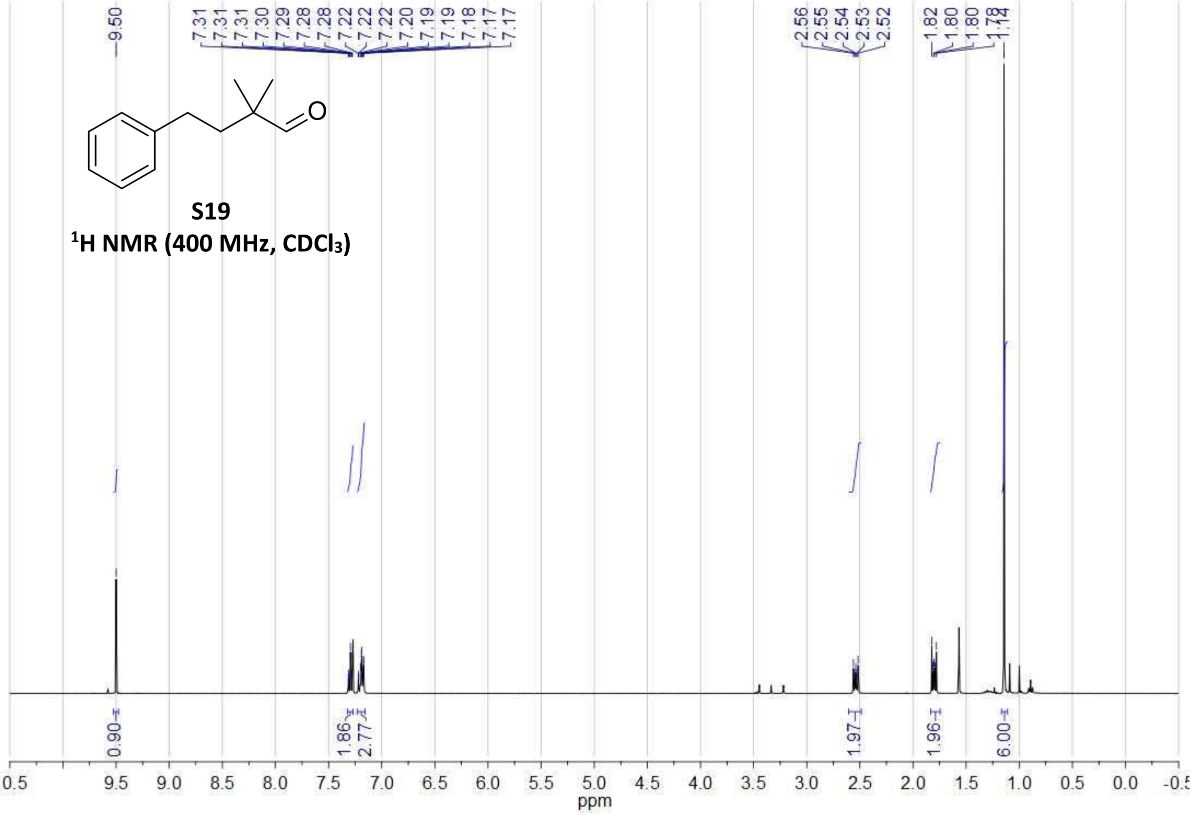
S19
1H NMR (400 MHz, CDCl3)
9.50
7.31 7.31 7.31 7.30 7.29 7.28 7.28 7.22 7.22 7.22 7.20 7.19 7.19 7.18 7.17 7.17
2.56 2.55 2.54 2.53 2.52
1.82 1.80 1.80 1.78 1.14
0.90 1.86 2.77 1.97 1.96 6.00
ppm

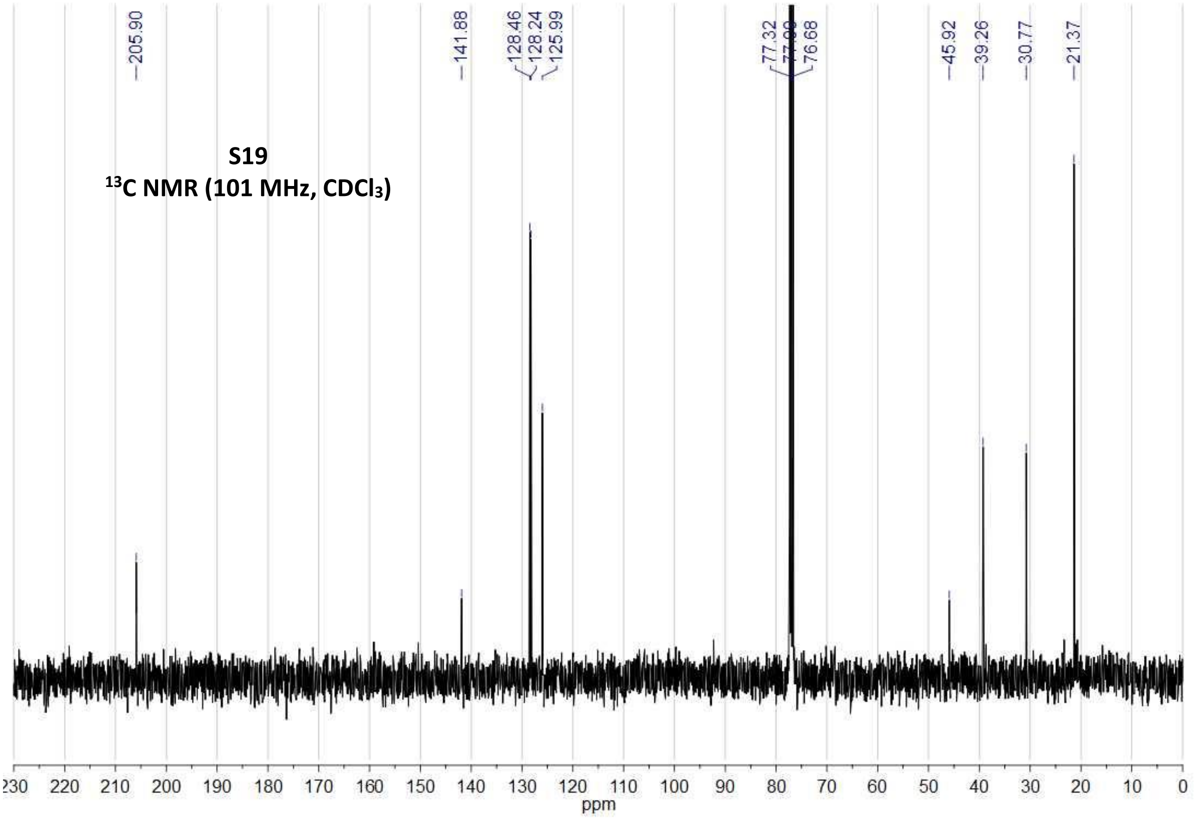
S19
13C NMR (101 MHz, CDCl3)
205.90
141.88
128.46 128.24 125.99
77.32 77.00 76.68
45.92
39.26
30.77
21.37
ppm

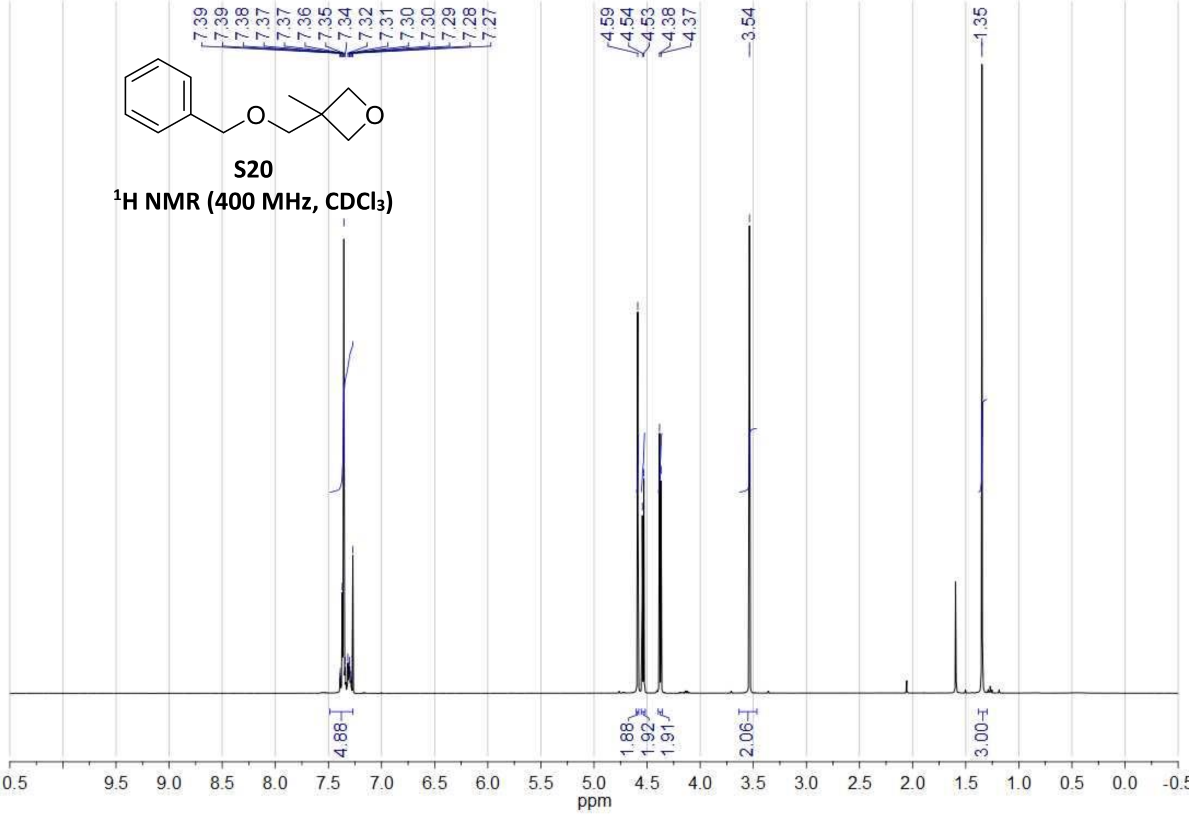
7.39 7.39 7.38 7.37 7.37 7.36 7.35 7.34 7.32 7.31 7.30 7.30 7.29 7.28 7.27
4.59 4.54 4.53 4.38 4.37
3.54
1.35
S20
1H NMR (400 MHz, CDCl3)
4.88
1.88 1.92 1.91
2.06
3.00
0.5 9.5 9.0 8.5 8.0 7.5 7.0 6.5 6.0 5.5 5.0 4.5 4.0 3.5 3.0 2.5 2.0 1.5 1.0 0.5 0.0 -0.5
ppm

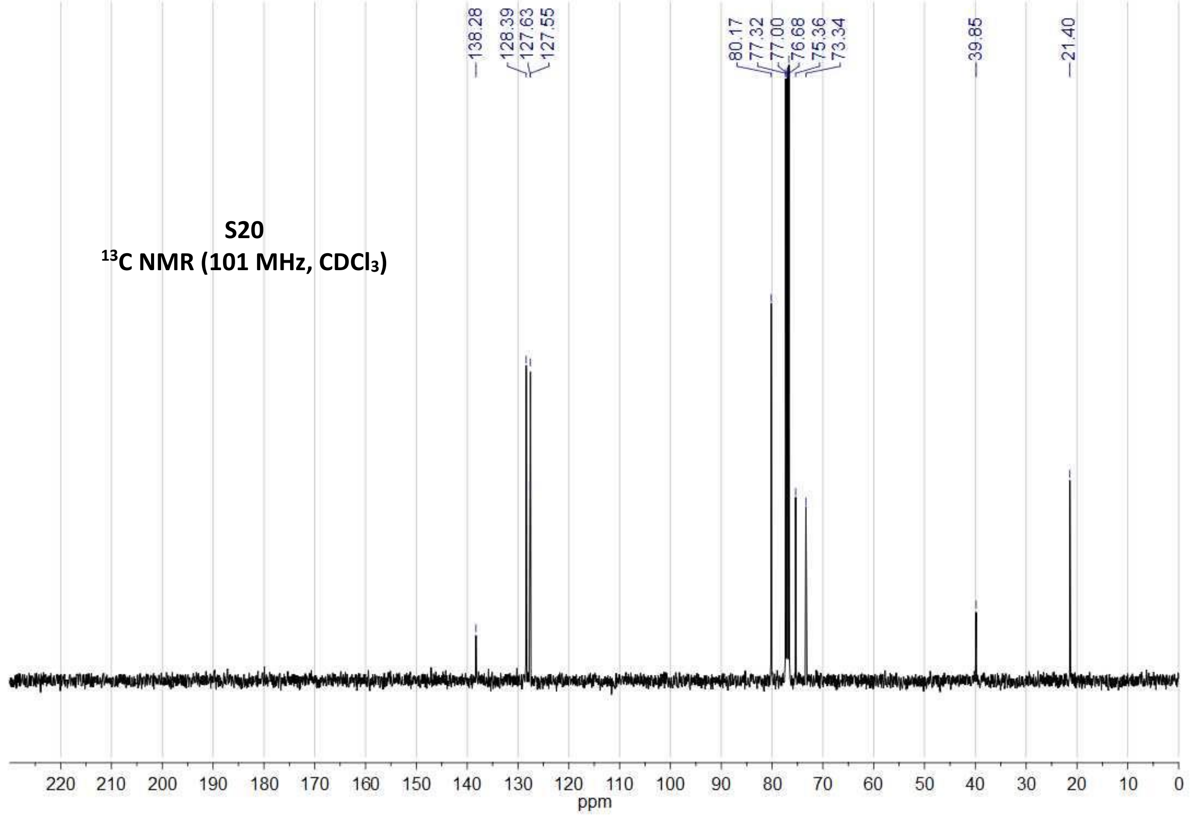
138.28
128.39 127.63 127.55
80.17 77.32 77.00 76.68 75.36 73.34
39.85
21.40
S20
13C NMR (101 MHz, CDCl3)
220 210 200 190 180 170 160 150 140 130 120 110 100 90 80 70 60 50 40 30 20 10 0
ppm

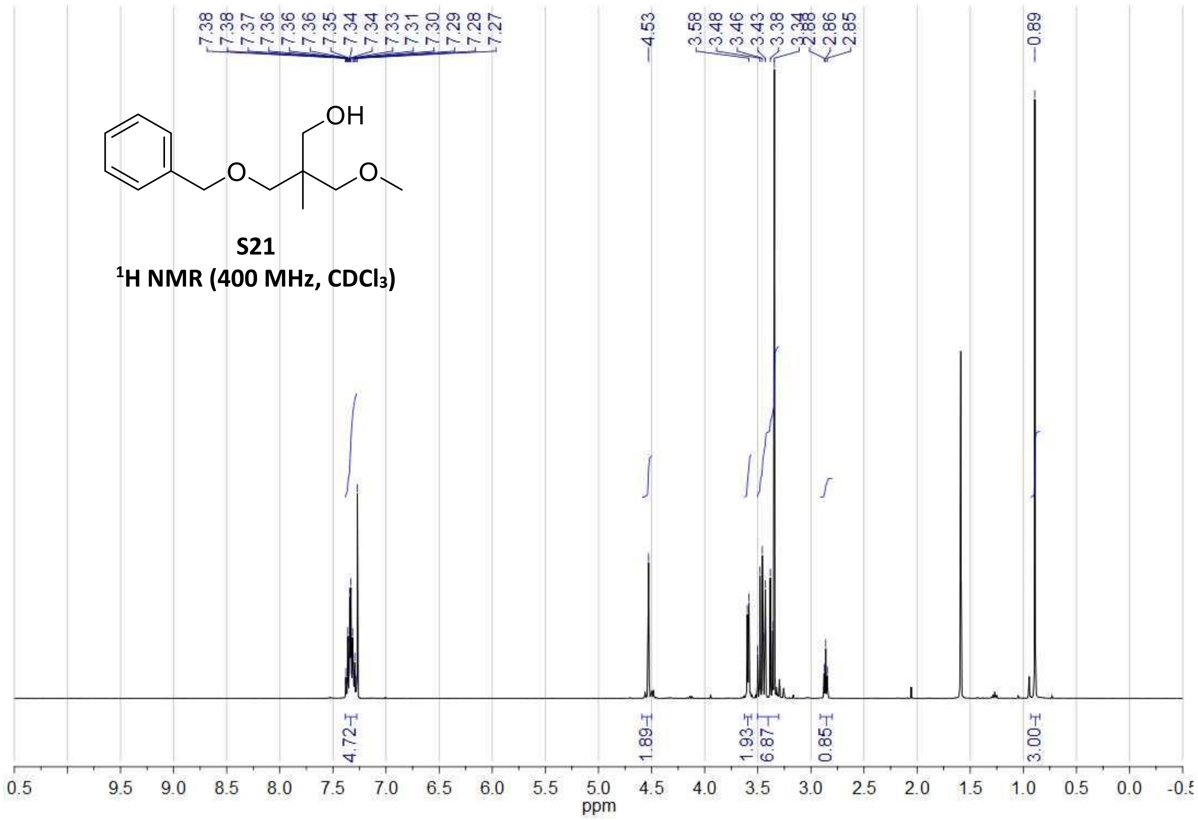

S21
^{1}H NMR (400 MHz, $CDCl_3$)

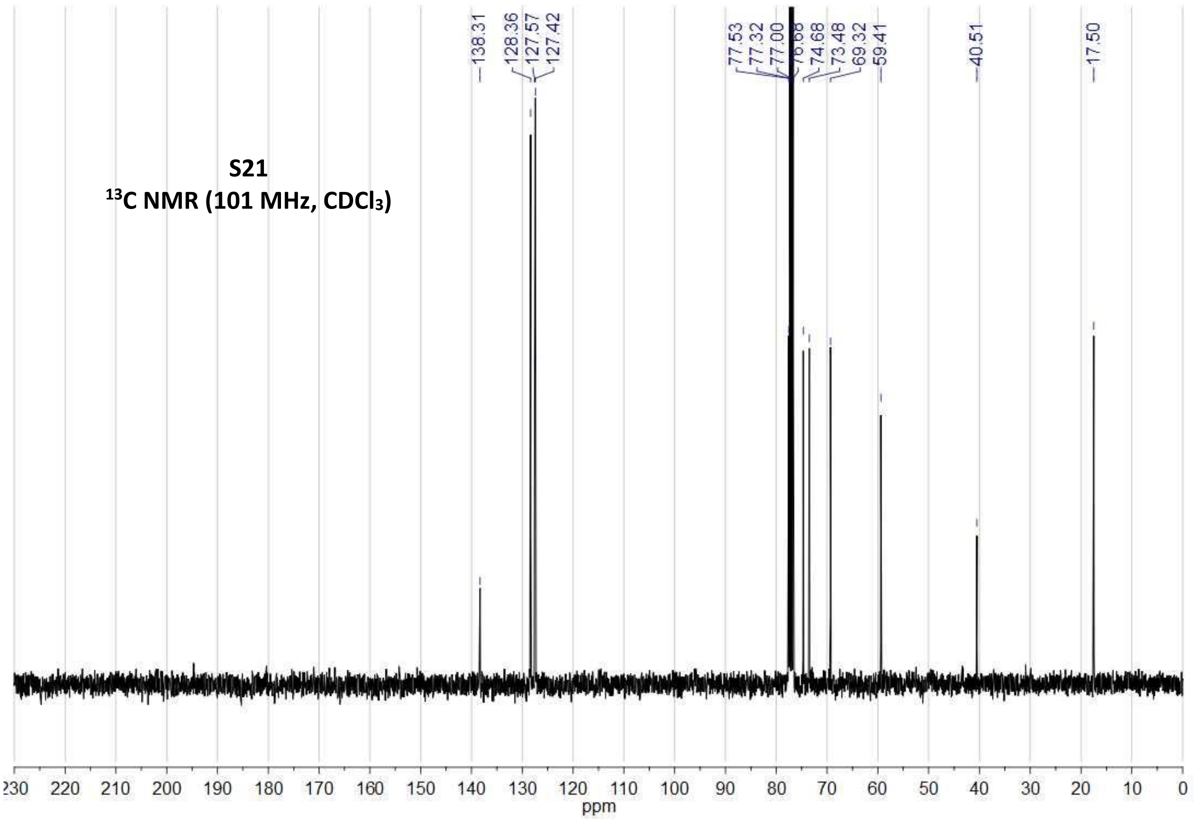

S21
^{13}C NMR (101 MHz, $CDCl_3$)

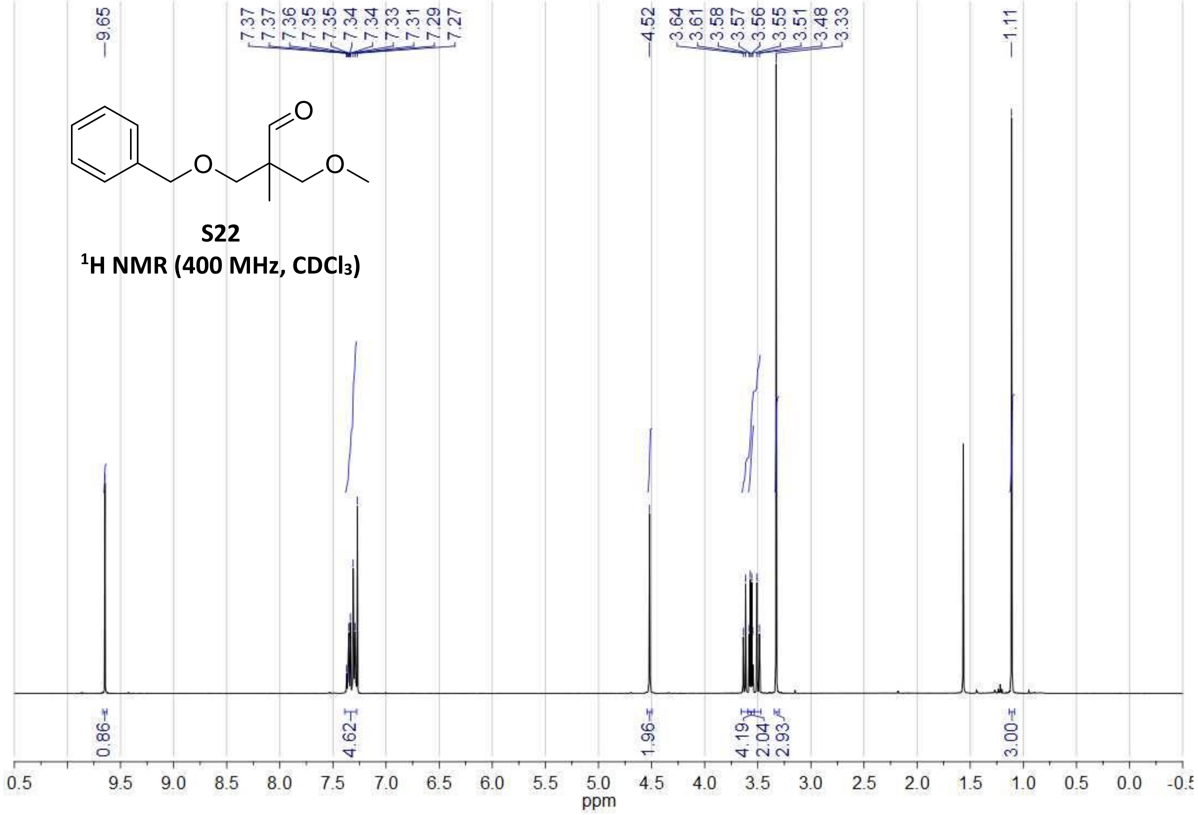
S22
^{1}H NMR (400 MHz, CDCl$_3$)
ppm

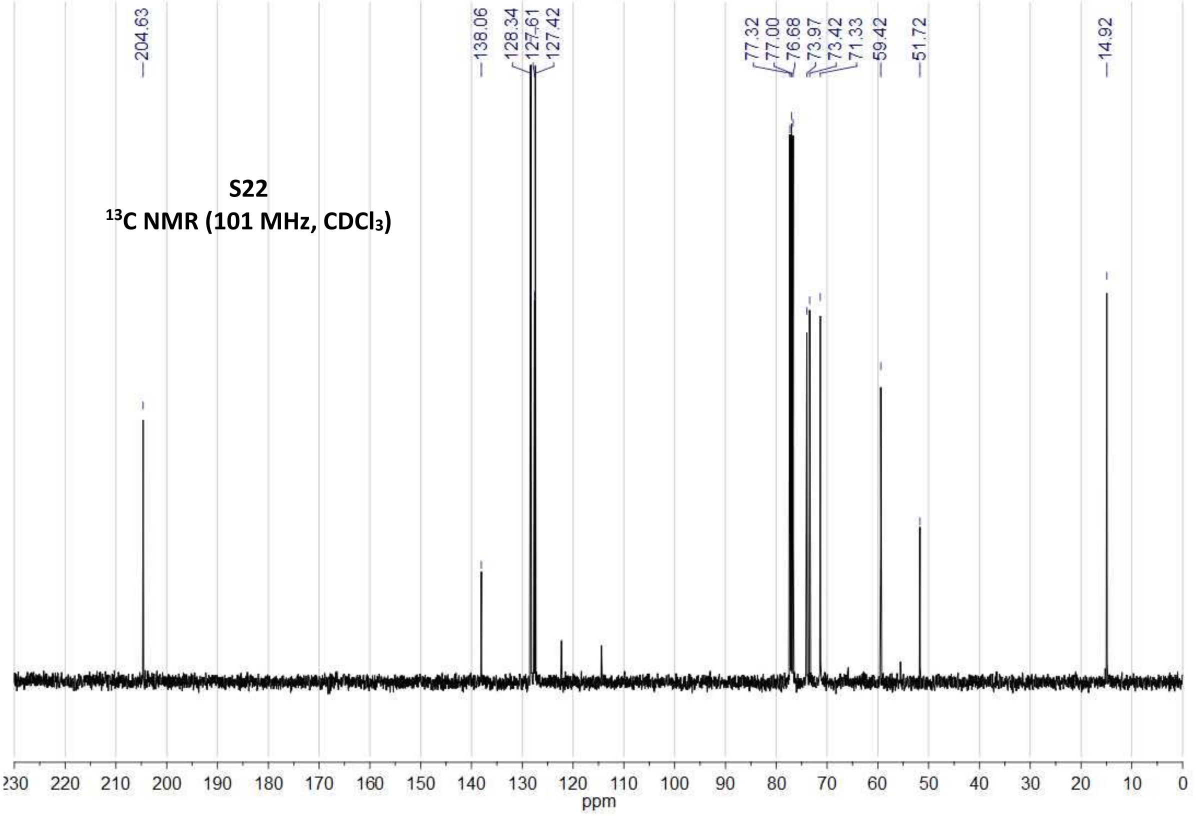
S22
^{13}C NMR (101 MHz, CDCl$_3$)
ppm

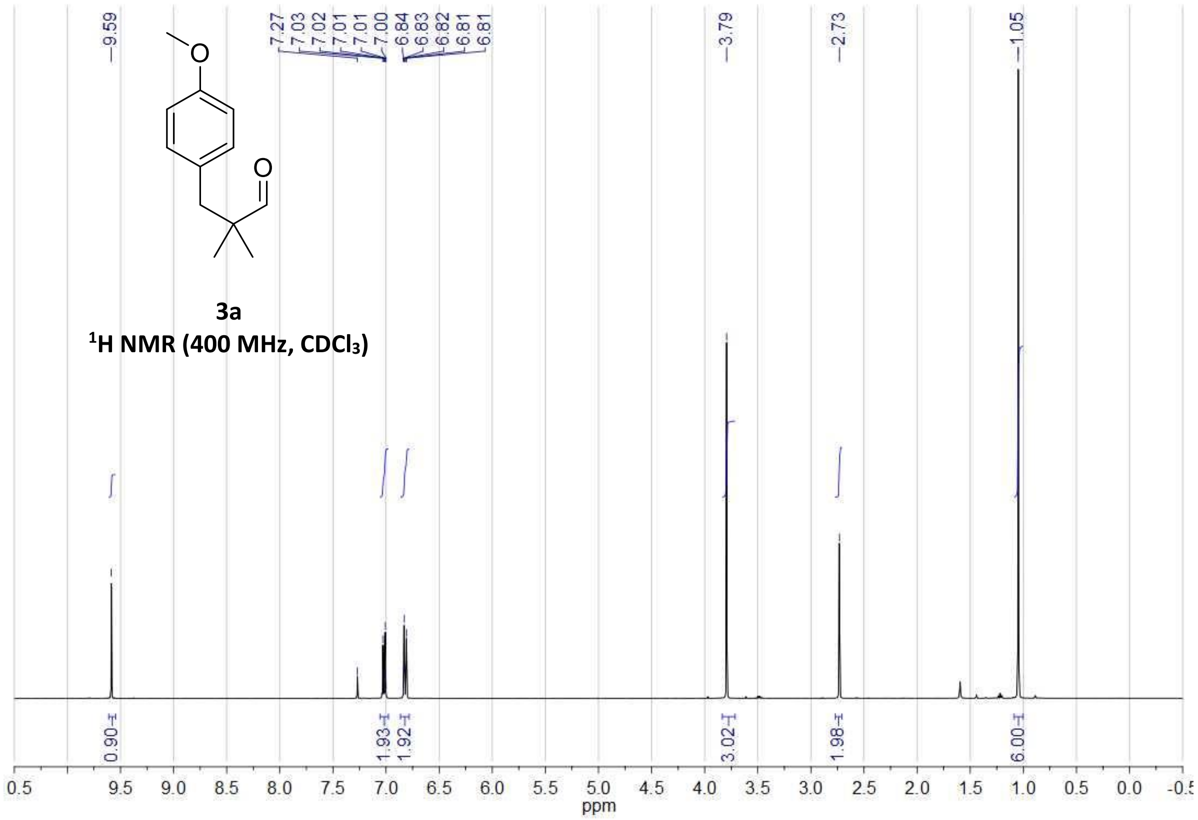
3a
^{1}H NMR (400 MHz, $CDCl_3$)
9.59
7.27 7.03 7.02 7.01 7.01 7.00 6.84 6.83 6.82 6.81 6.81
3.79
2.73
1.05
0.90 1.93 1.92 3.02 1.98 6.00
ppm

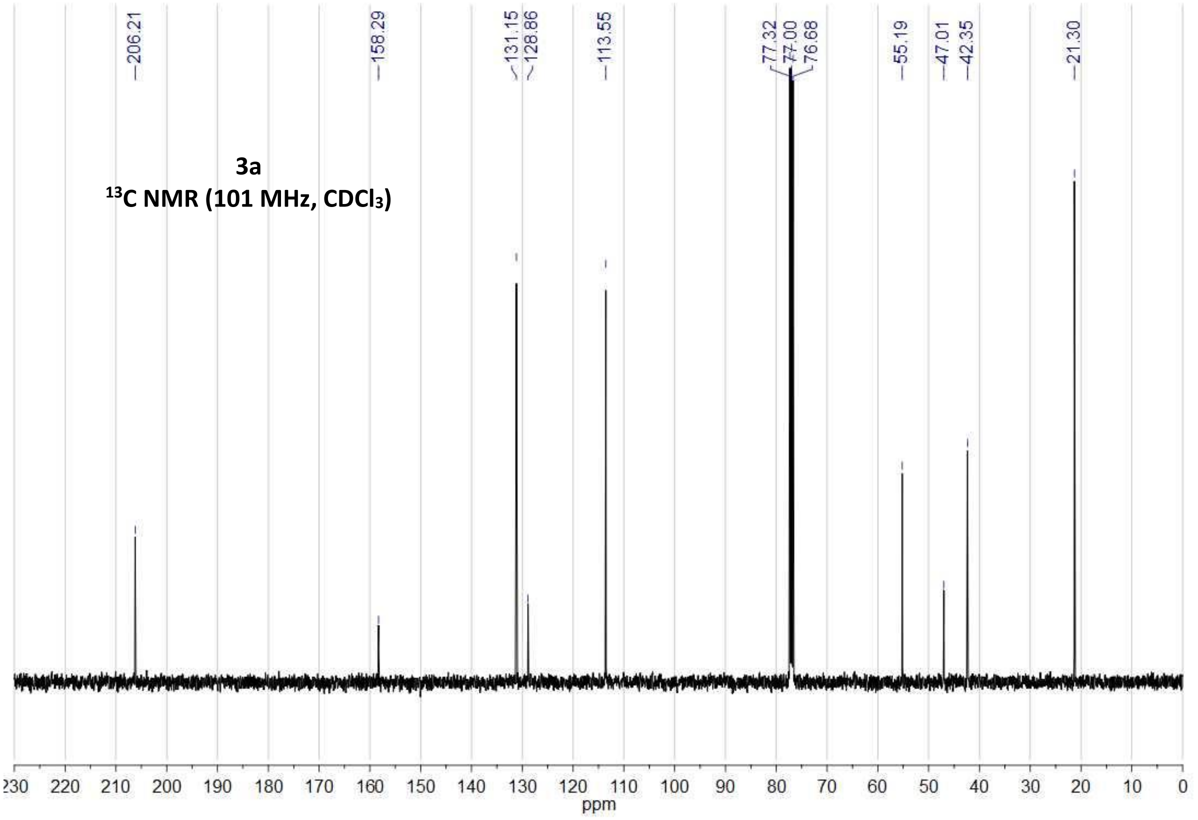
3a
^{13}C NMR (101 MHz, $CDCl_3$)
206.21
158.29
131.15 128.86
113.55
77.32 77.00 76.68
55.19
47.01 42.35
21.30
ppm

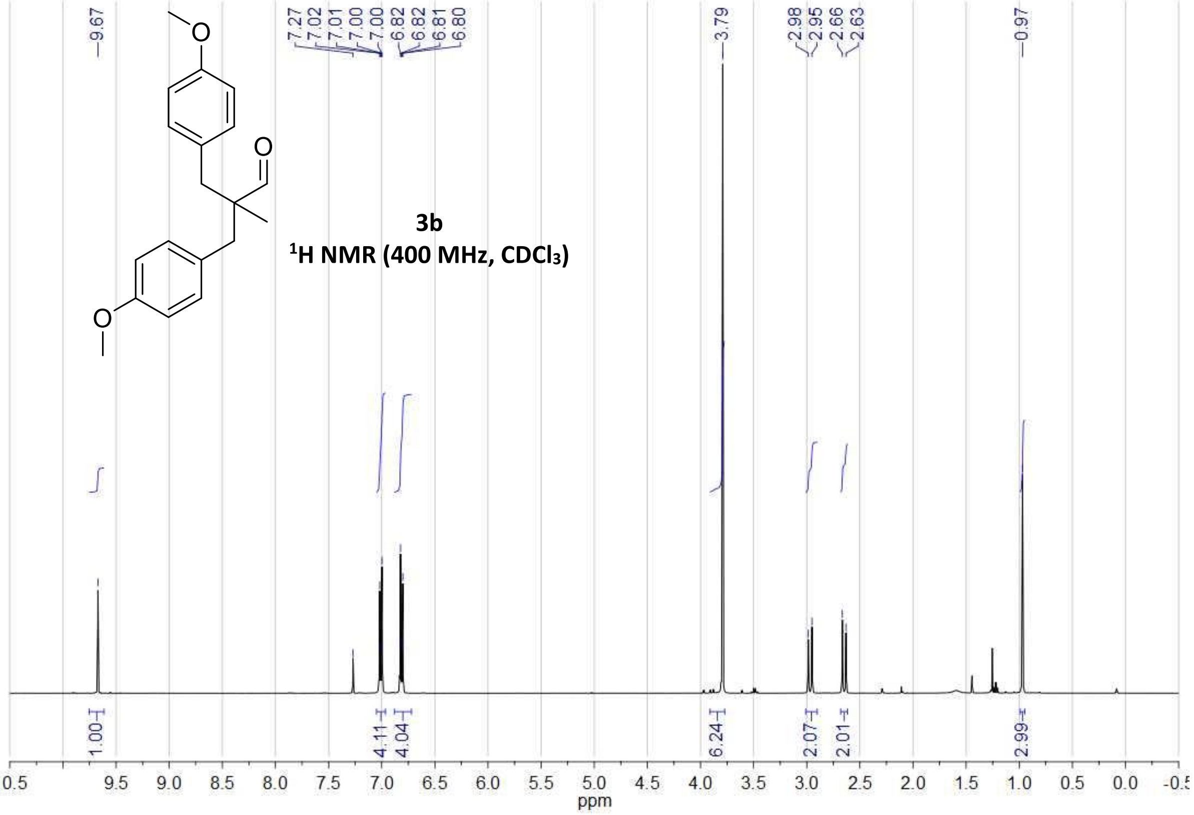
3b
1H NMR (400 MHz, CDCl3)
9.67
7.27 7.02 7.01 7.00 7.00 6.82 6.82 6.81 6.80
3.79
2.98 2.95 2.66 2.63
0.97
1.00
4.11 4.04
6.24
2.07 2.01
2.99
ppm

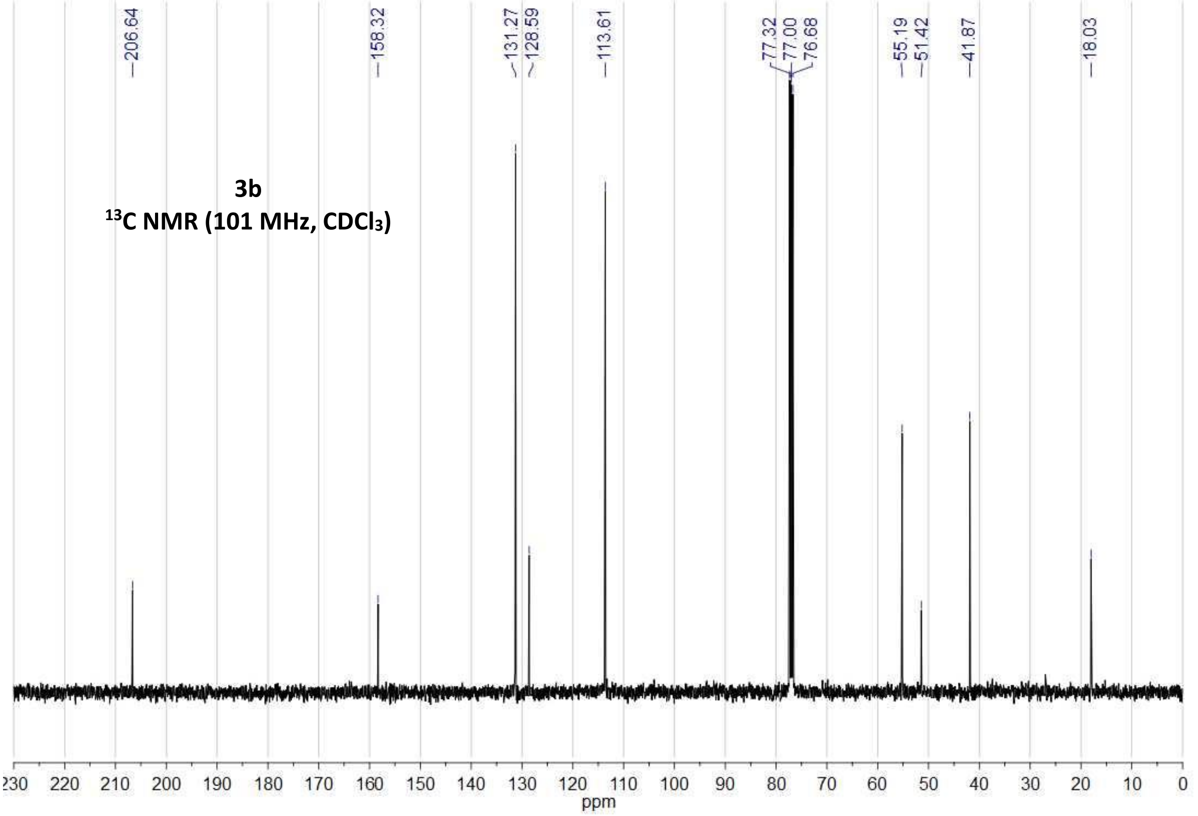
3b
13C NMR (101 MHz, CDCl3)
206.64
158.32
131.27 128.59
113.61
77.32 77.00 76.68
55.19 51.42
41.87
18.03
ppm

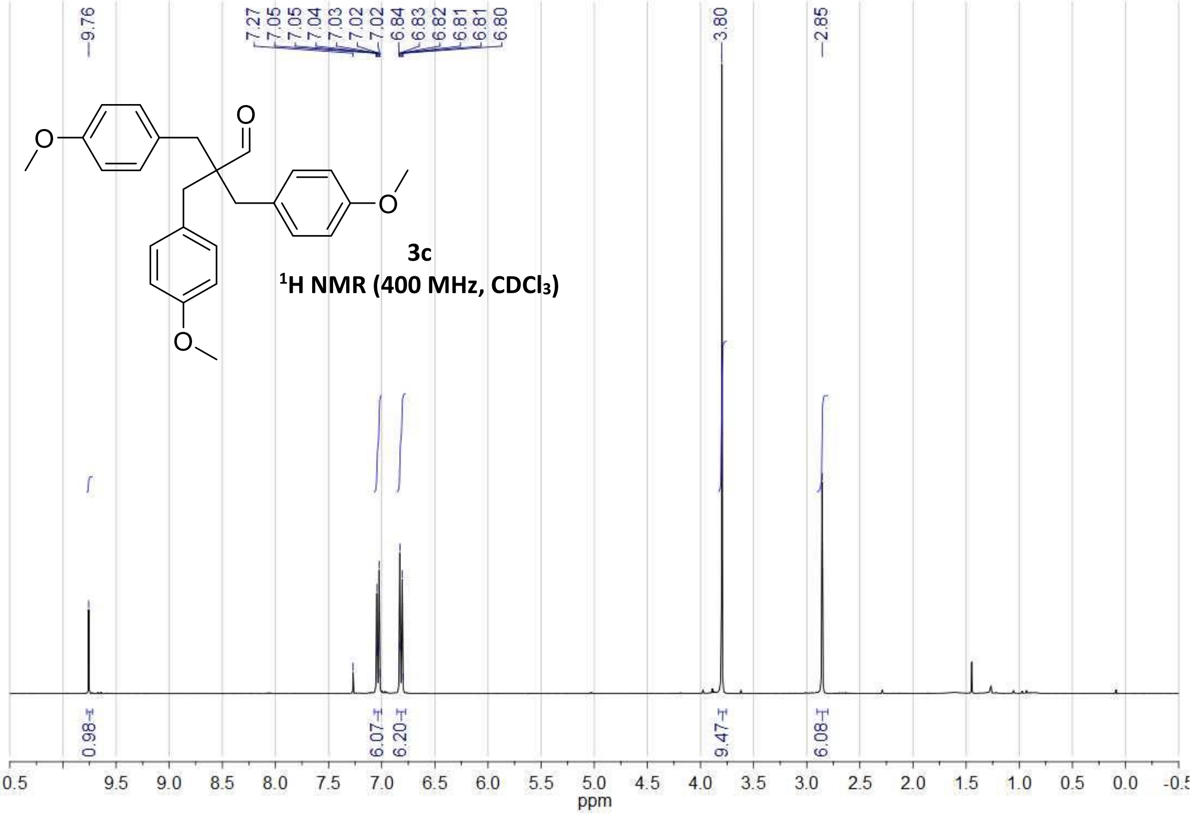

3c
^{1}H NMR (400 MHz, $CDCl_3$)

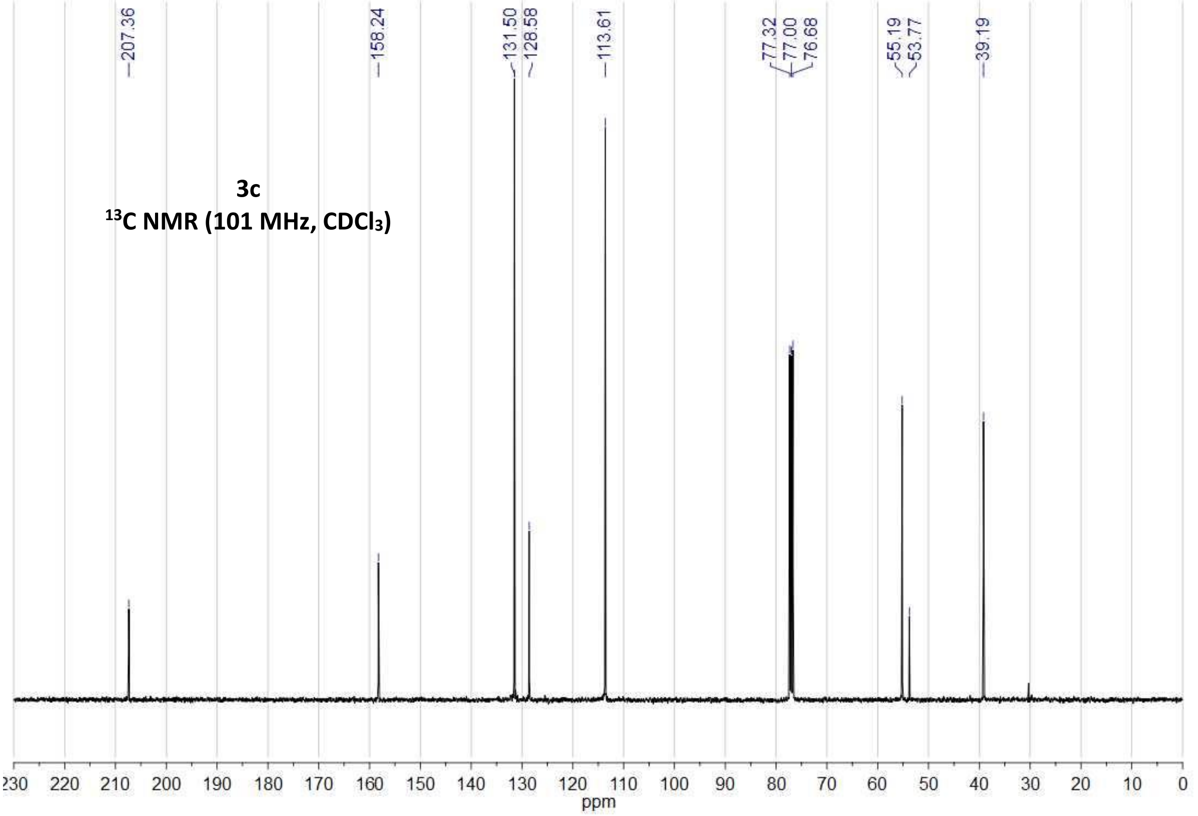

3c
^{13}C NMR (101 MHz, $CDCl_3$)

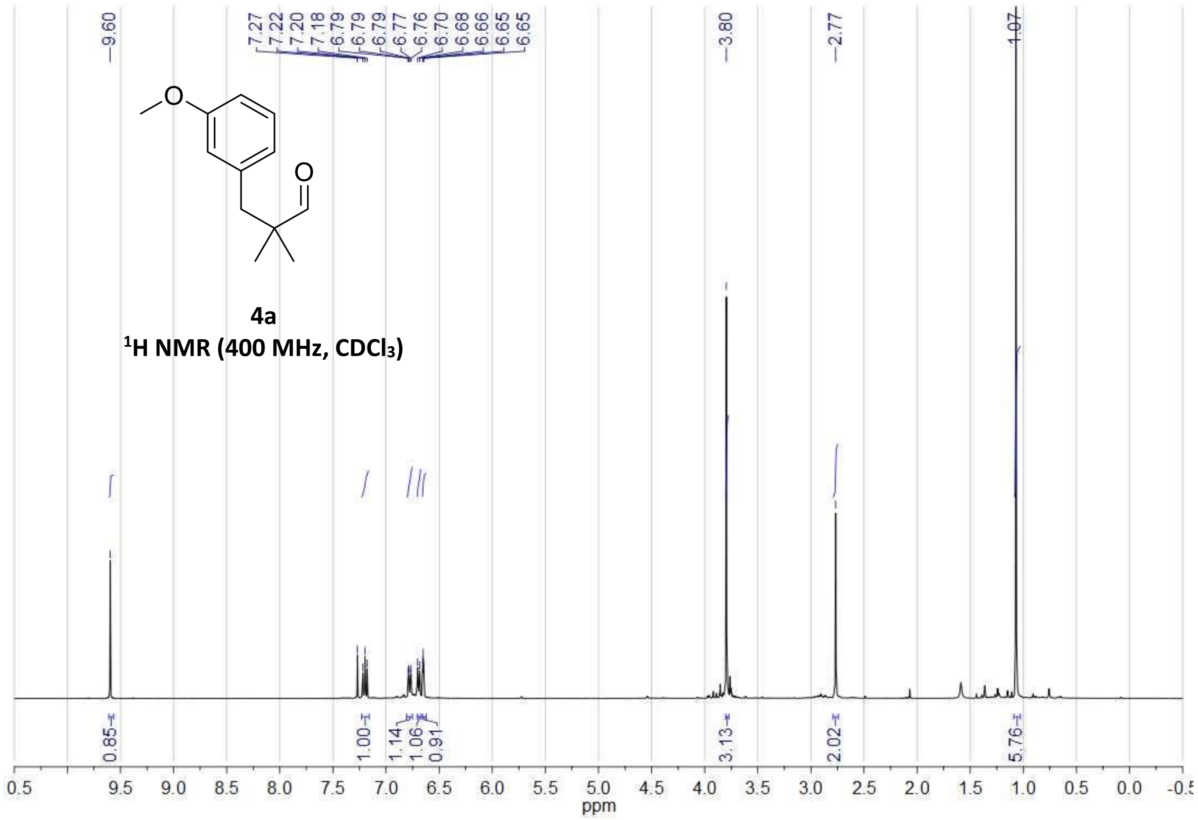
4a
^{1}H NMR (400 MHz, CDCl$_3$)
ppm

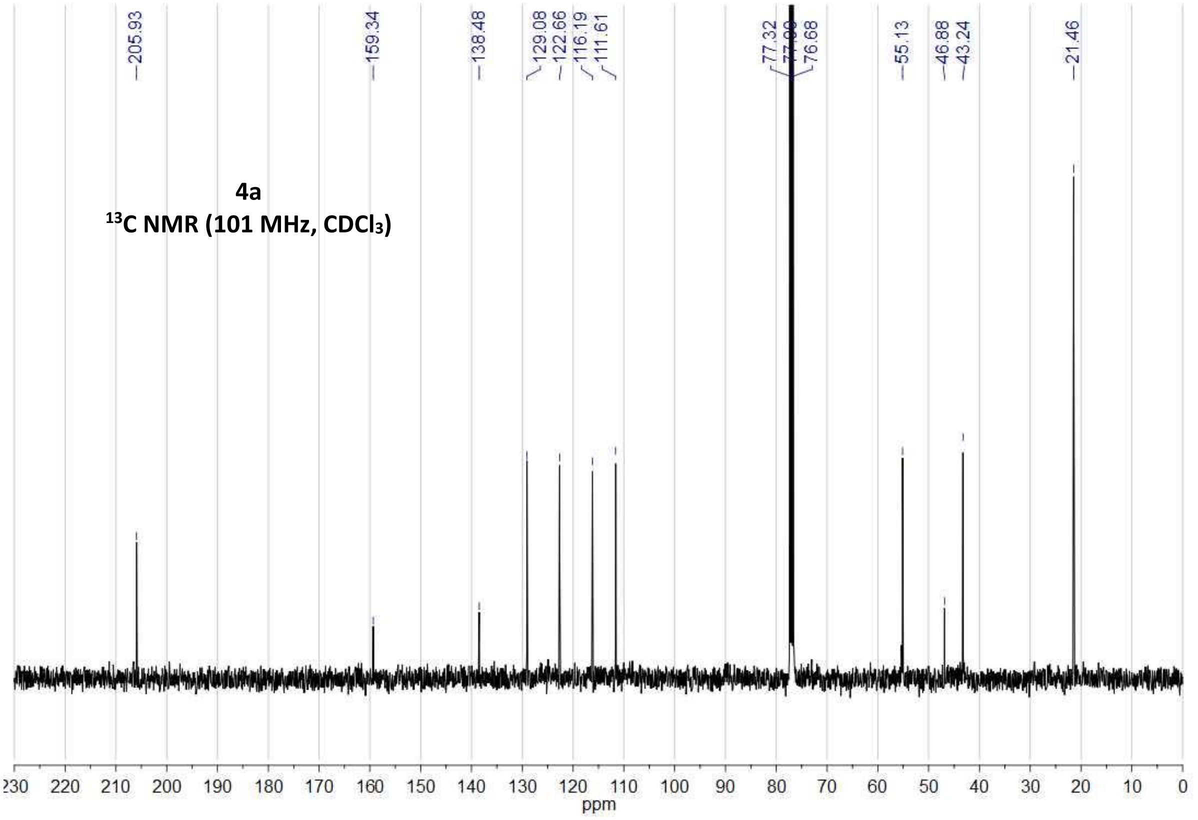
4a
^{13}C NMR (101 MHz, CDCl$_3$)
ppm

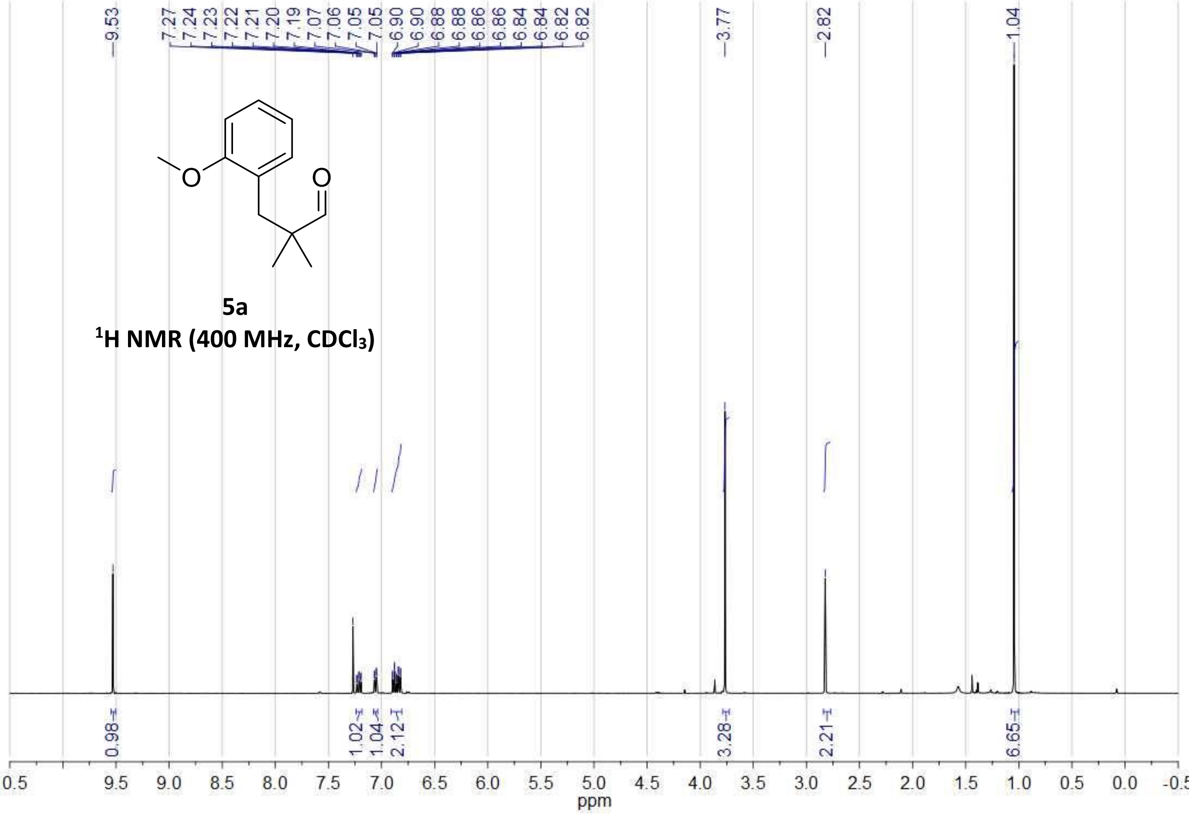

5a

^{1}H NMR (400 MHz, $CDCl_3$)

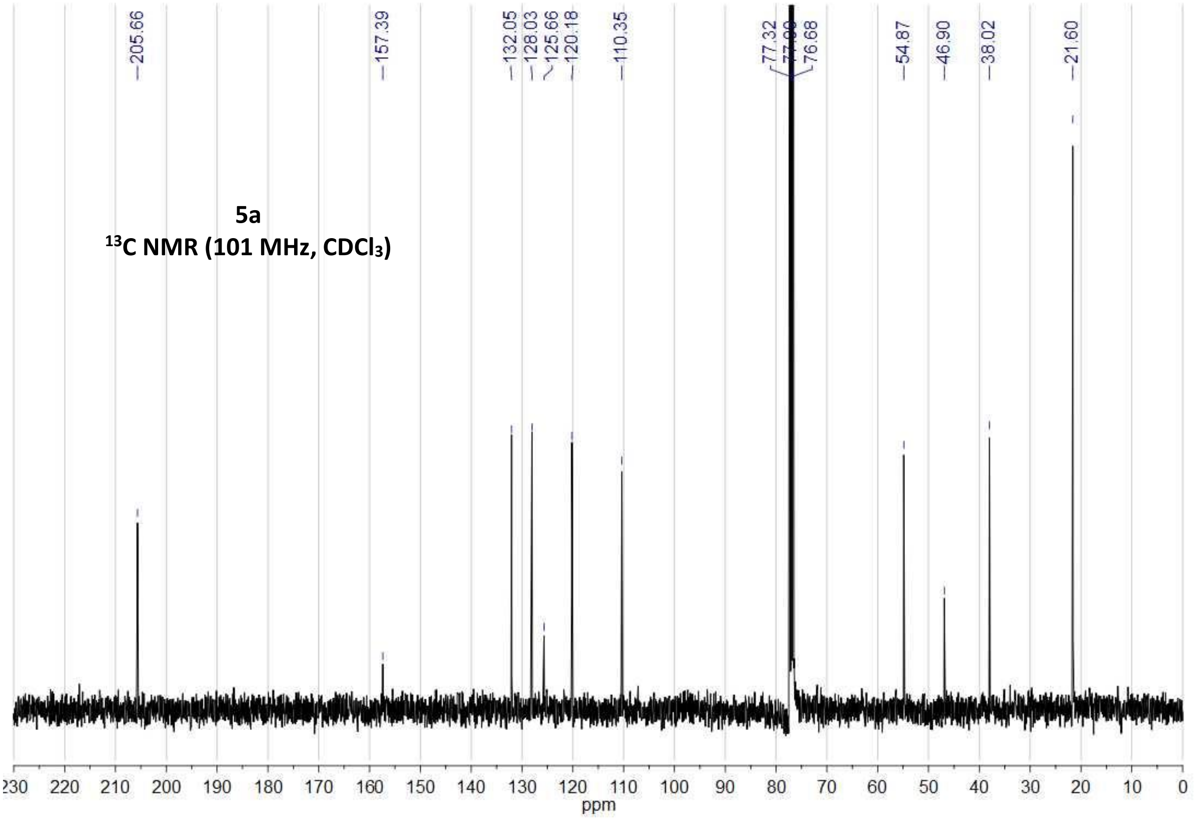

5a

^{13}C NMR (101 MHz, $CDCl_3$)

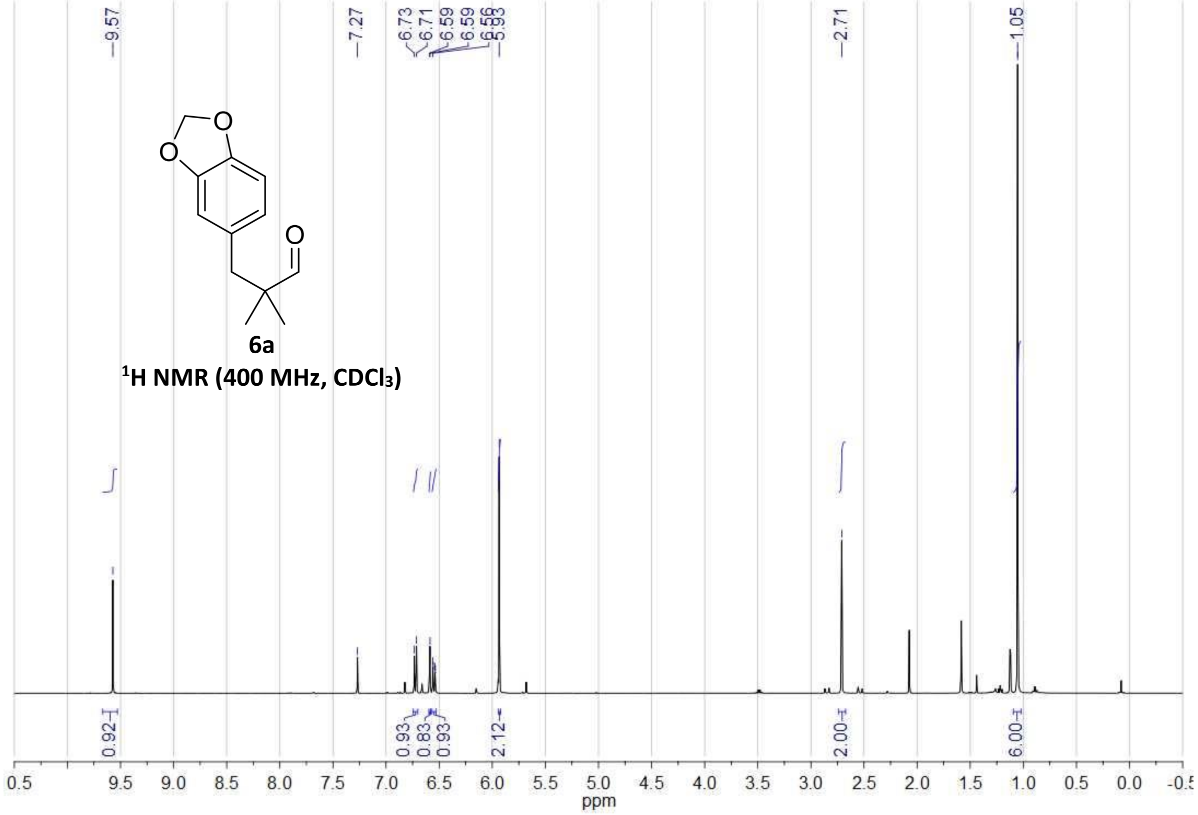
9.57
7.27
6.73
6.71
6.59
6.59
6.56
5.93
2.71
1.05
6a
1H NMR (400 MHz, CDCl3)
0.92
0.93
0.83
0.93
2.12
2.00
6.00
ppm

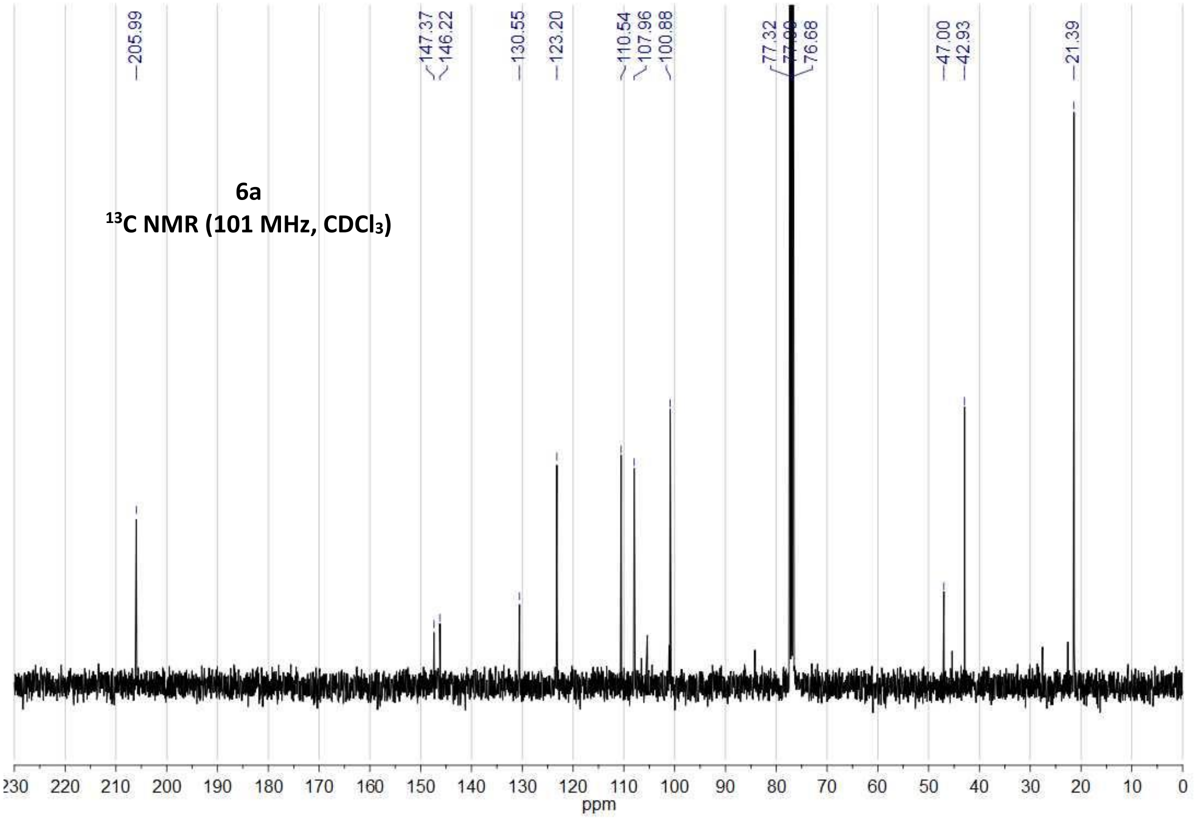
205.99
147.37
146.22
130.55
123.20
110.54
107.96
100.88
77.32
76.68
47.00
42.93
21.39
6a
13C NMR (101 MHz, CDCl3)
ppm

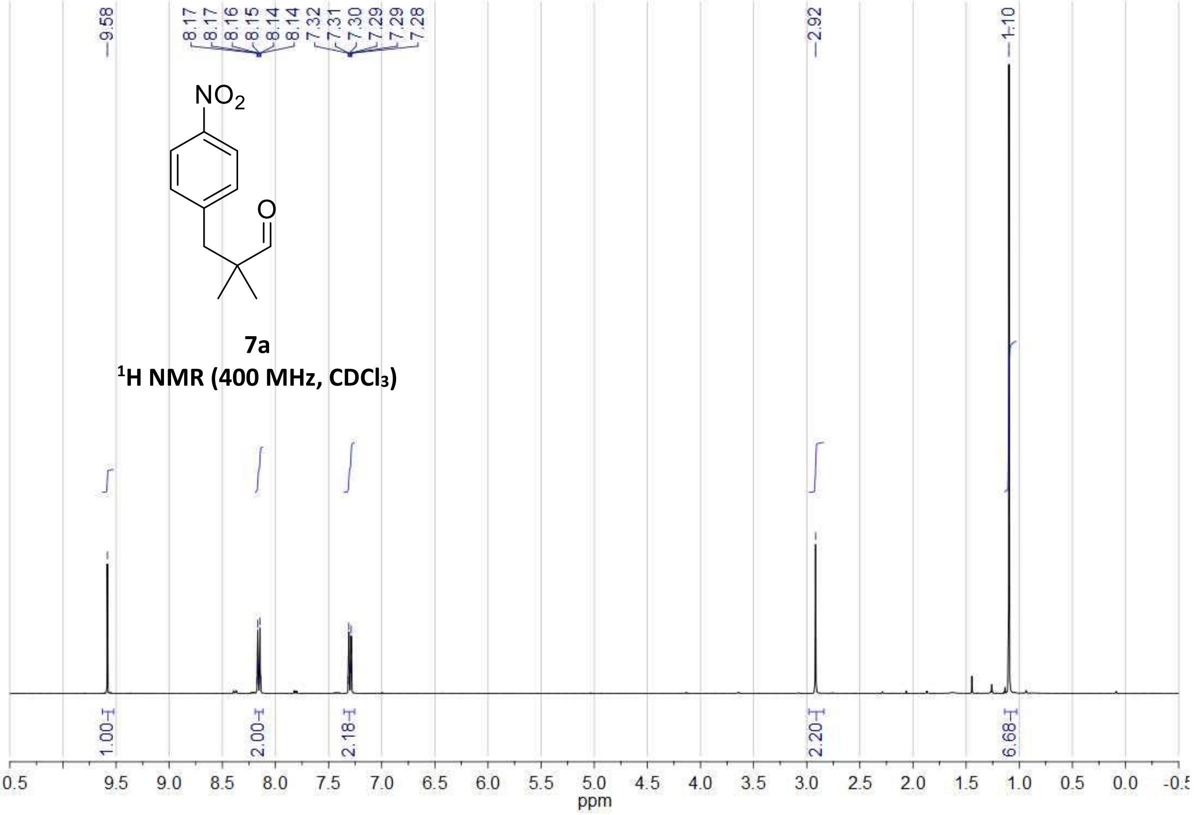
9.58
8.17
8.17
8.16
8.15
8.14
8.14
7.32
7.31
7.30
7.29
7.29
7.28
2.92
1.10
NO_2
O
7a
^{1}H NMR (400 MHz, $CDCl_3$)
1.00
2.00
2.18
2.20
6.68
ppm

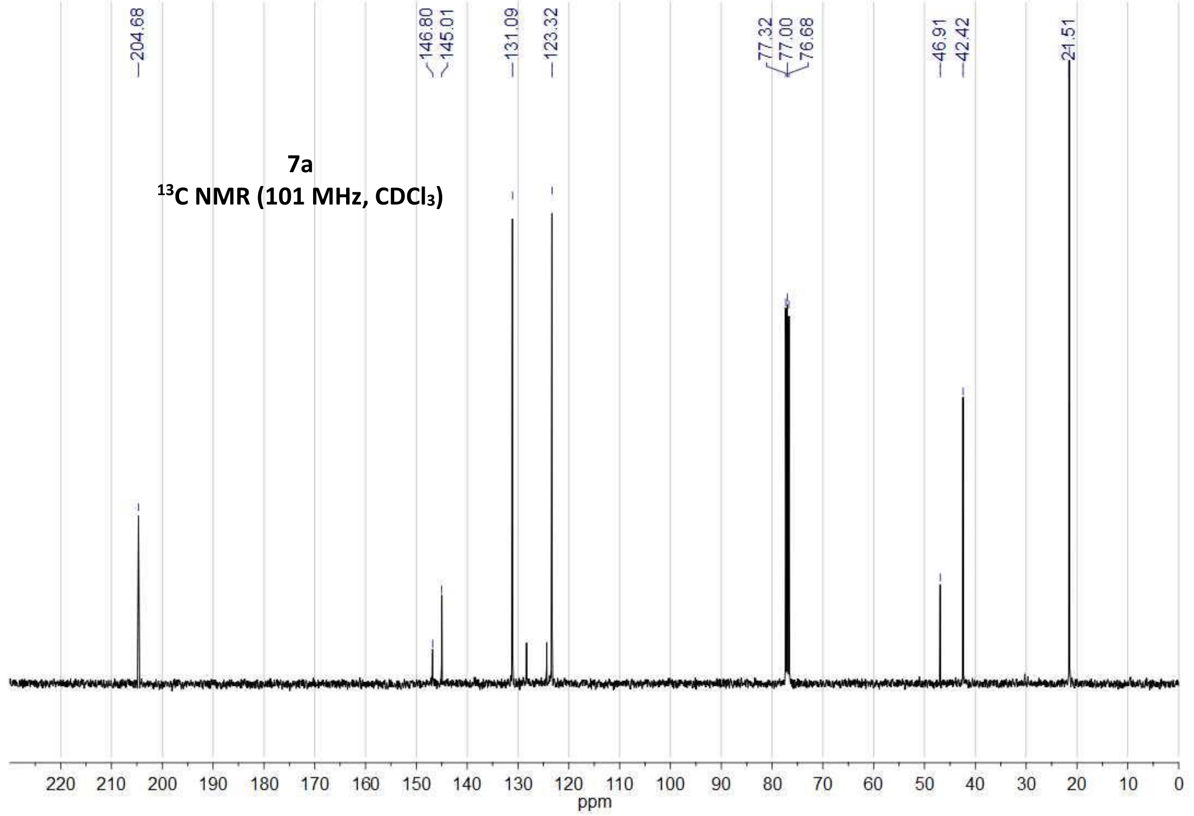
204.68
146.80
145.01
131.09
123.32
77.32
77.00
76.68
46.91
42.42
21.51
7a
^{13}C NMR (101 MHz, $CDCl_3$)
ppm

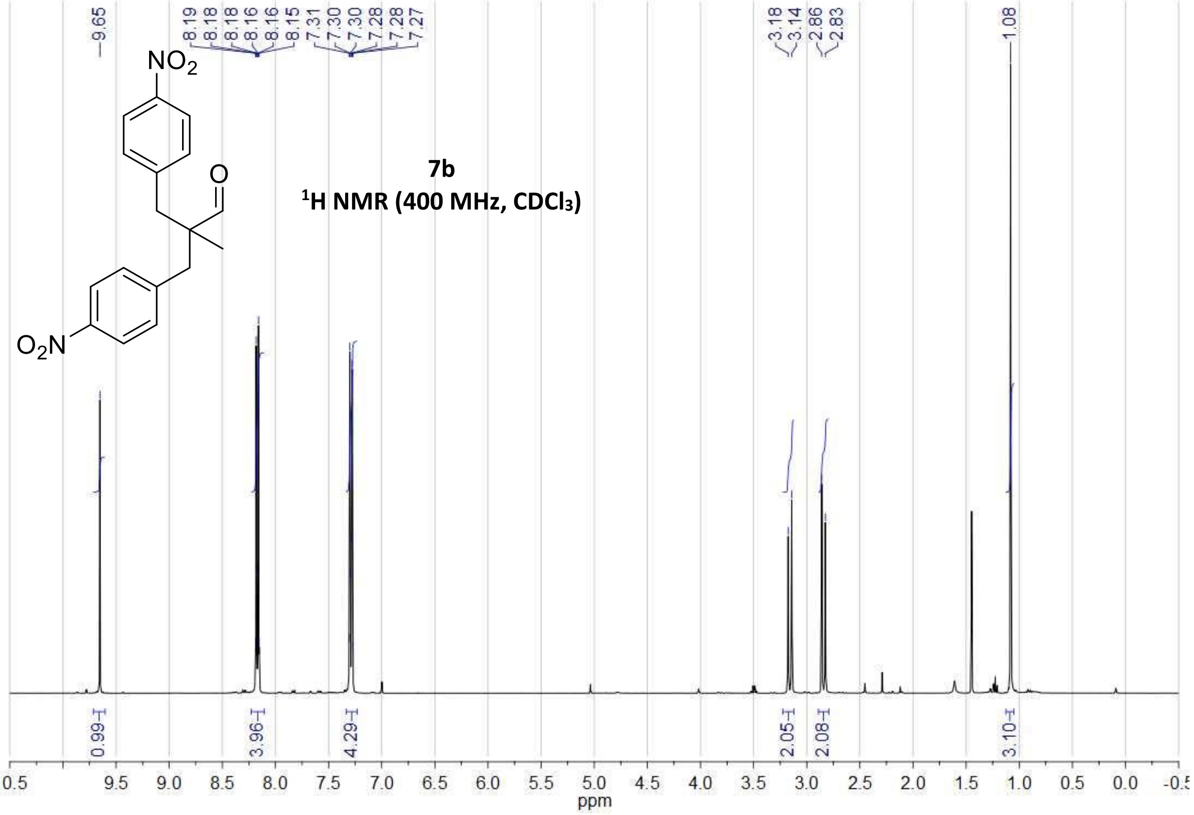
7b
1H NMR (400 MHz, CDCl3)
ppm

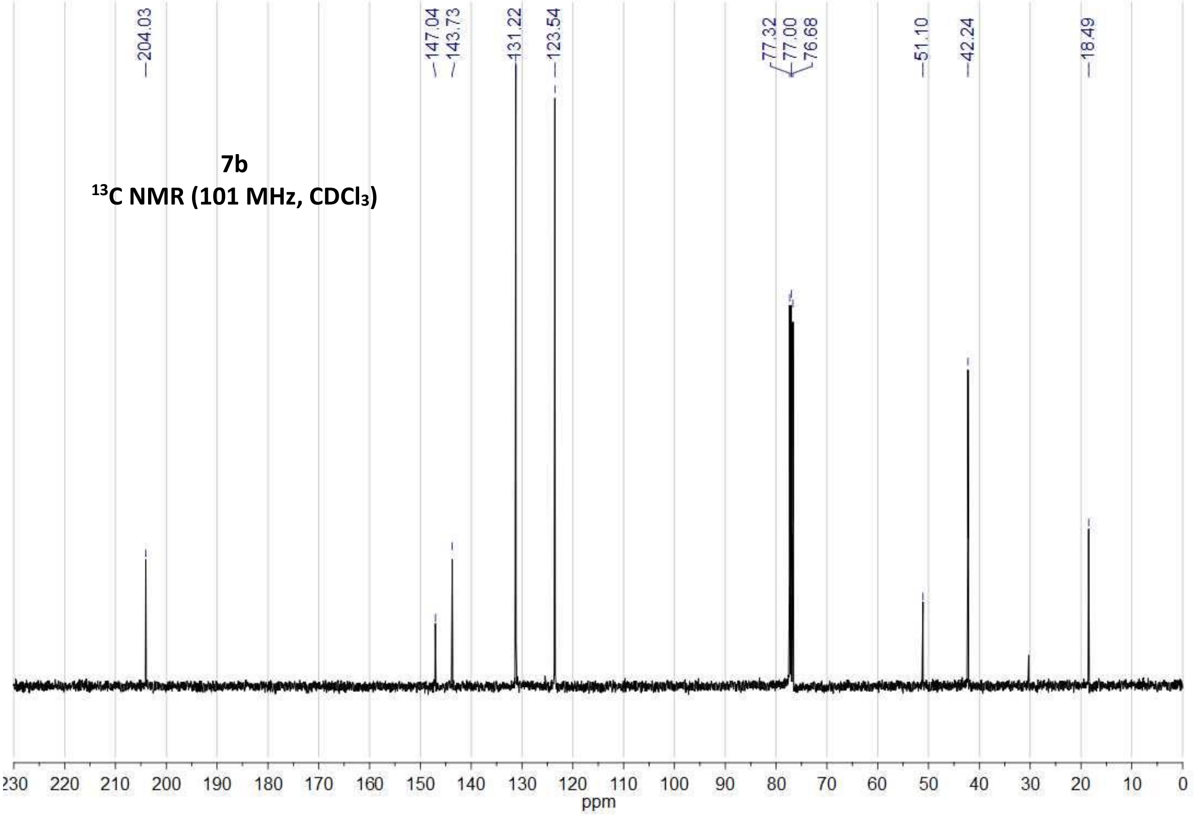
7b
13C NMR (101 MHz, CDCl3)
ppm

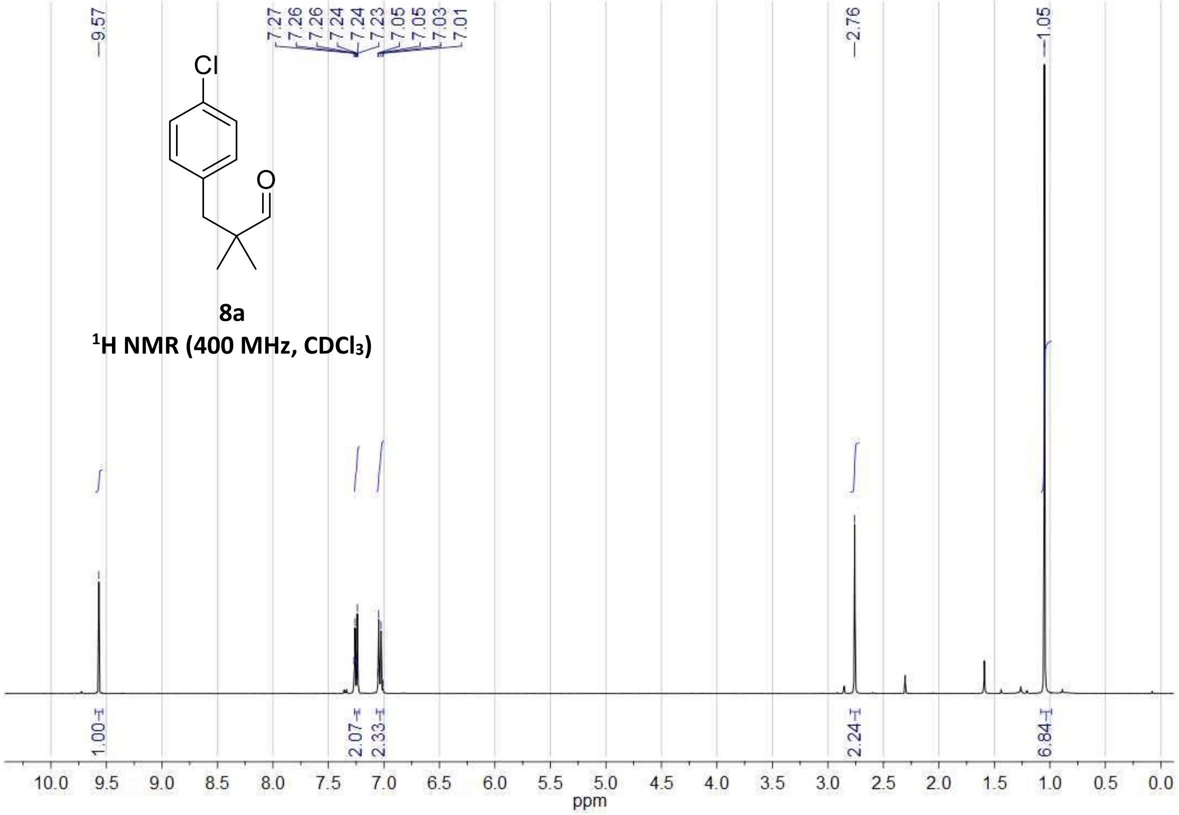

8a
^{1}H NMR (400 MHz, $CDCl_3$)

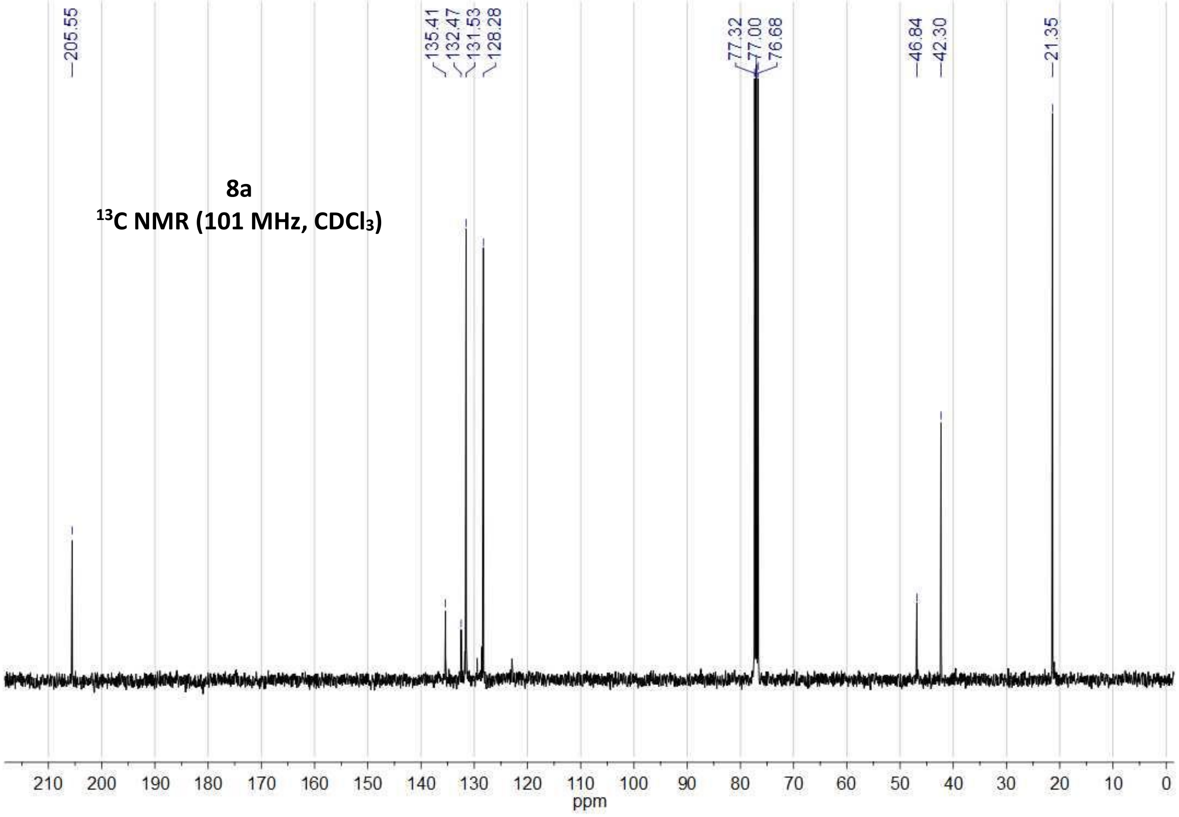

8a
^{13}C NMR (101 MHz, $CDCl_3$)

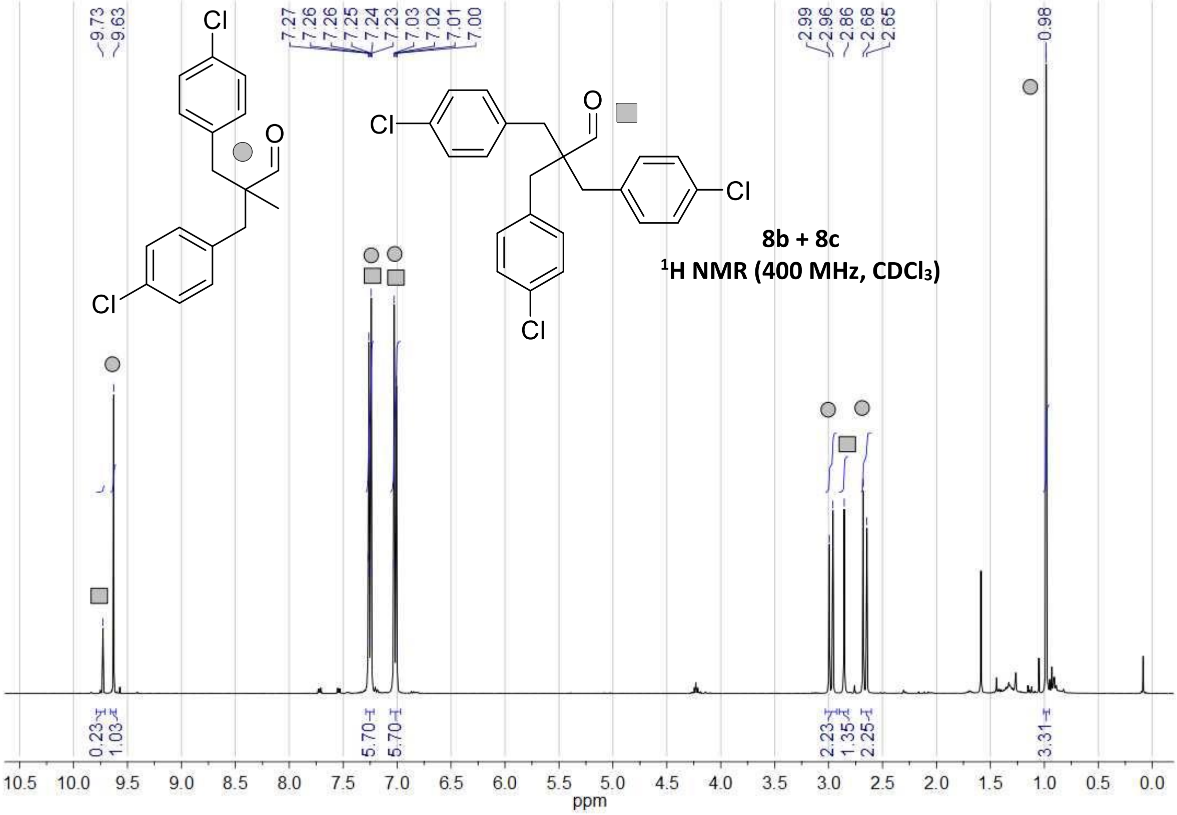
8b + 8c
¹H NMR (400 MHz, CDCl₃)
ppm

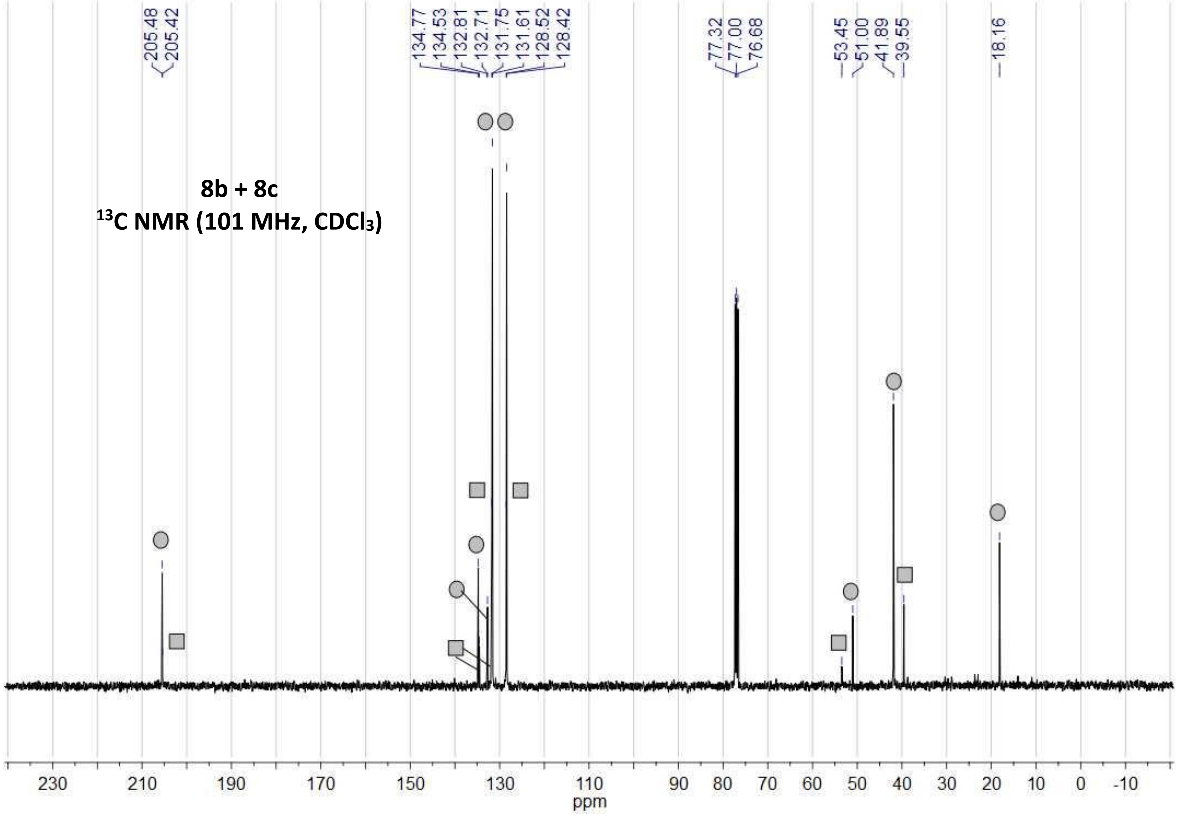
8b + 8c
¹³C NMR (101 MHz, CDCl₃)
ppm

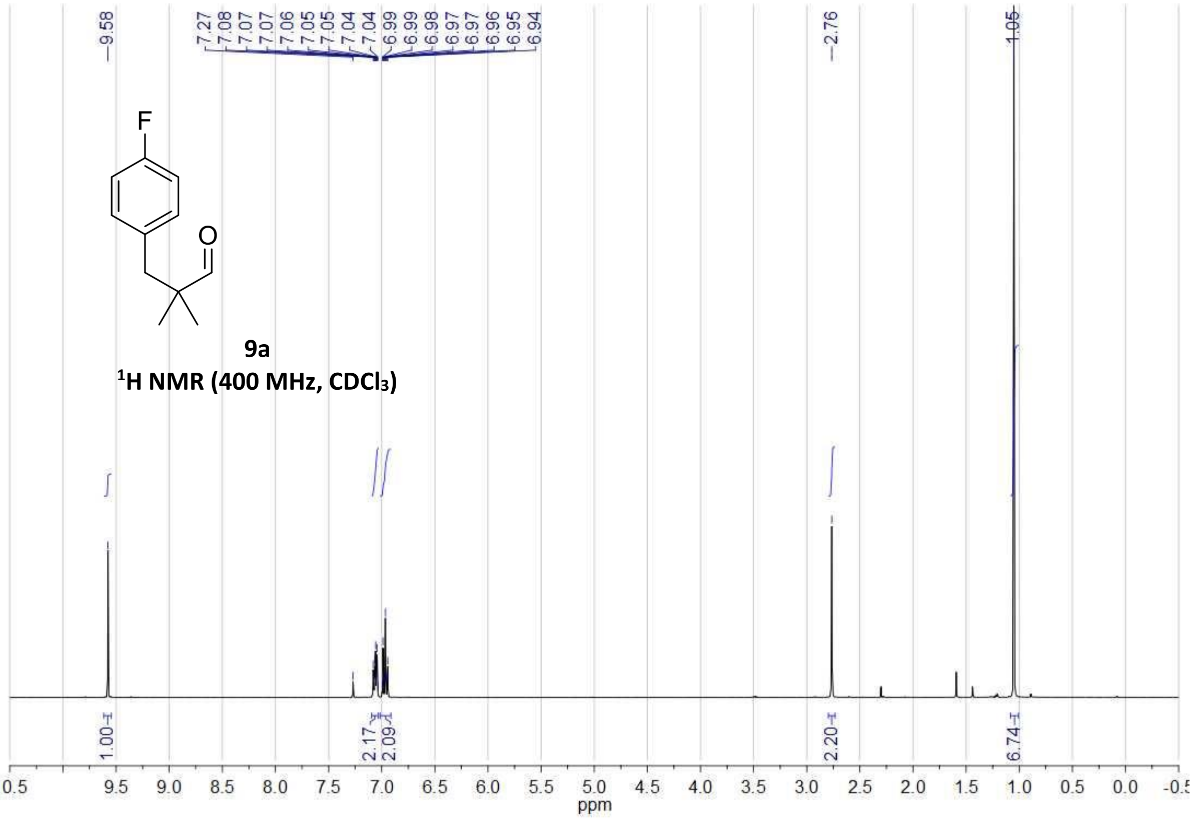
9a
^{1}H NMR (400 MHz, CDCl$_3$)
9.58
7.27 7.08 7.07 7.07 7.06 7.05 7.05 7.04 7.04 6.99 6.99 6.98 6.97 6.97 6.96 6.95 6.94
2.76
1.05
1.00
2.17
2.09
2.20
6.74
ppm

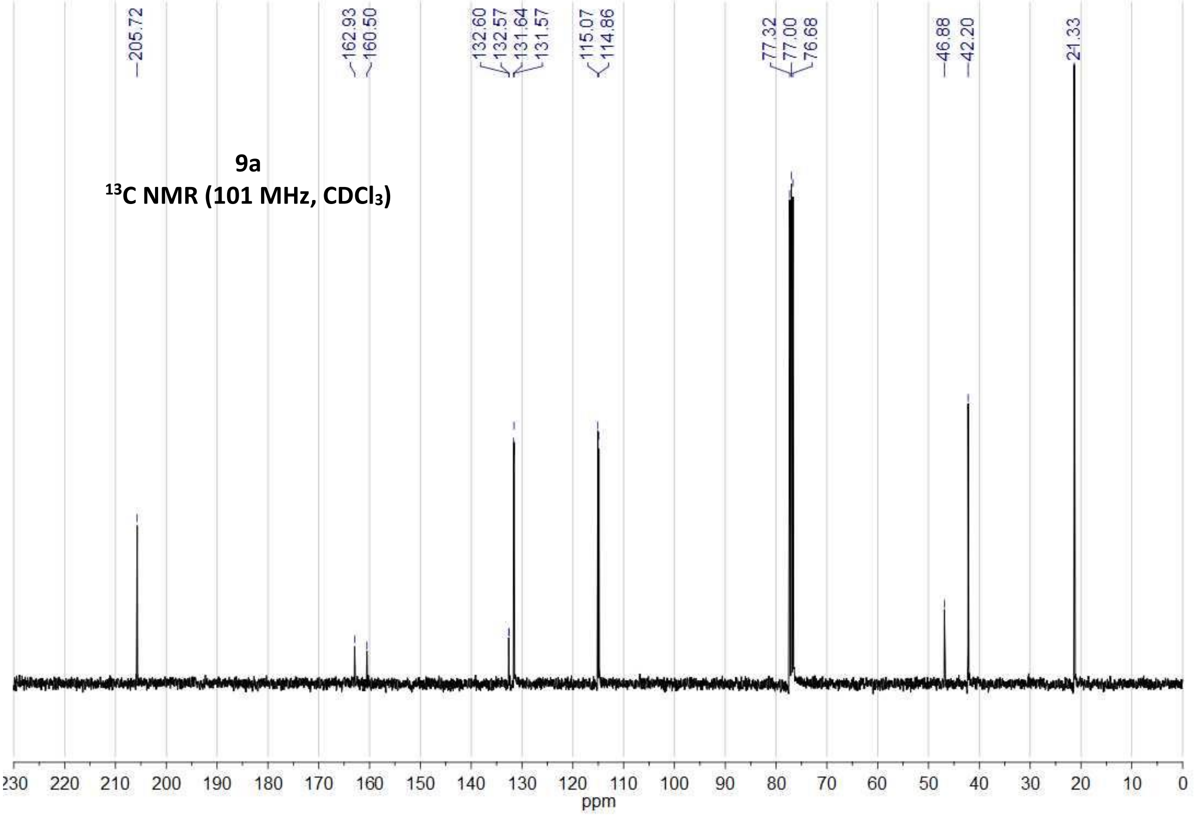
9a
^{13}C NMR (101 MHz, CDCl$_3$)
205.72
162.93 160.50
132.60 132.57 131.64 131.57
115.07 114.86
77.32 77.00 76.68
46.88 42.20
21.33
ppm

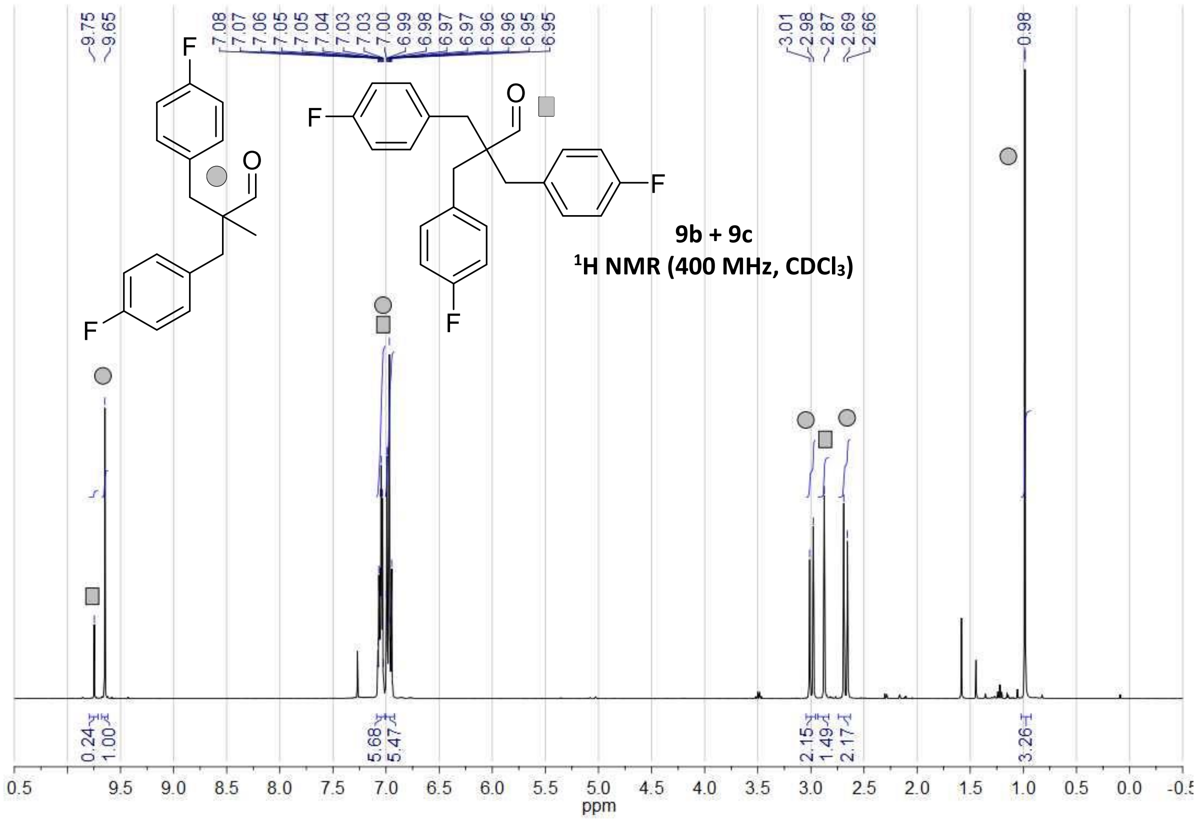
9b + 9c
^{1}H NMR (400 MHz, CDCl$_3$)
ppm

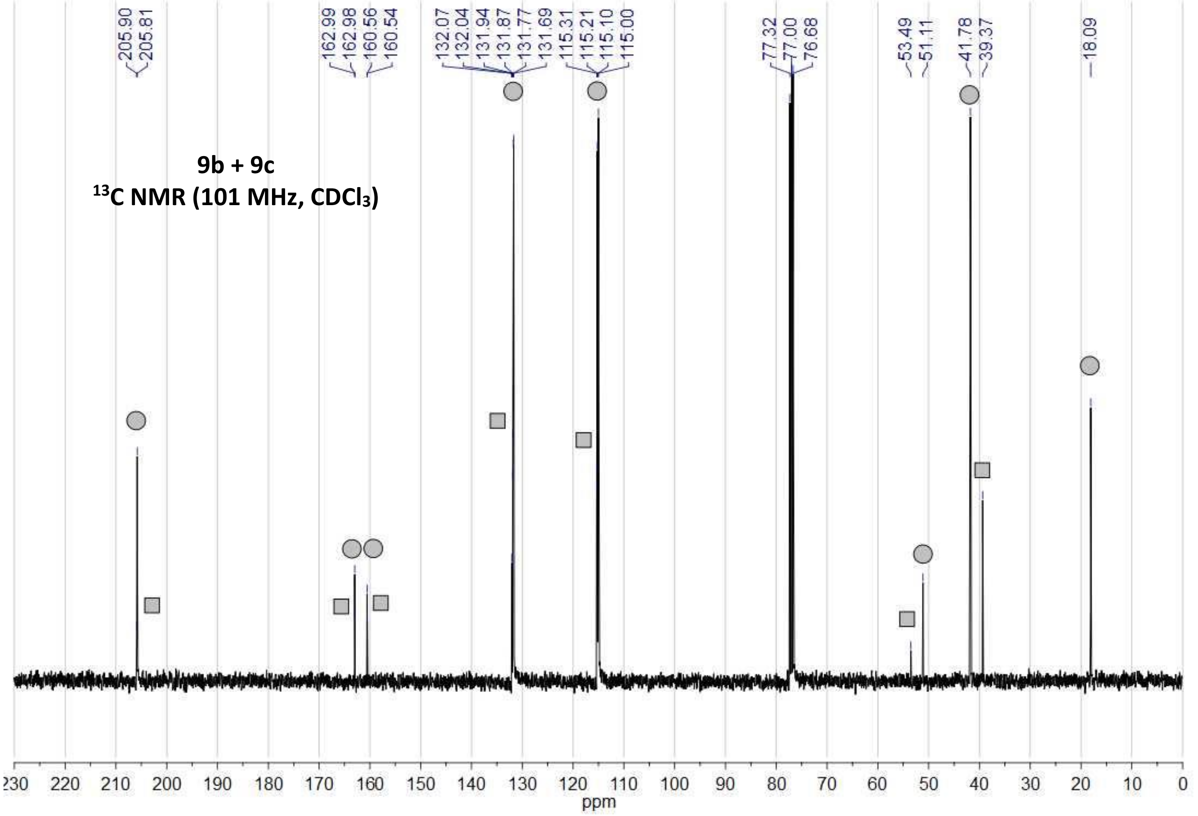
9b + 9c
^{13}C NMR (101 MHz, CDCl$_3$)
ppm

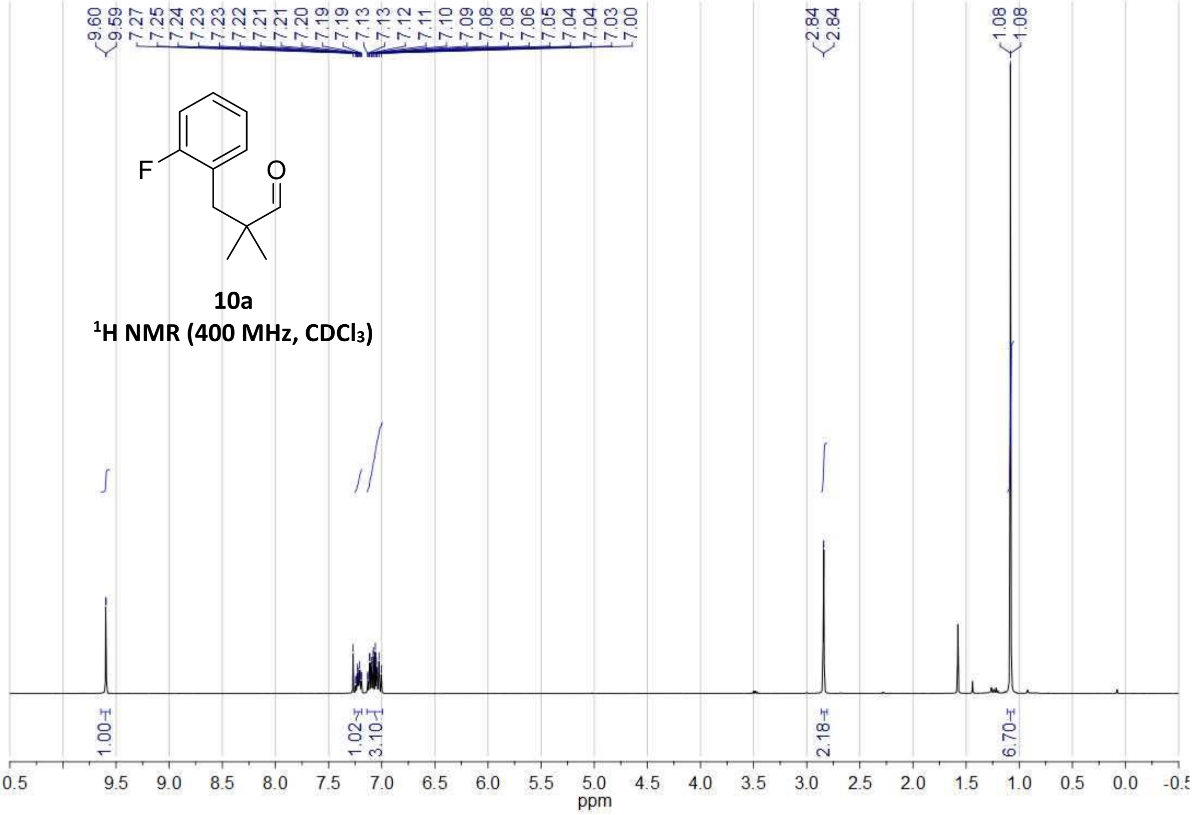

10a
^{1}H NMR (400 MHz, $CDCl_3$)

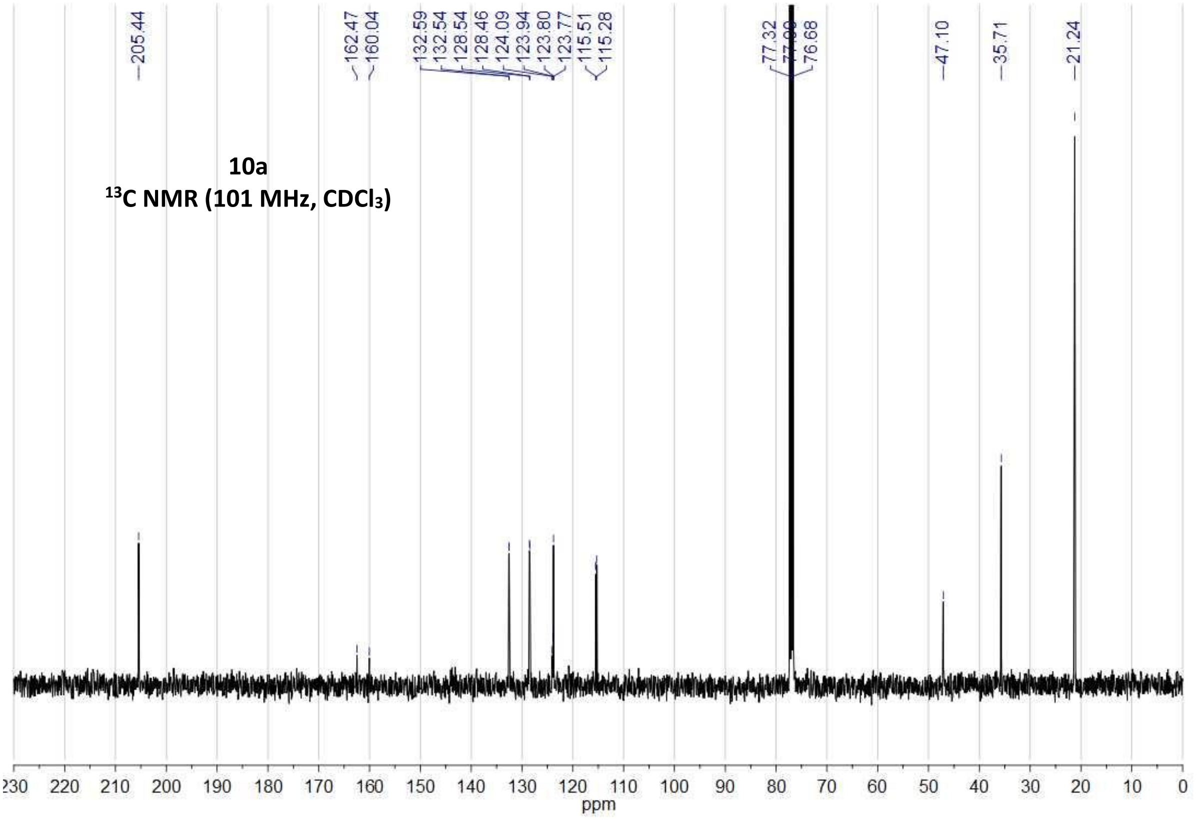

10a
^{13}C NMR (101 MHz, $CDCl_3$)

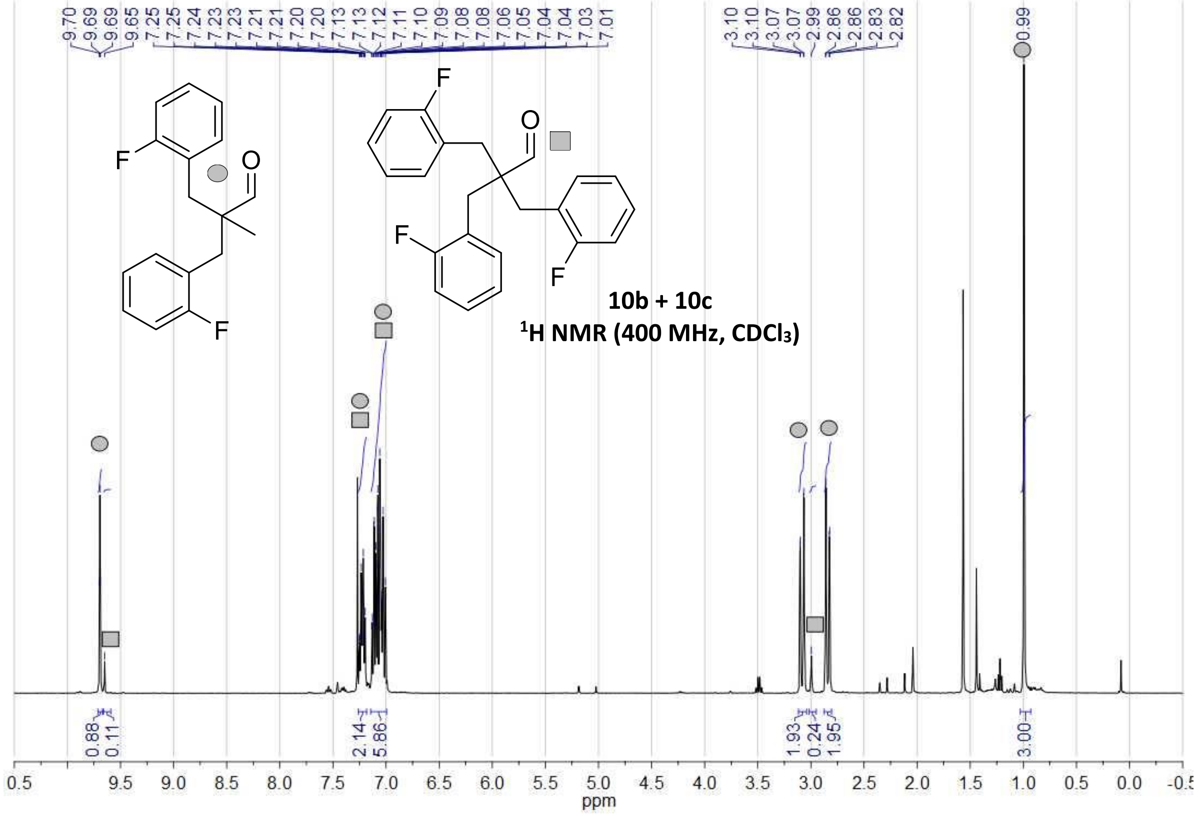
10b + 10c
¹H NMR (400 MHz, CDCl₃)

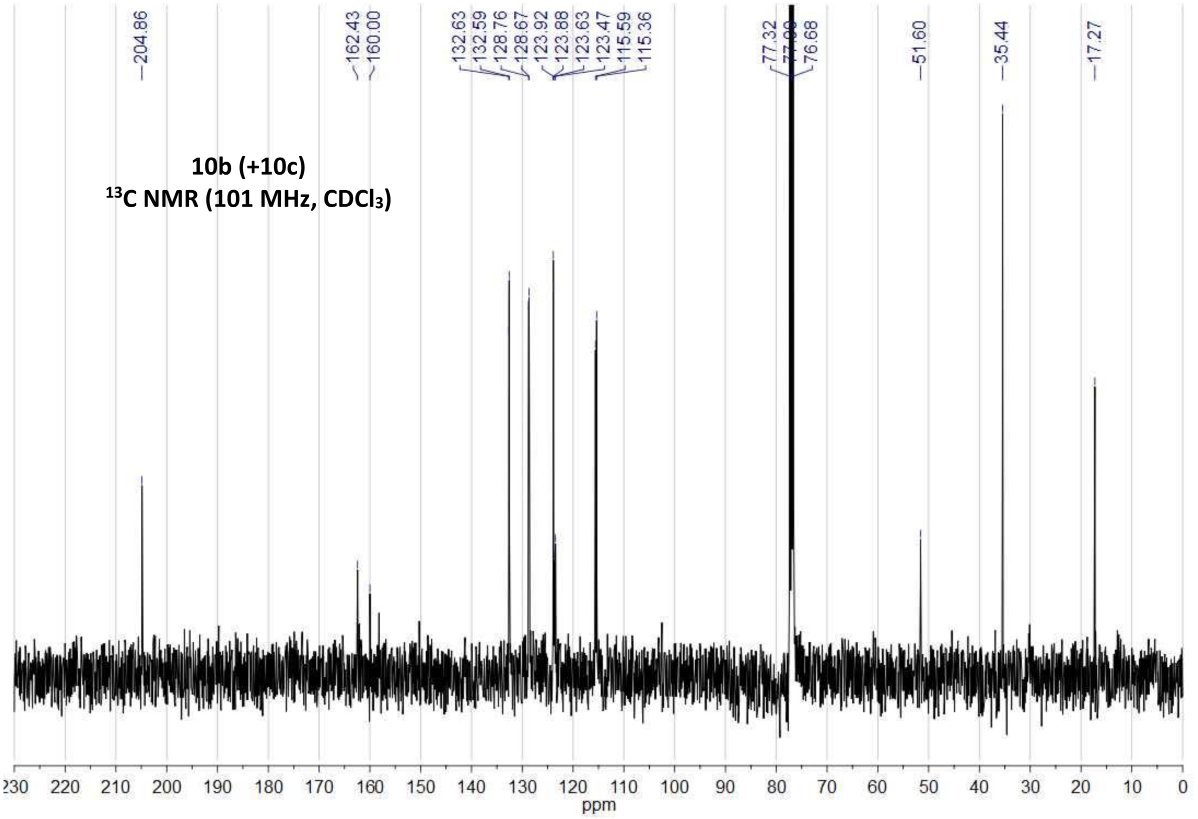
10b (+10c)
¹³C NMR (101 MHz, CDCl₃)

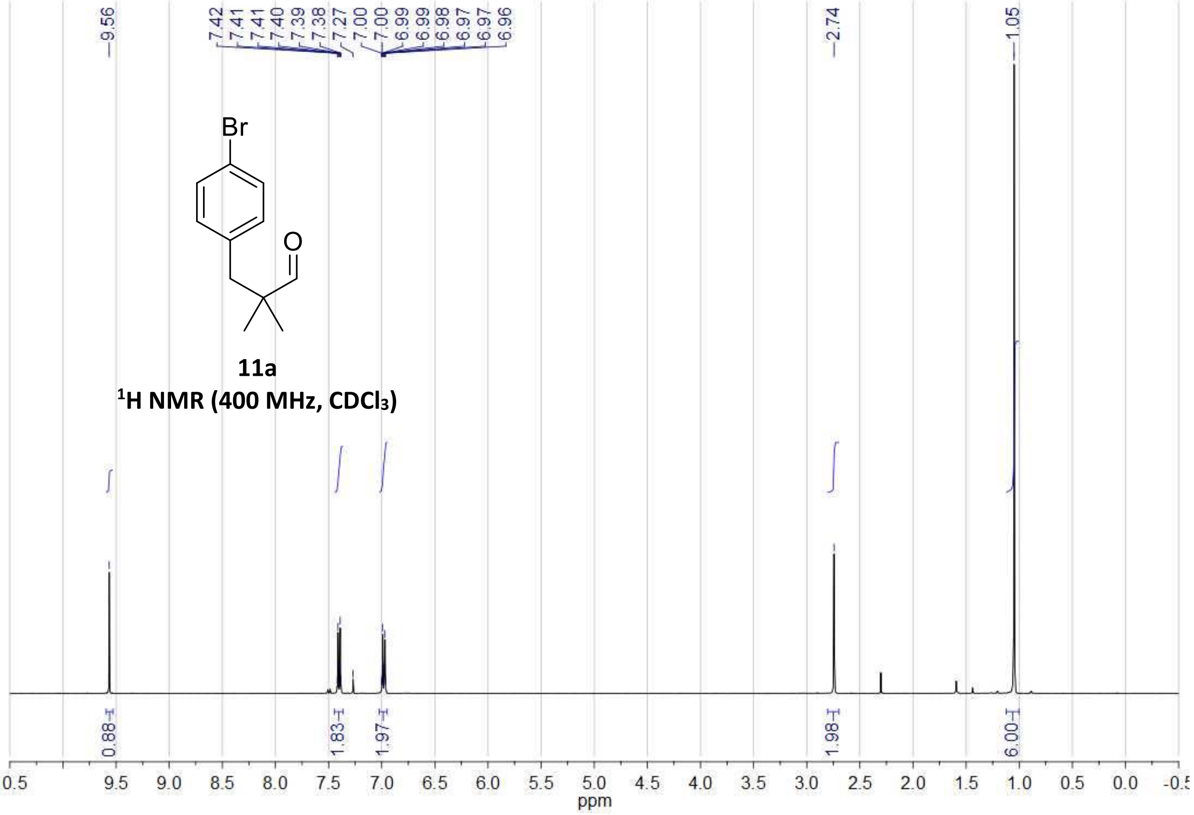
9.56
7.42 7.41 7.41 7.40 7.39 7.38 7.27 7.00 7.00 6.99 6.99 6.98 6.97 6.97 6.96
2.74
1.05
Br
O
11a
^{1}H NMR (400 MHz, CDCl₃)
0.88 1.83 1.97 1.98 6.00
ppm

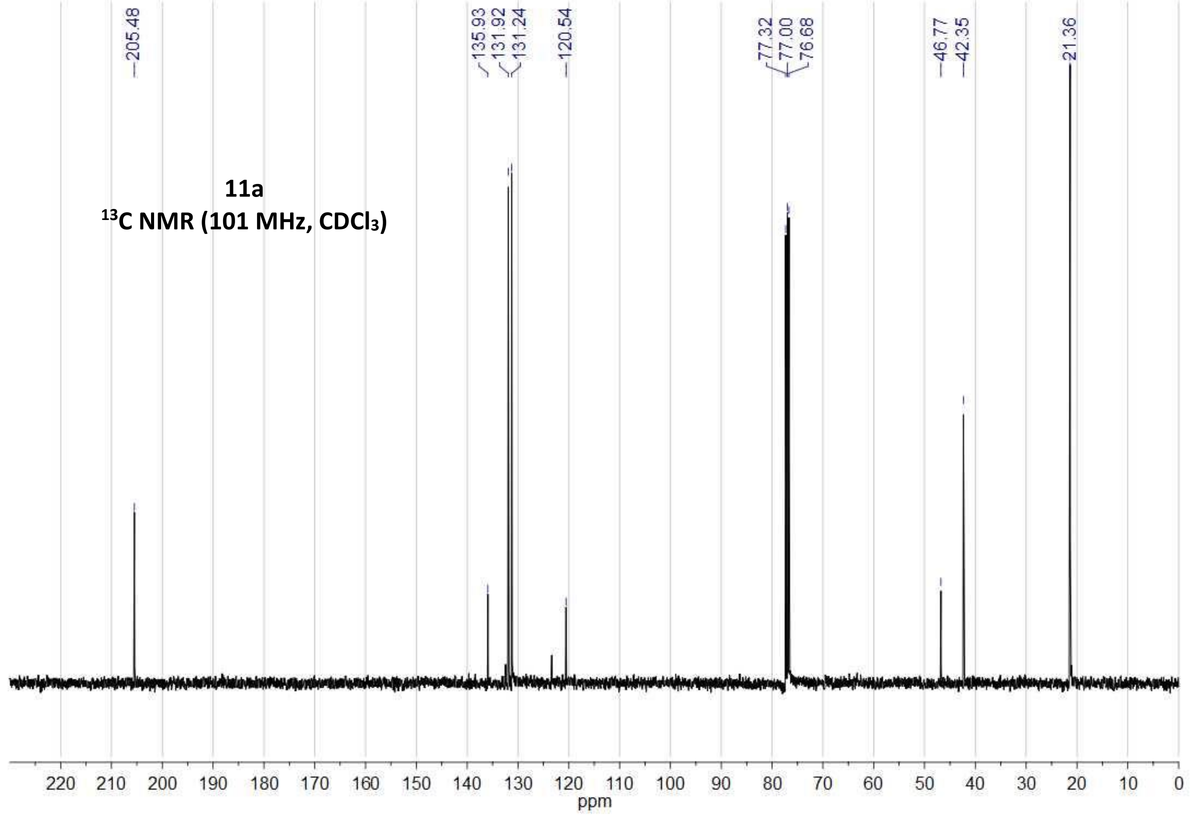
205.48
135.93 131.92 131.24
120.54
77.32 77.00 76.68
46.77 42.35
21.36
11a
^{13}C NMR (101 MHz, CDCl₃)
ppm

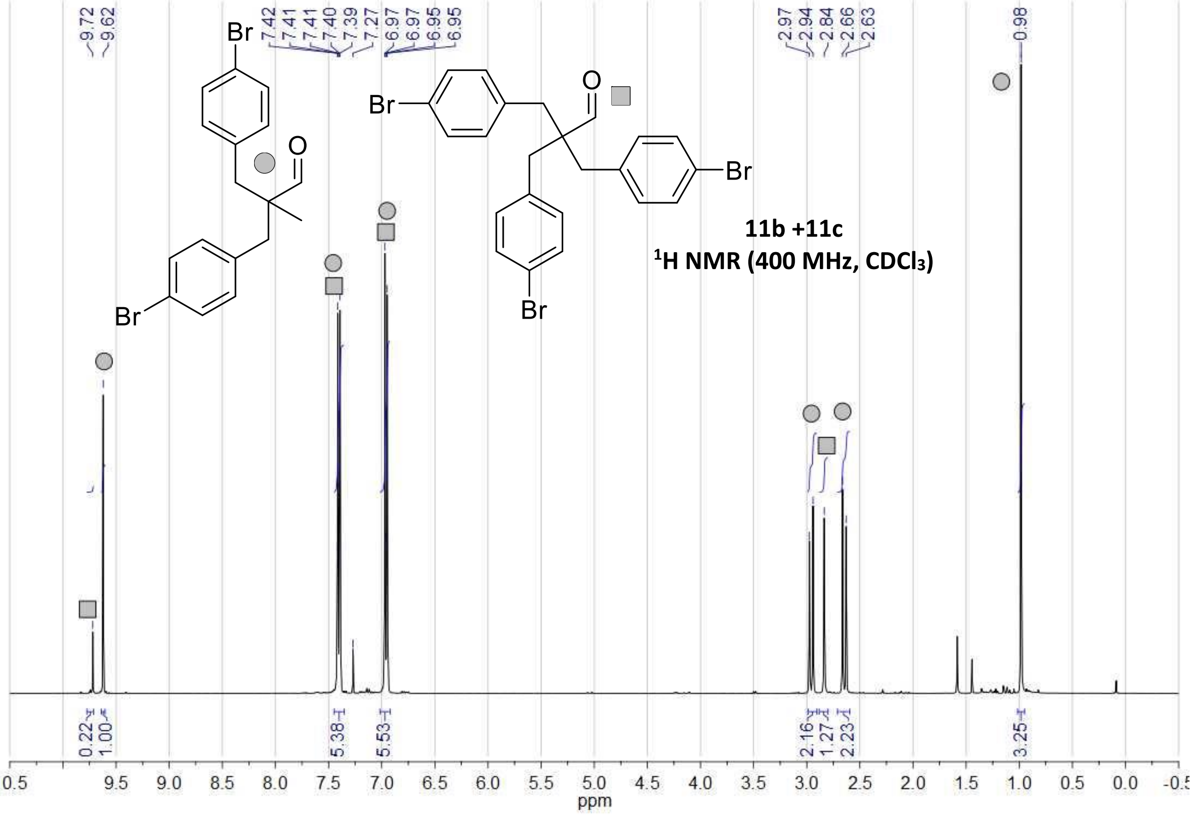
11b +11c
^{1}H NMR (400 MHz, CDCl$_3$)
Br
O
9.72
9.62
7.42
7.41
7.41
7.40
7.39
7.27
6.97
6.97
6.95
6.95
2.97
2.94
2.84
2.66
2.63
0.98
0.22
1.00
5.38
5.53
2.16
1.27
2.23
3.25
ppm

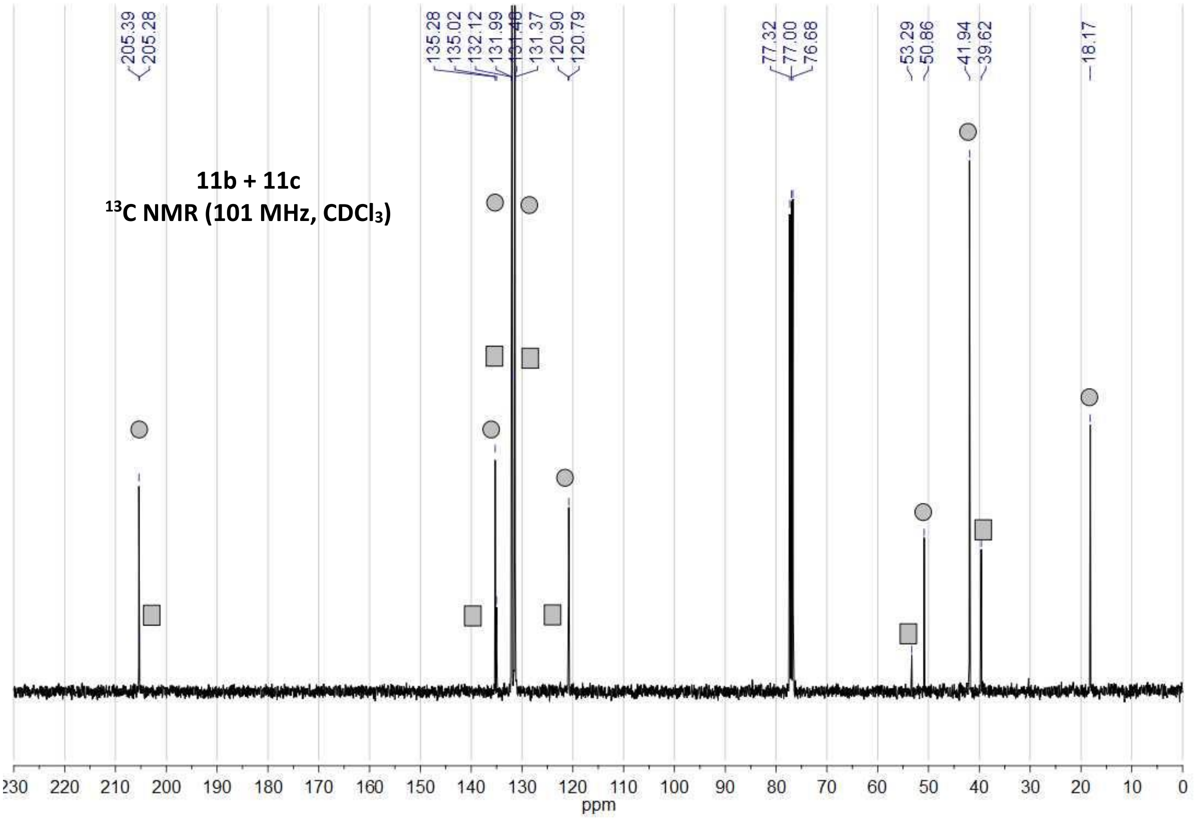
11b + 11c
^{13}C NMR (101 MHz, CDCl$_3$)
205.39
205.28
135.28
135.02
132.12
131.99
131.46
131.37
120.90
120.79
77.32
77.00
76.68
53.29
50.86
41.94
39.62
18.17
ppm

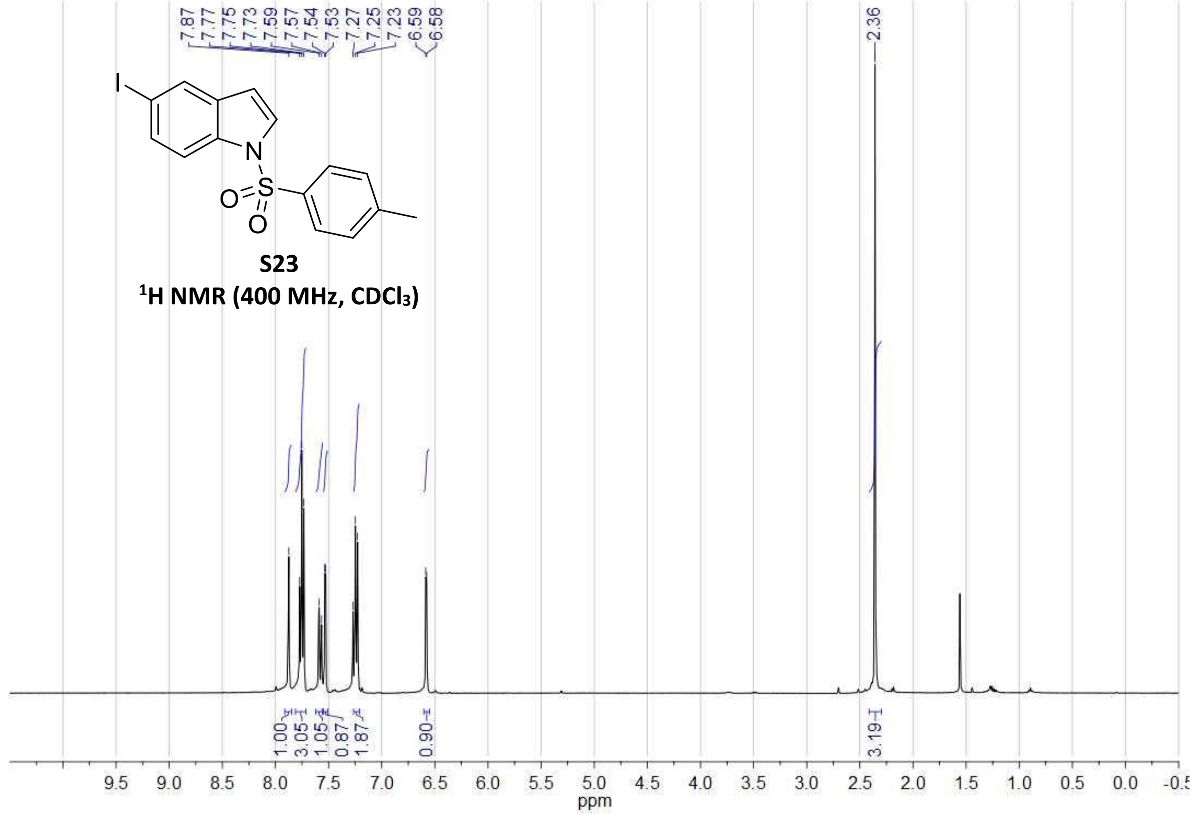

S23
^{1}H NMR (400 MHz, $CDCl_3$)

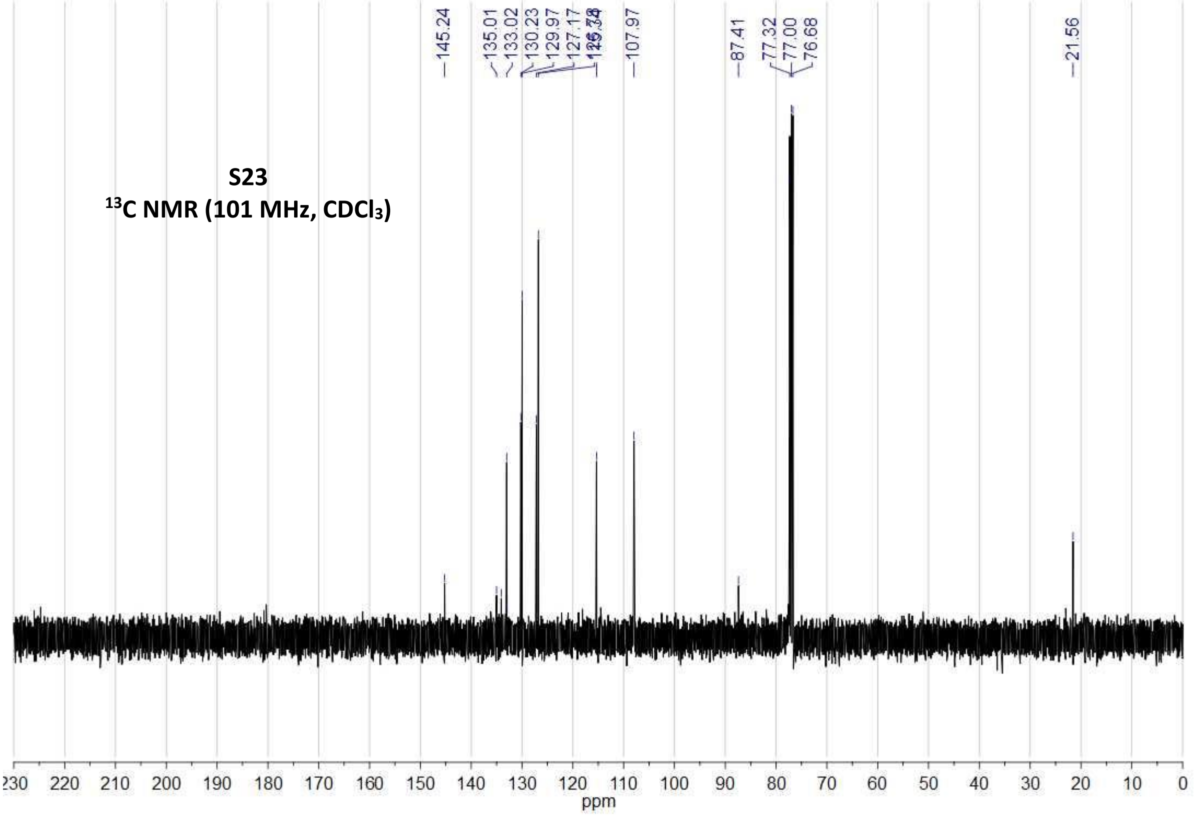

S23
^{13}C NMR (101 MHz, $CDCl_3$)

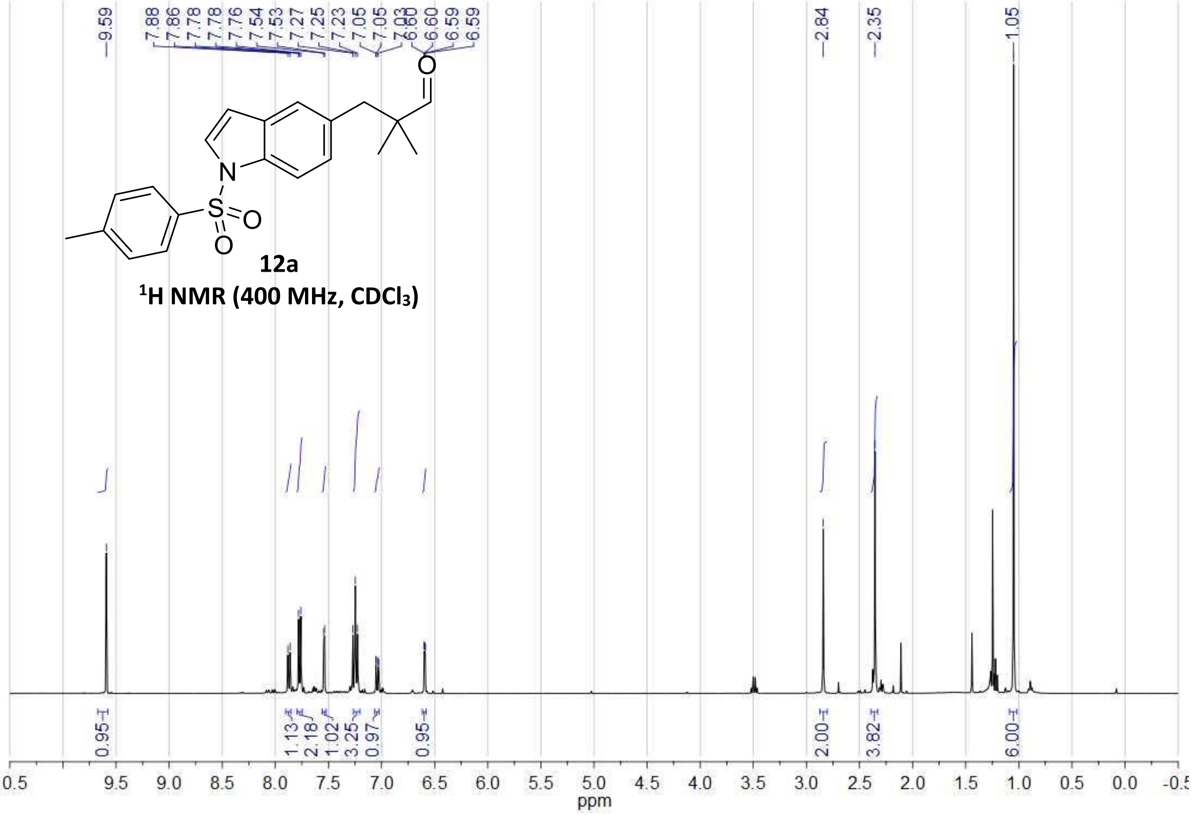

12a

^{1}H NMR (400 MHz, $CDCl_3$)

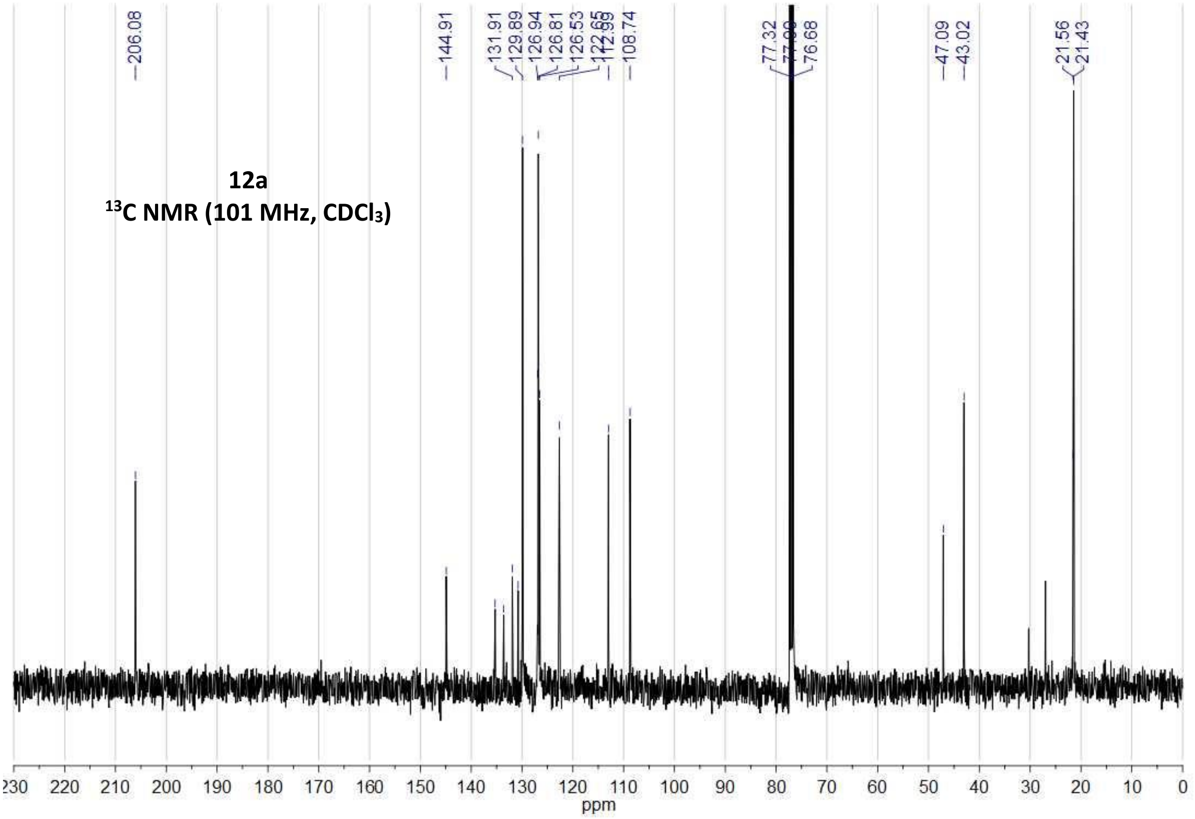

12a

^{13}C NMR (101 MHz, $CDCl_3$)

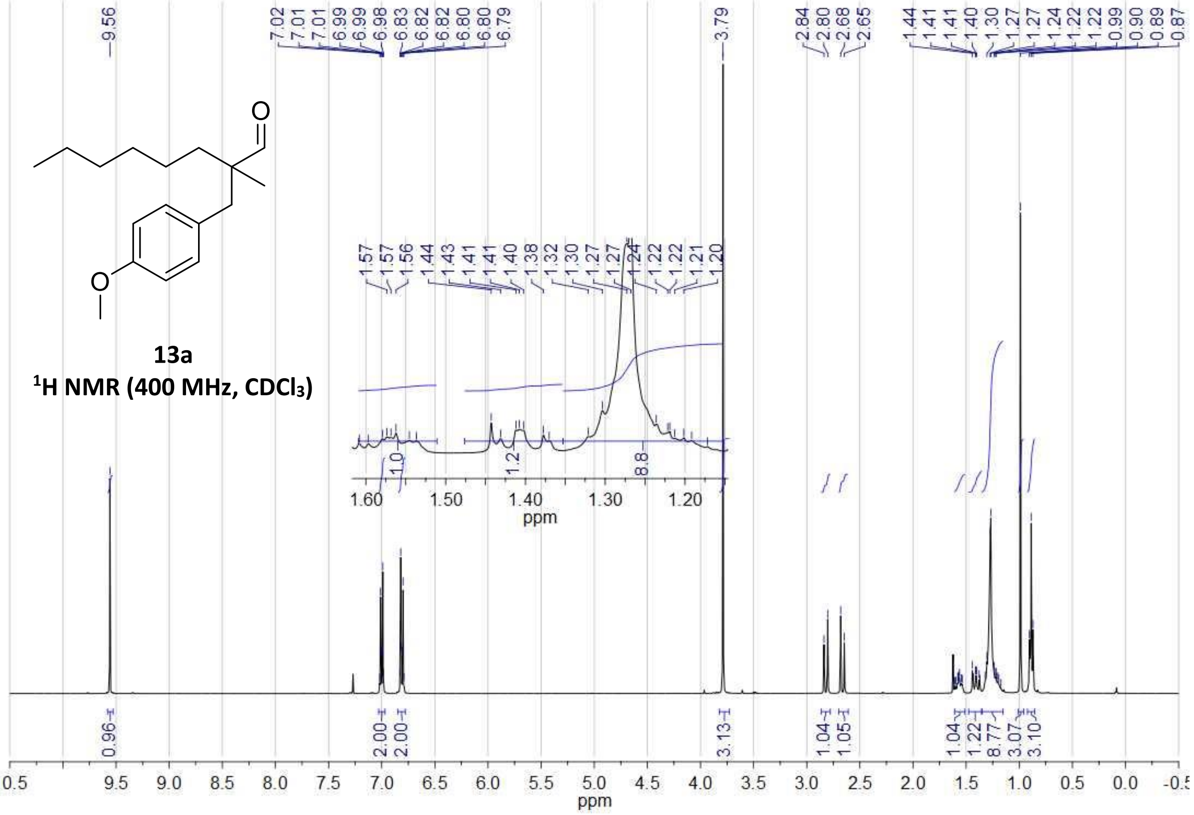
13a
1H NMR (400 MHz, CDCl3)
ppm

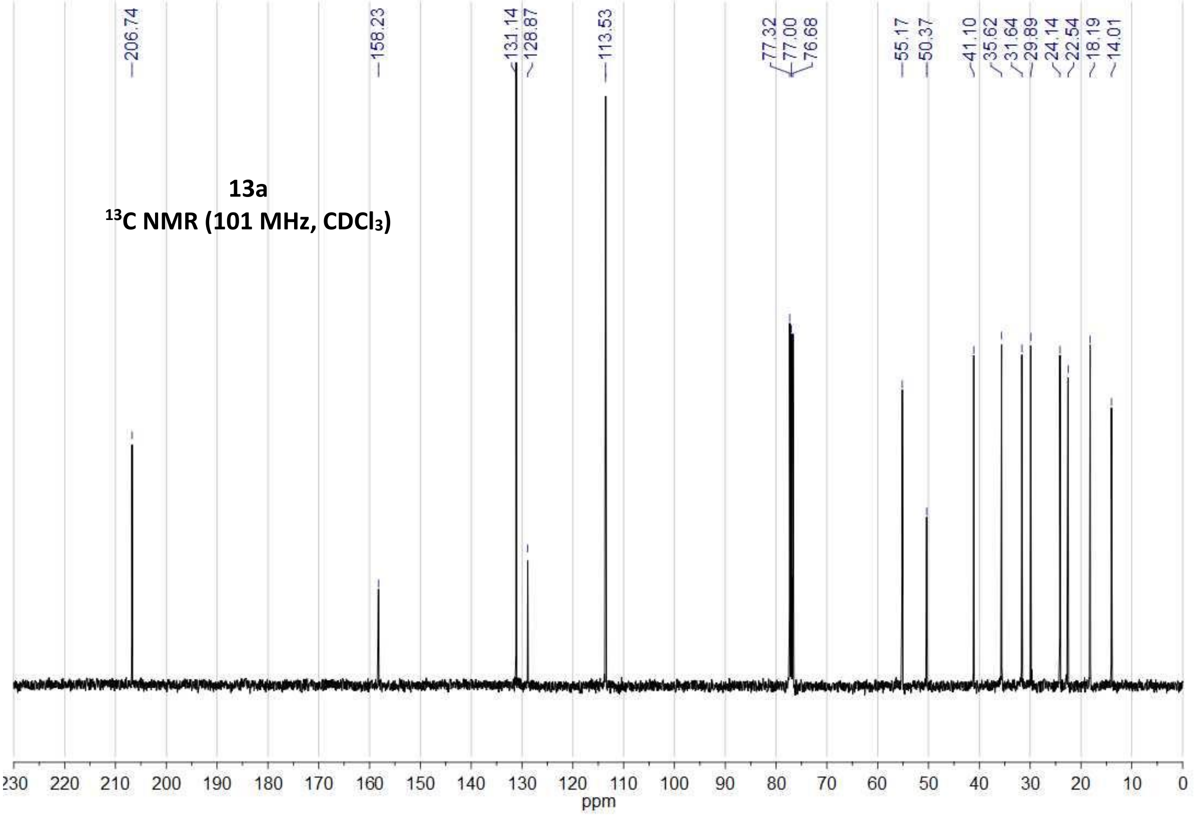
13a
13C NMR (101 MHz, CDCl3)
ppm

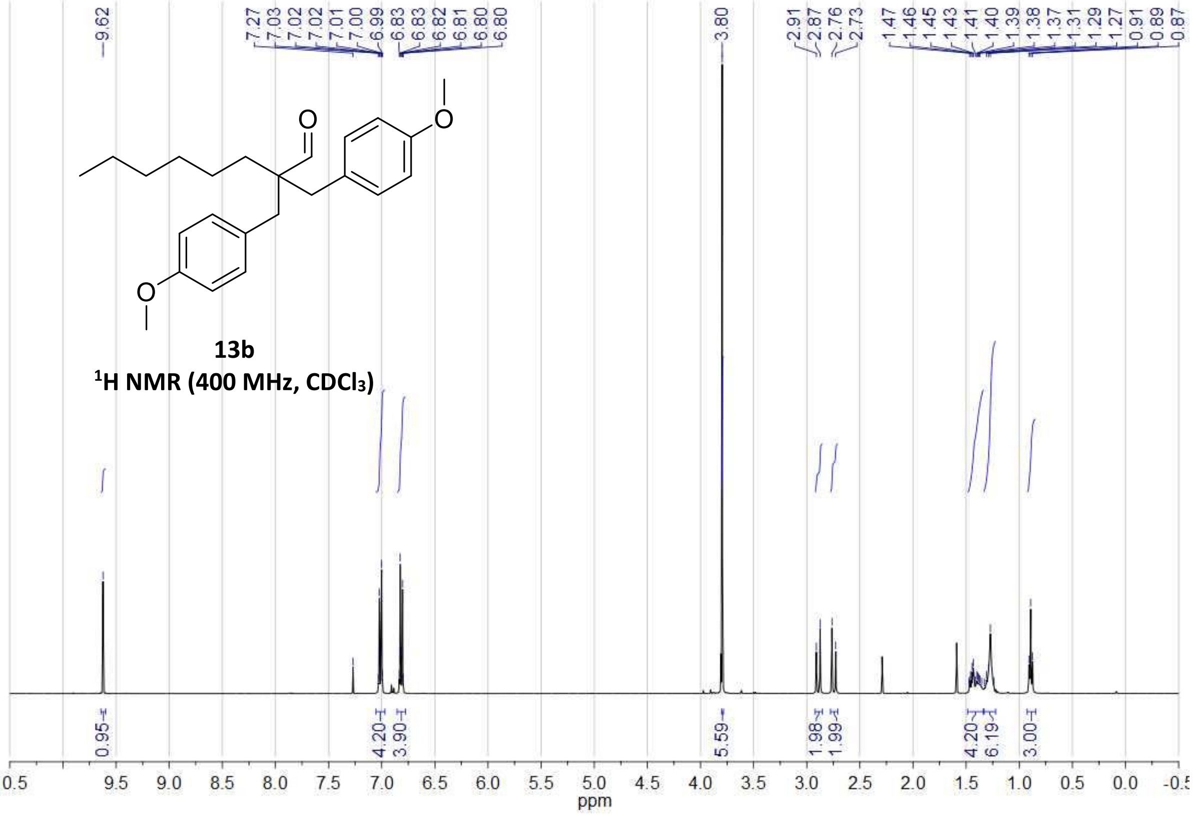
13b
1H NMR (400 MHz, CDCl3)
ppm

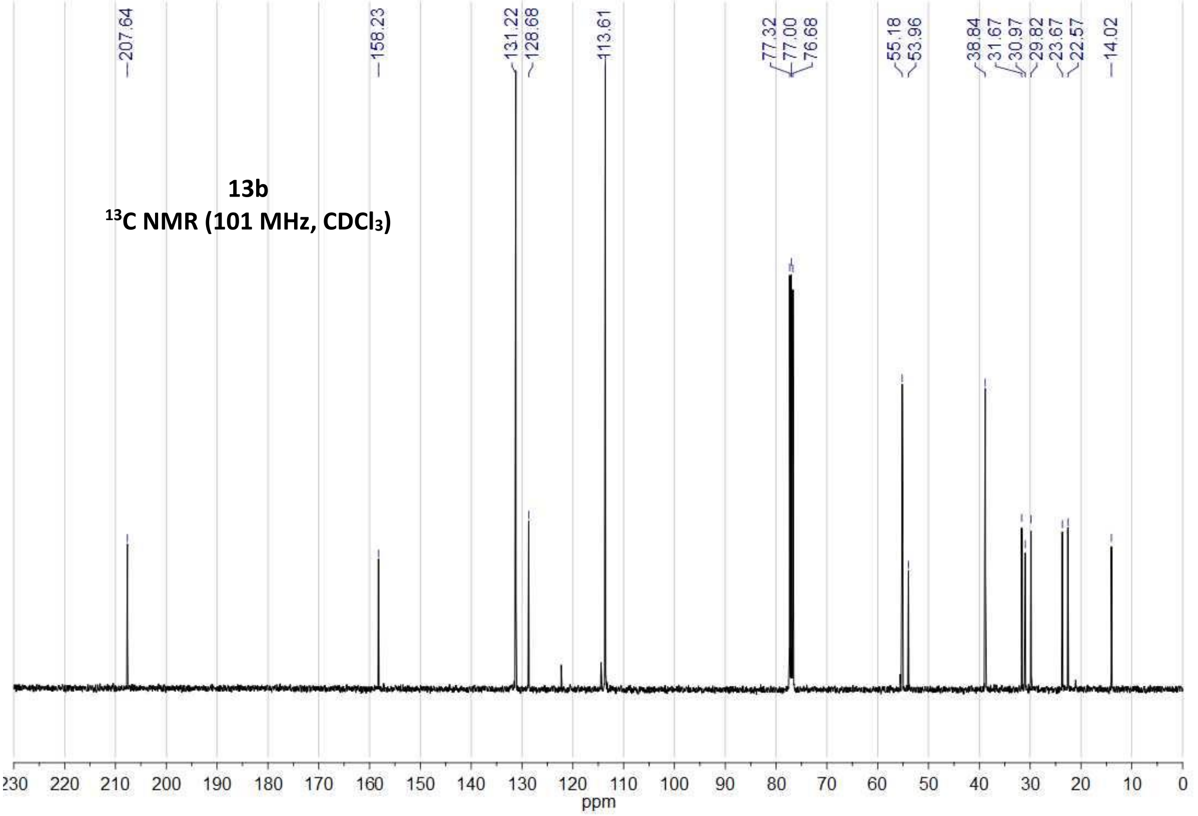
13b
13C NMR (101 MHz, CDCl3)
ppm

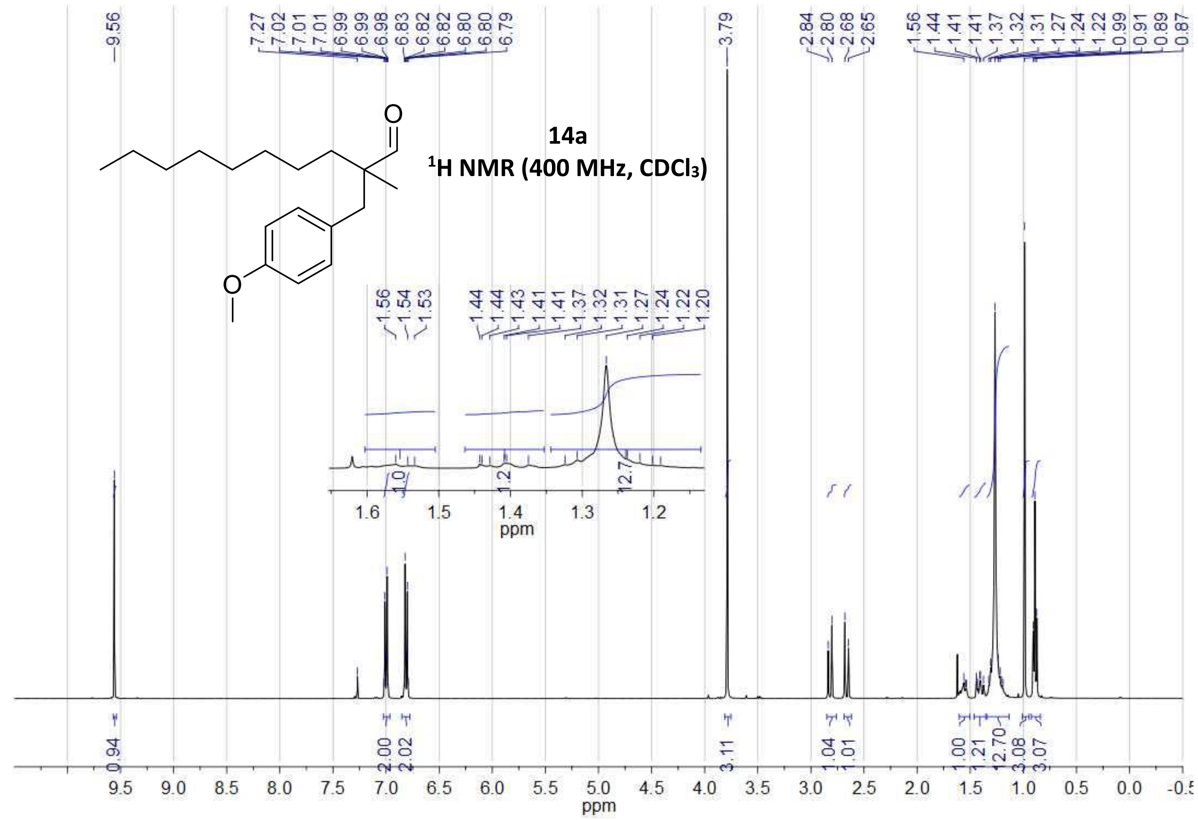
14a
^{1}H NMR (400 MHz, CDCl$_3$)
9.56
7.27 7.02 7.01 7.01 6.99 6.99 6.98 6.83 6.82 6.82 6.80 6.80 6.79
3.79
2.84 2.80 2.68 2.65
1.56 1.44 1.41 1.41 1.37 1.32 1.31 1.27 1.24 1.22 0.99 0.91 0.89 0.87
1.56 1.54 1.53 1.44 1.44 1.43 1.41 1.41 1.37 1.32 1.31 1.27 1.24 1.22 1.20
0.94 2.00 2.02 3.11 1.04 1.01 1.00 1.21 12.70 3.08 3.07
ppm

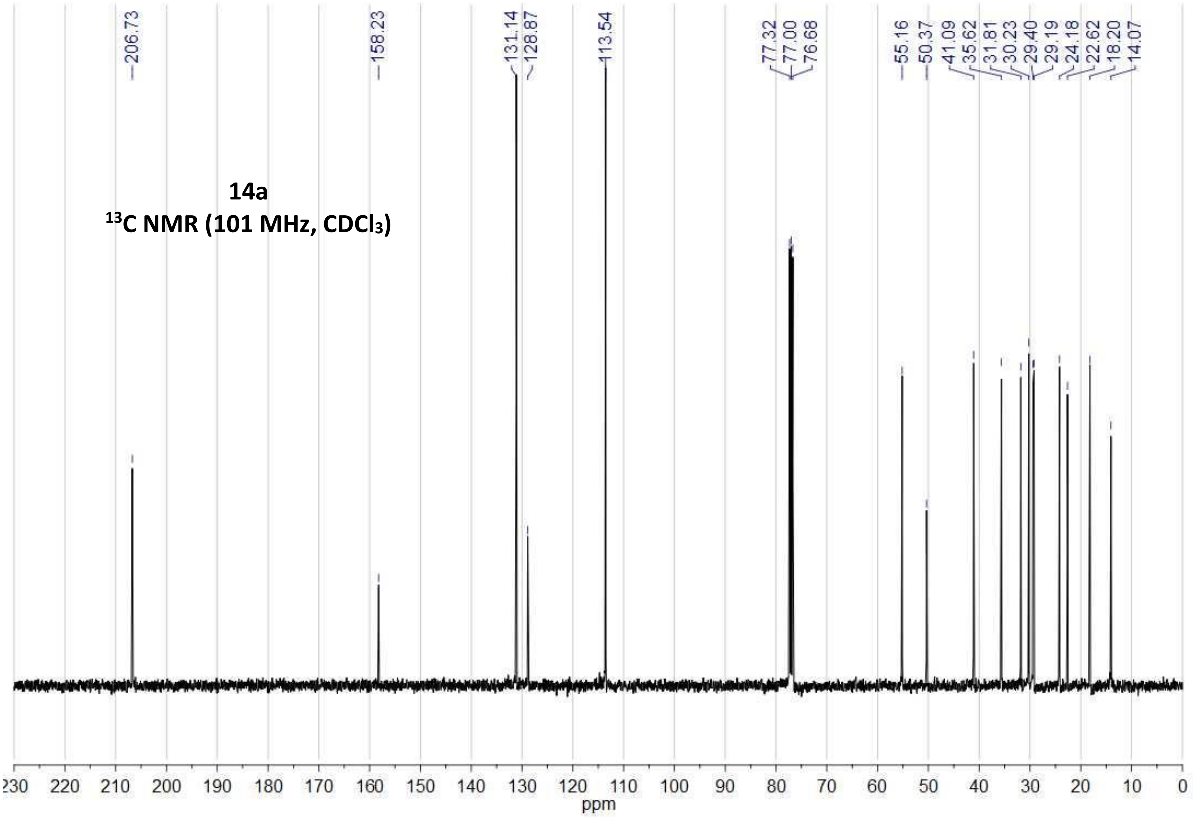
14a
^{13}C NMR (101 MHz, CDCl$_3$)
206.73 158.23 131.14 128.87 113.54 77.32 77.00 76.68 55.16 50.37 41.09 35.62 31.81 30.23 29.40 29.19 24.18 22.62 18.20 14.07
ppm

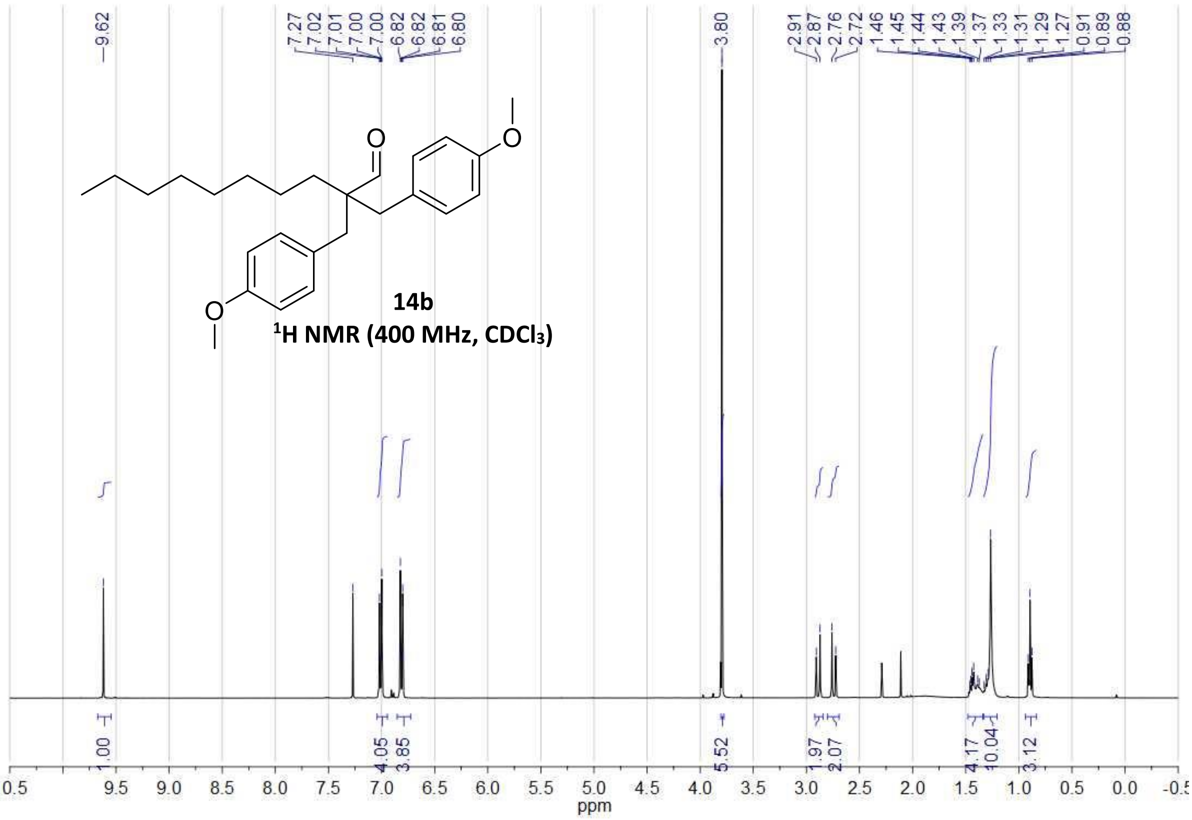
9.62
7.27 7.02 7.01 7.00 7.00 6.82 6.82 6.81 6.80
3.80
2.91 2.87 2.76 2.72 1.46 1.45 1.44 1.43 1.39 1.37 1.33 1.31 1.29 1.27 0.91 0.89 0.88
O
O
O
14b
1H NMR (400 MHz, CDCl3)
1.00 4.05 3.85 5.52 1.97 2.07 4.17 10.04 3.12
ppm

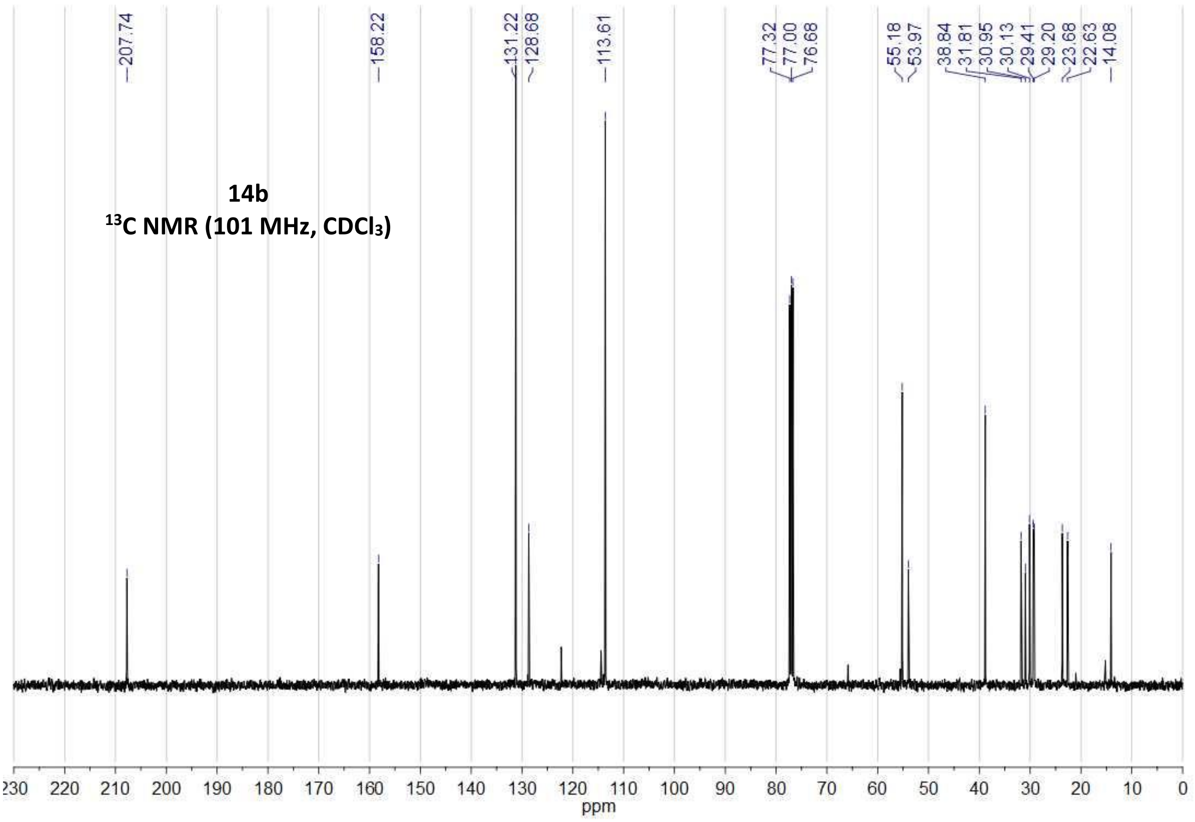
207.74
158.22
131.22 128.68
113.61
77.32 77.00 76.68
55.18 53.97
38.84 31.81 30.95 30.13 29.41 29.20 23.68 22.63 14.08
14b
13C NMR (101 MHz, CDCl3)
ppm

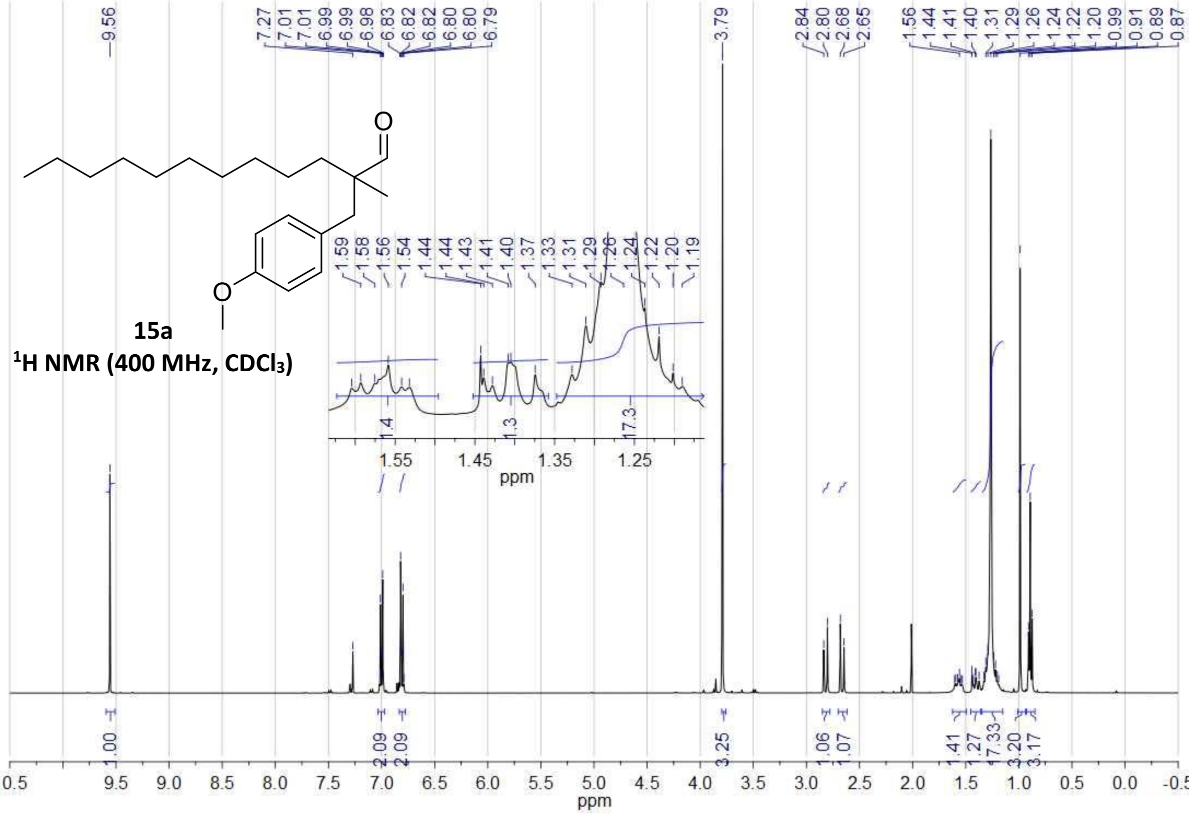

15a
^{1}H NMR (400 MHz, CDCl$_3$)

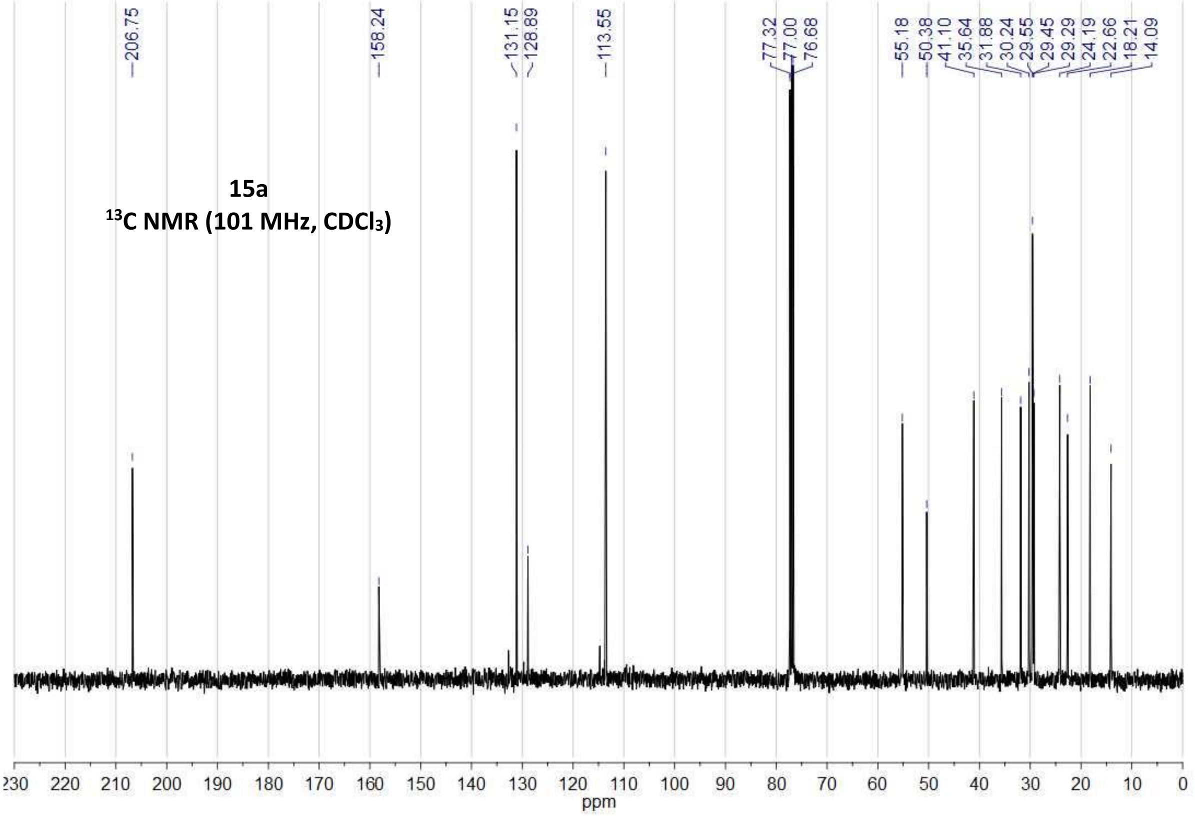

15a
^{13}C NMR (101 MHz, CDCl$_3$)

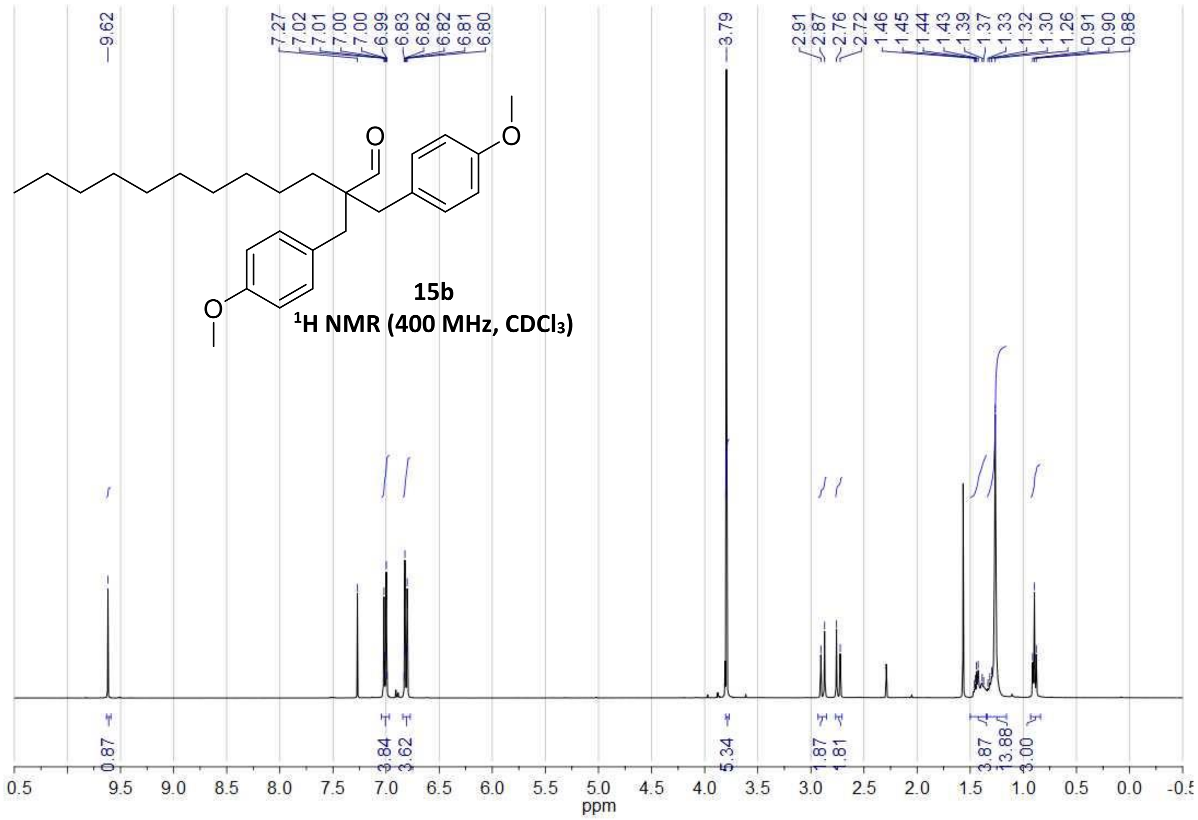
9.62
7.27
7.02
7.01
7.00
7.00
6.99
6.83
6.82
6.82
6.81
6.80
3.79
2.91
2.87
2.76
2.72
1.46
1.45
1.44
1.43
1.39
1.37
1.33
1.32
1.30
1.26
0.91
0.90
0.88
O
O
O
15b
1H NMR (400 MHz, CDCl3)
0.87
3.84
3.62
5.34
1.87
1.81
3.87
13.88
3.00
ppm

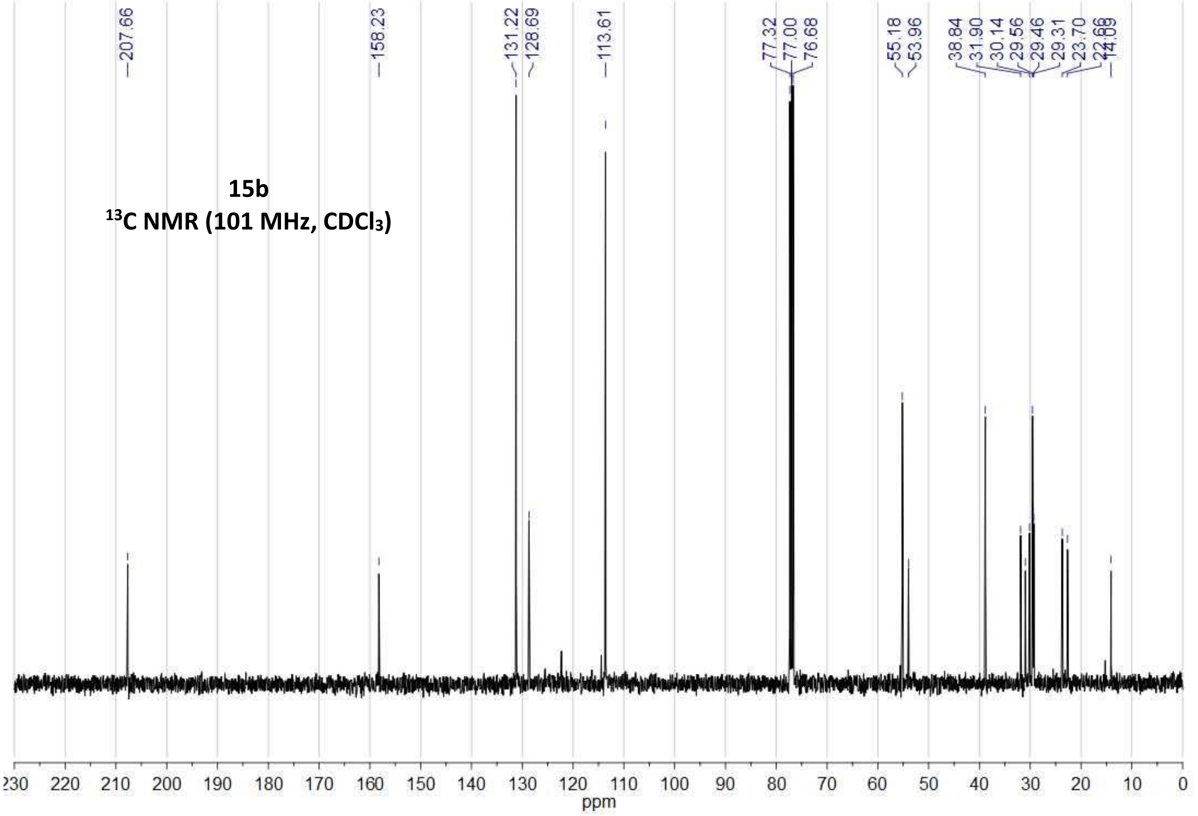
207.66
158.23
131.22
128.69
113.61
77.32
77.00
76.68
55.18
53.96
38.84
31.90
30.14
29.56
29.46
29.31
23.70
22.66
14.09
15b
13C NMR (101 MHz, CDCl3)
ppm

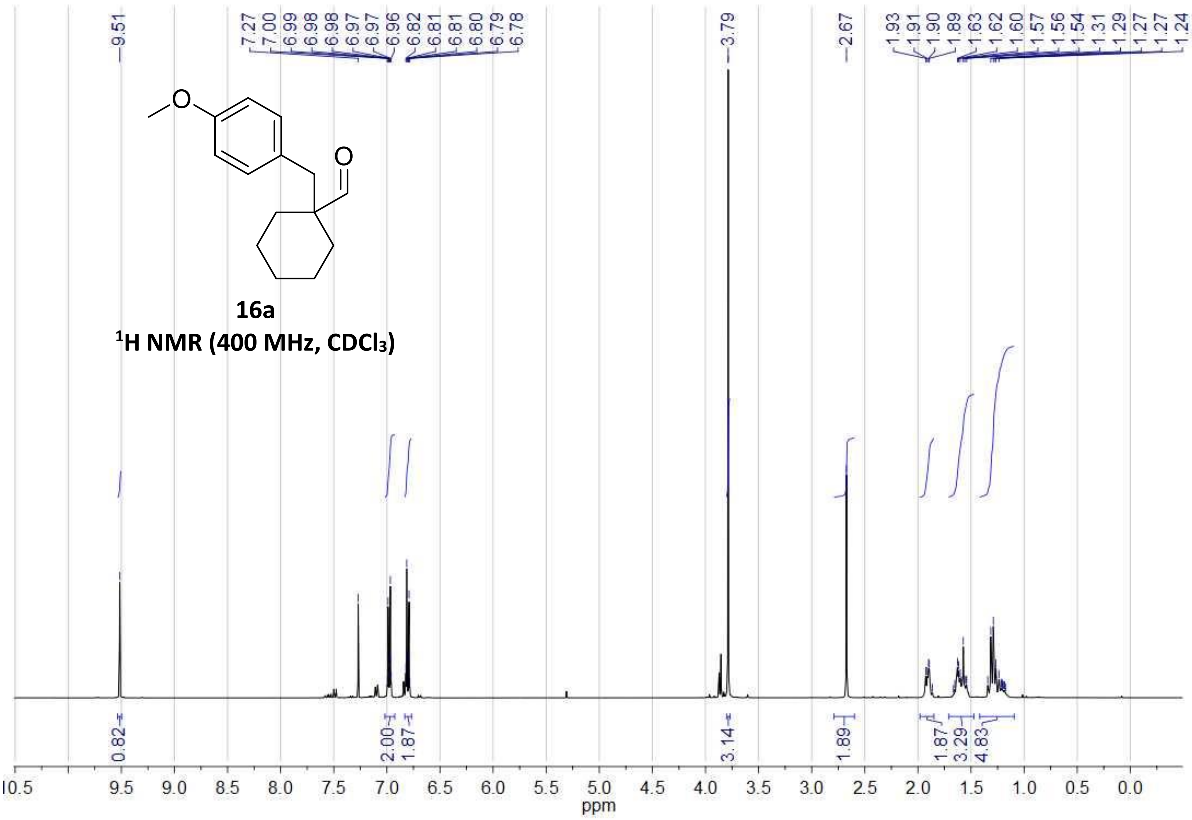

16a

^{1}H NMR (400 MHz, $CDCl_3$)

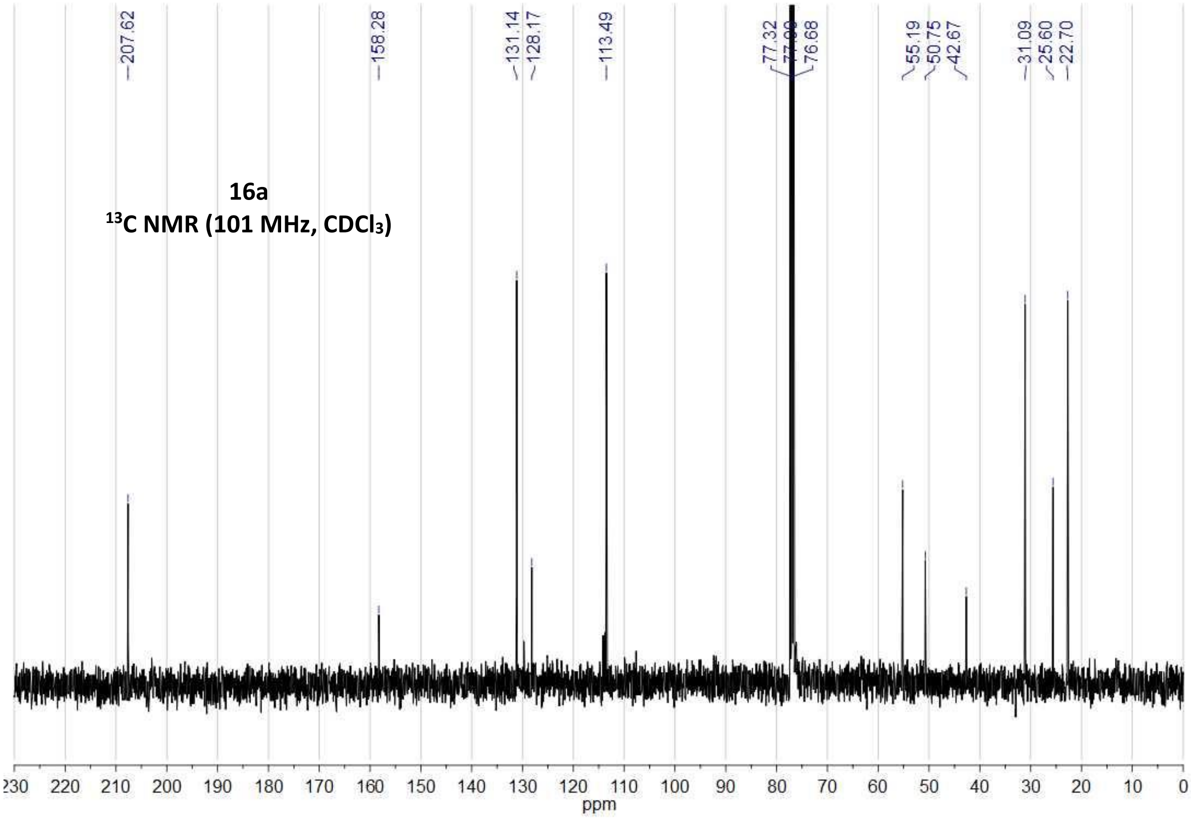

16a

^{13}C NMR (101 MHz, $CDCl_3$)

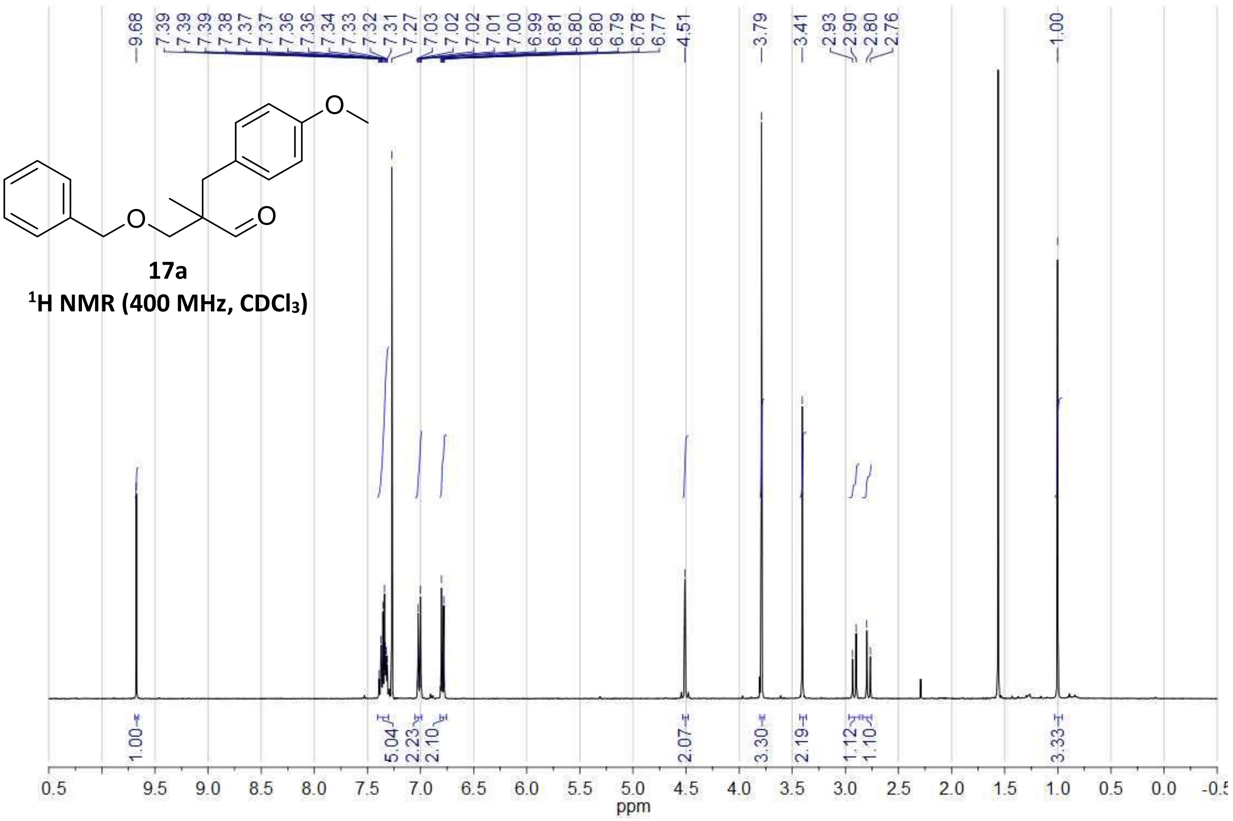
17a
^{1}H NMR (400 MHz, CDCl$_3$)
ppm

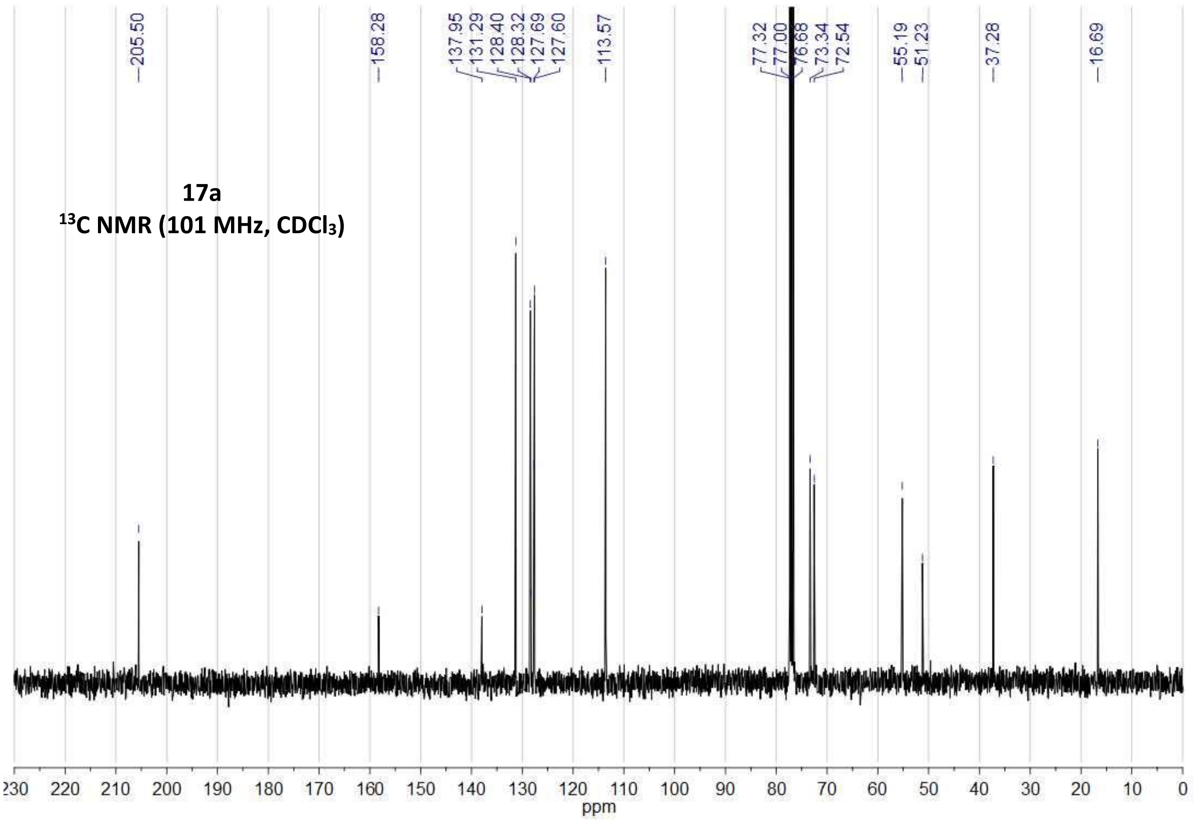
17a
^{13}C NMR (101 MHz, CDCl$_3$)
ppm

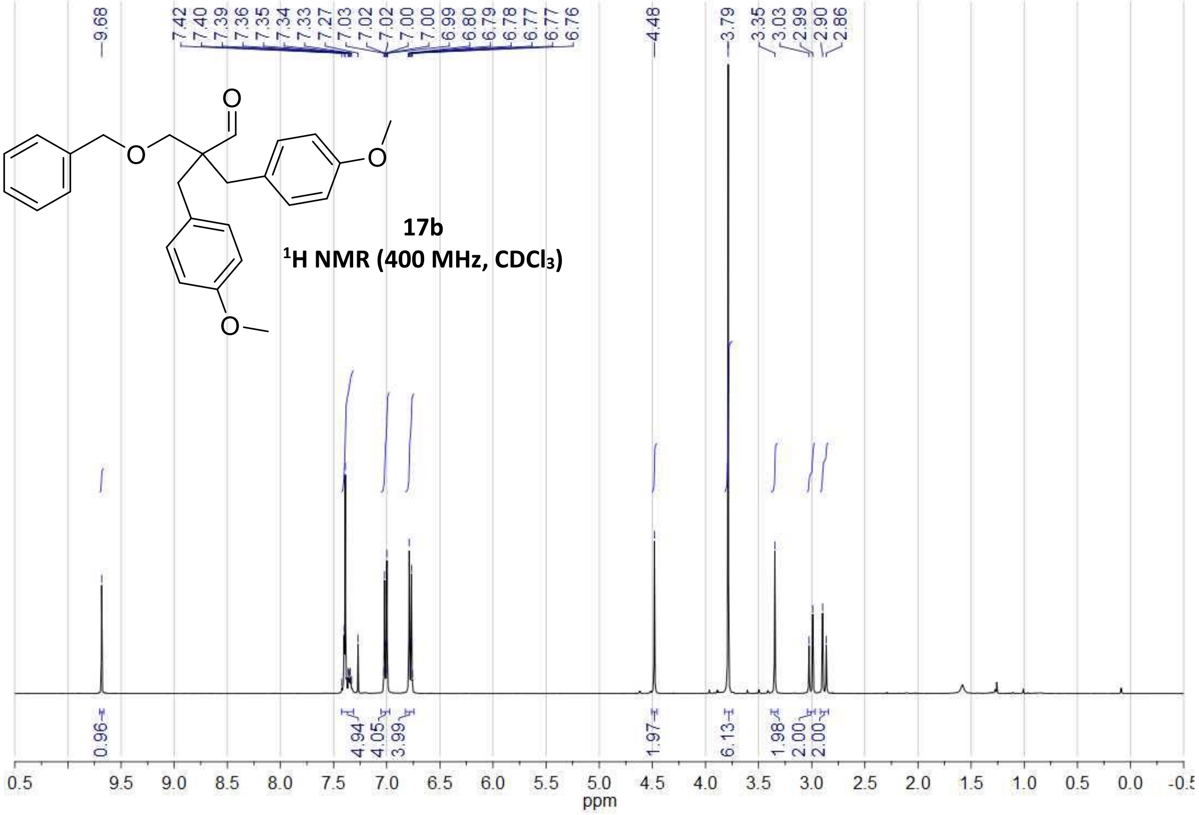
17b
^{1}H NMR (400 MHz, CDCl$_3$)
9.68
7.42 7.40 7.39 7.36 7.35 7.34 7.33 7.27 7.03 7.02 7.02 7.00 6.99 6.80 6.79 6.78 6.77 6.77 6.76
4.48
3.79
3.35 3.03 2.99 2.90 2.86
0.96 4.94 4.05 3.99 1.97 6.13 1.98 2.00 2.00
ppm

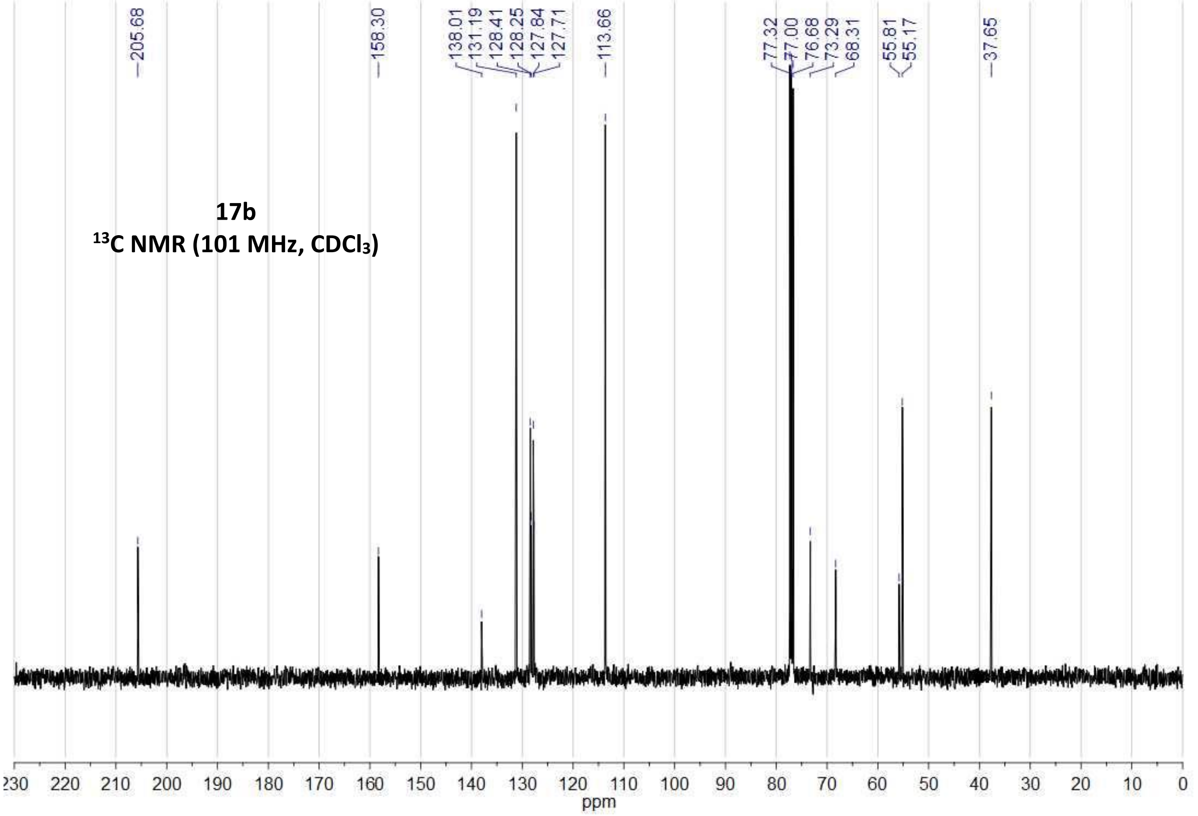
17b
^{13}C NMR (101 MHz, CDCl$_3$)
205.68
158.30
138.01 131.19 128.41 128.25 127.84 127.71
113.66
77.32 77.00 76.68 73.29 68.31
55.81 55.17
37.65
ppm

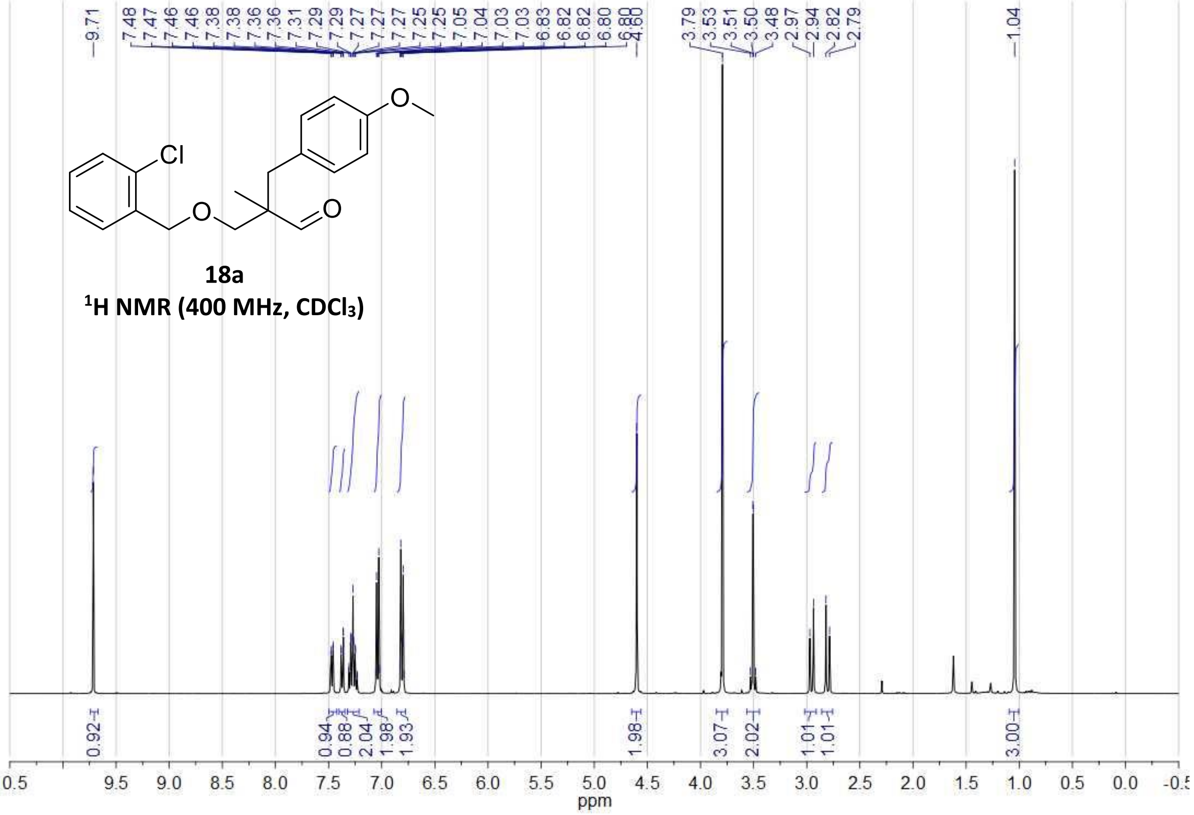

18a

^{1}H NMR (400 MHz, CDCl$_3$)

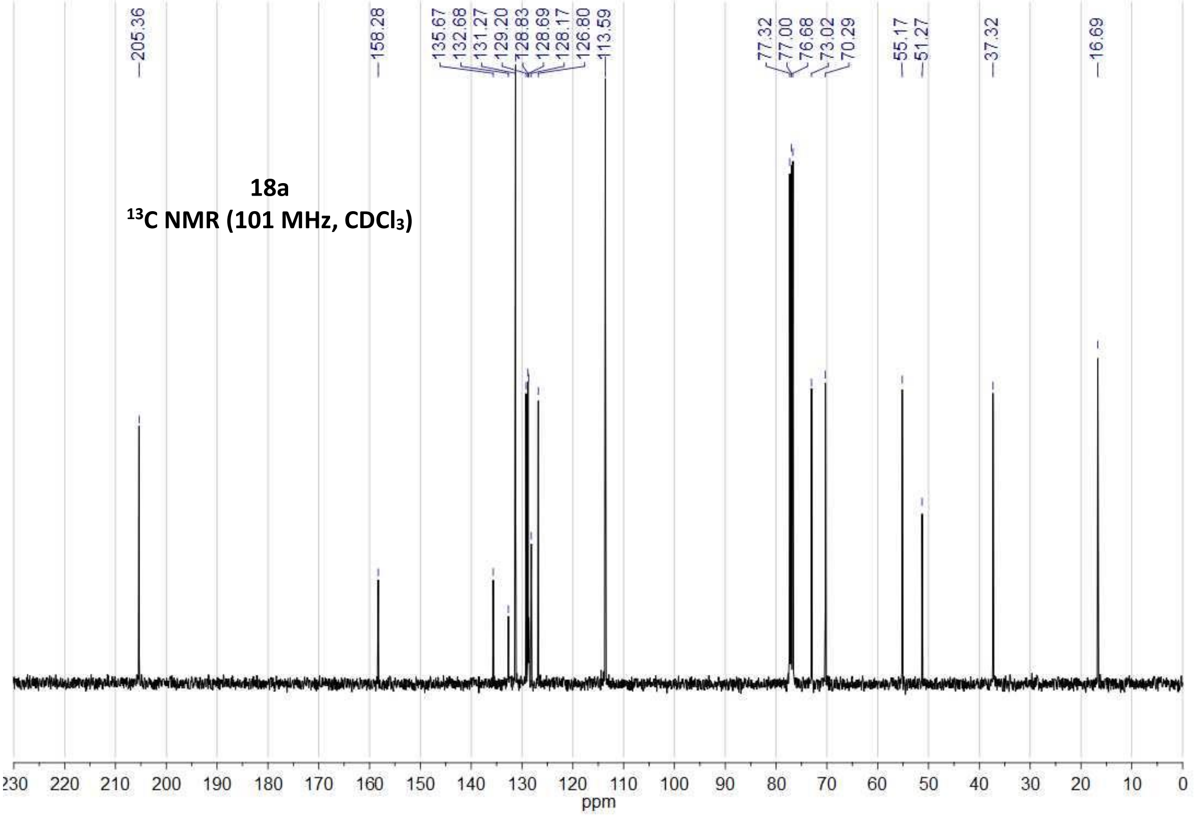

18a

^{13}C NMR (101 MHz, CDCl$_3$)

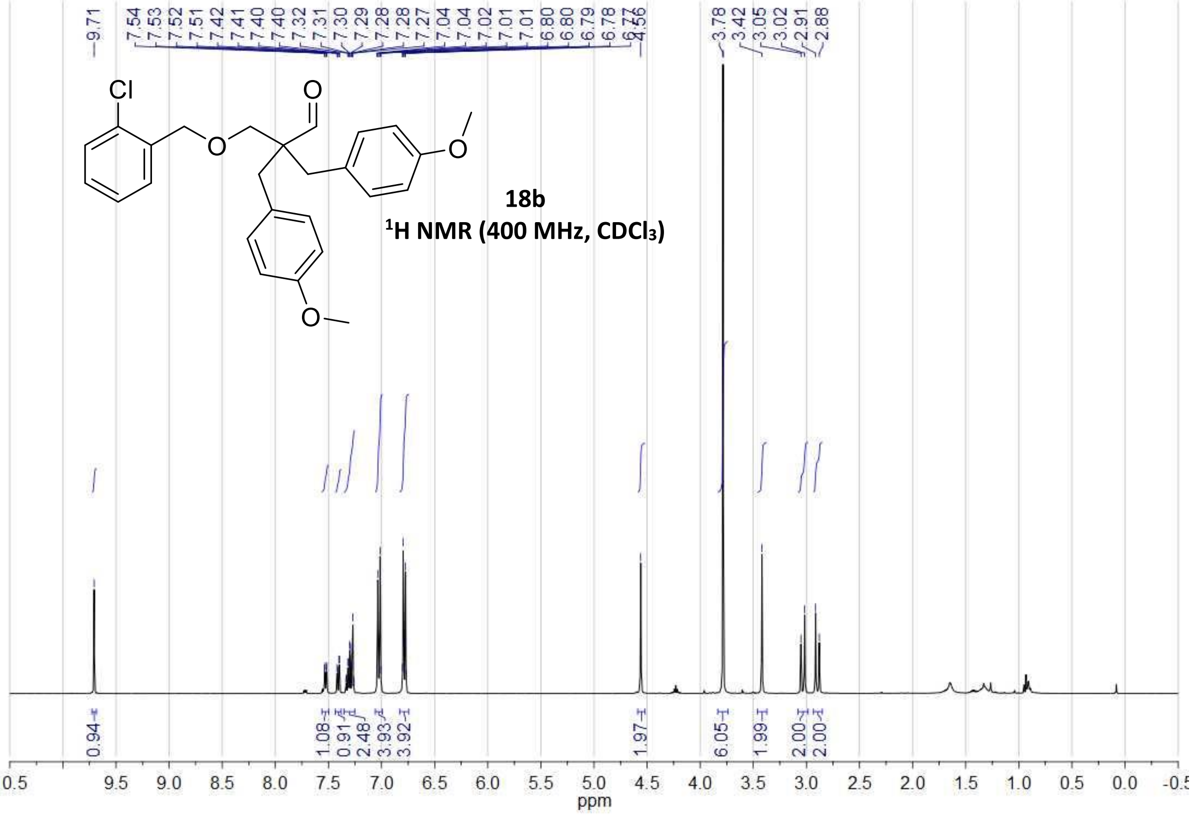
18b
^{1}H NMR (400 MHz, CDCl$_3$)
ppm

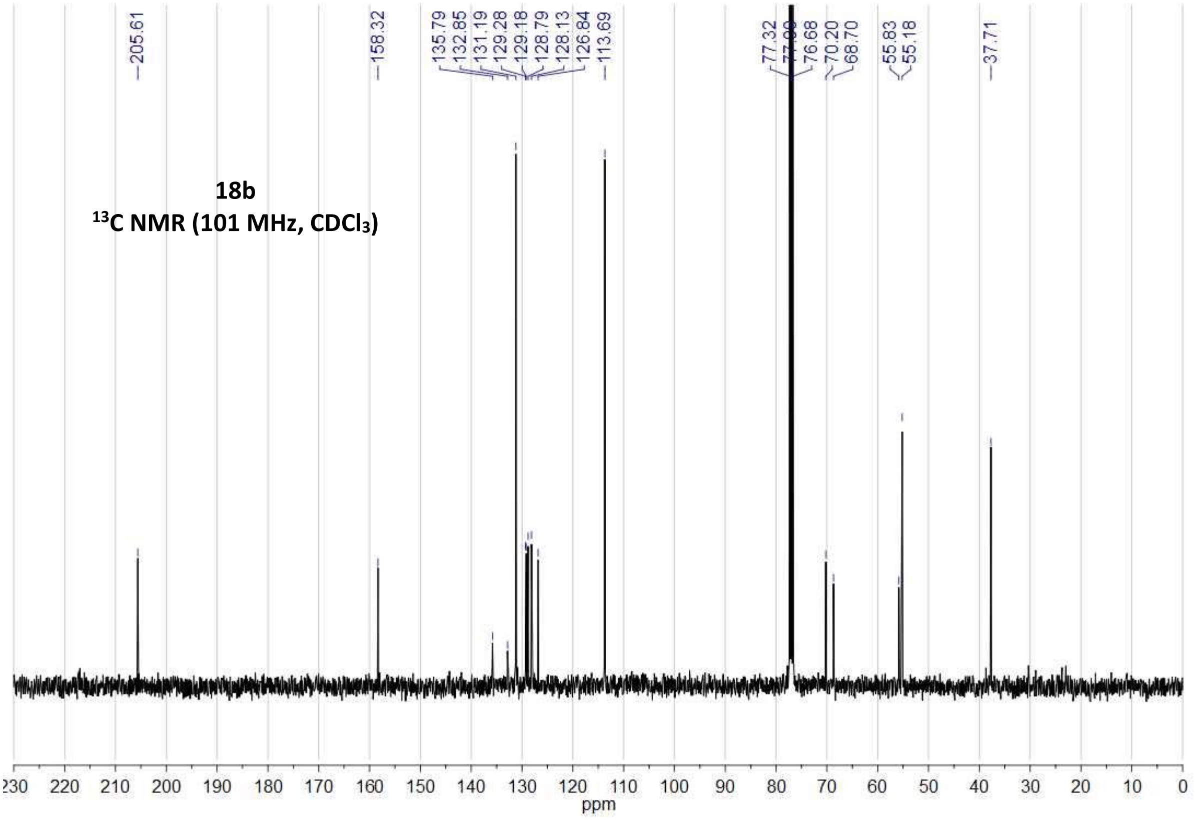
18b
^{13}C NMR (101 MHz, CDCl$_3$)
ppm

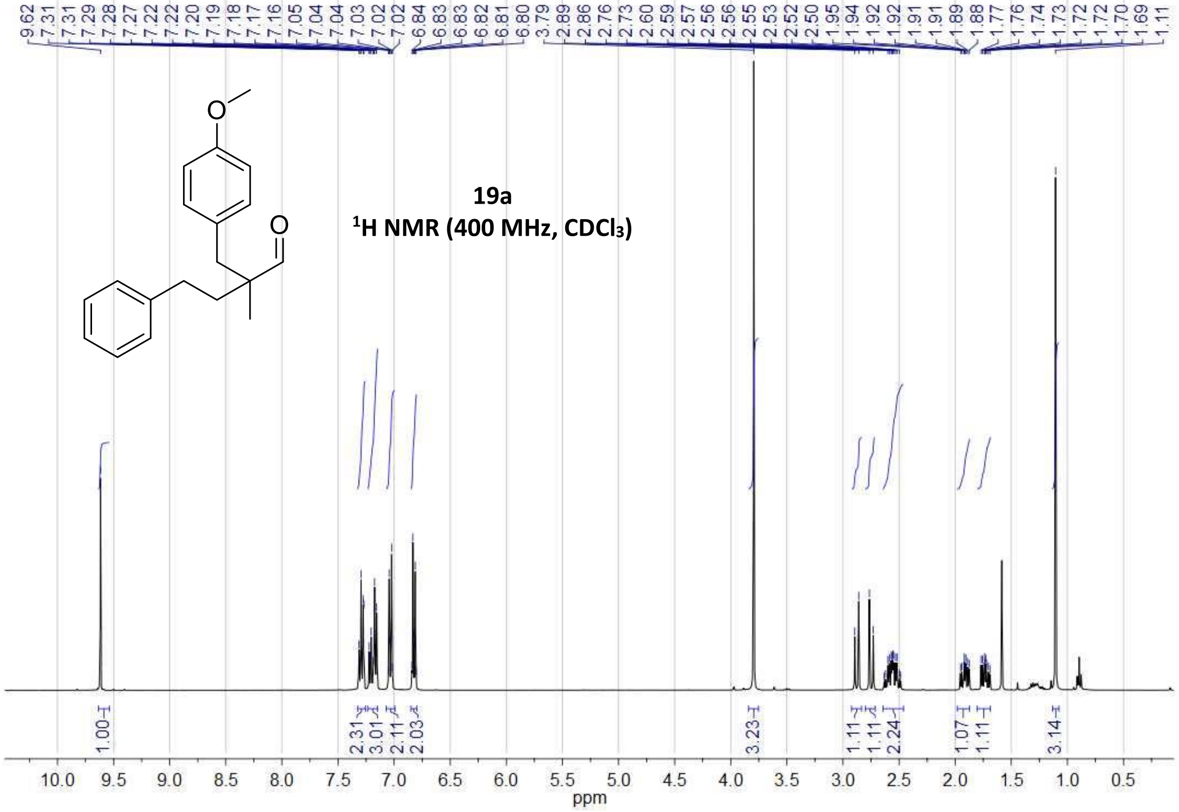
19a
^{1}H NMR (400 MHz, CDCl$_3$)
O
9.62 7.31 7.29 7.28 7.27 7.22 7.22 7.20 7.19 7.18 7.17 7.16 7.05 7.04 7.04 7.03 7.02 7.02 6.84 6.83 6.83 6.82 6.81 6.80 3.79 2.89 2.86 2.76 2.73 2.60 2.59 2.57 2.56 2.56 2.55 2.53 2.52 2.50 1.95 1.94 1.92 1.92 1.91 1.91 1.89 1.88 1.77 1.76 1.74 1.73 1.72 1.70 1.69 1.11
1.00 2.31 3.01 2.11 2.03 3.23 1.11 1.11 2.24 1.07 1.11 3.14
10.0 9.5 9.0 8.5 8.0 7.5 7.0 6.5 6.0 5.5 5.0 4.5 4.0 3.5 3.0 2.5 2.0 1.5 1.0 0.5
ppm

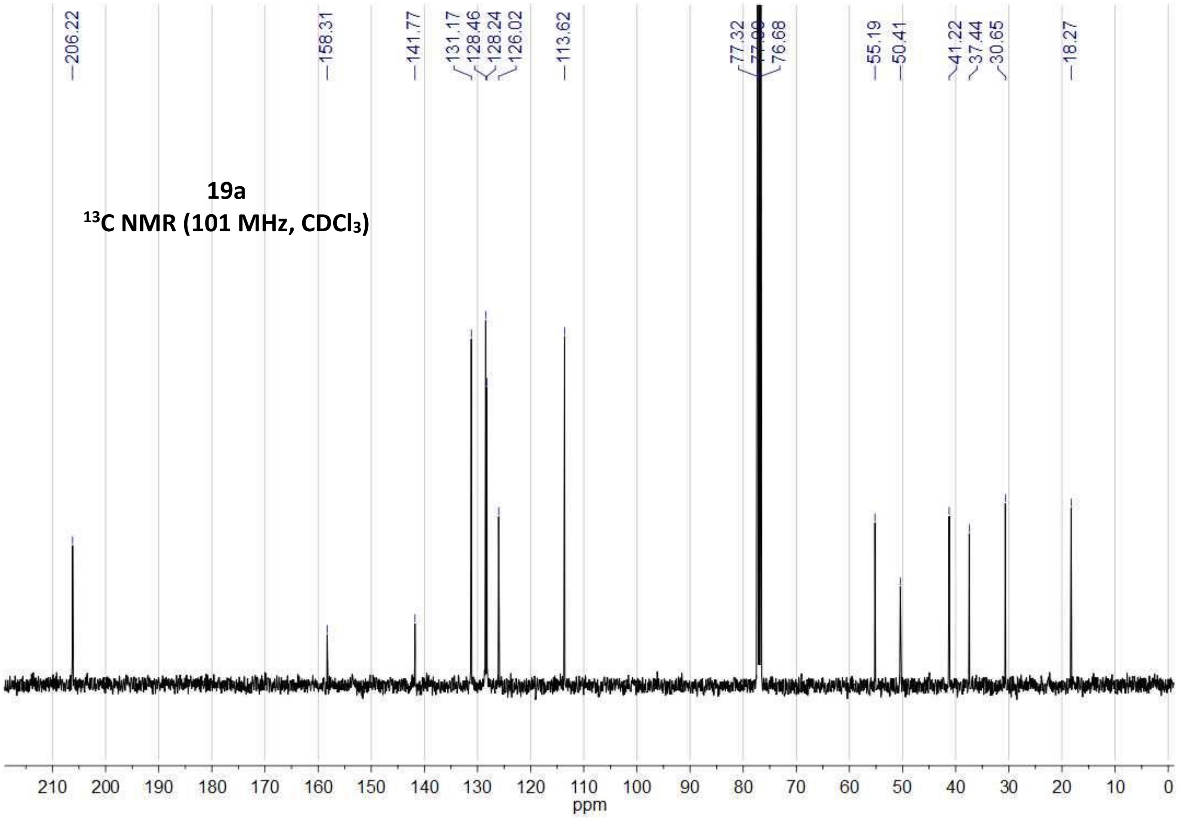
19a
^{13}C NMR (101 MHz, CDCl$_3$)
206.22 158.31 141.77 131.17 128.46 128.24 126.02 113.62 77.32 76.68 55.19 50.41 41.22 37.44 30.65 18.27
210 200 190 180 170 160 150 140 130 120 110 100 90 80 70 60 50 40 30 20 10 0
ppm

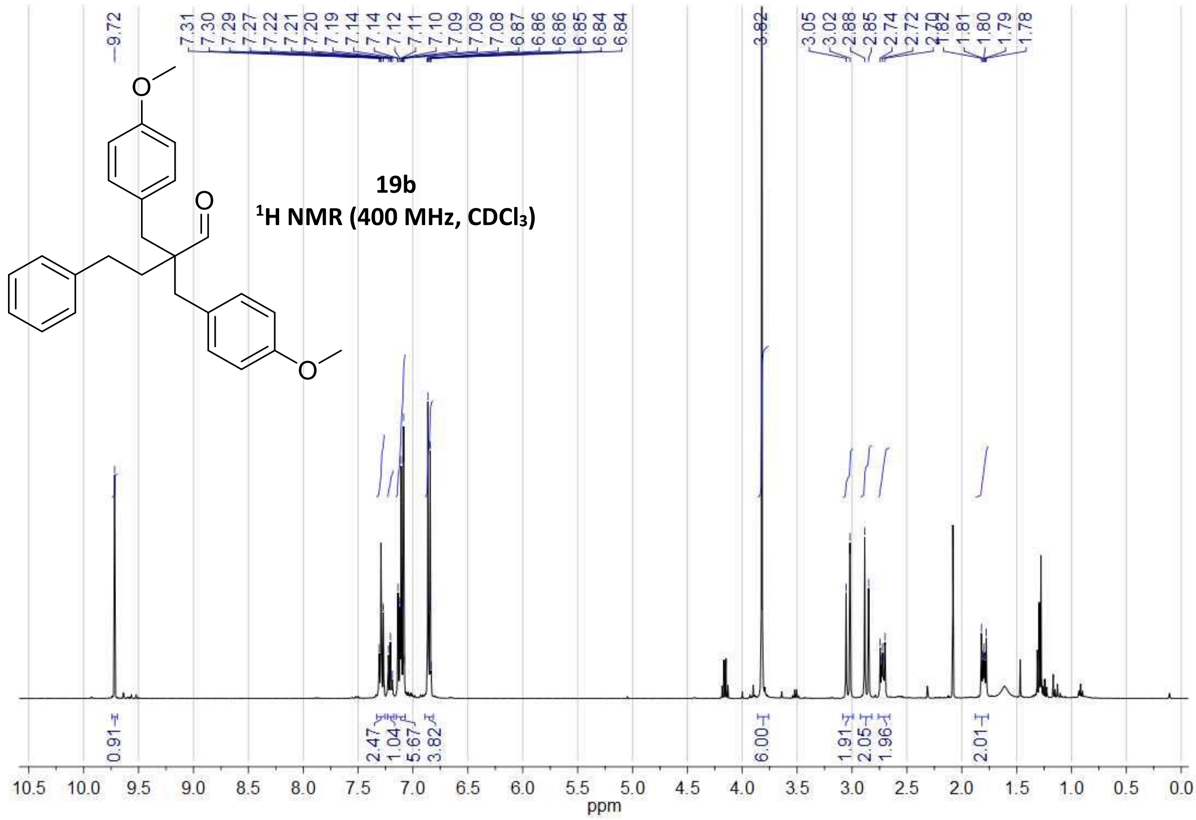
19b
^{1}H NMR (400 MHz, CDCl$_3$)
9.72
7.31 7.30 7.29 7.27 7.22 7.21 7.20 7.19 7.14 7.12 7.11 7.10 7.09 7.09 7.08 6.87 6.86 6.86 6.85 6.84 6.84
3.82
3.05 3.02 2.88 2.85 2.74 2.72 2.70 1.82 1.81 1.80 1.79 1.78
0.91 2.47 1.04 5.67 3.82 6.00 1.91 2.05 1.96 2.01
ppm

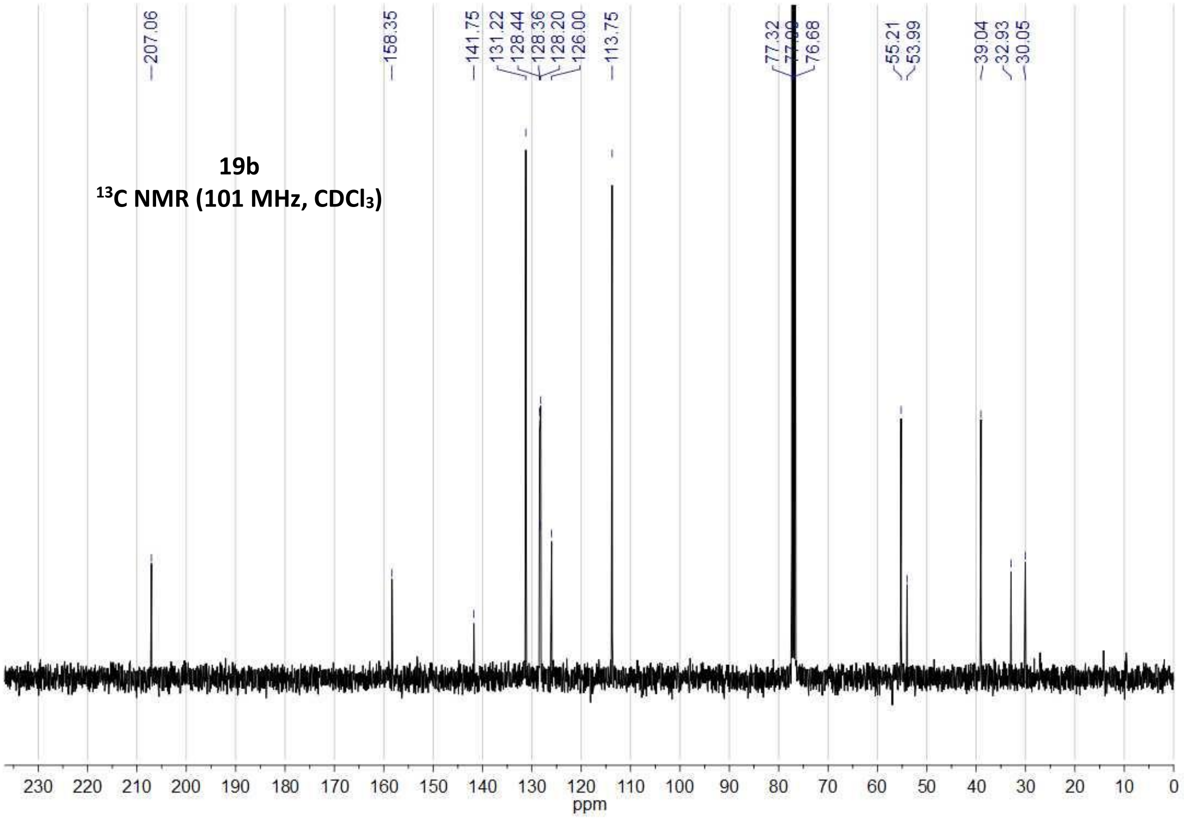
19b
^{13}C NMR (101 MHz, CDCl$_3$)
207.06
158.35
141.75 131.22 128.44 128.36 128.20 126.00 113.75
77.32 77.00 76.68
55.21 53.99
39.04 32.93 30.05
ppm

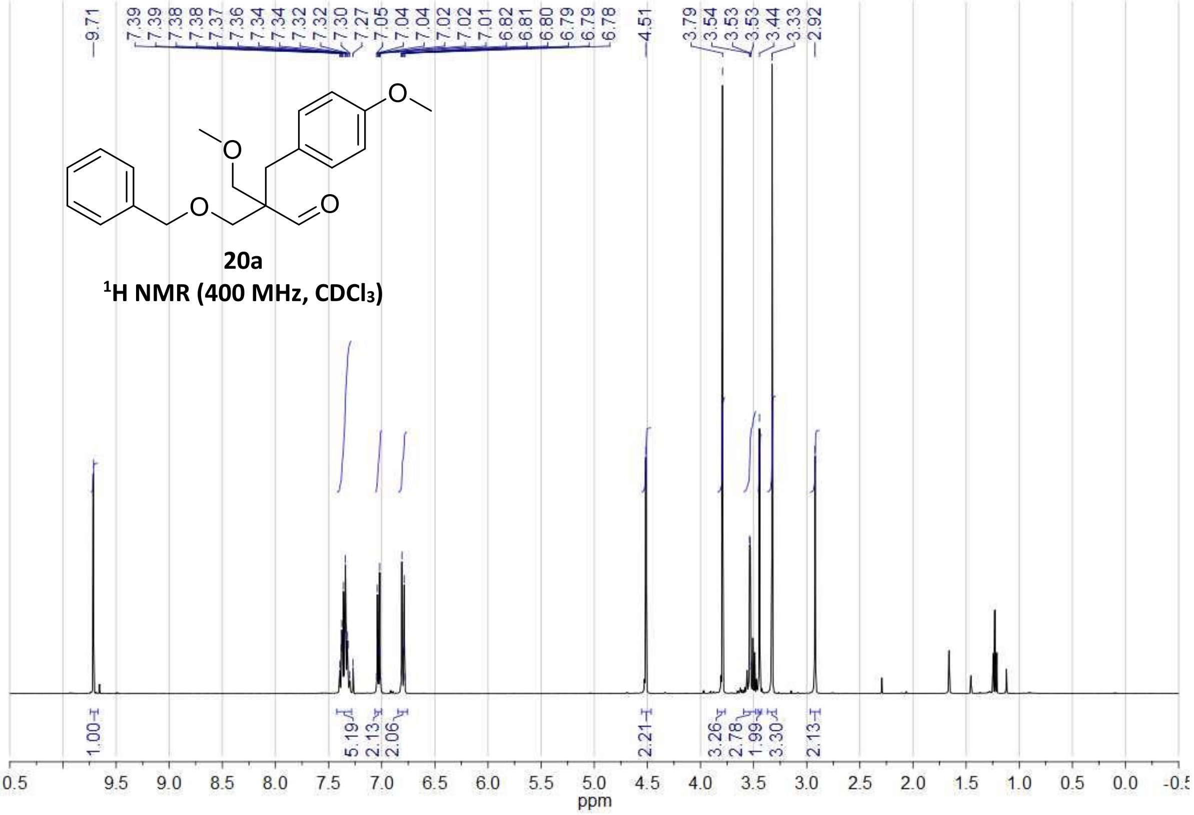
9.71
7.39 7.39 7.38 7.38 7.37 7.36 7.34 7.34 7.32 7.32 7.30 7.27 7.05 7.04 7.04 7.02 7.02 7.01 6.82 6.81 6.80 6.79 6.78
4.51
3.79 3.54 3.53 3.53 3.44 3.33 2.92
20a
^{1}H NMR (400 MHz, CDCl$_3$)
1.00 5.19 2.13 2.06 2.21 3.26 2.78 1.99 3.30 2.13
ppm

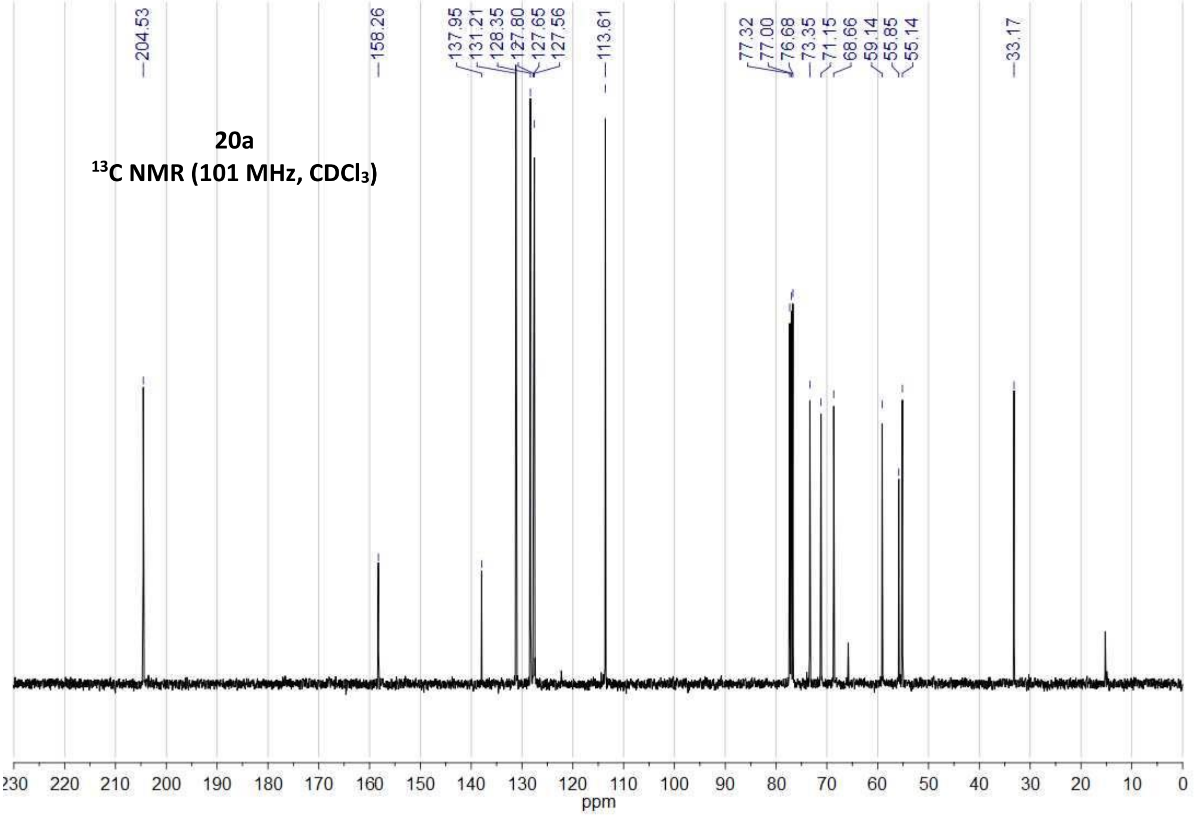
204.53
158.26
137.95 131.21 128.35 127.80 127.65 127.56
113.61
77.32 77.00 76.68 73.35 71.15 68.66 59.14 55.85 55.14
33.17
20a
^{13}C NMR (101 MHz, CDCl$_3$)
ppm

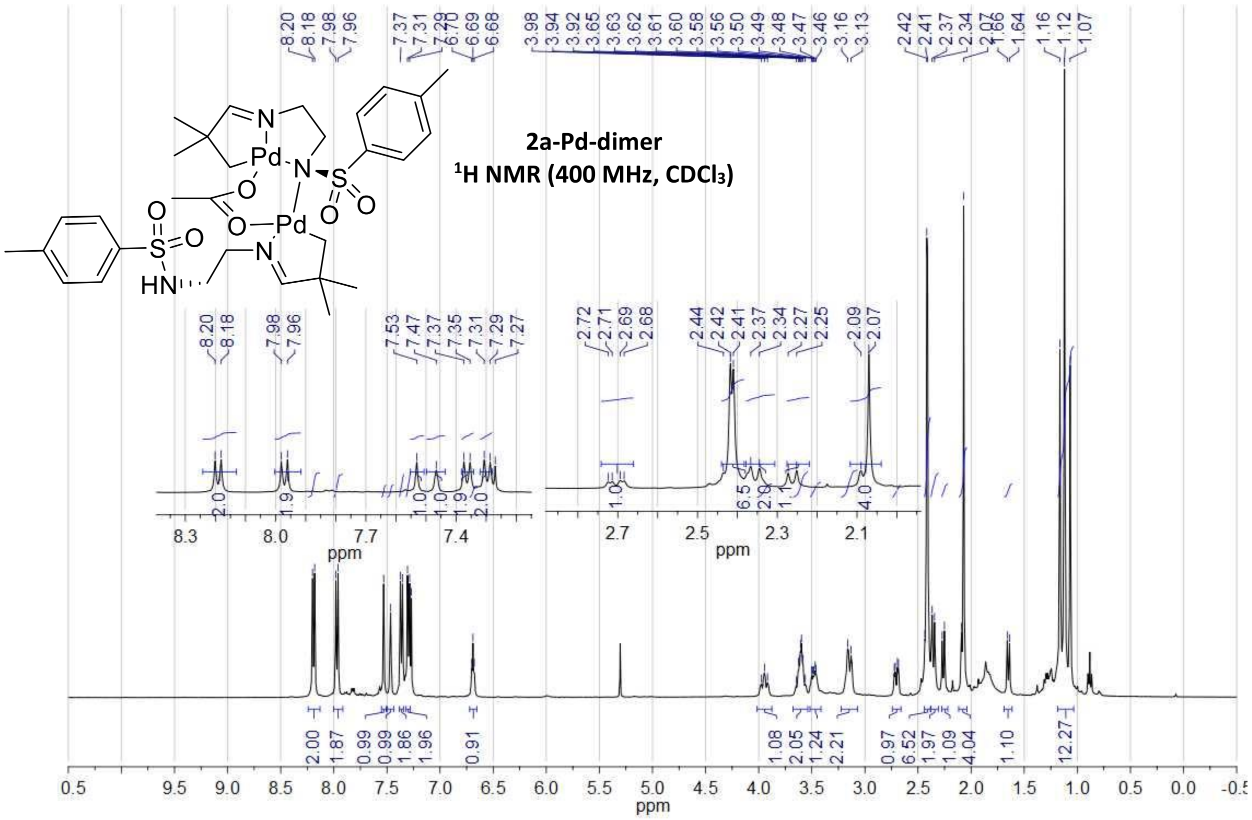
2a-Pd-dimer
1H NMR (400 MHz, CDCl3)
ppm

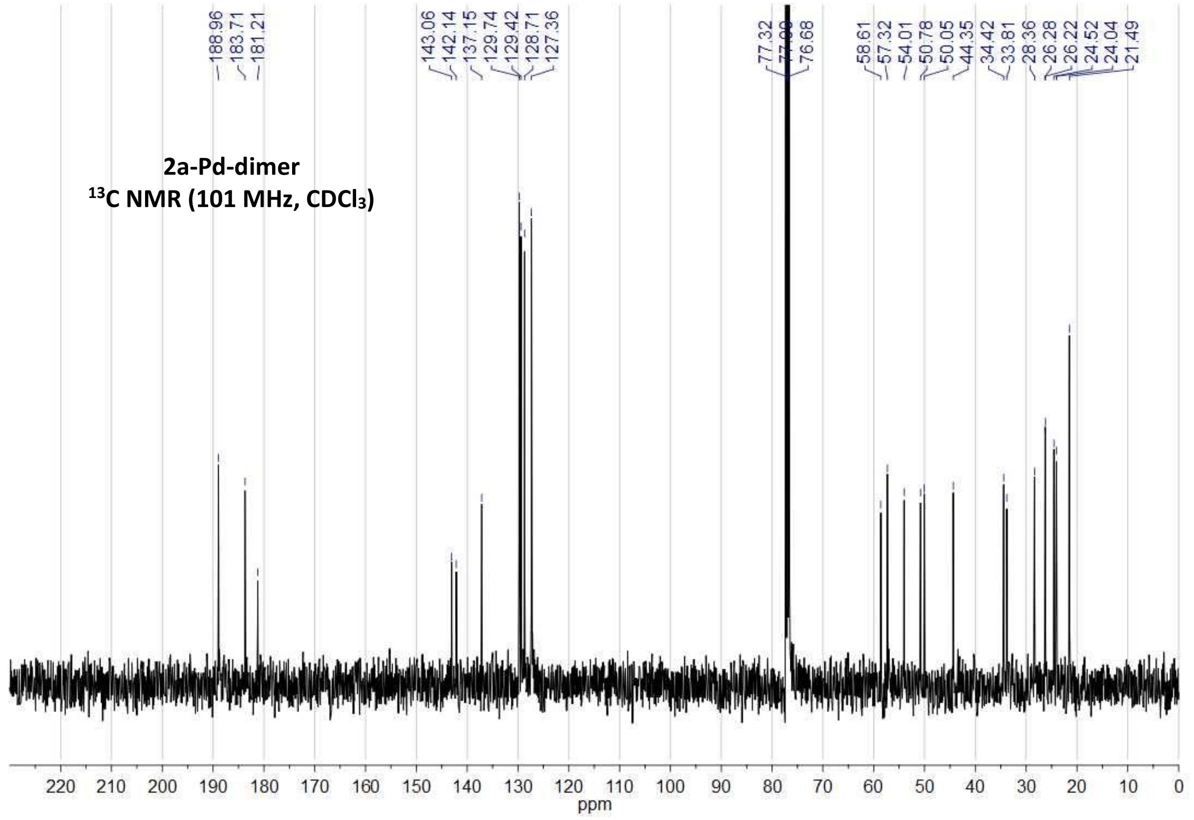
2a-Pd-dimer
13C NMR (101 MHz, CDCl3)
ppm

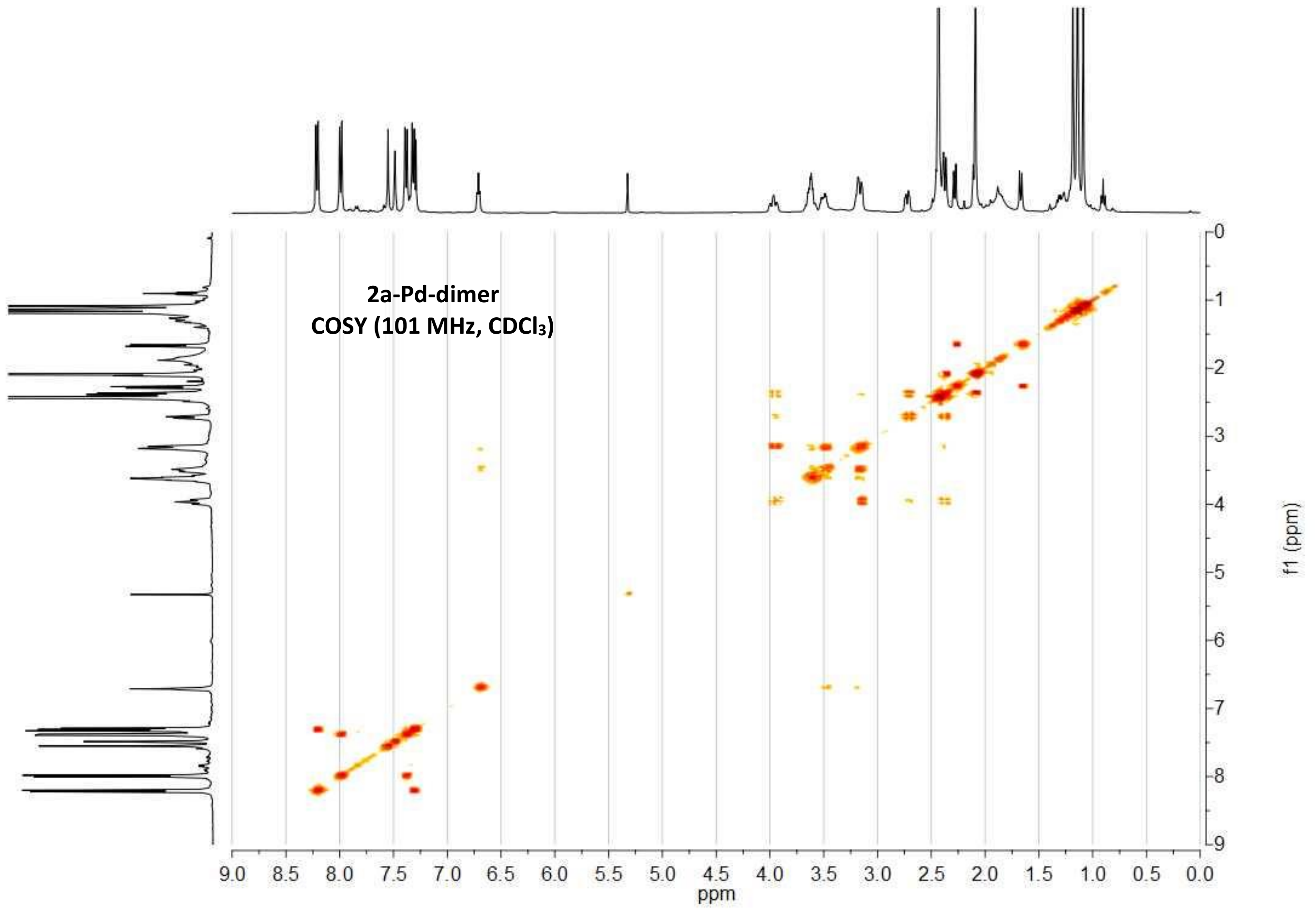
2a-Pd-dimer
COSY (101 MHz, CDCl₃)
f1 (ppm)
ppm

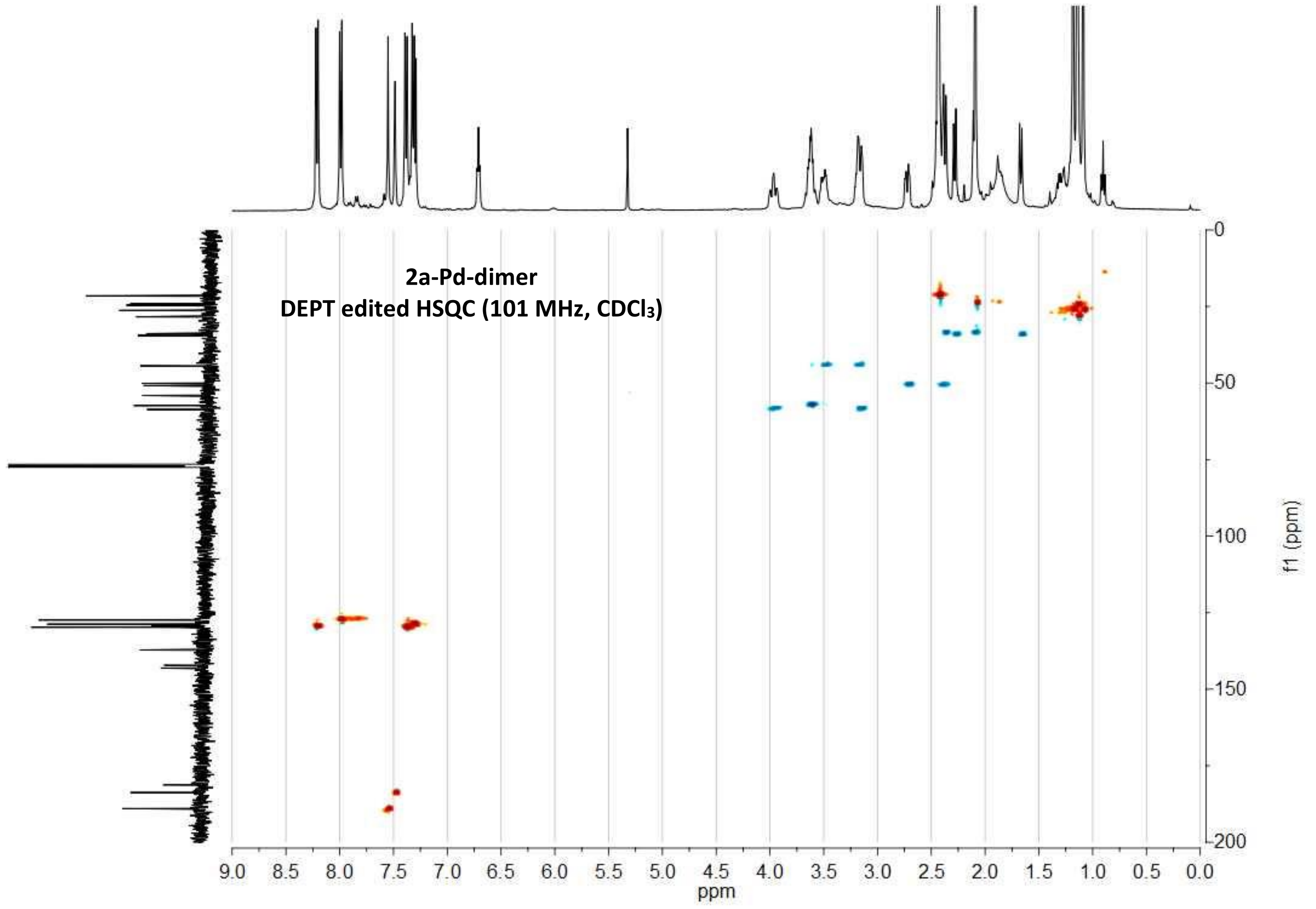
2a-Pd-dimer
DEPT edited HSQC (101 MHz, CDCl₃)
f1 (ppm)
ppm

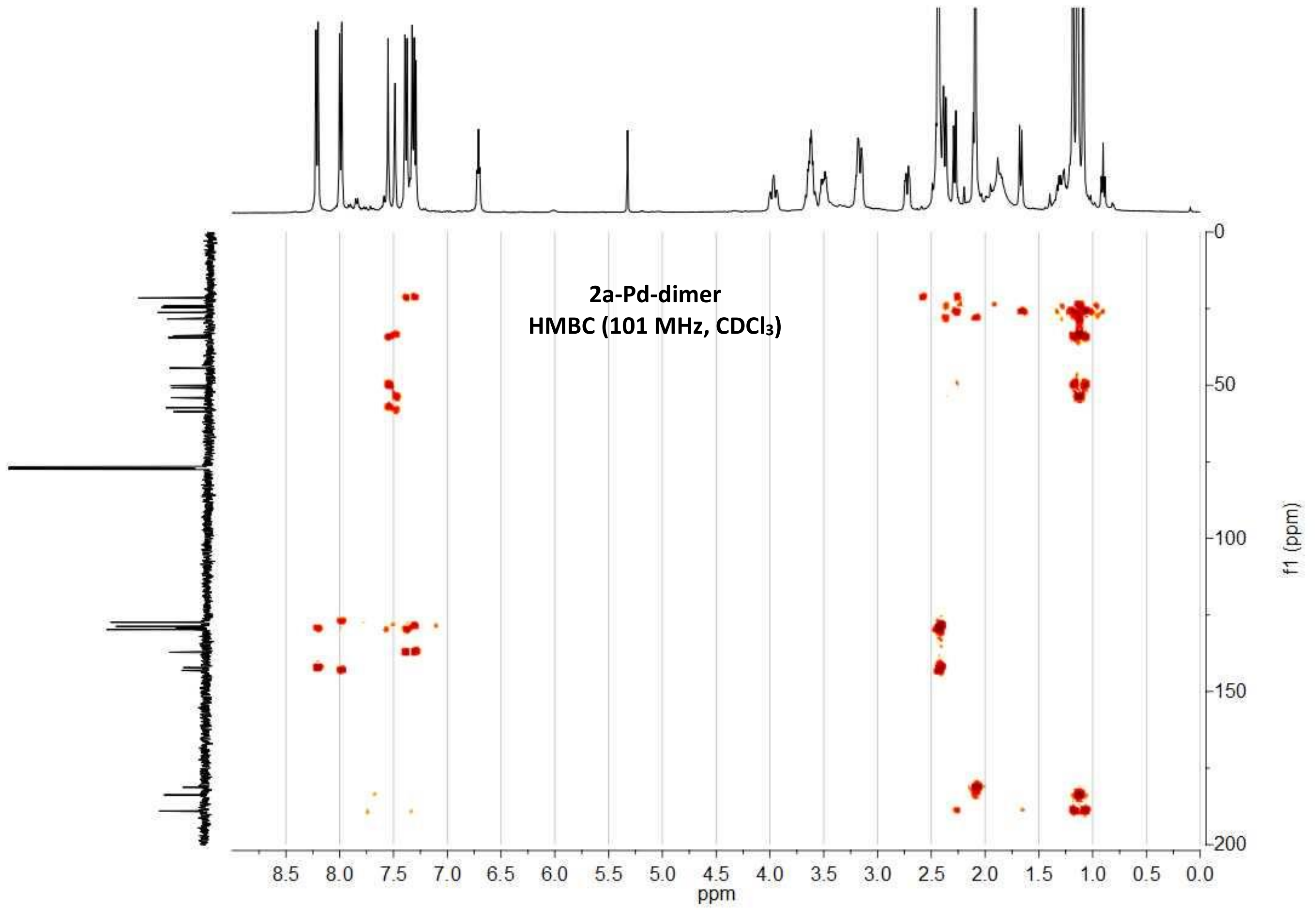
2a-Pd-dimer
HMBC (101 MHz, CDCl₃)
ppm
f1 (ppm)

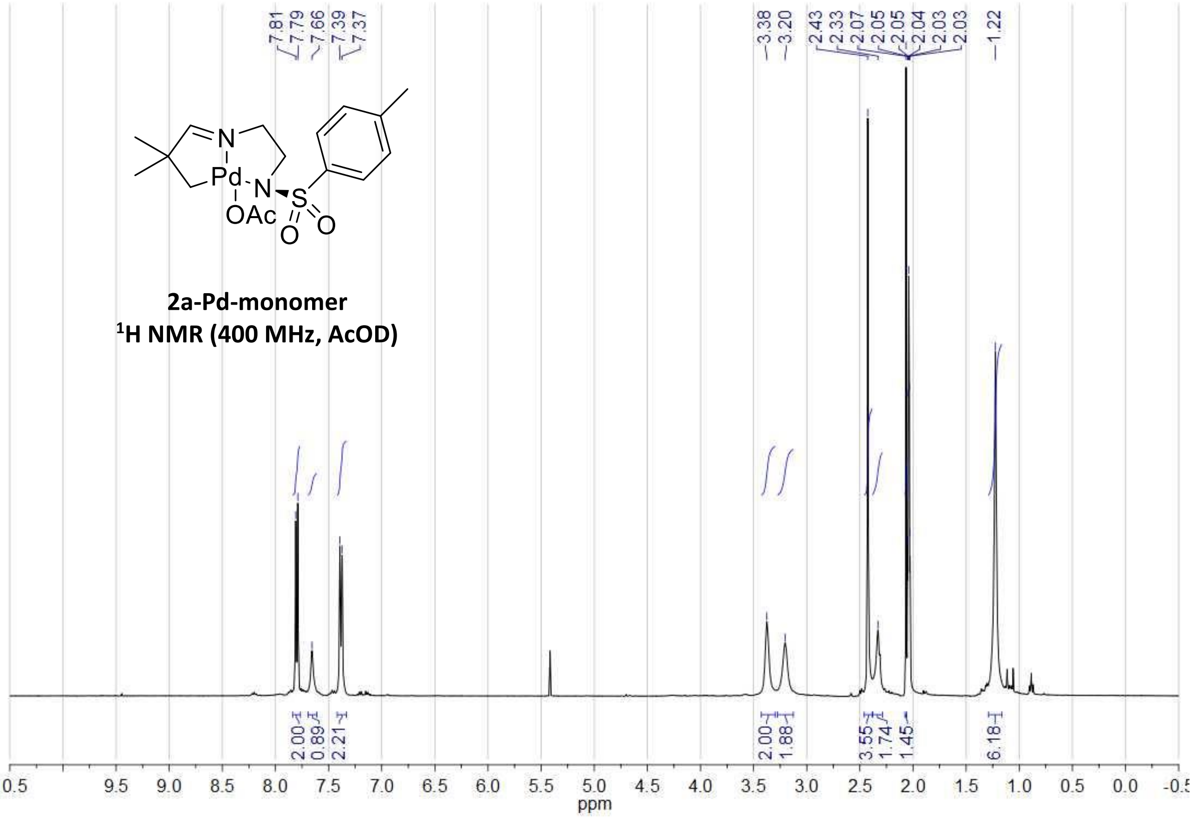
2a-Pd-monomer
1H NMR (400 MHz, AcOD)
7.81 7.79 7.66 7.39 7.37
3.38 3.20
2.43 2.33 2.07 2.05 2.05 2.04 2.03 2.03
1.22
2.00 0.89 2.21
2.00 1.88
3.55 1.74 1.45
6.18
ppm

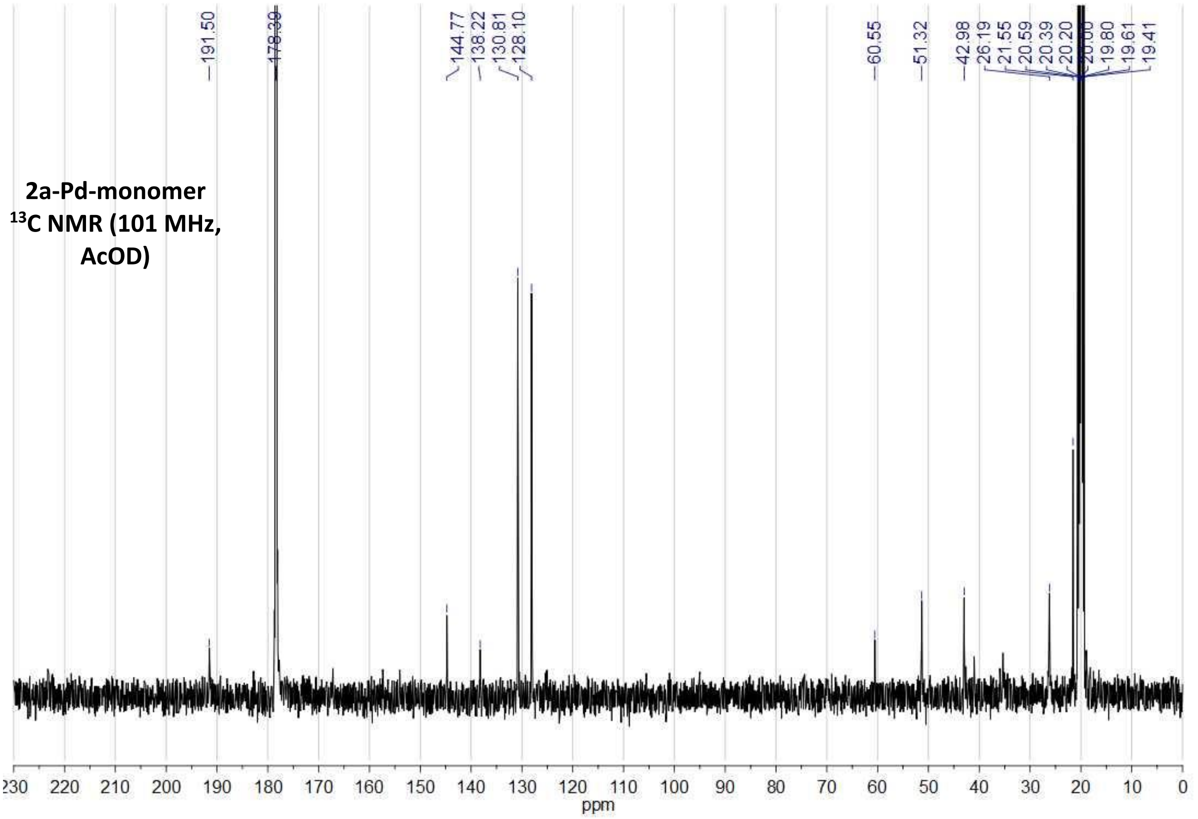
2a-Pd-monomer
13C NMR (101 MHz, AcOD)
191.50
178.39
144.77 138.22 130.81 128.10
60.55
51.32
42.98 26.19 21.55 20.59 20.39 20.20 20.00 19.80 19.61 19.41
ppm
